# Palladium(II)-Catalyzed Nondirected C(sp^2^)–H Alkoxycarbonylation of Arenes

**DOI:** 10.1021/jacsau.5c01351

**Published:** 2025-12-01

**Authors:** Simon Kaltenberger, Joshua Meinshausen, Jyotirmoy Dey, Celia Sánchez-González, Manuel van Gemmeren

**Affiliations:** Otto Diels-Institut für Organische Chemie, 9179 Christian-Albrechts-Universität zu Kiel, Otto-Hahn-Platz 4, 24098 Kiel, Germany

**Keywords:** Alkoxycarbonylation, Palladium Catalysis, Benzoates, C−H Activation, Arenes

## Abstract

Aromatic carboxylic acid derivatives are ubiquitous structural motifs in organic chemistry, which find widespread use as synthetic intermediates or target compounds for various applications. An ideal way to access these compounds is by C–H functionalization of a simple arene with a C1-building block like CO. State of the art methods, however, require the use of prefunctionalized arenes or substrates bearing directing groups, which negatively impact the step economy and/or significantly limit the scope of such processes. Herein we describe Pd-catalysts for the nondirected C–H alkoxycarbonylation of simple arenes. The protocol offers a highly efficient approach toward the synthesis of aromatic esters and enables the functionalization of a broad range of substrates. The predominantly sterically controlled regioselectivity renders the process complementary to electrophilic aromatic substitution reactions. We demonstrate the synthetic versatility of the obtained products to access diverse classes of compounds and present mechanistic studies to derive a plausible reaction mechanism.

## Graphical abstract

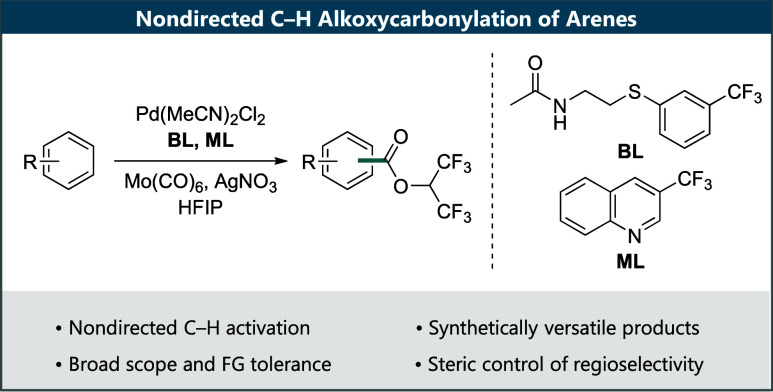

## Introduction

Aromatic carboxylic acid derivatives are ubiquitous structural motifs in organic chemistry, and their efficient synthesis remains of high interest.^1^ One of the most step- and atom-economic ways to access this class of compounds is the direct C–H functionalization of simple arenes with a C1-source.^2^ While CO_2_ would represent an attractive C1-building block for this transformation, inherent reactivity constraints generally confine the scope of such protocols to activated or structurally simple (hetero)­arenes or the use of preformed organometallic reagents.^3,4^ CO, on the other hand, has emerged as a versatile reagent in such transformations, and several strategies have been reported over the last years (Scheme 1). One approach is the use of a directing group (DG), which leads to high regioselectivity (typically *ortho*) and significantly increases the reactivity of a substrate (Scheme 1, Path A).^5,6^ However, the inherent need for an arene bearing a DG limits the scope of the reaction to a single class of substrates. This renders the development of complementary nondirected methods highly desirable. So far, such nondirected methods are limited to multistep protocols encompassing a prefunctionalization of the arene, i.e., through electrophilic aromatic substitution (Scheme 1, Path B).^7,8^ These prefunctionalization steps decrease the step economy of the protocol. Furthermore, the regioselectivities are then determined during the prefunctionalization step and are therefore usually governed by electronic factors favoring the most electron-rich site(s). Nondirected C–H functionalization is a highly attractive yet underdeveloped approach to circumvent these drawbacks.^9^ While preliminary studies have demonstrated the feasibility of such an approach in general, these studies remain limited by requiring an excess of the arene reaction partner and/or substantial chemoselectivity issues limiting the substrate scopes to a narrow range of structural variations.^10^ This spurred our interest to develop a generally applicable method for the nondirected C–H activation/alkoxycarbonylation of diverse arenes under steric control that is capable of suppressing the known side reactions (Scheme 1, Path C). Notably, such a method would complement existing methods and deliver regioisomers that are challenging to obtain through traditional approaches.

**Scheme 1:**
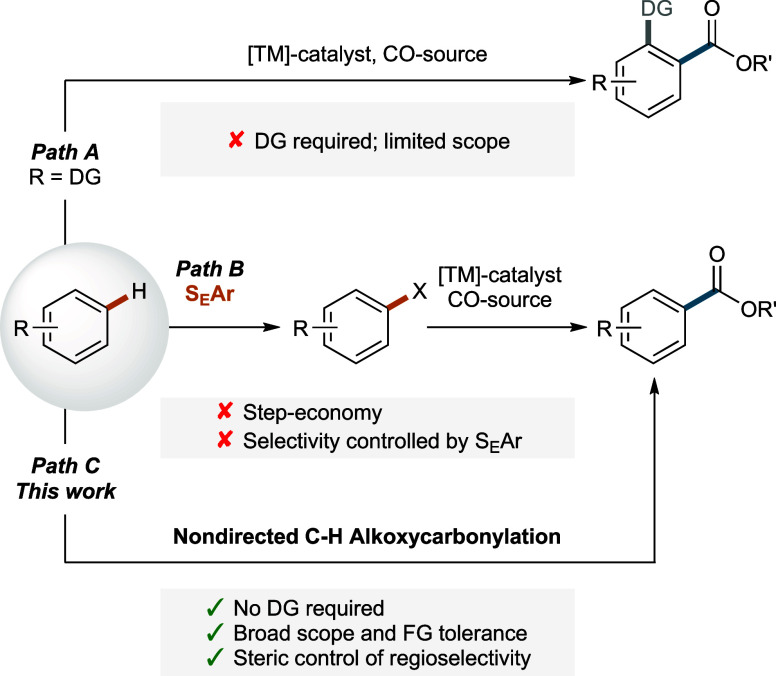
General Strategies for the Alkoxycarbonylation of Simple Arenes with CO

## Results and Discussion

### Reaction Development

We envisioned that the dual-ligand based Pd-catalysts reported by our group^11^ could be combined with a suitable source of CO to develop such a widely applicable method with structurally diverse arenes as limiting reagents under steric control of the regioselectivity. We expected that 1,1,1,3,3,3-hexa­fluoro­isopro­panol (HFIP), commonly used in our reactions, could serve as a solvent to enable C–H activation and at the same time as a nucleophile to deliver the HFIP-esters as final products. Using ethylbenzene (**1a**) as a model substrate and after extensive optimization, we identified a ligand system consisting of X,L-type^12^ thioether based ligand **BL1**, which is a well-established class of ligands for Pd-catalyzed C–H activations of aliphatic substrates,^13^ and 3-trifluoromethylquinoline (**ML1**) as optimal ligands that led to the almost quantitative conversion of **1a** and delivered HFIP-ester **2a** in an overall yield of 82% (Table 1, Entry 1). Mo­(CO)_6_ is used as a stable and convenient source of CO, which is readily available on laboratory scales and does not require specialized infrastructure and the strong precautions that are indespensable when working with gaseous CO (although gaseous CO can, in principle, also be used; see Supporting Information (SI) chapter 6). In contrast to the electronically controlled traditional methods, the high amounts of the *meta* isomer formed in the reaction suggest that the regioselectivity is largely governed by steric factors. Frequently used X,X-type ligands **BL2** and **BL3**, which contain a significantly stronger donor site and therefore should lead to slightly less electrophilic catalysts compared to **BL1**, gave inferior results in this transformation (Table 1, Entries 2 and 3). Notably, the regioselectivity commonly observed with dual-ligand based systems including X,X-type bidentate ligands matches the ones observed herein with the X,L-type ligand **BL1**, which is indicative of an analogous mechanism/selectivity-determining step.^11,14,15^ This observation is also in line with findings from related C–H activation protocols, where these ligand classes have also been found to allow fine-tuning of catalyst systems while staying in the same mechanistic/selectivity-determining regime.^13c,16^ AgNO_3_ is required as the oxidant (Table 1, Entry 4). The nature of the Ag-salt is important, and using AgOAc leads to the formation of side product **3a**, a reactivity that was previously described as a major pathway for sterically nonbiased substrates and has thus prevented the development of a widely applicable C–H alkoxycarbonylation so far (Table 1, Entry 5; for a plausible mechanism leading to the formation of **3**, see SI chapter 6).^10b^ While other solvents proved to be ineffective for the reaction, 2,2,2-trifluoroethanol resulted in the formation of the corresponding 2,2,2-trifluoroethyl ester in moderate yield (Table 1, Entry 6; see SI chapters 2.3 and 4.2 for further details).

**Table 1:**
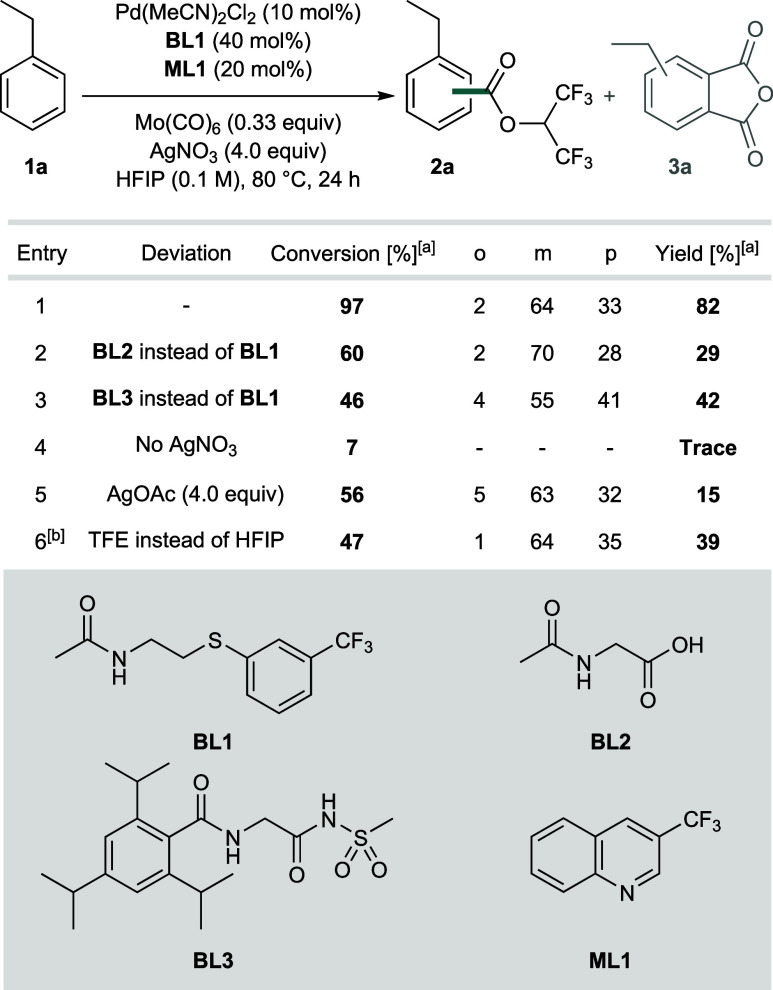
Optimized Reaction Conditions and Control Reactions

a Yields, conversions, and regioselectivities were determined by GC-FID using mesitylene as an internal standard. Reactions were conducted on a 0.1 mmol scale.

b TFE = 2,2,2-trifluoroethanol. In this case, the yield refers to the corresponding 2,2,2-trifluoroethyl ester.

### Scope Studies

With the optimized conditions in hand, we began exploring the scope of our method (Scheme 2). For enhanced purification, certain HFIP-esters **2** were hydrolyzed with aq. NaOH and isolated as carboxylic acids **4**. Benzene (**1b**) could be converted to benzoic acid (**4b**) in an 84% isolated yield. Model substrate ethylbenzene was isolated as **2a** in 68% yield with an m:p ratio of 65:35. Halogenated arenes (**1c** and **1d**) could both be employed and lead to high yields. As in previous reports by our group,^11^ the halide can act as a weak directing group, resulting in the formation of slightly higher amounts of *ortho* isomer. In the case of bromobenzene (**1d**), the formation of a small amount of the *ipso*-substituted isomer suggests that oxidative addition of the catalyst into C–Br bonds can take place under the reaction conditions. TBS-protected phenol **2e** could be isolated in excellent yield with the *meta* isomer as the main product. These findings demonstrate that our catalyst system is highly sensitive to steric control, which suppresses or substantially reduces substitution in *ortho*-sites. Notably, electronic factors are known to generally favor electron-rich sites in related reactions/catalyst systems.^14^ The fact that an almost statistical 2:1 mixture of *meta*- and *para*-products is observed for the above substrates, even in the case of the electronically highly biased product **2e**, demonstrates that our catalyst features a very low sensitivity to electronic effects. Silylated benzene derivative **2f** was isolated in good yield with a nearly statistical distribution of the *meta* and *para* isomer. Biphenyl derivative **4g** was effectively obtained under the reaction conditions. For electron-poor arenes, a slightly higher temperature and higher concentration proved to be beneficial. Under these reoptimized conditions, **4h** and **4i** were obtained in moderate to very good yield. In contrast to the ketone moiety in **4h**, where only a small amount of *ortho* isomer is obtained, the high amount of *ortho* isomer in **4i** suggests that the benzamide moiety serves as a directing group outcompeting the nondirected pathway under the reaction conditions.

**Scheme 2:**
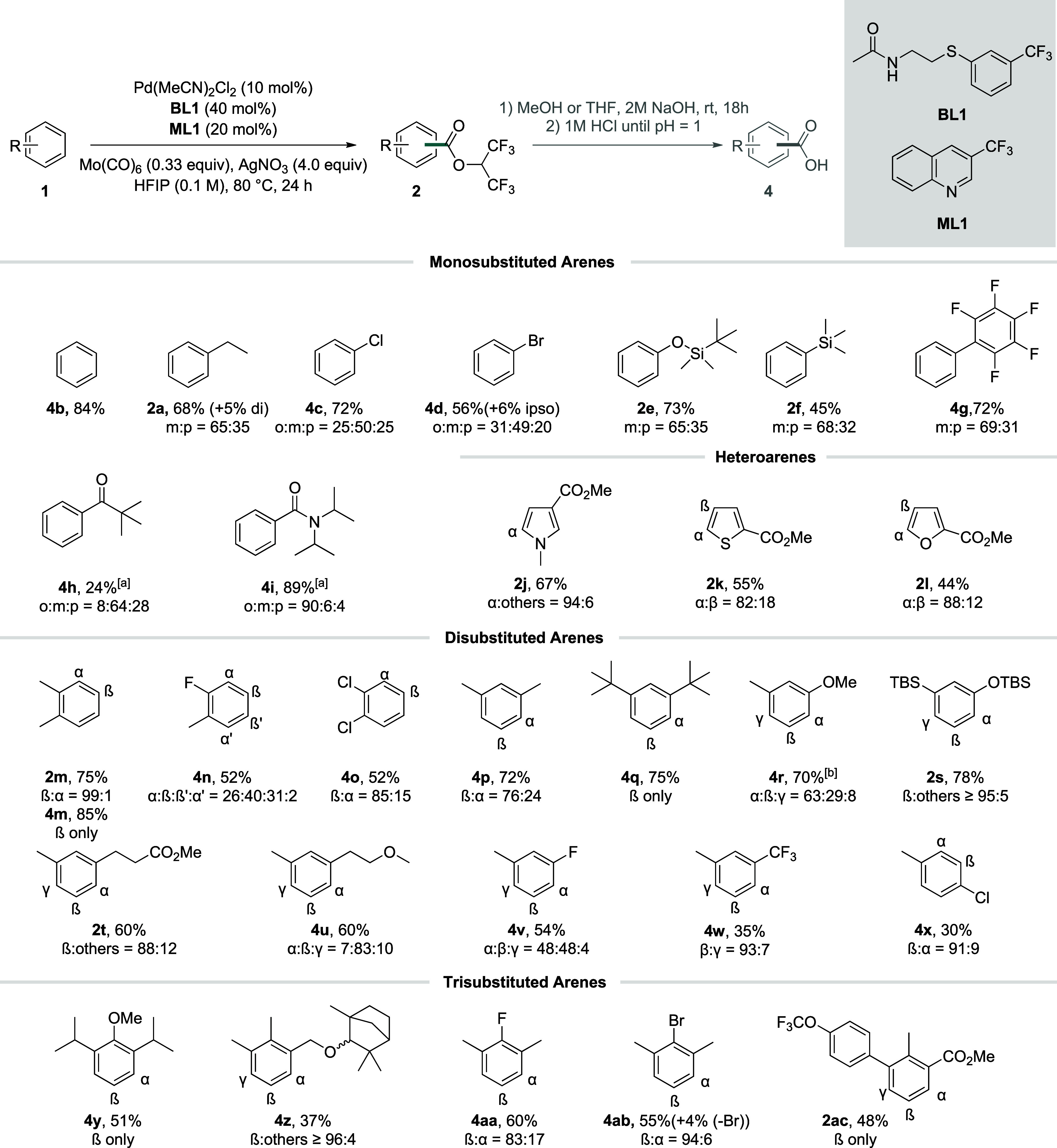
Scope of the Reaction^a^

a Reaction performed at 85 °C in HFIP (1.2 mL).
b Quinoxaline (0.040 mmol) was used as monodentate ligand instead of **ML1**.
c Reaction conditions: substrate **1** (0.20 mmol), Pd­(MeCN)_2_Cl_2_ (0.020 mmol), **BL1** (0.080 mmol), **ML1** (0.040 mmol), Mo­(CO)_6_ (0.066 mmol), AgNO_3_ (0.80 mmol), HFIP (2.0 mL), 80 °C, 24 h.

Five-membered heteroarenes **1j**–**1l** containing an electron-withdrawing ester group gave moderate to high yields with predominant formation of the sterically and electronically preferred α isomer. Since the carbonylation and carboxylation of heteroarenes are comparably well developed,^17^ we did not further extend our scope studies with heteroarene substrates.

We next turned our attention to difunctionalized arenes. Substrates **1m**–**1o** could effectively be employed. As before, the reaction led to the predominant formation of β and β′ isomers, whereas only very little product was formed from sterically hindered positions. 1,3-Disubstituted arenes **1p** and **1q** provided very high yields and selectivity. The comparison of these two examples illustrates a general trend observed during the scope studies. While even small methyl groups substantially reduce the level of functionalization in their adjacent positions, larger substituents, such as the ^*t*^Bu-groups in **1q**, fully suppress the reactivity in such positions. **1r**–**1u** gave high yields and preferential functionalization in the sterically less hindered β position. Only for compound **1r** bearing a very electron-rich and sterically little hindered position adjacent to the OMe-group was the formation of the electronically preferred α-functionalized product observed as the main isomer in **4r**. This is in sharp contrast to that of product **2s** carrying an OTBS-group, where the α position is barely functionalized. **2s** further highlights the high degree of steric control of our catalyst system capable of overcoming electronic effects. **1v**, containing a halide substituent, was functionalized to significant degrees in α and β positions, while **1w** was functionalized with high selectivity for the sterically and electronically preferred β position. As expected, 1,4-disubstituted arene **1x** could also be employed in the reaction but only gave a moderate yield with the reaction taking place mostly *ortho* to the chloride substituent.

1,2,3-Trisubstituted propofol derivative **1y** and fenchol derivative **1z** gave moderate to good yields and almost exclusive formation of the sterically less hindered β isomer. Halogenated compounds **1aa** and **1ab**, as well as sonidegib precursor **1ac**, were equally well suited for the reaction. As shown in compound **4ab**, even sterically hindered C–Br is not completely inert during the reaction conditions, albeit dehalogenation occurred to a lesser extent than in **1d**.

Taken together, the following trends can be deduced. The transformation prefers sterically accessible sites, resulting in excellent selectivity for one isomer in a majority of 1,2- or 1,3-disubstituted and 1,2,3-trisubstituted arenes. Heteroatoms with lone-pairs directly attached to the arene, such as halides or methoxy groups, increase the functionalization in adjacent sites. Substrates in which specific positions are both sterically and electronically preferred, like in **4w**, display an improved selectivity.

It should also be noted that in selected cases such as **4r**, or when performing the reaction on a larger scale (see below), the regioisomers could be separated by simple column chromatography. Notably, it has been demonstrated in related studies that pure regioisomers can reliably be obtained by preparative HPLC purification, a routine technique when preparing compound libraries, for example, in medicinal chemistry laboratories.^18^

### Transformations of HFIP-Esters and Scale-Up Reactions

As evidenced by the scope of the reaction, we have developed a broadly applicable and high yielding system to obtain aromatic HFIP-esters. In the next step, we set out to further probe the synthetic utility of our protocol (Scheme 3).

**Scheme 3:**
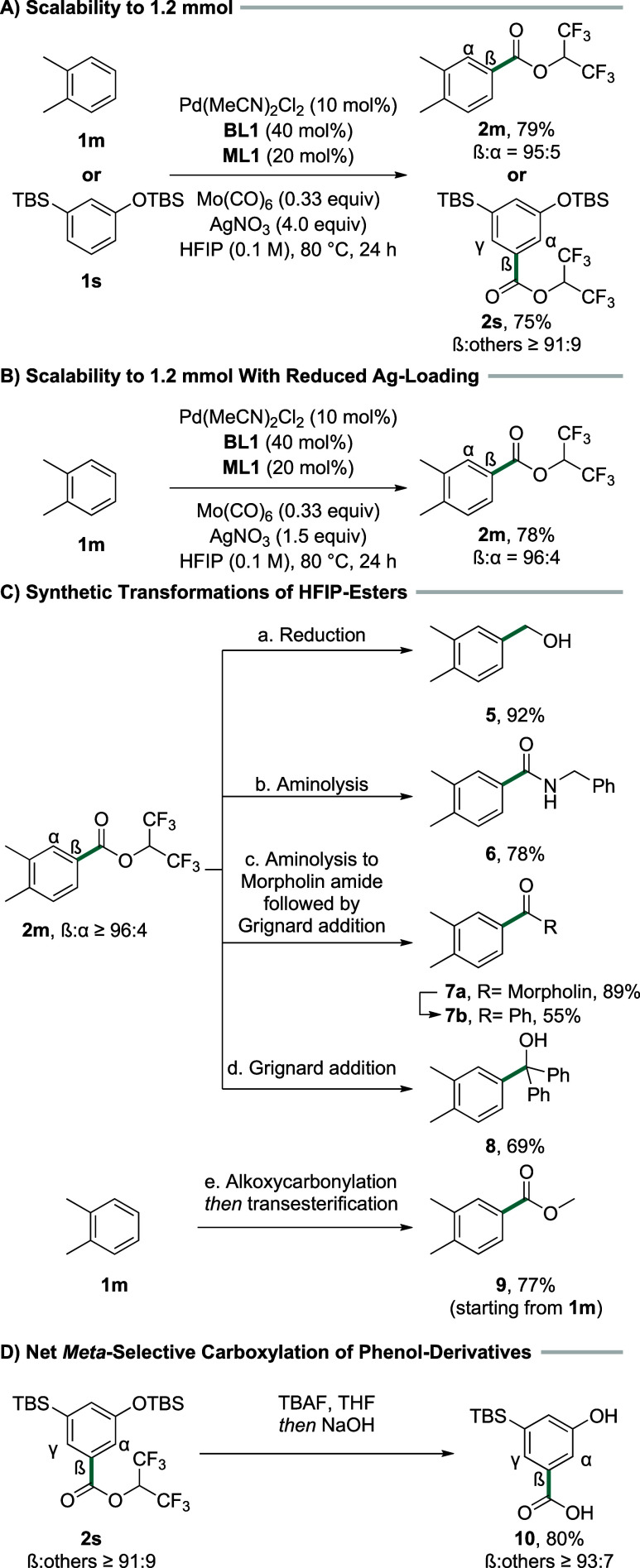
(A) Scalability of the Reaction to 1.2 mmol; (B) Scalability of the Reaction with Reduced Ag-Loading; (C) Synthetic Transformations of HFIP-Esters;^a^ and (D) Net *Meta*-Selective Carboxylation of Phenol Derivatives^b^

a (a) LiAlH_4_ (1.0 equiv), Et_2_O (0.1 M), 0 °C → rt, 3 h; (b) benzylamine (1.0 equiv), K_3_PO_4_ (1.0 equiv), THF (2 M), 90 °C, 22 h; (c) **7a**: morpholine (1.0 equiv), K_3_PO_4_ (1.0 equiv), THF (2 M), 90 °C, 22 h, **7b**: PhMgBr (4.0 equiv), THF:Et_2_O (0.05 M), 0 °C → rt, 6 h; (d) PhMgBr (6.0 equiv), THF:Et_2_O (0.07 M), 60 °C, 20 h; (e) Pd­(MeCN)_2_Cl_2_ (10 mol %), **BL1** (40 mol %), **ML1** (20 mol %), Mo­(CO)_6_ (0.33 equiv), AgNO_3_ (4.0 equiv), HFIP (0.1 M), 80 °C, 24 h, then NEt_3_ (10 equiv), MeOH (0.05 M), 65 °C, 72 h.
b Tetra-*N*-butylammonium fluoride (TBAF, 3.0 equiv), THF (0.1 M), rt, 1 h, then aq. NaOH (2 M):THF = 1:1 (0.05 M), rt, 24 h.

First, we tested whether the reaction could be conducted on a larger scale. We performed the reaction using **1m** and **1s** on a 1.2 mmol scale and obtained the corresponding products **2m** and **2s** in virtually the same yields and selectivities as on the 0.2 mmol scale used during scope studies (Scheme 3A). However, in contrast to the comparably small amounts required during the scope studies, the use of multiple equivalents of AgNO_3_ is a major drawback when performing the reaction on a larger scale such that we opted to reduce the use of AgNO_3_ to the minimum possible amount (see the SI for further details). After further screening, we were able to conduct the reaction on a 1.2 mmol scale with only 1.5 equiv of AgNO_3_ without impairing the results, as evidenced by the carbonylation of **1m** (Scheme 3B). Next, we probed the reactivity of the obtained HFIP-ester moieties. Treatment of **2m** with LiAlH_4_ in Et_2_O afforded the corresponding benzylic alcohol **5** in 92% yield (Scheme 3C,a). The higher reactivity of the HFIP-ester compared to that of normal aliphatic esters is evidenced by the mild and efficient aminolysis of this functional group.^19^ Treating **2m** with benzylamine (Scheme 3C,b) or morpholine (Scheme 3C,c) in the presence of K_3_PO_4_ afforded the products **6** or **7a** in 78% or 89% yield, respectively. Subsequent treatment of morpholine-amide **7a** with PhMgBr yielded benzophenone derivative **7b** in 55% yield.^20^ Direct treatment of **2m** with PhMgBr, on the other hand, led to the formation of tertiary alcohol **8** in 69% yield (Scheme 3C,d). We also tested whether the HFIP-ester can be converted to other esters through transesterification. When exposing **1m** to the standard reaction conditions for alkoxycarbonylation, followed by treatment with NEt_3_/MeOH, the corresponding methyl ester **9** could be obtained in 77% yield over two steps (Scheme 3C,e).

Lastly, we exploited the remarkable steric control of our protocol. Reacting **2s** with TBAF, followed by aq. NaOH, led to deprotection of the OTBS-group and hydrolysis of the HFIP-ester moiety (Scheme 3D), affording phenol **10** in 80% yield (60% over 2 steps starting from **1s**) with the sterically least hindered β isomer as an almost exclusive product. Taken together, this synthetic sequence represents a net *meta*-selective C–H carboxylation of protected phenols, which overrides strong electronic preferences in the substrate and gives access to the sterically preferred product.

### Mechanistic Studies

To gain insights into the underlying reaction mechanism, experiments with ex situ generated gaseous CO in a two-chamber system were performed.^21^ These experiments revealed several distinct mechanistic roles for AgNO_3_ and Mo­(CO)_6_ (Table 2). First, AgNO_3_ is needed to generate the active catalyst by halide abstraction from Pd­(MeCN)_2_Cl_2_ (Entries 2 and 4). It is also needed to reoxidize low-valent Pd-species in the catalytic cycle, although Entry 4 suggests that catalyst turnover could also be feasible via a silver-free yet inefficient reoxidation pathway, e.g. involving atmospheric oxygen^22^ or NO_3_^–^.^23^ Mo­(CO)_6_ is solely a source of CO under the reaction conditions, and neither Mo­(CO)_6_ nor AgNO_3_ are involved in the selectivity-determining step, as evidenced by the virtually same regioselectivities in AgNO_3_- and Mo­(CO)_6_-free reactions. These findings are further supported by reactions with other CO-sources, stoichiometric experiments, reactions with gaseous CO, and alternative oxidants (see SI chapter 6 for full details).

**Table 2:**
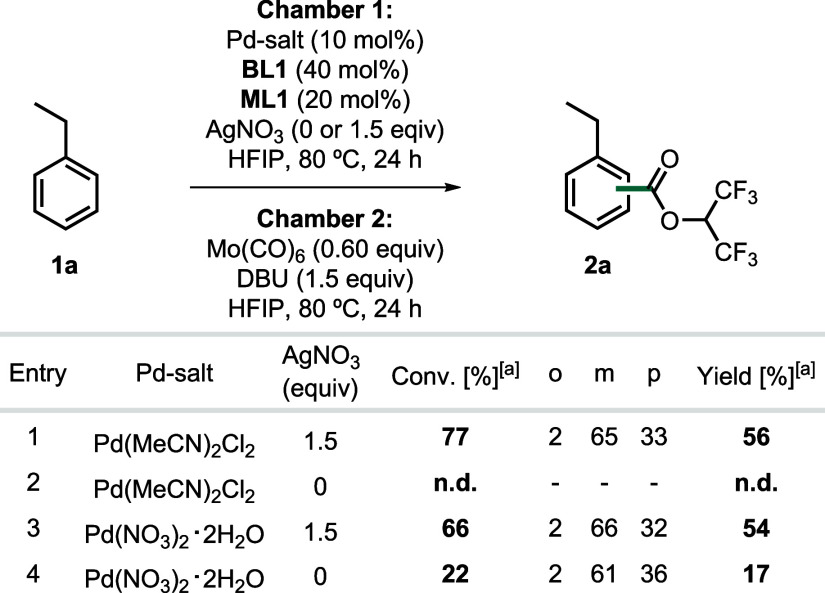
Carbonylation Reactions Using Ex Situ Generated CO Gas

a Yields, conversions, and regioselectivities were determined by GC-FID using mesitylene as an internal standard. Reactions were conducted on a 0.1 mmol scale. DBU = 1,8-diazabicyclo[5.4.0]­undec-7-en.

Based on these experiments, the extensive mechanistic studies on our dual-ligand based Pd-catalysts,^14^ and mechanistic knowledge on Pd-catalyzed carbonylation,^24^ a plausible reaction mechanism is shown in Scheme 4.

**Scheme 4:**
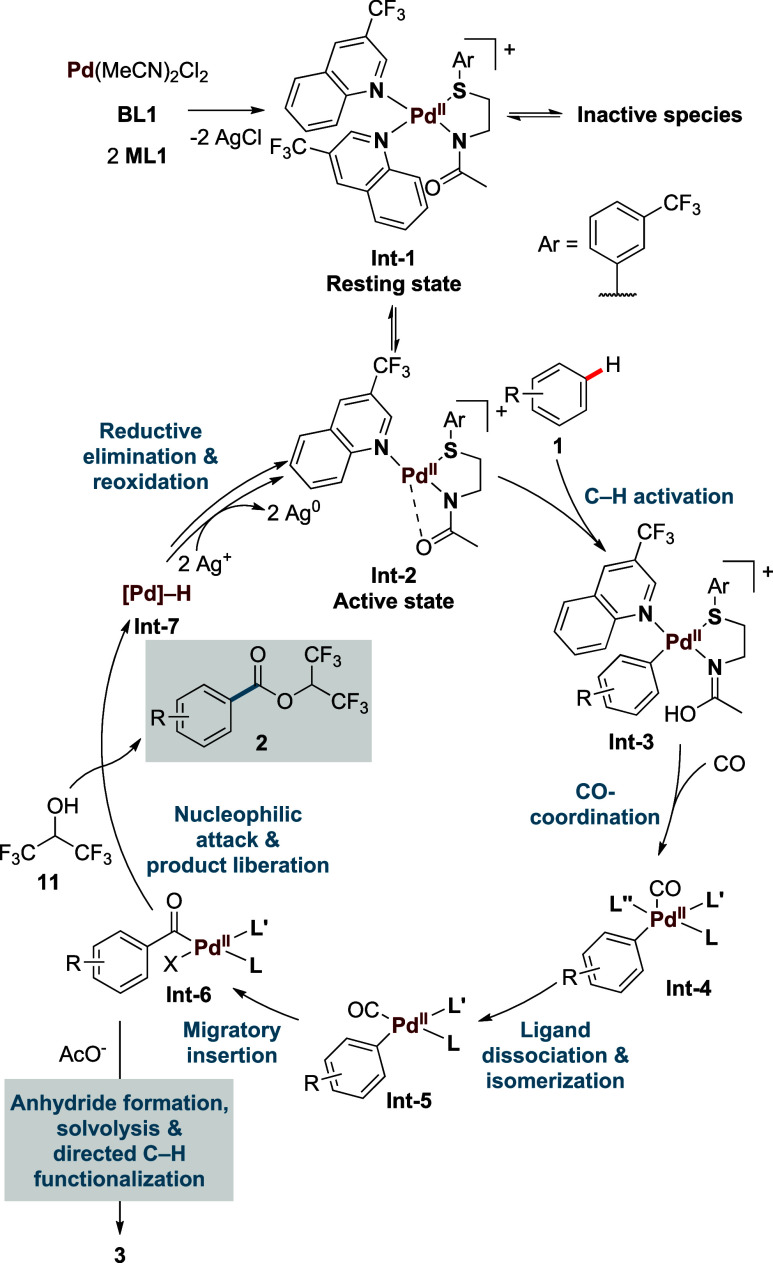
Proposed Mechanism of the Reaction

First Pd­(MeCN)_2_Cl_2_ forms a complex with the respective ligands in a stoichiometry of Pd:**BL1**:**ML1** = 1:1:2 (**Int-1**) and precipitation of 2 equiv of AgCl. From the inactive resting state **Int-1** the active catalyst is generated by the dissociation of one **ML1** to form **Int-2**. This can undergo C–H activation with the starting material **1** to generate the aryl-palladium species **Int-3**. From here an association of CO to form a penta-coordinated complex **Int-4** followed by dissociation of a ligand and isomerization to form **Int-5** is expected to take place.^24^ Alternatively, CO could also coordinate to Pd in a dissociation–association pathway, meaning that ligand dissociation occurs before CO-binding. In this case, no penta-coordinated species are involved. Acyl-Pd complex **Int-6** is subsequently formed by migratory insertion. Nucleophilic attack by HFIP (**11**) liberates product **2** and forms **Int-7**.^25^ From **Int-7**, reductive elimination to Pd^0^ and reoxidation by Ag^+^ regenerates the active catalyst **Int-2**. Additionally, AgNO_3_ could be needed to oxidize low-valent Mo complexes that would otherwise be detrimental to the reaction (also see SI chapter 6).^26^

If a competing nucleophile (i.e., acetate from AgOAc) is present, another pathway becomes feasible from **Int-6** that leads to the formation of side product **3**. Acetate is expected to outcompete HFIP (**11**) as a nucleophile and lead to the formation of mixed anhydrides, a reactivity profile that has already been described by Fujiwara.^10a,27^ Solvolysis to the corresponding benzoic acid, followed by directed C–H functionalization, similar to a report by Yu et al.,^5a^ then leads to the formation of the phthalic anhydride derivative **3** (see SI chapter 6 for more details). Consequently, avoiding the use of competing nucleophiles leads to a more efficient catalytic system.

## Conclusion

In summary, we have developed a highly efficient method for the nondirected C–H alkoxycarbonylation of simple arenes, which proceeds in a single step with the arene as the limiting reagent and does not require the use of directing groups in the substrate. The Pd based catalyst system consists of an X,L-type thioether-derived ligand in combination with a monodentate ligand. AgNO_3_ serves as oxidant in this transformation, and Mo­(CO)_6_ can be used as a convenient source of CO for lab-scale purposes. The developed protocol tolerates a large variety of functional groups and affords synthetically versatile HFIP-esters in high yields under predominantly sterically controlled regioselectivity, giving access to regioisomers that are challenging to obtain through traditional approaches. The demonstrated scalability and downstream derivatizations further highlight the practical utility and synthetic versatility of this reaction. Mechanistic studies have uncovered the distinct roles of the reaction components, leading to a well-founded mechanistic proposal, and show that the reaction can also be performed with gaseous CO. We expect the method to be a highly useful addition to the existing synthetic toolbox that provides valuable compounds with unique substitution patterns.

## Supplementary Material



## Notes

Additional experimental details, screening data, methods, characterization of isolated compounds, mechanistic studies, and NMR spectra (PDF)

## References

[1] Goossen L. J., Rodríguez N., Goossen K. (2008). Carboxylic acids as
substrates in homogeneous catalysis. Angew.
Chem., Int. Ed..

[2] Kuhl N., Hopkinson M. N., Wencel-Delord J., Glorius F. (2012). Beyond directing groups: transition-metal-catalyzed
C-H activation of simple arenes. Angew. Chem.,
Int. Ed..

[3] Sakakibara K., Yamashita M., Nozaki K. (2005). An efficient Pd­(II)-based catalyst
system for carboxylation of aromatic C-H bond by addition of a phosphenium
salt. Tetrahedron Lett..

[4] Zhang W., Lü X. (2012). Synthesis of Carboxylic Acids and
Derivatives Using CO_2_ as Carboxylative Reagent. Chin. J. Catal..

[5] Giri R., Yu J.-Q. (2008). Synthesis of 1,2-
and 1,3-dicarboxylic acids via Pd­(II)-catalyzed carboxylation of aryl
and vinyl C-H bonds. J. Am. Chem. Soc..

[6] Gadge S. T., Gautam P., Bhanage B. M. (2016). Transition
Metal-Catalyzed
Carbonylative C-H Bond Functionalization of Arenes and C­(sp^3^)-H Bond of Alkanes. Chem. Rec..

[7] Brennführer A., Neumann H., Beller M. (2009). Palladium-catalyzed
carbonylation reactions of aryl halides and related compounds. Angew. Chem., Int. Ed..

[8] de Jesus R., Dey J., Chakraborty E., van Gemmeren M. (2025). Sterically Controlled C­(sp^2^)-H Carboxylation
and Formylation: A Complementary Strategy to S_E_Ar Approaches. Org. Lett..

[9] Wedi P., van Gemmeren M. (2018). Arene-Limited
Nondirected C-H Activation of Arenes. Angew.
Chem., Int. Ed..

[10] Jia C., Kitamura T., Fujiwara Y. (2001). Catalytic
functionalization of arenes and alkanes via C-H bond activation. Acc. Chem. Res..

[11] Kaltenberger S., van Gemmeren M. (2023). Controlling Reactivity and Selectivity in the Nondirected
C-H Activation of Arenes with Palladium. Acc.
Chem. Res..

[12] Meng G., Wang Z., Chan H. S. S., Chekshin N., Li Z., Wang P., Yu J.-Q. (2023). Dual-Ligand Catalyst for the Nondirected
C-H Olefination of Heteroarenes. J. Am. Chem.
Soc..

[13] Zhuang Z., Yu C.-B., Chen G., Wu Q.-F., Hsiao Y., Joe C. L., Qiao J. X., Poss M. A., Yu J.-Q. (2018). Ligand-Enabled β-C­(sp^3^)-H Olefination of Free Carboxylic Acids. J. Am. Chem. Soc..

[14] Wedi P., Farizyan M., Bergander K., Mück-Lichtenfeld C., van Gemmeren M. (2021). Mechanism
of the Arene-Limited Nondirected C-H Activation
of Arenes with Palladium. Angew. Chem., Int.
Ed..

[15] Bairagi Y., Porey S., Vummaleti S. V. C., Zhang X., Lahiri G. K., Maiti D. (2024). Synthesis of β
- (Hetero)­aryl Ketones via Ligand-Enabled Nondirected C-H Alkylation. ACS Catal..

[16] Wu K., Lam N., Strassfeld D. A., Fan Z., Qiao J. X., Liu T., Stamos D., Yu J.-Q. (2024). Palladium
(II)-Catalyzed C-H Activation with Bifunctional Ligands: From Curiosity
to Industrialization. Angew. Chem., Int. Ed..

[17] Banerjee A., Dick G. R., Yoshino T., Kanan M. W. (2016). Carbon
dioxide utilization via carbonate-promoted C-H carboxylation. Nature.

[18] Zhao D., Xu P., Ritter T. (2019). Palladium-Catalyzed Late-Stage Direct Arene Cyanation. Chem..

[19] Caldwell N., Jamieson C., Simpson I., Watson A. J. B. (2015). Catalytic amidation
of unactivated ester derivatives mediated by trifluoroethanol. Chem. Commun..

[20] Martín R., Romea P., Tey C., Urpí F., Vilarrasa J. (1997). Simple and Efficient Preparation of Ketones from Morpholine
Amides. Synlett.

[21] Åkerbladh L., Odell L., Larhed M. (2019). Palladium-Catalyzed
Molybdenum Hexacarbonyl-Mediated
Gas-Free Carbonylative Reactions. Synlett.

[22] Beck E. M., Grimster N. P., Hatley R., Gaunt M. J. (2006). Mild aerobic
oxidative Palladium (II) catalyzed C-H bond functionalization: regioselective
and switchable C-H alkenylation and annulation of pyrroles. J. Am. Chem. Soc..

[23] Gu C.-H., Zhang Z., Shen S.-J., Xu H.-J., Hu Y. (2023). A Cheap and
Efficient Oxidant (^
*n*
^Bu)_4_NNO_3_-Enabled C­(sp^2^)- and C­(sp^3^)-H Olefination
at Room Temperature. Org. Lett..

[24] Anderson G. K., Cross R. J. (1984). Carbonyl-insertion
reactions of square-planar
complexes. Acc. Chem. Res..

[25] Sang R., Hu Y., Razzaq R., Jackstell R., Franke R., Beller M. (2021). State-of-the-art
palladium-catalyzed alkoxycarbonylations. Org.
Chem. Front..

[26] Abbott A.
P., Malkov A. V., Zimmermann N., Raynor J. B., Ahmed G., Steele J., Kočovský P. (1997). Oxidation of Molybdenum(0)
and Tungsten(0) Carbonyl Complexes with Silver Triflate. Organometallics.

[27] Fujiwara Y., Kawauchi T., Taniguchi H. (1980). Palladium-promoted
one-step carboxylation of aromatic compounds with carbon monoxide. J. Chem. Soc., Chem. Commun..

