# Supplementary material for: Palladium(II)-Catalyzed Nondirected C(sp2)–H Alkoxycarbonylation of Arenes
Source: JACS Au. 2025 Dec 1;5(12):6343–50. doi: 10.1021/jacsau.5c01351 (PMC12728605; doi:10.1021/jacsau.5c01351)
Supplement: Supplementary file 1 [file au5c01351_si_001.pdf]

Supporting Information

Palladium(II)-Catalyzed Nondirected C(sp^2)–H Alkoxycarbonylation of Arenes

Simon Kaltenberger‡, Joshua Meinshausen‡, Jyotirmoy Dey‡, Celia Sánchez-González‡ and Manuel van Gemmeren*,‡

‡ Otto Diels-Institut für organische Chemie, Otto-Hahn-Platz 4, 24098 Kiel, Germany

* vangemmeren@oc.uni-kiel.de

Table of contents:

1. General Experimental Methods 2

2. Optimization of the Reaction Conditions 4

2.1. Optimization with Ethylbenzene 4

2.2 Re-Optimization for Arenes Containing an Electron-Withdrawing Group 28

2.3 Screening of Reduced Ag-Amount for Scale-up Reactions and alternative solvents/ ligands 31

3. Synthesis of Starting Materials and Ligands 34

3.1 Synthesis of Authentic Samples 34

3.2 Synthesis of Ligands 36

3.3 Synthesis of Starting Materials 39

4. Scope of the Reaction 42

4.1. Scope shown in the main manuscript 42

4.2. Additional substrates 62

5. Scalability and Follow-up Transformations 64

5.1 Scale-up Reactions 64

5.2 Synthetic Transformations of the HFIP-Ester Moiety 67

5.3 Net meta-Selective Carboxylation of Protected Phenols 71

6. Preliminary Mechanistic Studies 72

7. NMR-Spectra 80

7.1 Authentic Samples 80

7.2 Ligands 87

7.3 Starting Materials 96

7.4 Scope shown in the main mansucript 100

7.5. Additional substrates 144

7.6 Follow-up Transformations 148

7.6.1 Scale-up to 1.2 mmol 148

7.6.2 Synthetic Transformations of the HFIP-Ester Moiety 158

7.6.3 Net meta-Selective Carboxylation of Protected Phenols 164

8. References 165

1. General Experimental Methods

General Information and Solvents

All reactions were conducted in oven-dried glassware (120 °C) under ambient conditions and atmosphere. Reaction temperatures refer to the temperature of the aluminum-block surrounding the reaction vessel.

Commercially available chemicals were obtained from ABCR, Acros Organics, BLD-pharm, Alfa Aesar, Deutero, Eurisotop, Fluorochem, Sigma Aldrich, or TCI Europe and used as received.

1,1,1,3,3,3-hexafluoroisopropanol (HFIP) was purchased from Fluorochem and used as received. The quality of HFIP was crucial for reliable reaction outcomes, so care should be taken to avoid contaminations. Solvents used for column chromatography were distilled prior to use.

$Mo(CO)_6$ was purchased from Fluorochem and stored in a N_2-filled Glovebox. Small batches for short term use were extracted and stored under N_2 in a Schlenk-flask. The compound was weighed in air.

Sodium 1,1,1,3,3,3-hexafluoroisopropanolate (NaHFIP) was prepared as previously described by our group.[1]

Chromatography

Analytical thin layer chromatography (TLC) was performed on silica gel ALUGRAM Xtra SIL G/UV$_{254}$ plates (Macherey-Nagel) or aluminum oxide 150 F$_{254}$, neutral plates (Merck). Compounds were visualized by ultraviolet light (254 nm or 366 nm) or by staining with $KMnO_4$ (1 g $KMnO_4$, 6 g K_2CO_3 and 0.1 g KOH in 100 mL of H_2O). Flash chromatography was performed on silica gel 60M (0.04-0.063 mm) or aluminum oxide (aluminum oxide 90, neutral, activity level 1). Positive overpressure was applied. Automated flash chromatography was performed on a Biotage Isolera One system. Compounds were detected by a UV-detector.

Nuclear Magnetic Resonance (NMR) Spectroscopy

^{1}H, ^{13}C, and ^{19}F-{^{1}H} NMR spectra were recorded at room temperature on a Bruker AvanceNeo 500 or a Bruker Avance 600 device. Chemical shifts (δ) are given relative to tetramethylsilane (TMS) and referenced to residual solvent signals as tabulated by Fulmer et al.[2] Chemical shifts are given with two decimal numbers (^{1}H) or one decimal number (^{13}C, ^{19}F). Data is reported in the following order: Chemical shift (multiplicity [s = singlet, d = doublet, t = triplet, q = quartet, quint = quintet, hept = septet, m = multiplet, br = broad signal], coupling constant (*J* [Hz]) and number of H-atoms). All NMR-spectra were processed using MestReNova.

Infrared spectroscopy (IR) and melting points

IR-spectroscopy was performed on a Perkin Elmer ATR spectrometer. Samples were measured neat. Melting points were determined on a Büchi instrument with automated temperature control. The temperature was raised by 2 °C/ min during measurements.

Mass Spectrometry (MS)

High resolution mass spectra (HRMS) were recorded on a Jeol AccuTOF (EI) or a ThermoFisher Orbitrap (ESI) device.

Gas Chromatography with Flame Ionization Detection (GC-FID)

GC-FID analysis was performed using an Agilent Technologies 7890B or 8860 instrument with an HP5 column (30 m, 0.32mm × 0.25 μm) and hydrogen as carrier gas.

Gas Chromatography with Mass Spectrometry (GC-MS)

GC-MS was performed on an Agilent Technologies 8890 system coupled to an Agilent Technologies 5977B mass detector (EI) and an HP-5MS column (30 m, 0.32mm × 0.25 μm). Helium was used as carrier gas.

2. Optimization of the Reaction Conditions

2.1. Optimization with Ethylbenzene

General procedure during optimization studies: An oven-dried 10 mL Schlenk-flask was charged with a stirring bar, a Pd-salt, a bidentate-ligand **BL**, a monodentate-ligand **ML**, a base and an Ag-salt. $Mo(CO)_6$ followed by 1,1,1,3,3,3-hexafluoroisopropanol (HFIP) were added in quick succession, the flask was tightly sealed and the mixture was stirred at room temperature for approximately 10 min. Ethylbenzene (**1**) (10.6 mg, 0.100 mmol, 1.0 equiv) was added, the flask was tightly sealed, placed in a pre-heated aluminum block, and stirred with 1000 rpm at the indicated temperature for the indicated time. After cooling to room-temperature mesitylene (a weighed amount of approx. 0.1 mmol in 0.5 mL EtOAc) and EtOAc (3 mL) were added and the mixture was rapidly stirred at room temperature for 10 min. 500 µL of this mixture were filtered through a small plug of silica and eluted with EtOAc (1.5 mL). Conversion of **1**, regioselectivities of **2** and overall yield of **2** were determined by GC-FID using mesitylene as internal standard.

Table S 1: Initial examination of reaction parameters:

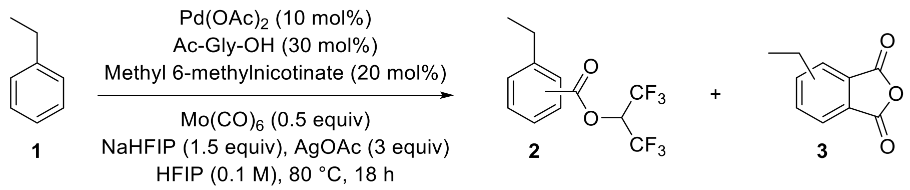

Entry	Deviation	**1** Conversion [%]	**2** o	m	p	∑ Yield **2** [%]	Chemo-selectivity*
1	No NaHFIP	**36**	5	62	32	**6**	0.14
2	-	**25**	1	68	31	**11**	0.42
3	$Mo(CO)_6$ (1.0 equiv)	**19**	2	67	31	**9**	0.49
4	HFIP (1.5 mL)	**23**	1	69	30	**9**	0.38

In the beginning a significant challenge was to prevent the formation of by-product **3**. Without NaHFIP (Entry 1) **3** was the main product while with the addition of NaHFIP the amount of **3** significantly decreased (Entry 2). The addition of more $Mo(CO)_6$ and the dilution of the reaction mixture did not impact the reaction outcome.

* $Chemoselectivity = \frac{\sum \text{Yield } \mathbf{2}\ [\%]}{\text{Conversion } (\mathbf{1})\ [\%]}$; 0.0 = Lowest possible chemoselectivity → 1.0 = Highest possible chemoselectivity.

Table S 2: Initial temperature and time screening.

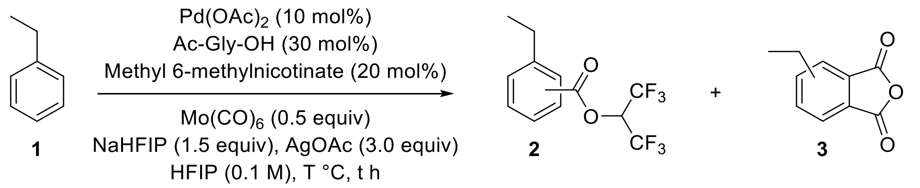

Entry	T [°C] / t [h]	1<br>Conversion [%]	2<br>o m p	 ∑ Yield **2** [%]	Chemo-<br>selectivity
1	70 / 18	**38**	2 67 31	**14**	0.36
2	70 / 48	**38**	2 67 32	**15**	0.39
3	80 / 18	**28**	2 68 30	**10**	0.36
4	80 / 48	**27**	1 68 30	**8**	0.31
5	90 / 18	**27**	2 67 31	**9**	0.35
6	90 / 48	**27**	2 68 31	**9**	0.32

Table S 3: Screening of Ag-loading and CO-sources.

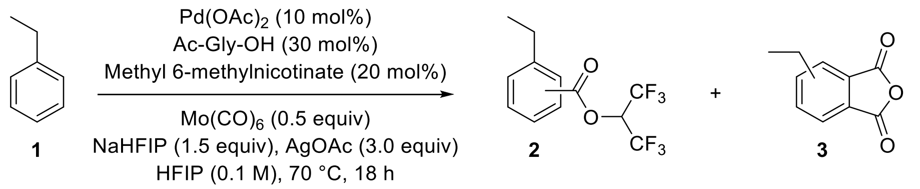

Entry	Deviation	1<br>Conversion [%]	2<br>o m p	 ∑ Yield **2** [%]	Chemo-<br>selectivity
1	-	**40**	1 67 32	**16**	0.41
2	AgOAc (4 equiv)	**41**	2 66 32	**17**	0.42
3	$Ru_3(CO)_{12}$ (0.25 equiv)	**9**	4 56 40	**2**	0.22
4	$W(CO)_6$ (0.5 equiv)	**23**	8 64 28	**3**	0.13
5	$Mn_2(CO)_{10}$ (0.3 equiv)	**15**	5 56 39	**4**	0.27

Table S 4: Further temperature screening.

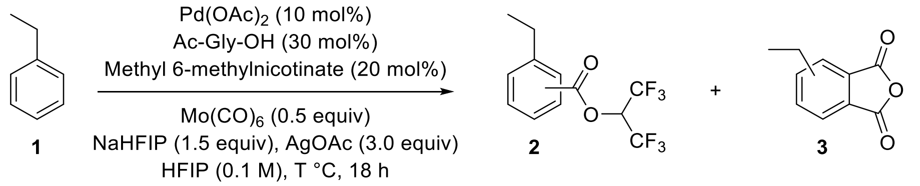

Entry	T [°C]	1 Conversion [%]	2 o	m	p	∑ Yield 2 [%]	Chemo-selectivity
1	40	**8**	5	64	31	**5**	0.64
2	50	**14**	1	68	30	**9**	0.64
3	60	**24**	2	67	31	**14**	0.56
4	70	**38**	2	66	32	**17**	0.44

Table S 5: Screening of Ag-salts.

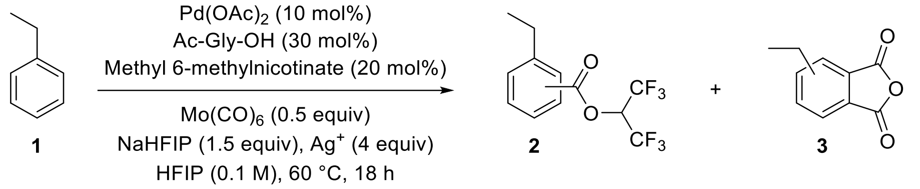

Entry	Ag-salt (4 equiv Ag)	1 Conversion [%]	2 o	m	p	∑ Yield 2 [%]	Chemo-selectivity
1	AgOAc	**25**	1	67	31	**15**	0.58
2	Ag_2CO_3	**6**	3	70	27	**6**	0.97
3	Ag_2O	**7**	3	67	30	**6**	0.75
4	Ag_3PO_4	**5**	1	72	27	**5**	0.97
5	AgF	**12**	3	60	36	**9**	0.70
6	AgTFA	**24**	9	40	51	**17**	0.73
7	$AgNO_2$	**5**	17	58	25	**1**	0.26
8	$AgNO_3$	**25**	2	67	31	**17**	0.67

Note: By exchanging AgOAc with other silver salts the formation of **3** was almost completely suppressed. We reasoned that the addition of acetate ions is necessary for the formation of **3** to occur. Trace amounts of **3** can still form due to the presence of acetate from $Pd(OAc)_2$.For a more detailed discussion, see chapter 7.

Table S 6: Screening of NaHFIP-loading.

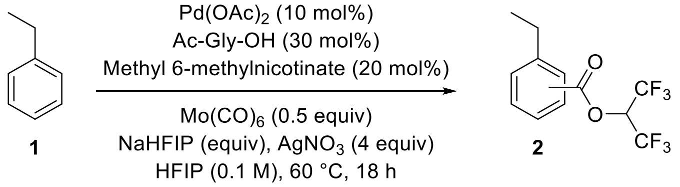

Entry	NaHFIP [equiv]	**1** Conversion [%]	**2** o m p	**2** ∑ Yield **2** [%]	Chemo-selectivity
1	0	**19**	4 68 28	**8**	0.44
2	0.5	**23**	2 68 29	**13**	0.56
3	1.0	**26**	2 68 30	**14**	0.55
4	1.5	**26**	2 67 31	**16**	0.60
5	2.0	**29**	2 66 33	**18**	0.63
6	2.5	**26**	1 66 33	**16**	0.59
7	3.0	**22**	1 65 34	**13**	0.57

Table S 7: Further temperature screening.

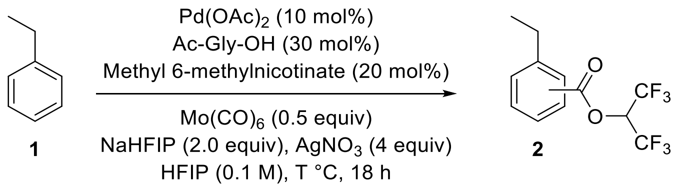

Entry	T [°C]	**1** Conversion [%]	**2** o	m	p	∑ Yield **2** [%]	Chemo-selectivity
1	60	**26**	1	66	33	**17**	0.63
2	70	**33**	2	68	30	**23**	0.70
3	80 (Ref. 1. Experiment)	**39**	2	69	29	**25**	0.64
4	80 (Ref. 2. Experiment)	**45**	2	69	29	**27**	0.59
5	90	**44**	2	70	28	**24**	0.55
6	100	**32**	1	72	27	**14**	0.45
7	110	**31**	2	71	27	**8**	0.24

Table S 8: Initial Base-screening.

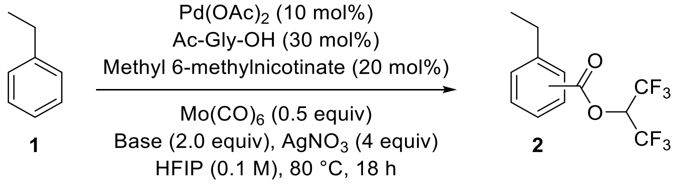

Entry	Base [2 equiv]	**1** Conversion [%]	**2** o	m	p	∑ Yield **2** [%]	Chemo-selectivity
1	NaHFIP	**41**	2	69	29	**24**	0.57
2	LiHFIP	**30**	3	69	29	**16**	0.55
3	KHFIP	**35**	2	64	34	**15**	0.43
4	Cs_2CO_3	**14**	3	66	32	**7**	0.51
5	NaO^{t}Bu	**19**	2	70	28	**10**	0.52

Table S 9: Further base-screening.

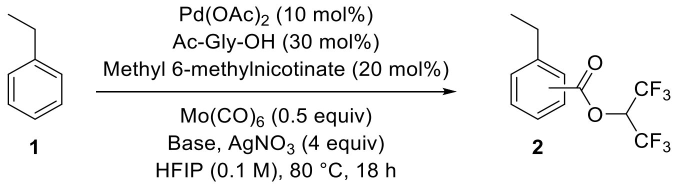

Entry	Base [equiv]	**1**	**2**				Chemo-selectivity
		Conversion [%]	o	m	p	∑ Yield **2** [%]	
1	NaHFIP (2)	**44**	2	69	29	**25**	0.56
2	$NaHCO_3$ (2)	**43**	2	69	29	**17**	0.41
3	Na_2CO_3 (1)	**42**	2	70	28	**19**	0.45
4	Na_2CO_3 (2)	**32**	2	71	28	**17**	0.53
5	NaOAc (2)	**54**	2	68	29	**12**	0.22
6	NaTFA (2)	**15**	12	45	43	**3**	0.20
7	Na_2HPO_4 (2)	**43**	2	69	29	**17**	0.39

Table S 10: Further base-screening.

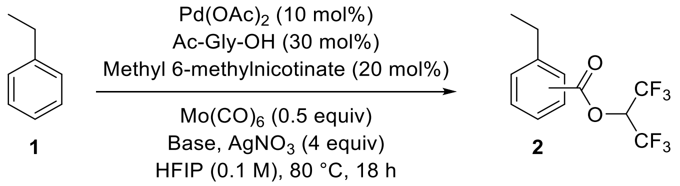

Entry	Base [equiv]	**1** Conversion [%]	**2** o m p	∑ Yield **2** [%]	Chemo-selectivity
1	NaHFIP (2)	**35**	2 69 29	**27**	0.76
2	DIPEA (2)	**21**	2 66 32	**13**	0.62
3	DBU (2)	**29**	2 67 30	**24**	0.83
4	$CaCO_3$ (2)	**21**	2 71 27	**10**	0.47
5	Na_2HPO_4 (1)	**36**	3 69 29	**19**	0.52
6	$KHCO_3$ (2)	**27**	2 70 28	**20**	0.72
7	$(NaPO_3)_3$ (0.7)	**38**	2 69 29	**22**	0.56
8	Li_2CO_3 (1)	**36**	2 69 29	**20**	0.57

Table S 11: Initial bidentate-ligand-screening.

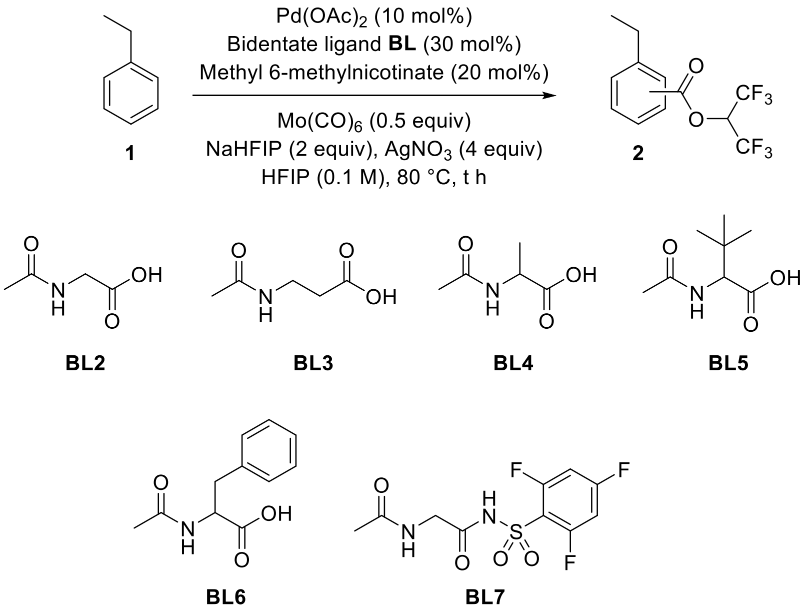

Entry	Ligand/ time [h]	**1** Conversion [%]	**2** o	m	p	∑ Yield **2** [%]	Chemo-selectivity
1	**BL2**/ 18	**45**	2	69	29	**26**	0.56
2	**BL2**/ 38	**48**	2	69	29	**27**	0.56
3	No **BL**/ 38	**12**	16	39	45	**2**	0.18
4	**BL3**/ 38	**40**	2	62	36	**14**	0.36
5	**BL4**/ 38	**60**	2	66	33	**30**	0.50
6	**BL5**/ 38	**21**	6	56	38	**9**	0.43
7	**BL6**/ 38	**40**	0	67	33	**23**	0.57
8	**BL7**/ 38	**30**	27	37	36	**17**	0.56

To ensure maximum conversion is reached, reactions were performed for 38 h during ligand screening.

Table S 12: Further bidentate-ligand-screening.

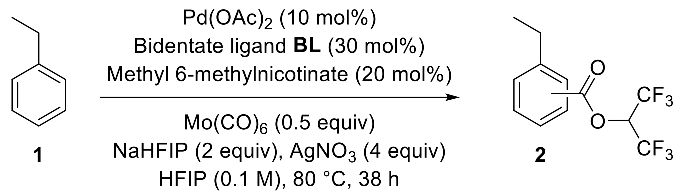

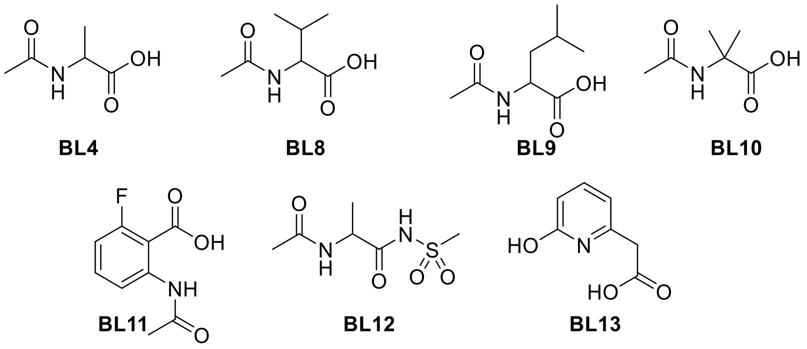

Entry	Ligand	**1** Conversion [%]	**2** o	m	p	∑ Yield **2** [%]	Chemo-selectivity
1	**BL4**	**57**	2	66	33	**28**	0.49
2	**BL8**	**52**	1	65	34	**26**	0.50
3	**BL9**	**60**	2	64	34	**32**	0.53
4	**BL10**	**24**	5	58	37	**11**	0.47
5	**BL11**	**37**	2	57	41	**19**	0.53
6	**BL12**	**69**	23	33	44	**48**	0.69
7	**BL13**	**12**	2	62	35	**4**	0.31

Table S 13: Further bidentate-ligand-screening.

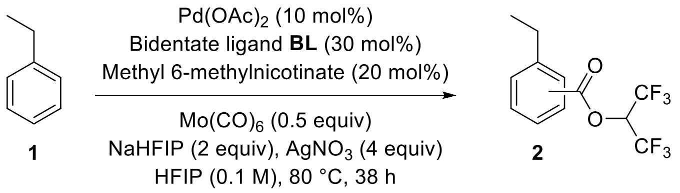

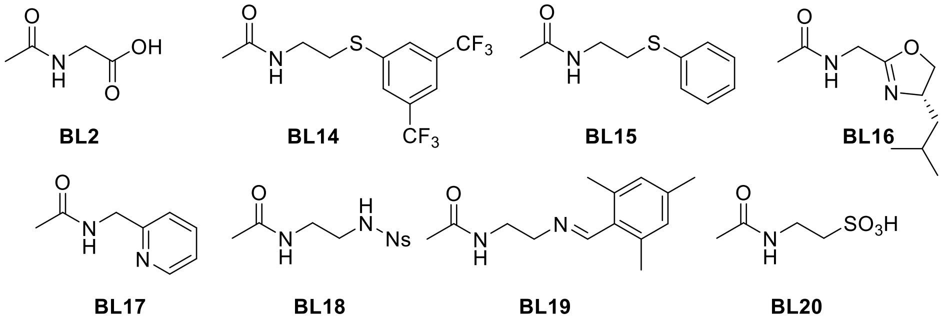

Entry	Ligand	**1** Conversion [%]	**2** o	m	p	∑ Yield **2** [%]	Chemo-selectivity
1	**BL2**	**51**	2	69	29	**31**	0.61
2	**BL14**	**48**	2	62	35	**35**	0.73
3	**BL15**	**46**	5	58	37	**32**	0.71
4	**BL16**	**18**	5	63	32	**9**	0.50
5	**BL17**	**12**	6	64	31	**2**	0.19
6	**BL18**	**15**	19	45	36	**5**	0.31
7	**BL19**	**9**	12	54	34	**1**	0.12
8	**BL20**	**23**	5	53	42	**10**	0.42

Table S 14: Further bidentate-ligand-screening.

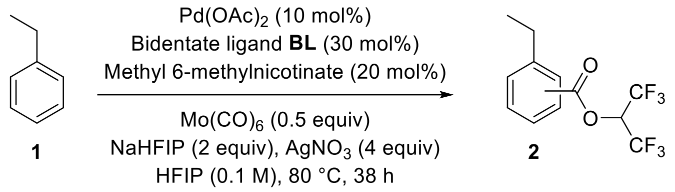

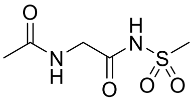
BL21

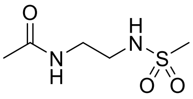
BL22

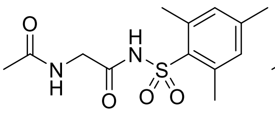
BL23

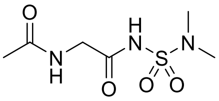
BL24

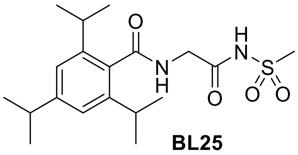
BL25

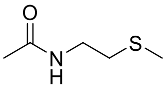
BL26

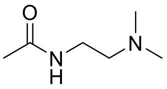
BL27

Entry	Ligand	**1**	**2**				Chemo-selectivity
		Conversion [%]	o	m	p	∑ Yield **2** [%]	
1	**BL21**	**30**	25	37	38	**20**	0.67
2	**BL22**	**15**	14	51	35	**6**	0.38
3	**BL23**	**41**	29	32	39	**27**	0.67
4	**BL24**	**20**	25	37	38	**13**	0.66
5	**BL25**	**71**	9	48	42	**52**	0.73
6	**BL26**	**35**	5	59	35	**26**	0.73
7	**BL27**	**11**	16	40	44	**3**	0.28

Table S 15: Evaluation of reaction times.

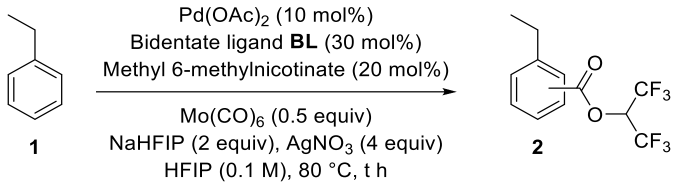

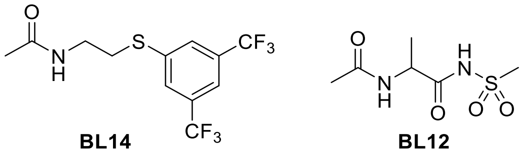

Entry	Ligand / Time [h]	**1** Conversion [%]	**2** o	m	p	∑ Yield **2** [%]	Chemo-selectivity
1	**B14** / 18	**39**	2	63	35	**32**	0.80
2	**B14** / 38	**39**	2	63	35	**29**	0.75
3	**B12** / 18	**41**	20	36	44	**27**	0.65
4	**B12** / 38 (Ref. 1. Experiment)	**57**	20	36	44	**37**	0.65
5	**B12** / 38(Ref. 2. Experiment)	**49**	18	37	45	**33**	0.67
6	**B12** / 72	**58**	19	36	45	**38**	0.66

Note: Due to a contamination in our HFIP the yields are slightly lower in this table. Nevertheless, a reaction time of 38 h for thioether ligands (to ensure maximum conversion until the end of the screening) and a reaction time of 72 h for *N*-acylsulfonamide-ligands was used for the rest of the screening.

Table S 16: Comparison of different *N*-acylsulfonamide-ligands.

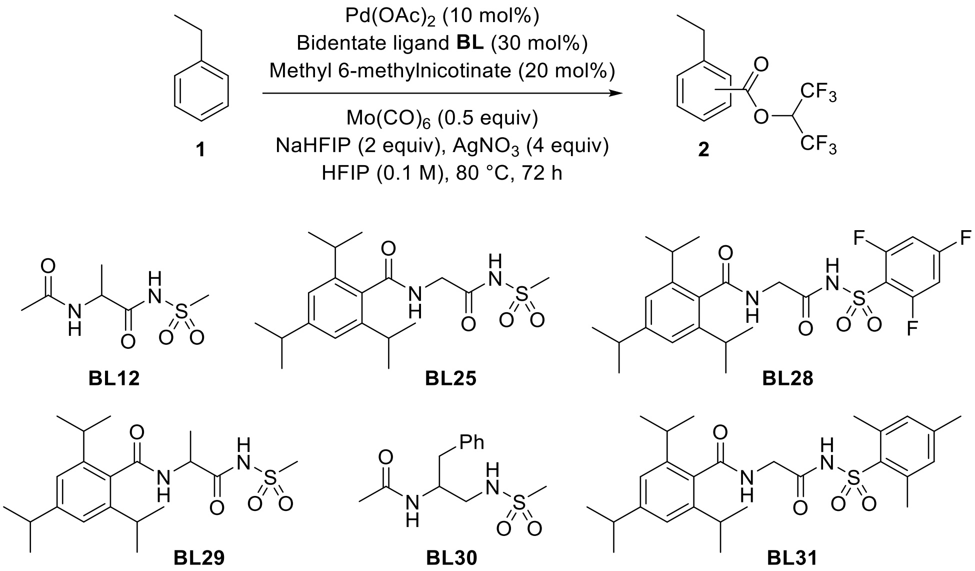

Entry	Ligand	**1** Conversion [%]	**2** o	m	p	∑ Yield **2** [%]	Chemo-selectivity
1	**BL12**	**65**	20	35	45	**45**	0.69
2	**BL25**	**65**	7	51	42	**45**	0.69
3	**BL28**	**41**	8	48	44	**29**	0.71
4	**BL29**	**54**	8	58	34	**37**	0.69
5	**BL30**	**53**	14	43	43	**36**	0.68
6	**BL31**	**39**	6	58	36	**25**	0.64

Table S 17: Comparison between different thioether-ligands.

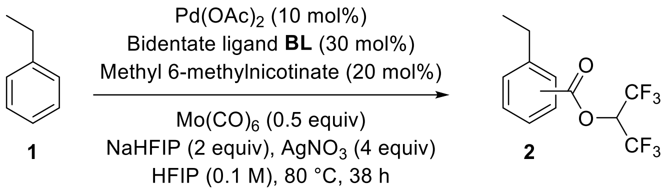

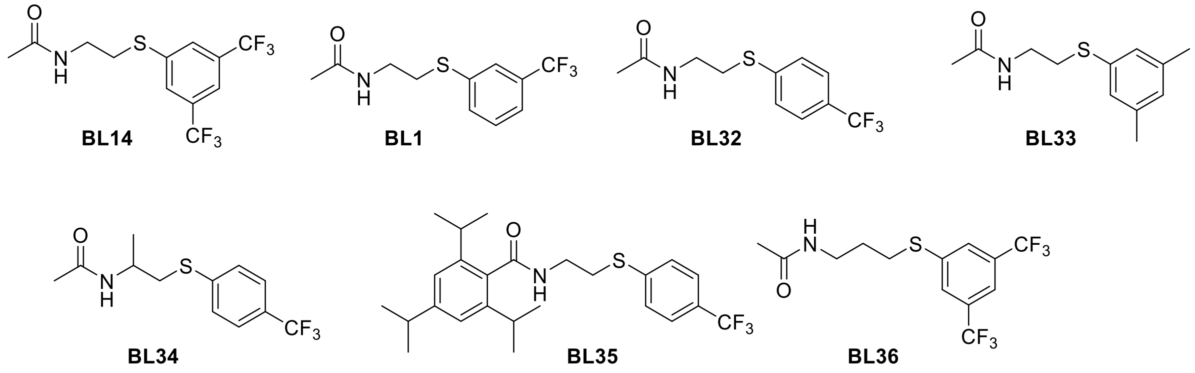

Entry	Ligand	**1** Conversion [%]	**2** o	m	p	∑ Yield **2** [%]	Chemo-selectivity
1	**BL14**	**44**	2	62	36	**34**	0.78
2	**BL14** / No Methyl-6-methylnicotinate	**25**	6	53	41	**18**	0.73
3	**BL1**	**57**	3	64	33	**47**	0.83
4	**BL32**	**49**	3	64	33	**40**	0.81
5	**BL33**	**46**	8	54	38	**37**	0.80
6	**BL34**	**41**	4	63	34	**35**	0.86
7	**BL35**	**24**	3	60	36	**20**	0.81
8	**BL36**	**11**	5	54	41	**6**	0.57

Table S 18: Screening of monodentate ligands **ML** together with **BL25**.

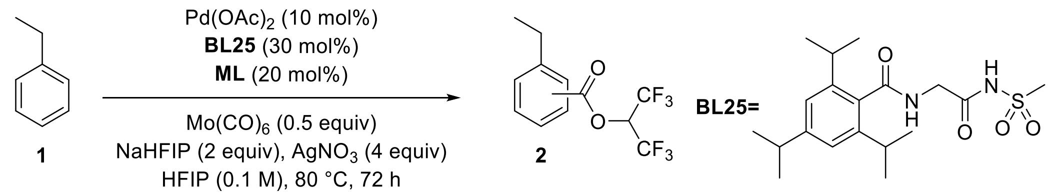

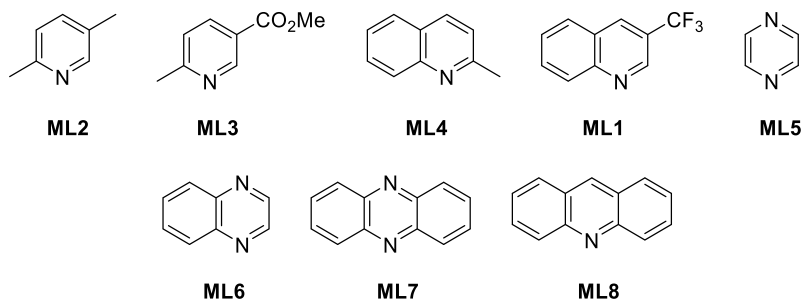

Entry	Ligand	**1** Conversion [%]	**2** o	m	p	∑ Yield **2** [%]	Chemo-selectivity
1	**ML2**	**62**	10	47	43	**41**	0.66
2	**ML3**	**68**	8	50	43	**48**	0.70
3	**ML4**	**79**	8	50	42	**55**	0.70
4	**ML1**	**75**	8	49	43	**53**	0.71
5	**ML5**	**80**	8	49	43	**59**	0.74
6	**ML6**	**79**	8	48	43	**60**	0.77
7	**ML7**	**84**	8	49	43	**65**	0.77
8	**ML8**	**77**	8	50	42	**54**	0.70

Table S 19: Further screening of monodentate ligands **ML** together with **BL25**.

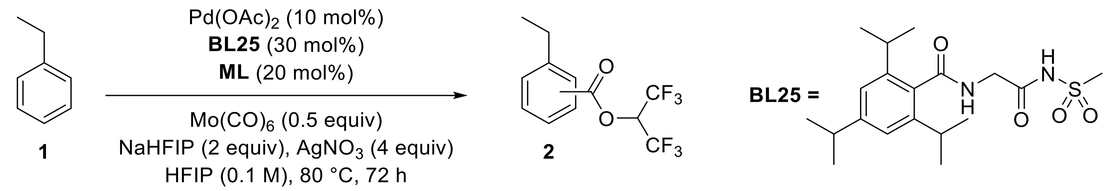

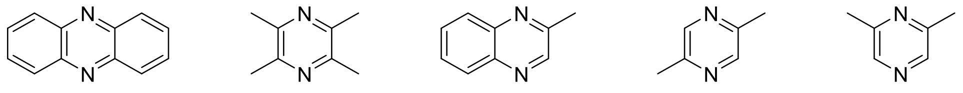

ML7 **ML9** **ML10** **ML11** **ML12**

Entry	Ligand	1<br>Conversion [%]	2<br>o m p	 ∑ Yield **2** [%]	Chemo-selectivity
1	**ML7**	**85**	9 48 43	**68**	0.81
2	**ML9**	**83**	8 50 42	**65**	0.78
3	**ML10**	**80**	8 49 43	**64**	0.79
4	**ML11**	**79**	8 49 43	**61**	0.78
5	**ML12**	**76**	9 49 42	**59**	0.78

Table S 20: Screening of monodentate ligands **ML** together with **BL1**.

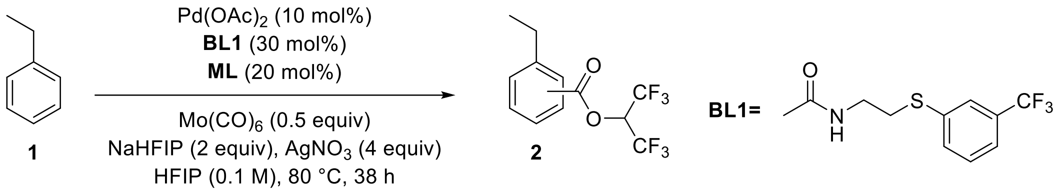

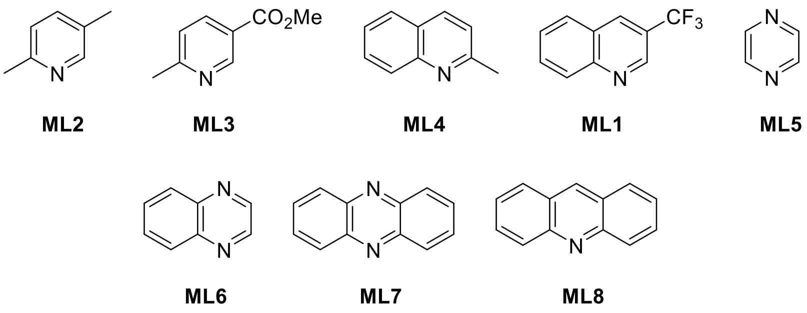

Entry	Ligand	1 Conversion [%]	2 o	m	p	∑ Yield **2** [%]	Chemo-selectivity
1	**ML2**	**45**	3	67	31	**35**	0.77
2	**ML3**	**61**	3	64	33	**50**	0.82
3	**ML4**	**57**	3	64	33	**45**	0.78
4	**ML1**	**82**	2	67	30	**68**	0.83
5	**ML5**	**61**	6	54	39	**48**	0.79
6	**ML6**	**82**	3	60	36	**70**	0.85
7	**ML7**	**57**	3	62	35	**46**	0.82
8	**ML8**	**76**	1	67	32	**59**	0.79

ML1 and **ML6** lead to an almost identical reaction outcome. However, the distribution of regioisomers suggest a slightly more sterically controlled reaction when using **ML1**, such that this ligand was used in further investigations.

Table S 21: Further screening of monodentate-ligands **ML** together with **B28**.

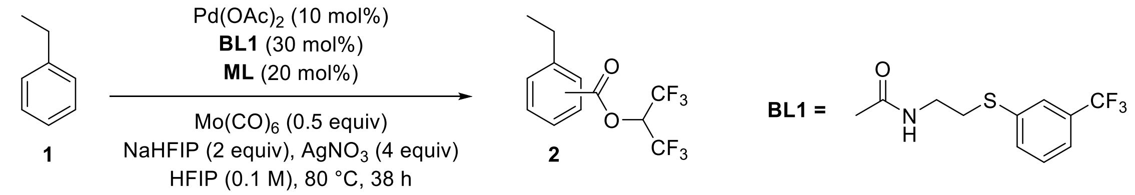

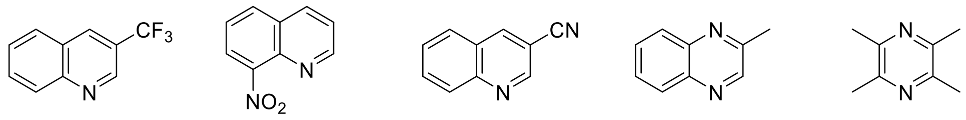

ML1 **ML13** **ML14** **ML10** **ML9**

Entry	Ligand	1	2				Chemo-selectivity
		Conversion [%]	o	m	p	∑ Yield **2** [%]	
1	**ML1**	**84**	2	67	30	**70**	0.84
2	**ML13**	**18**	8	48	44	**14**	0.75
3	**ML14**	**64**	4	60	36	**55**	0.86
4	**ML10**	**67**	3	60	36	**59**	0.88
5	**ML9**	**41**	6	52	41	**37**	0.89

BL1 together with **ML1** proved to be the optimal combination of ligands that give the highest conversion, the highest yield and the highest Conversion/ Yield-ratio. Moreover, steric factors are more pronounced in the distribution of regioisomers when using a combination of **BL15** and **ML7**. **BL1** and **ML1** were therefore used in the rest of the optimization campaign.

Table S 22: Ligand-ratio screening.

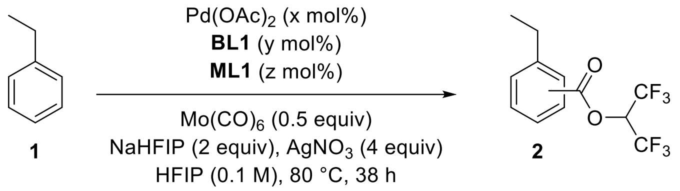

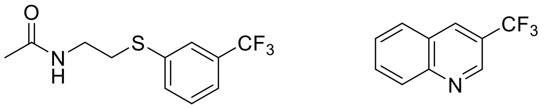

Entry	x:y:z	**1** Conversion [%]	**2** o	m	p	∑ Yield **2** [%]	Chemo-selectivity
1	10:10:10	**59**	3	68	29	**42**	0.72
2	10:20:10	**74**	3	66	31	**57**	0.78
3	10:30:10	**74**	3	65	32	**59**	0.80
4	10:40:10	**80**	3	65	32	**65**	0.82
5	10:10:20	**64**	2	70	28	**48**	0.75
6	10:20:20	**78**	2	68	30	**61**	0.79
7	10:30:20	**83**	2	67	30	**67**	0.81
8	10:40:20	**88**	2	66	31	**72**	0.81

Table S 23: Screening of Ag-loading.

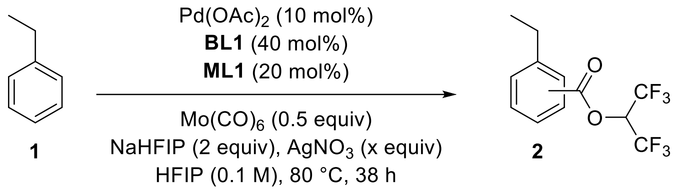

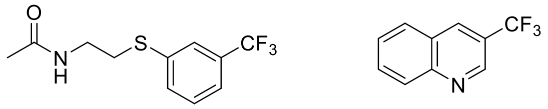

BL1 **ML1**

Entry	x	**1** Conversion [%]	**2** o	m	p	∑ Yield **2** [%]	Chemo-selectivity
1	**2.0**	**17**	4	65	31	**13**	0,78
2	**2.5**	**23**	5	64	32	**19**	0,81
3	**3.0**	**39**	4	66	30	**32**	0,81
4	**3.5**	**79**	2	67	31	**65**	0,83
5	**4.0**	**84**	2	66	31	**69**	0,82
6	**4.5**	**74**	3	67	31	**60**	0,81

Table S 24: Screening of $Mo(CO)_6$-loading.

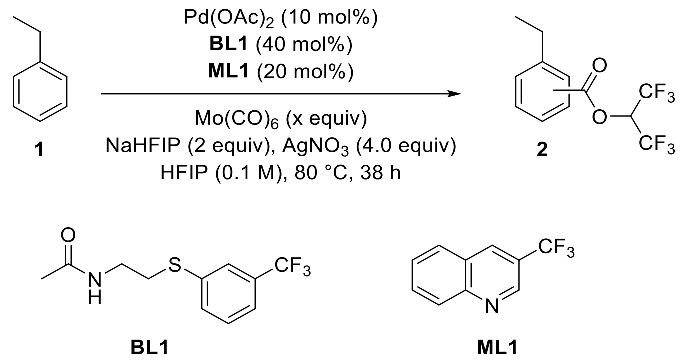

Entry	x	**1**<br>Conversion [%]	**2**<br>o m p	 ∑ Yield **2** [%]	Chemo-selectivity
1	**0.17**	**69**	1 66 33	**55**	0.79
2	**0.33**	**89**	2 66 32	**75**	0.84
3	**0.5**	**85**	2 66 31	**69**	0.81
4	**0.75**	**69**	3 66 31	**57**	0.83
5	**1.0**	**59**	3 66 31	**49**	0.84
6	**1.25**	**50**	3 66 31	**41**	0.82

Table S 25: Screening of Pd-salt and reaction time.

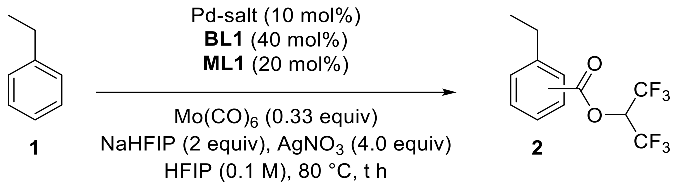

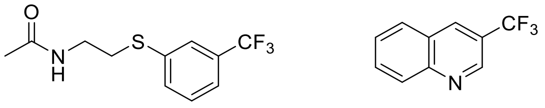

BL1 **ML1**

Entry	Pd-salt / Time [h]	**1** Conversion [%]	**2** o	m	p	∑ Yield **2** [%]	Chemo-selectivity
1	$Pd(OAc)_2$ / 18	**87**	2	66	32	**70**	0.80
2	$Pd(OAc)_2$ / 38	**94**	2	66	33	**75**	0.81
3	$Pd(TFA)_2$ / 18	**90**	2	66	33	**76**	0.85
4	$Pd(TFA)_2$ / 38	**93**	2	65	33	**79**	0.85
5	$Pd(MeCN)_2Cl_2$ / 18	**89**	2	66	32	**77**	0.87
6	$Pd(MeCN)_2Cl_2$ / 38	**92**	2	65	33	**81**	0.88
7	$Pd(NO_3)_2 \cdot 2\ H_2O$ / 18	**83**	2	66	31	**74**	0.88
8	$Pd(NO_3)_2 \cdot 2\ H_2O$ / 38	**90**	2	66	32	**77**	0.86

Table S 26: Further ligand ratio, time and ligand screening.

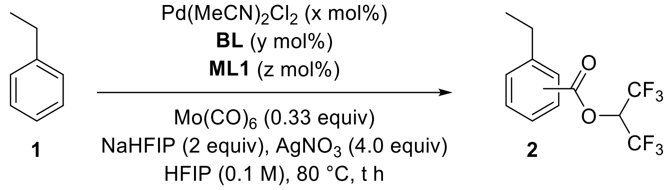

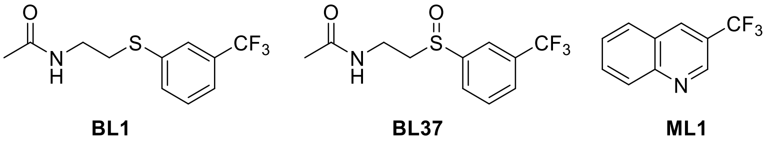

Entry	**BL**; x:y:z; t [h]	**1** Conversion [%]	**2** o	m	p	∑ Yield **2** [%]	Chemo-selectivity
1	**BL1**; 10:40:20; 38	**84**	2	66	32	**77**	0.92
2	**BL1**; 10:40:20; 24	**88**	2	66	32	**77**	0.88
3	**BL1**; 10:50:20; 24	**85**	2	65	32	**75**	0.89
4	**BL1**; 10:60:20; 24	**83**	2	65	33	**74**	0.89
5	**BL1**; 5:20:10; 24	**46**	2	66	32	**40**	0.88
6	**BL37**; 10:40:20; 24	**45**	2	70	29	**37**	0.82

Table S 27: Control reactions.

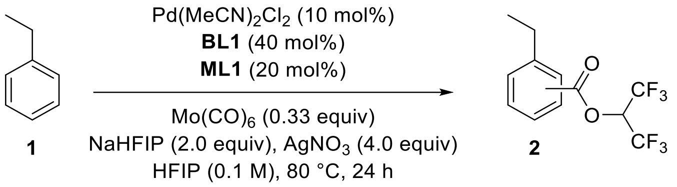

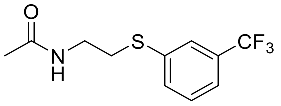

BL1

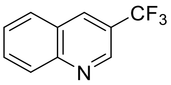

ML1

Entry	Deviation	**1** Conversion [%]	**2** o	m	p	∑ Yield **2** [%]	Chemo-selectivity
1	-	**95**	2	65	33	**83**	0.88
2	No $Pd(MeCN)_2Cl_2$	**0**	-	-	-	**n.d.**	n.d.
3	No **BL1**	**4**	0	60	40	**Trace**	0.08
4	No **ML1**	**61**	11	46	43	**54**	0.88
5	No **BL1** + No **ML1**	**3**	0	51	49	**Trace**	0.03
6	No $Pd(MeCN)_2Cl_2$+**BL1**+**ML1**	**1**	-	-	-	**n.d.**	n.d.
7	No $AgNO_3$	**3**	5	63	32	**1**	0.35
8	No NaHFIP	**96**	2	65	33	**82**	0.85

2.2 Re-Optimization for Arenes Containing an Electron-Withdrawing Group

Table S 28: Initial screening for better reaction conditions for **1'**.

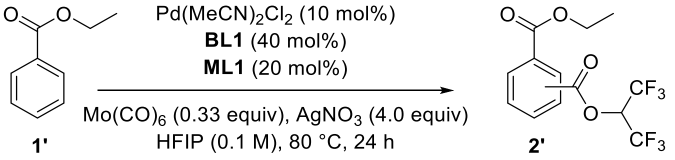

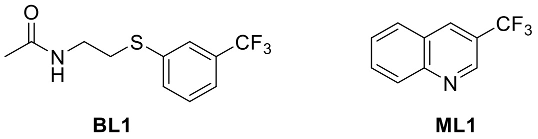

Entry	Deviation	2			
		o	m	p	∑ Yield **2** [%]
1	85 °C	5	72	23	**83**
2	$Pd(MeCN)_2Cl_2$ (15 mol%), **BL1** (60 mol%), **ML1** (30 mol%)	6	72	22	**78**
3	NaHFIP (2 equiv)	11	70	19	**74**
4	48 h	7	70	23	**62**
5	-	5	72	22	**75**
6	HFIP (0.15M)	7	70	23	**84**

Note: The yields in this table were obtained using the same screening procedure with ethyl benzoate (**1'**, 15.0 mg, 0.100 mmol, 1.0 equiv) instead of ethylbenzene (**1**) as starting material. However, no authentic sample of **2'** could be obtained, such that the calibration factor was estimated using a crude reaction mixture calibrated to the ^{1}H-NMR yield with mesitylene as internal standard. This may lead to a significant error in the value of the calibration factor, such that the determined GC-yields are most likely too high. However, the qualitative factors to increase the yield for this electron poor arene could still be determined and were verified during scope studies.

Table S 29: Temperature screening with **1'**.

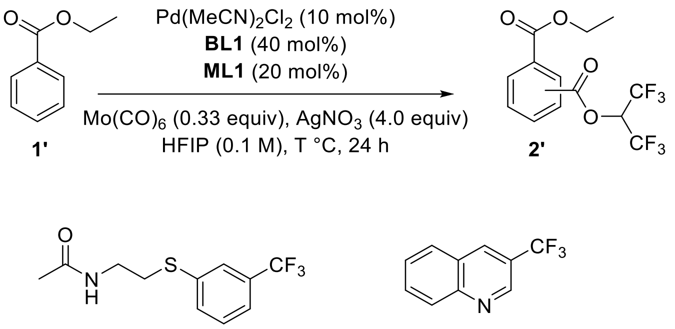

BL1 **ML1**

Entry	**T [°C]**	**2**			
		o	m	p	∑ Yield **2** [%]
1	80	8	71	22	**79**
2	85	3	75	22	**87**
3	90	3	74	23	**78**
4	100	3	72	25	**61**

Table S 30: Concentration screening with **1’**.

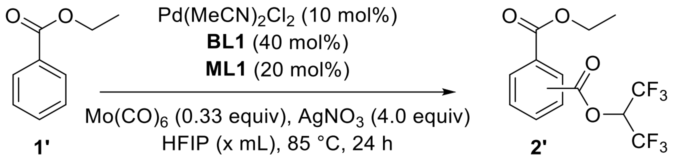

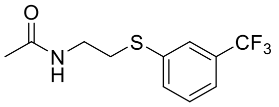

BL1

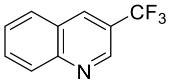

ML1

Entry	**HFIP (mL)**	**2**			
		o	m	p	∑ Yield **2** [%]
1	0.4	4	74	23	**80**
2	0.5	3	74	23	**91**
3	0.6	3	73	23	**91**
4	0.7	1	74	42	**82**
5	0.8	3	73	23	**86**

Note: During scope studies, the reaction temperature was adjusted to 85 °C and reactions were performed at a concentration of 0.17M for electron-poor arenes.

2.3 Screening of Reduced Ag-Amount for Scale-up Reactions and alternative solvents/ ligands

Table S 31: Screening with reduced $AgNO_3$ loading.

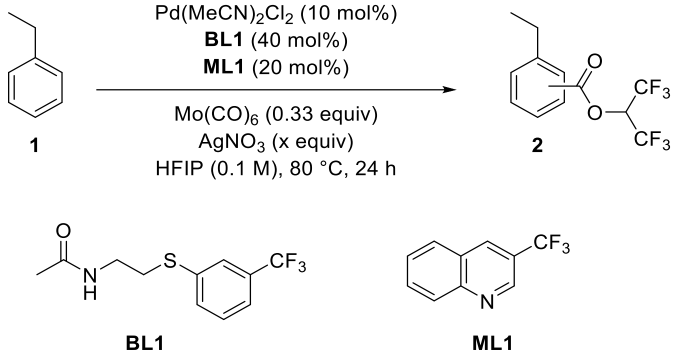

Entry	$AgNO_3$ (equiv)	**1** Conversion [%]	**2** o m p	∑ Yield **2** [%]	Chemo-selectivity
1	4.0	**98**	2 64 34	**83**	0.83
2	2.5	**97**	2 64 34	**80**	0.82
3	2.0	**97**	2 64 34	**82**	0.84
4	1.5	**96**	2 65 33	**80**	0.84
5	1.0	**82**	2 65 33	**71**	0.87
6	0.5	**7**	5 60 35	**7**	0.98
7	0.0	**7**	2 91 7	**1**	0.17

Note: This screening table was produced after finishing the scope studies in order to lower the required amounts of $AgNO_3$ for scale-up reactions. It showed that under optimized conditions even 1.5 equiv of $AgNO_3$ are enough to achieve virtually the same results than with 4.0 equiv of $AgNO_3$ used for the scope studies. This is in sharp contrast to the initial results contained in Table S23, where a significant decrease in yield was observed when employing less than 3.5 equiv of $AgNO_3$. In order to explore the nature of this sharp decrease in yield the following reactions were performed.

Table S 32: Screening of reaction parameters with $AgNO_3$ (3.0 equiv).

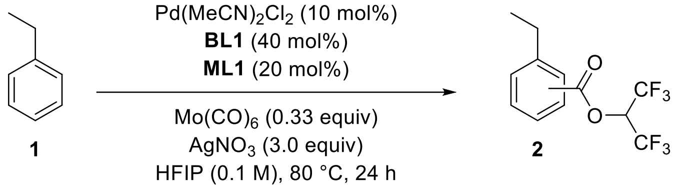

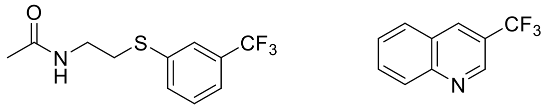

BL1 **ML1**

Entry	Deviation	**1** Conversion [%]	**2** o	m	p	∑ Yield **2** [%]	Chemo-selectivity
1	$AgNO_3$ (4 equiv)	**95**	3	65	33	**81**	0.86
2	-	**98**	2	64	34	**82**	0.84
3	NaHFIP (2.0 equiv)	**90**	2	66	32	**82**	0.82
4	NaHFIP (2.0 equiv) and $Pd(OAc)_2$	**89**	2	67	32	**80**	0.89
5	NaHFIP (2.0 equiv), $Pd(OAc)_2$ and $Mo(CO)_6$ (0.5 equiv)	**38**	4	66	30	**35**	0.92
6	NaHFIP (2.0 equiv), $Pd(OAc)_2$,$Mo(CO)_6$ (0.5 equiv) and 38 h	**44**	4	66	30	**40**	0.92
7	38 h	**98**	2	34	34	**82**	0.84

Note: Entry 4 and 5 show that increasing the equivalents of $Mo(CO)_6$ from 0.33 to 0.5 (which was used in Table S23) has a detrimental effect on the yield. This detrimental effect can be compensated by the addition of more $AgNO_3$ as shown in Table S23. Lowering the equivalents of $Mo(CO)_6$ therefore also led to the requirement of less $AgNO_3$ in the optimized conditions, which is highly beneficial for large scale reactions.

Additionally, we tested the reaction in alternative solvents and using other commercially available amino acid derived ligands (Table S 33). The reaction also proceeded in 2,2,2-trifluoroethanol with moderate yield, resulting in the formation of the respective 2,2,2-trifluoroethyl esters (Entry 2). Trace amounts of the respective isopropyl esters were also detected when the reaction was performed in isopropanol (Entry 4). Other solvents did not lead to any product formation (Entry 3+5+6). Commercially available **BL6** and **BL9** only resulted in moderate product formation (Entry 7+8).

Table S 33: Screening of additional reaction parameters

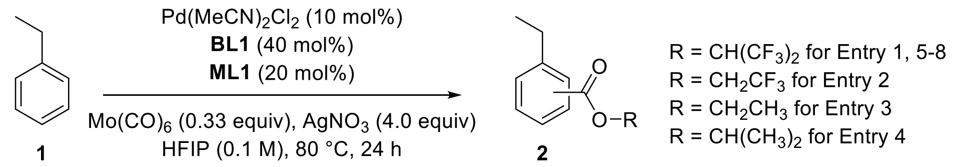

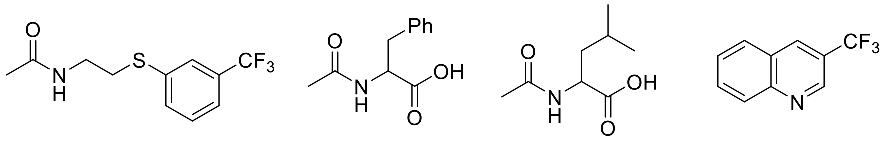

BL1 **BL6** **BL9** **ML1**

Entry	Deviation	**1** Conversion [%]	**2** o	m	p	∑ Yield **2** [%]	Conversion / Yield (**2**)
1	-	**97**	2	64	34	**91**	0.94
2	**2,2,2-Trifluoroethanol instead of HFIP**	**47**	1	64	35	**39[a]**	0.83
3	**Ethanol instead of HFIP**	**0**	-	-	-	**n.d.**	
4	**Isopropanol instead of HFIP**	**Trace**	-	-	-	**Trace[b]**	
5	**MeCN + HFIP (2 equiv) instead of HFIP**	**0**	-	-	-	**n.d.**	
6	**DCE + HFIP (2 equiv) instead of HFIP**	**2**	-	-	-	**n.d.**	
7	**BL6 instead of BL1**	**44**	0	70	30	**26**	0.58
8	**BL9 instead of BL1**	**48**	2	71	27	**23**	0.47

[a]Yield of **2** refers to the respective 2,2,2-trifluoroethyl esters instead of 1,1,1,3,3,3-hexafluoroisopropyl esters. Additionally, the reaction was performed on 0.2 mmol scale and the product was isolated as carboxylic acid (see chapter 4.2). [b] Trace amounts of the respective isopropyl esters were detected.

3. Synthesis of Starting Materials and Ligands

3.1 Synthesis of Authentic Samples

General procedure for the preparation of authentic samples: The corresponding benzoic acid-derivative was added to a Schlenk-tube, placed under N_2 and dissolved in dry CH_2Cl_2 (0.3 M). $SOCl_2$ (1.2 equiv) and *N,N*-dimethylformamide (1-2 drops) were added, the flask was tightly sealed and placed in a pre-heated aluminum block. where the reaction mixture was stirred at 50 °C for 4 h. All volatiles were evaporated under reduced pressure to afford the corresponding benzoyl chloride which was used without further purification.

Dry NEt_3 (2.5 equiv), HFIP (8.0 equiv), and dry CH_2Cl_2 (0.3 M) were added to a Schlenk-tube under N_2 atmosphere. The solution was cooled to 0 °C using an ice-bath and the crude benzoyl chloride (dissolved in a minimal amount of CH_2Cl_2) was added carefully via syringe. The ice-bath was removed and the solution was stirred at room temperature overnight. The reaction mixture was directly adsorbed on silica using a rotary evaporator and the target compound was isolated by automated flash column chromatography (pentane).

1,1,1,3,3,3-hexafluoropropan-2-yl 2-ethylbenzoate

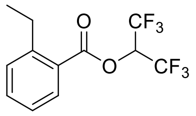

Prepared according to the general procedure using 2-ethylbenzoic acid (1.50 g, 10.0 mmol). The title compound (2.17g, 7.23 mmol, 72%) was obtained as colorless liquid.

^{1}H NMR (500 MHz, CDCl$_3$) δ = 7.99-7.97 (m, 1H), 7.57-7.53 (m, 1H), 7.38 – 7.29 (m, 2H), 6.02 (hept, *J* = 6.2 Hz, 1H), 3.00 (q, *J* = 7.5 Hz, 2H), 1.24 (t, *J* = 7.5 Hz, 3H) ppm.
^{13}C NMR (126 MHz, CDCl$_3$) δ 163.7, 148.0, 134.1, 131.5, 130.8, 126.3, 125.7, 120.8 (q, *J* = 284.6 Hz), 66.7 (hept., *J* = 34.0 Hz), 27.8, 15.8 ppm.
^{19}F NMR (471 MHz, CDCl$_3$) δ = −73.1 ppm.
HRMS (ESI-pos): Calcd. for $C_{12}H_{11}F_6O_2$ = 301.06578, found = 301.06518.
IR (neat): 2981, 2973, 1753, 1457, 1386, 1355, 1275, 1260, 1230, 1192, 1096 cm^{-1}.

1,1,1,3,3,3-hexafluoropropan-2-yl 3-ethylbenzoate

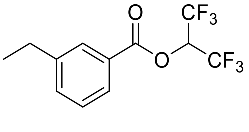

Prepared according to the general procedure using 3-ethylbenzoic acid (0.15 g, 1.0 mmol). The title compound (0.13 g, 0.43 mmol, 43%) was obtained as colorless liquid.

^{1}H NMR (500 MHz, CDCl$_3$) δ = 7.97-7.91 (m, 2H), 7.53-7.50 (m, 1H), 7.46-7.40 (m, 1H), 6.02 (hept, *J* = 6.1 Hz, 1H), 2.74 (q, *J* = 7.6 Hz, 2H), 1.28 (t, *J* = 7.6 Hz, 3H) ppm.
^{13}C NMR (126 MHz, CDCl$_3$) δ 163.6, 145.3, 134.6, 130.0, 129.0, 128.0, 127.0, 120.8 (q, *J* = 284.3 Hz), 67.0 (hept. *J* = 34.8 Hz), 28.8, 15.6 ppm.
^{19}F NMR (471 MHz, CDCl$_3$) δ = −73.2 ppm.
HRMS (ESI-pos): Calcd. for $C_{12}H_{11}F_6O_2$ = 301.06578, found = 301.06541.
IR (neat): 2972, 1753, 1590, 1386, 1357, 1292, 1275, 1225, 1198, 1105 cm^{-1}.

1,1,1,3,3,3-hexafluoropropan-2-yl 4-ethylbenzoate

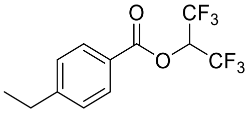

Prepared according to the general procedure using 4-ethylbenzoic acid (1.50 g, 10.0 mmol). The title compound (0.523 g, 1.74 mmol, 17%) was obtained as colorless liquid.

^{1}H NMR (500 MHz, CDCl$_3$) δ = 8.07-8.01 (m, 2H), 7.37-7.30 (m, 2H), 6.01 (hept, *J* = 6.2 Hz, 1H), 2.74 (q, *J* = 7.6 Hz, 2H), 1.27 (t, *J* = 7.6 Hz, 3H) ppm.
^{13}C NMR (126 MHz, CDCl$_3$) δ = 163.4, 152.2, 130.8, 128.6, 124.4, 120.8 (q, *J* = 284.7 Hz), 66.9 (hept. *J* = 34.9 Hz), 29.3, 15.3 ppm.
^{19}F NMR (471 MHz, CDCl$_3$) δ = −73.2 ppm.
HRMS (ESI-pos): Calcd. for $C_{12}H_{11}F_6O_2$ = 301.06578, found = 301.06536.
IR (neat): 2972, 1751, 1613, 1386, 1356, 1293, 1259, 1225, 1196, 1178, 1100 cm^{-1}.

2,2,2-trifluoroethyl 4-ethylbenzoate

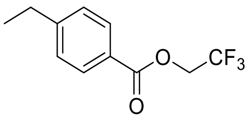

Prepared according to the general procedure using 4-ethylbenzoic acid (1.50 g, 10.0 mmol, 1.0 equiv) and 2,2,2-trifluoroethanol (2.2 mL, 30.0 mmol, 3.0 equiv) instead of HFIP. The title compound (0.598 g, 2.58 mmol, 26%) was obtained as colorless liquid.

^{1}H NMR (500 MHz, CDCl$_3$) δ = 8.03 – 7.97 (m, 2H), 7.33 – 7.27 (m, 2H), 4.69 (q, *J* = 8.5 Hz, 2H), 2.72 (q, *J* = 7.6 Hz, 2H), 1.26 (t, *J* = 7.6 Hz, 3H) ppm.
^{13}C NMR (126 MHz, CDCl$_3$) δ = 165.2, 151.1, 130.3, 128.3, 126.0, 123.3 (q, *J* = 280.0 Hz), 60.8 (q, *J* = 36.5 Hz), 29.2, 15.3 ppm.
^{19}F NMR (471 MHz, CDCl$_3$) δ = –73.7 ppm.
HRMS (EI): Calcd. for $C_{11}H_{11}F_3O_2$ = 232.07111, found = 232.07101.
IR (neat): 2971, 2938, 2879, 1734, 1612, 1576, 1511, 1455, 1416, 1291, 1255, 1162, 1104 cm^{-1}.

3.2 Synthesis of Ligands

General procedure for the synthesis of thioether-based ligands: Synthesized according to a modified procedure from Yu *et al.*[3] The corresponding thiophenol (1.0 equiv), 2-methyl-2-oxazoline, and dry toluene (1 M) were added to a Schlenk-flask. The flask was tightly sealed and placed in a pre-heated aluminum-block, where the reaction mixture was stirred at 100 °C overnight. The crude compound was directly absorbed on silica using a rotary evaporator and purified by flash column chromatography.

***N*-(2-((3,5-dimethylphenyl)thio)ethyl)acetamide**

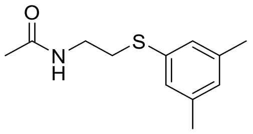

Prepared according to the general procedure using 3,5-dimethylthiophenol (1.38 g, 1.40 mL, 10.0 mmol), 2-methyl-2-oxazoline (0.85 mL, 10 mmol), and toluene (10 mL). Purification by flash column chromatography (CH_2Cl_2) afforded the title compound (0.481 g, 2.16 mmol, 22%) as colorless solid.

^{1}H NMR (500 MHz, CDCl$_3$) δ = 7.00-6.99 (m, 2H), 6.85-6.83 (m, 1H), 5.83 (s, 1H), 3.48-3.44 (m, 2H), 3.05-3.03 (m, 2H), 2.29-2.28 (m, 6H), 1.94 (s, 3H) ppm.
^{13}C NMR (126 MHz, CDCl$_3$) δ = 170.2, 138.9, 134.5, 128.6, 127.6, 38.8, 33.8, 23.4, 21.4 ppm.
HRMS (ESI-pos): Calcd. for $C_{12}H_{18}NOS$ = 224.11036, found = 224.11009.
IR (neat): 3210, 3006, 2973, 2864, 1739, 1632, 1577, 1437, 1371, 1276, 1261 cm^{-1}.
Melting point: 59 °C

***N*-(2-((4-trifluoromethyl)thio)ethyl)acetamide**

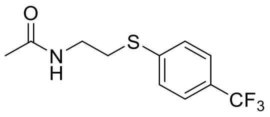

Prepared according to the general procedure using 4-trifluoromethylthiophenol (1.85 g, 1.40 mL, 10.4 mmol), 2-methyl-2-oxazoline (0.85 mL, 10 mmol), and toluene (10 mL). Purification by flash column chromatography (CH_2Cl_2 → CH_2Cl_2:MeOH = 97:3) afforded the title compound (2.24 g, 8.51 mmol, 82%) as colorless solid.

^{1}H NMR (500 MHz, CDCl$_3$) δ = 7.55-7.52 (m, 2H), 7.44-7.41 (m, 2H), 5.83 (s, 1H), 3.51-3.48 (m, 2H), 3.14 (t, J = 6.6 Hz, 2H), 1.96 (s, 3H) ppm.
^{13}C NMR (126 MHz, CDCl$_3$) δ = 170.3, 140.8, 127.8, 125.9 (m), 38.6, 32.2, 23.2 ppm. *Note:* Signal of the CF_3 could not be resolved.
^{19}F NMR (471 MHz, CDCl$_3$) δ = −62.5 ppm.
HRMS (ESI-pos): Calcd. for $C_{11}H_{13}F_3NOS$ = 264.06645, found = 264.06603.
IR (neat): 3287, 3094, 3005, 2980, 2917, 1737, 1636, 1609, 1553, 1438, 1332, 1293 cm^{-1}.
Melting point: 101 °C (decomp.)

***N*-(2-((3 -trifluoromethyl)thio)ethyl)acetamide (BL1)**

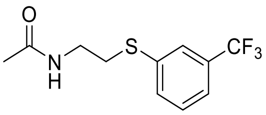

Prepared according to the general procedure using 3-trifluoromethylthiophenol (2.00 g, 1.60 mL, 11.2 mmol), 2-methyl-2-oxazoline (0.92 mL, 11 mmol), and toluene (12 mL). Purification by flash column chromatography (CH_2Cl_2 → CH_2Cl_2:EtOAc = 90:10) afforded the title compound (**BL1**) (2.58 g, 9.8 mmol, 88%) as colorless solid.

^{1}H NMR (500 MHz, $CDCl_3$) δ = 7.60-7.58 (m, 1H), 7.56-7.53 (m, 1H), 7.46 – 7.40 (m, 2H), 5.82 (s, 1H), 3.50-3.46 (m, 2H), 3.13 (t, *J* = 6.5 Hz, 2H), 1.96 (s, 3H) ppm.
^{13}C NMR (126 MHz, $CDCl_3$) δ = 170.4, 137.1, 132.1, 131.5, 129.7, 125.7 (d, *J* = 3.4 Hz), 123.1 (d, *J* = 3.4 Hz), 38.8, 33.2, 23.3 ppm. Note: Signals of the CF_3-group could not be resolved.
^{19}F NMR (471 MHz, $CDCl_3$) δ = −62.9 ppm.
HRMS (ESI-pos): Calcd. for $C_{11}H_{13}F_3NOS$ = 264.06645, found = 264.06600.
IR (neat): 3311, 3082, 2937, 1633, 1552, 1419, 1364, 1325, 1273, 1159 cm^{-1}.
Melting point: 60 °C

***N*-(2-((3,5-bis(trifluoromethyl)phenyl)thio)ethyl)acetamide**

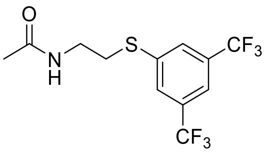

Prepared according to the general procedure using 3,5-bis-(trifluoromethyl)-thiophenol (1.23 g, 5.00 mmol), 2-methyl-2-oxazoline (0.43 mL, 5.0 mmol), and toluene (5 mL).. Purification by flash column chromatography (CH_2Cl_2 → CH_2Cl_2:MeOH = 99:1) afforded the title compound (1.45 g, 4.38 mmol, 88%) as colorless solid. Spectral data matches those reported in literature.[4]

^{1}H NMR (500 MHz, $CDCl_3$) δ = 7.76-7.75 (m, 2H), 7.66-7.65 (m, 1H), 5.83 (s, 1H), 3.53-3.49 (m, 2H), 3.20 (t, *J* = 6.6 Hz, 2H), 1.98 (s, 3H) ppm.
^{13}C NMR (126 MHz, $CDCl_3$) δ = 170.5, 139.8, 132.4 (q, *J* = 33.9 Hz), 127.8, 123.1 (q, *J* = 274.0 Hz), 119.7 (m), 38.7, 32.7, 23.3 ppm.
^{19}F NMR (471 MHz, $CDCl_3$) δ = −63.1 ppm.
HRMS (ESI-pos): Calcd. for $C_{12}H_{12}F_6NOS$ = 332.05383, found = 332.05315.
IR (neat): 3298, 2982, 1739, 1649, 1543, 1355, 1277 cm^{-1}.
Melting point: 88 °C (decomp.)

_N_-(2-((3-(trifluoromethyl)phenyl)sulfinyl)ethyl)acetamide

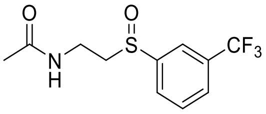

Prepared in analogy to a report from Jiao *et al.*[5] A 100 mL round-bottom flask was charged with *N*-(2-((3 -trifluoromethyl)thio)ethyl)acetamide (**BL1**) (0.500 g, 1.90 mmol, 1.0 equiv) and CH_2Cl_2 (38 mL, 0.05 M) under air atmosphere. The mixture was stirred until all solids were dissolved and subsequently cooled to 0 °C using an ice-bath. *Meta*-Chloroperoxybenzoic acid (0.468 g of a 70% w/w mixture with 3-chlorobenzoic acid and water, 1.90 mmol, 1.0 equiv) was added in small portions and the reaction mixture was stirred at 0 °C for 1 h. Afterwards the mixture was transferred to a separatory funnel and washed with saturated aq. $NaHCO_3$-solution (2 × 30 mL). The combined aq. phases were extracted with CH_2Cl_2 (1 × 30 mL). The combined organic phases were dried with anhydrous $MgSO_4$, filtered and directly absorbed on silica using a rotary evaporator. Purification by flash column chromatography (CH_2Cl_2:EtOAc = 90:10 → CH_2Cl_2:EtOAc:MeOH = 87:10:3) afforded the title compound as colorless solid (0.323 g, 1.16 mmol, 61%).

^{1}H NMR (500 MHz, DMSO-*d*$_6$) δ = 8.06-8.03 (m, 2H), 8.01-7.98 (m, 1H), 7.94-7.91 (m, 1H), 7.85-7.81 (m, 1H), 3.41-3.34 (m, 1H), 3.30-3.24 (m, 1H), 3.19-3.14 (m, 1H), 3.00-2.95 (m,1H), 1.74 (s, 3H) ppm.
^{13}C NMR (126 MHz, DMSO-*d*$_6$) δ = 169.4, 145.9, 130.5, 129.9 (q, *J* = 31.9 Hz), 128.2, 127.5, 127.4, 123.8 (q, *J* = 273.4 Hz), 120.7, 120.7, 55.0, 32.0, 22.4 ppm.
^{19}F NMR (471 MHz, DMSO-*d*$_6$) δ = −61.8 ppm.
HRMS (ESI-pos): Calcd. for $C_{11}H_{13}F_3NO_2S$ = 280.06136, found = 280.06106.
IR (neat): 3260, 3212, 3066, 3005, 2990, 1740, 1663, 1557, 1437, 1372, 1321, 1275 cm^{-1}.
Melting point: 87 °C

3.3 Synthesis of Starting Materials

tert-butyl(3-((tert-butyldimethylsilyl)oxy)phenyl)dimethylsilane (1s)

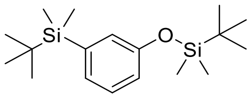

The synthesis was performed over two steps, wherein the first step was performed as described by Snieckus *et al.*[6] Under nitrogen atmosphere 3-Bromophenol (2.31 g, 13.4 mmol, 1.0 equiv) was dissolved in dry THF (20 mL). The mixture was stirred until everything had dissolved and subsequently cooled to -78 °C using a dry ice/acetone-bath. *tert*-Butyllithium (25 mL of a 1.7 M solution in pentane, 42.5 mmol, 3.2 equiv) was added dropwise via syringe and stirring was continued for 30 min at -78 °C. A solution of *tert*-butyldimethylsilyl chloride (2.37 g, 15.8 mmol, 1.2 equiv) in dry THF (4 mL) was slowly added to the reaction mixture via syringe and the bright yellow solution was stirred at -78 °C for 3 h. The cooling bath was removed and the mixture was stirred at room temperature for further 2.5 h. After cooling to 0 °C using an ice-bath, the reaction mixture was quenched by the slow addition of saturated NH_4Cl-solution (20 mL) and further acidified by the addition of 1M HCl (20 mL). The organic phase was separated in a separatory funnel and the aqueous phase was extracted with EtOAc (2 × 30 mL). The combined organic phases were dried over anhydrous Na_2SO_4, filtered, and directly absorbed on silica using a rotary evaporator. Purification by flash-column chromatography (pentane:EtOAc = 90:10) afforded 3-[*tert*-butyl(dimethyl)silyl]phenol (1.12 g, 5.40 mmol, 40%) as colorless solid. Spectral data matches the ones described in literature.[6]

The second step was done in analogy to a report from Zuilhof *et al.*[7] 3-[*tert*-butyl(dimethyl)silyl]phenol (1.12 g, 5.40 mmol, 1.0 equiv) and imidazole (0.810 g, 11.9 mmol, 2.2 equiv) were dissolved in dry CH_2Cl_2 (7 mL). *Tert*-butyldimethylsilyl chloride (0.859 g, 5.7 mmol, 1.1 equiv) was added and the mixture was stirred at room temperature overnight. The reaction mixture was transferred to a separatory funnel using Et_2O (20 mL) and the organic phase was extracted with water (3 × 20 mL). The organic phase was dried over anhydrous Na_2SO_4, filtered and directly absorbed on silica using a rotary evaporator. Purification by flash-column chromatography (pentane:Et_2O = 99:1) afforded *tert*-butyl(3-((tert-butyldimethylsilyl)oxy)phenyl)dimethylsilane (**1s**) (1.45 g, 4.49 mmol, 83%) as colorless oil.

^{1}H NMR (500 MHz, $CDCl_3$) δ = 7.21 (m, 1H), 7.08 (m,1H), 6.97 (m, 1H), 6.83 (ddd, *J* = 8.0, 2.6, 1.1 Hz, 1H), 0.99 (s, 9H), 0.87 (s, 9H), 0.25 (s, 6H), 0.19 (s, 6H) ppm.
^{13}C NMR (126 MHz, $CDCl_3$) δ = 155.0, 139.5, 128.7, 127.5, 126.0, 120.7, 26.7, 25.9, 18.4, 17.0, −4.2, −6.1 ppm.
HRMS (ESI-pos): Calcd. for $C_{18}H_{35}OSi_2$ = 323.22210, found = 323.22193.
IR (neat): 2954, 2929, 2885, 2857, 1586, 1568, 1472, 1463, 1396, 1362, 1257, 1238. cm^{-1}

2-((2,3-dimethylbenzyl)oxy)-1,3,3-trimethylbicyclo[2.2.1]heptane (1z)

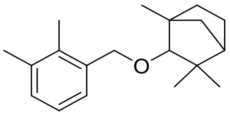

Inspired by a procedure from Xia *et al.*[8] Under N_2-atmosphere a 100 mL Schlenk-flask was charged with a stirring bar, fenchol (mixture of isomers (natural ≥97%, Sigma-Aldrich), 6.5 mmol, 1.0 g, 1.0 equiv), and dry THF (20 mL). The mixture was cooled to 0 °C using an ice-bath, NaH (60% dispersion in mineral oil, 8.5 mmol, 0.34 g, 1.3 equiv) was slowly added and the mixture was stirred for 10 min at 0 °C. 1-(bromomethyl)-2,3-dimethylbenzene (6.5 mmol, 1.3 g, 1.0 equiv) was added in portions, the ice-bath was removed and the mixture was stirred at room temperature for 48 h. The reaction was quenched carefully with distilled water (20 mL), transferred to a separatory funnel with CH_2Cl_2 (20 mL), and the layers were separated. The aqueous phase was extracted with CH_2Cl_2 (2 x 20 mL), the combined organic phases were dried over anhydrous Na_2SO_4, filtered, and directly adsorbed on silica using a rotary evaporator. Purification by flash column chromatography (pentane:Et_2O = 97:3) afforded the title compound (**1z**) as colorless oil (1.53 g, 5.6 mmol, 86%).

^{1}H NMR (600 MHz, $CDCl_3$) δ = 7.27 – 7.22 (m, 1H), 7.13 – 7.05 (m, 2H), 4.55 (d, *J* = 11.7 Hz, 1H), 4.45 (d, *J* = 11.7 Hz, 1H), 3.05 (dd, *J* = 1.9, 0.5 Hz, 1H), 2.30 (s, 3H), 2.23 (s, 3H), 1.85 – 1.76 (m, 1H), 1.74 – 1.66 (m, 1H), 1.66 – 1.62 (m, 1H), 1.50 – 1.44 (m, 1H), 1.44 – 1.34 (m, 1H), 1.12 – 1.07 (m, 4H), 1.07 – 0.94 (m, 7H) ppm.
^{13}C NMR (151 MHz, $CDCl_3$) δ = 137.4, 136.8, 135.0, 129.2, 126.5, 125.3, 92.9, 72.6, 49.4, 49.0, 41.6, 39.7, 31.9, 31.8, 26.3, 26.2, 21.0, 20.5, 20.3, 14.9 ppm.
HRMS (EI): Calcd. for $C_{19}H_{28}O_1$ = 272.21401, found = 272.21404.
IR (neat): 2948, 2925, 2868, 1460, 1384, 1348, 1126, 1113, 1089, 1070, 1033 cm^{-1}.

Methyl 2-methyl-4'-(trifluoromethoxy)-[1,1'-biphenyl]-3-carboxylate (1ac)

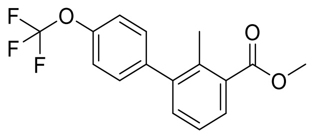

In a 100 mL round bottom flask, MeI (0.62 mL, 1.4 g, 10 mmol, 5.0 equiv.) was added to a solution of 2-methyl-4'-(trifluoromethoxy)-[1,1'-biphenyl]-3-carboxylic acid (592 mg, 2.00 mmol, 1.0 equiv) and K_2CO_3 (1.11 g, 8.00 mmol, 4.0 equiv.) in acetone (20 mL, 0.1 M) at room temperature. After stirring the reaction mixture at room temperature for 16 h, saturated $NaHCO_3$ solution (20 mL) was added and the resulting mixture was extracted with EtOAc (3 x 30 mL). The combined organic phases were filtered through Na_2SO_4, concentrated under reduced pressure, and the remaining residue was purified by silica gel column chromatography using pentane:EtOAc = 98:2 as the eluent. The target compound Methyl 2-methyl-4'-(trifluoromethoxy)-[1,1'-biphenyl]-3-carboxylate (**1ac**) was obtained as colorless liquid (590 mg, 1.90 mmol, 95%). The observed analytical data are in accordance with the ones reported in literature.[9]

^{1}H NMR (500 MHz, Acetone-d$_6$) δ 7.81 (dd, *J* = 7.6, 0.4 Hz, 1H), 7.50 – 7.45 (m, 2H), 7.45 – 7.33 (m, 4H), 3.88 (s, 3H), 2.37 (s, 3H) ppm.
^{13}C NMR (126 MHz, Acetone-d$_6$) δ 168.9, 149.2, 143.1, 141.6, 136.9, 133.9, 132.9, 132.0, 130.3, 126.5, 121.7, 121.6 (q, *J* = 256.8 Hz), 52.3, 18.5 ppm.
^{19}F NMR (471 MHz, Acetone-d$_6$) δ −57.5 ppm.
HRMS (ESI-pos) m/z: Calcd for $C_{16}H_{14}O_3F_3$ 311.08896, Found 311.08851.

4. Scope of the Reaction

4.1. Scope shown in the main manuscript

General procedure for scope studies: An oven-dried 25 mL Schlenk-flask was charged with a stirring bar, $Pd(MeCN)_2Cl_2$ (5.2 mg, 0.020 mmol, 10 mol%), *N*-(2-((3-trifluoromethyl)thio)ethyl)acetamide (**BL1**) (21.0 mg, 0.0800 mmol, 40 mol%), 3-trifluoromethylquinoline (**ML1**) (7.9 mg, 0.040 mmol, 20 mol%), $AgNO_3$ (135.8 mg, 0.8000 mmol, 4.0 equiv), $Mo(CO)_6$ (17.4 mg, 0.066 mmol, 0.33 equiv), and HFIP (2.0 mL). The flask was tightly sealed and the mixture was stirred at room temperature for approximately 10 min. The respective substrate (0.200 mmol) was added, the flask was tightly sealed, placed in a pre-heated oil-bath at 80 °C, and the mixture was stirred with 1000 rpm for 24 h. After cooling to room-temperature the mixture was filtered through a pad of celite and eluted with CH_2Cl_2 (70 mL). The solvent was evaporated and the crude mixtures were either directly subjected to flash-column chromatography on silica or hydrolyzed. For the latter, the mixtures were treated with a mixture of THF or MeOH (2 mL) and NaOH (aq., 2M, 2 mL) and stirred at room temperature overnight (the procedure for hydrolysis was inspired by Yu et al.[41]). Further treatment is described in the respective scope entries.

Note: The quality of HFIP, a fast and homogeneous heating rate and sufficient head-space to avoid an excess of dissolved gases (CO, NO_X) were found to be crucial for obtaining optimal results during scope-studies. Therefore, an oil-bath was used for heating and care was taken that the headspace of the flask was at least at a ratio of 1:10 (i.e., 1.0 mL of the reaction mixture in a 10 mL Schlenk-flask or 2.0 mL of the reaction mixture in a 25 mL Schlenk-flask).

Benzoic acid (4b)

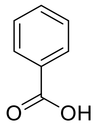

4b

Following the general procedure using benzene (**2b**) (15.6 mg, 0.200 mmol). After hydrolysis in MeOH:NaOH (aq., 2M) = 1:1, the aqueous phase was acidified with HCl (1M) until pH = 1 and extracted with CH_2Cl_2 (3 x 20 mL). The organic layer was dried over anhydrous Na_2SO_4, filtered, and directly absorbed on silica using a rotary evaporator. Purification by flash column chromatography (pentane:EtOAc:HCOOH = 94.5:5:0.5) afforded the title compound as colorless solid (20.5 mg, 0.168 mmol = 84%). The observed analytical data are in accordance with the ones reported in literature.[10]

^{1}H NMR (500 MHz, $CDCl_3$) δ = 8.18 – 8.09 (m, 1H), 7.70 – 7.59 (m, 1H), 7.53 – 7.44 (m, 1H) ppm.
^{13}C NMR (126 MHz, $CDCl_3$) δ = 172.2, 134.0, 130.4, 129.5, 128.6 ppm.
HRMS (ESI-neg): Calcd. for C7H5O2- = 121.02950, found = 121.02940.

1,1,1,3,3,3-hexafluoropropan-2-yl ethylbenzoate (2a)

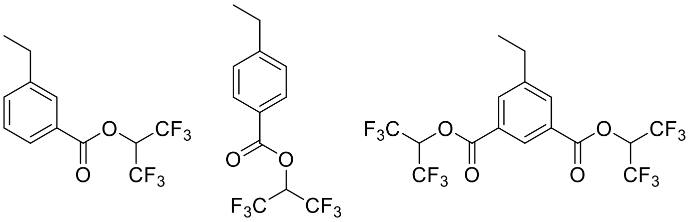

2a-m **2a-p** **2a-di**

Following the general procedure using ethylbenzene (**1a**) (21.2 mg, 0.200 mmol). Purification by flash column chromatography (pentane → pentane:Et_2O = 98:2) afforded a mixture of the shown isomers as colorless oil (45.9 mg, containing 40.7 mg (0.136 mmol, 68% of **2a-m** and **2a-p** (m:p = 65:35), and 5.2 mg (0.011 mmol, 5% of **2a-di**), meta:para:di = 60:33:7). The assignment was achieved by comparison with the authentic samples synthesized within this work.

^{1}H NMR (500 MHz, $CDCl_3$) δ = 8.63 (m, 0.12H, 2a-di), 8.23 – 8.19 (m, 0.25H, 2a-di), 8.06 – 8.01 (m, 1.10H, 2a-p), 7.94 (m, 2H, 2a-m), 7.51 (m, 1H, 2a-m), 7.46 – 7.40 (m, 1H, 2a-m), 7.37 – 7.31 (m, 1.13H, 2a-p), 6.02 (m, 1.88H), 2.84 (q, J = 7.6 Hz, 0.3H, 2a-di), 2.78 – 2.70 (m, 3.19H), 1.34 (t, J = 7.9 Hz, 0.5H, 2a-di), 1.29 – 1.23 (m, 3.86H) ppm.
^{13}C NMR (126 MHz, $CDCl_3$) δ = 163.5, 163.3, 162.3, 152.1, 146.6, 145.2, 135.5, 134.5, 130.7, 129.8, 128.8, 128.4, 128.0, 127.9, 126.8, 124.2, 120.6 (q, J = 283.6 Hz), 120.4 (q, J = 281.8 Hz), 67.5 – 66.3 (m), 29.1, 28.6, 28.5, 15.4, 15.2, 15.1 ppm.
^{19}F NMR (471 MHz, $CDCl_3$) δ = −73.2, −73.2, −73.3 ppm.
HRMS (ESI-pos): Calcd. for $C_{12}H_{11}F_6O_2$ = 301.06578, found = 301.06513.

Chlorobenzenoic acid (4c)

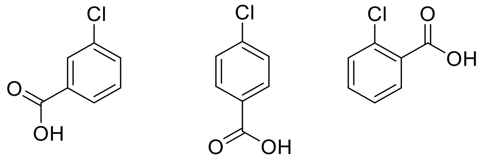

4c-m **4c-p** **4c-o**

Following the general procedure using chlorobenzene (**1c**) (22.5 mg, 0.145 mmol). After hydrolysis in MeOH:NaOH (aq., 2M) = 1:1, the aqueous phase was acidified with HCl (1M) until pH = 1 and extracted with CH_2Cl_2 (3 x 20 mL). The organic layer was dried over anhydrous Na_2SO_4, filtered, and directly absorbed on silica using a rotary evaporator. Purification by flash column chromatography (pentane:EtOAc:HCOOH = 94.5:5:0.5) afforded the title compound as colorless solid (22.7 mg, containing 11.4 mg (0.072 mmol, 36%) of **4c-m**, 5.7 mg (0.036 mmol, 18%) of **4c-o** and 5.7 mg (0.036 mmol, 18%) of **4c-p**, 0.144 mmol, 72%, o:m:p = 25:50:25). The observed analytical data are in accordance with the ones reported in literature.[11]

^{1}H NMR (600 MHz, DMSO-d_6) δ = 7.97 – 7.91 (m, 1.03H, 4c-p), 7.91 – 7.88 (m, 2H, 4c-m), 7.77 (ddd, J = 7.7, 1.5, 0.7 Hz, 1H, 4c-o), 7.71 – 7.67 (m, 1H, 4c-m), 7.59 – 7.54 (m, 1.05H, 4c-p), 7.54 – 7.53 (m, 1.04H, 4c-o), 7.53 – 7.51 (m, 1H, 4c-m), 7.42 (ddd, J = 7.7, 6.3, 2.3 Hz, 0.49H,4c-o) ppm.
^{13}C NMR (151 MHz, DMSO-d_6) δ = 166.8, 166.5, 166.1, 137.8, 133.3, 133.0, 132.7, 132.5, 131.7, 131.5, 131.2, 130.8, 130.7, 130.6, 130.1, 129.7, 128.8, 128.8, 127.9, 127.2 ppm.

HRMS (ESI-pos): Calcd. for $C_7H_6ClO_2^+$= 157,00508, found = 157.00492.

Bromobenzoic acid (4d)

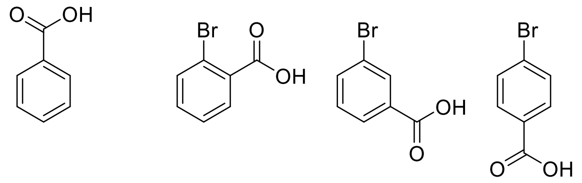

4d-ipso **4d-o** **4d-m** **4d-p**

Following the general procedure using bromobenzene (31.4 mg, 0.200 mmol). After hydrolysis in MeOH:NaOH (aq., 2M) = 1:1, the aqueous phase was extracted with CH_2Cl_2 (3 x 20 mL) before acidifying with HCl (1M) until pH = 1 and extracting again with CH_2Cl_2 (3 x 20 mL). The organic layer was dried over anhydrous Na_2SO_4, filtered, and directly absorbed on silica using a rotary evaporator. Purification by flash column chromatography (pentane:EtOAc:HCOOH = 94.5:5:0.5) afforded the title compound as colorless solid (24.1 mg, containing 1.5 mg (0.012 mmol, 6%) of **4d-ipso** and 22.6 mg (0.112 mmol, 56%) of **4d-o** + **4d-m** + **4d-p** (o:m:p = 31:49:20), ipso:o:m:p = 10:28:44:18). Spectral data are in accordance with the literature.[12,13]

^{1}H NMR (600 MHz, CDCl$_3$) δ = 8.26 (m, 1.00H, 4d-m), 8.13 – 8.12 (m, 0.23H, 4d-ipso), 8.06 – 8.04 (m, 1.00H, 4d-m), 8.02 – 7.99 (m, 0.61H, 4d-o), 7.99 – 7.96 (m, 0.83H, 4d-p), 7.76 – 7.74 (m, 1.04H, 4d-m), 7.73 – 7.70 (m, 0.63H, 4d-o), 7.65 – 7.61 (m, 0.95H, 3-ipso+ 4d-p), 7.50 – 7.49 (m, 0.23H, 4d-ipso), 7.44 – 7.34 (m, 2.29H, 4d-o + 4d-p + 4d-m) ppm.

^{13}C NMR (151 MHz, CDCl$_3$) δ = 172.3, 171.5, 171.2, 170.8, 137.0, 135.0, 134.1, 133.7, 133.4, 132.5, 132.1, 131.9, 131.3, 130.6, 130.4, 130.2, 129.4, 129.3, 128.9, 128.7, 128.2, 127.4, 122.7, 122.7 ppm.

HRMS (ESI-neg): Calcd. for $C_7H_4O_2{}^{79}Br$ = 198.94002, found = 198.93935.

1,1,1,3,3,3-hexafluoropropan-2-yl ((tert-butyldimethylsilyl)oxy)benzoate (2e)

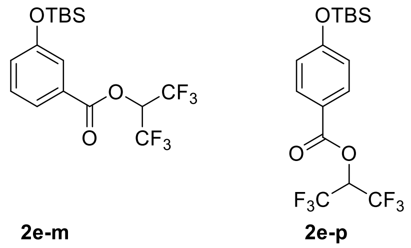

Following the general procedure using *tert*-butyldimethyl(phenoxy)silane (41.7 mg, 0.200 mmol). Purification by flash column chromatography (pentane) afforded the title compound as colorless oil (58.6 mg, 0.146 mmol, 73%, m:p = 65:35).

^{1}H NMR (500 MHz, CDCl$_3$) δ = 8.05 – 7.99 (m, 1.07H, 2e-p), 7.71 (ddd, *J* = 7.8, 1.7, 1.0 Hz, 1.00H, 2e-m), 7.57 – 7.52 (m, 0.98H, 2e-m), 7.40 – 7.34 (m, 1.03H, 2e-m), 7.14 (ddd, *J* = 8.2, 2.5, 1.0 Hz, 1.01H, 2e-m), 6.95 – 6.89 (m, 1.09H, 2e-p), 6.03 – 5.96 (m, 1.56H), 1.01 – 0.99 (m,14H), 0.25 (s, 3H, 2e-p), 0.23 (s, 6H, 2e-m) ppm.
^{13}C NMR (151 MHz, CDCl$_3$) δ = 163.2, 163.0, 161.9, 156.2, 132.9, 130.1, 128.2, 126.8, 123.5, 121.8, 120.8 (q, *J* = 287.5 Hz), 120.7 (q, *J* = 285.3 Hz) 120.5, 119.7, 67.6 – 66.4 (m), 25.8, 25.7, 18.4(2), 18.4, −4.2, −4.3 ppm.
^{19}F NMR (471 MHz, CDCl$_3$) δ = −73.3 (2e-m + 2e-p) ppm.
HRMS (EI): Calcd. for $C_{16}H_{20}F_6O_3{}^{28}Si$ = 402.10859, found = 402.10867.
IR (neat): 2959, 2935, 2863, 1754, 1601, 1510, 1487, 1474, 1440, 1387, 1356, 1277, 1258, 1193 cm^{-1}.

1,1,1,3,3,3-hexafluoropropan-2-yl 3-(trimethylsilyl)benzoate (2f)

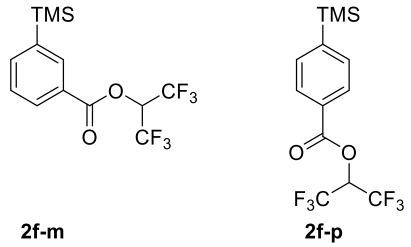

Following the general procedure using trimethyl(phenyl)silane (30.1 mg, 0.200 mmol). Purification by flash column chromatography (pentane) afforded the title compound as colorless oil (30.8 mg, 0.0895 mmol, 45%, m:p = 68:32).

^{1}H NMR (500 MHz, CDCl$_3$) δ = 8.27 – 8.22 (m, 0.95H, 2f-m), 8.11 – 8.04 (m, 1.91H, 2f-m + 2f-p), 7.82 (dt, *J* = 7.3, 1.3 Hz, 0.98H, 2f-m), 7.69 – 7.64 (m, 0.96H, 2f-p), 7.50 (ddd, *J* = 7.9, 7.3, 0.6 Hz, 1H, 2f-m), 6.08 – 5.98 (m, 1.45H, 2f-m + 2f-p), 0.32 (s, 8H, 2f-m), 0.31 (s, 4H, 2f-p) ppm.
^{13}C NMR (151 MHz, CDCl$_3$) δ = 163.8, 163.6, 149.8, 142.2, 139.8, 135.4, 133.9, 130.8, 129.4, 128.3, 127.0, 126.3, 120.7 (q, *J* = 282.2 Hz), 67.1 (hept, *J* = 34.9 Hz), –1.2, –1.3 ppm.
^{19}F NMR (471 MHz, CDCl$_3$) δ = –73.2, –73.2 ppm.
HRMS (EI): Calcd. for $C_{13}H_{14}F_6O_2Si_1$ = 344.06672, found = 344.06677.
IR (neat): 2960, 1753, 1387, 1355, 1293, 1250, 1197, 1102 cm^{-1}.

2',3',4',5',6'-pentafluoro-[1,1'-biphenyl]-carboxylic acid (4g)

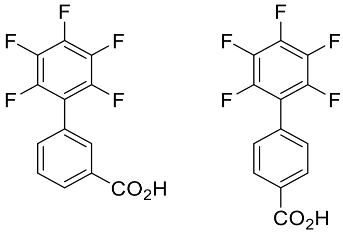

4g-m **4g-p**

Following the general procedure using 2,3,4,5,6-pentafluorbiphenyl (48.8 mg, 0.200 mmol, 1.0 equiv). After hydrolysis in THF:NaOH (aq., 2M) = 1:1, the aqueous phase was acidified with HCl (aq., 1M) until pH = 1 and extracted with CH_2Cl_2 (3 x 20 mL). The organic layer was dried over anhydrous Na_2SO_4, filtered, and directly absorbed on silica using a rotary evaporator. Purification by flash column chromatography (pentane:EtOAc:HCOOH = 94.5:5:0.5) afforded the title compound as colorless solid (41.5 mg, 0.144 mmol, 72%, m:p = 69:31).

^{1}H NMR (500 MHz, Acetone-d$_6$) δ = 8.23 – 8.15 (m, 3.28H, 4g-m + 4g-p), 7.89 – 7.80 (m, 1.13H, 4g-m), 7.75 – 7.71 (m, 1.16H, 4g-m), 7.71 – 7.67 (m, 1.00H, 4g-p) ppm.
^{13}C NMR (126 MHz, Acetone-d$_6$) δ = 167.1, 167.0, 146.2, 144.2, 142.6, 140.6 139.8, 137.8, 135.5, 132.3, 132.2, 131.5, 131.4, 130.8, 130.1, 127.8, 116.2, 116.0 ppm.
^{19}F NMR (471 MHz, Acetone-d$_6$) δ = −143.50 – −143.58 (m), −143.83 – −143.91 (m), −156.27– −156.36 (m), −156.68– −156.77 (m), −163.37– −163.63 (m) ppm. Note: The signals at around −63 ppm are most likely due to traces of co-eluted ligands. The content of these in the final product however is <5%.
HRMS (ESI-neg): Calcd. for $C_{13}H_4O_2F_5$ = 287.01369, found = 287.01384.
IR (neat): 2671, 2556, 1682, 1652, 1610, 1585, 1527, 1496, 1419, 1407, 1276 cm^{-1}.

Pivaloylbenzoic acid (4h)

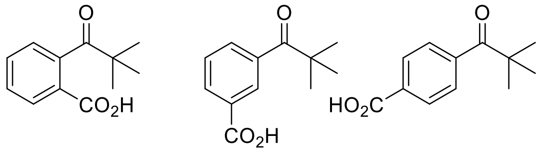

4h-o **4h-m** **4h-p**

Following the general procedure using 2,2-dimethylpropiophenon (32.4 mg, 0.200 mmol, 1.0 equiv) at 85 °C and with HFIP (1.2 mL). After hydrolysis in THF:NaOH (aq., 2M) = 1:1, the aqueous phase was extracted with CH_2Cl_2 (3 x 20 mL) before acidifying with HCl (1M) until pH = 1 and extracting again with CH_2Cl_2 (3 x 20 mL). The organic layer was dried over anhydrous Na_2SO_4, filtered, and directly absorbed on silica using a rotary evaporator. Purification by flash column chromatography (pentane:EtOAc:HCOOH = 94.5:5:0.5) afforded the title compound as slightly yellow solid (9.8 mg, 0.048 mmol, 24%, o:m:p = 8:64:28).

^{1}H NMR (600 MHz, $CDCl_3$) δ = 8.44 – 8.43 (m, 1.00H, 4h-m), 8.22 – 8.20 (m, 1.03H, 4h-m), 8.16 – 8.11 (m, 0.87H, 4h-p), 7.93 – 7.88 (m, 1.01H, 4h-m), 7.89 – 7.87 (m, 0.13H, 4h-o), 7.71 – 7.66 (m, 1.01H, 4h-p + 4h-o), 7.63 – 7.56 (m, 0.30H, 4h-o), 7.56 – 7.51 (m, 1.02H, 4h-m), 1.38 (s, 9.03H, 4h-m), 1.34 (s, 4.00H, 4h-p), 1.11 (s, 1.06H, 4h-o) ppm.
^{13}C NMR (151 MHz, $CDCl_3$) δ = 209.8, 208.4, 171.0, 170.9, 144.0, 139.1, 134.2, 133.2, 132.4, 130.9, 130.6, 130.1, 129.5, 129.3, 128.7, 127.5, 125.9, 124.0, 44.6, 44.5, 28.1, 27.8, 25.0 ppm.
HRMS (ESI-pos): Calcd. for $C_{12}H_{15}O_3$ = 207.10157, found = 207.10147.
IR (neat): 2671, 2556, 1682, 1652, 1610, 1585, 1527, 1496, 1419, 1407, 1276 cm^{-1}.

2-(diisopropylcarbamoyl)benzoic acid (4i)

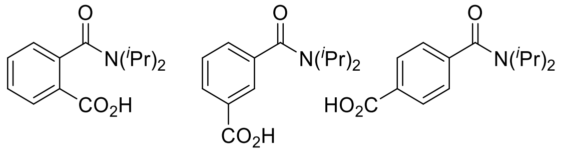

4i-o **4i-m** **4i-p**

Following the general procedure using *N*,*N*-diisopropylbenzamide (41.1 mg, 0.200 mmol, 1.0 equiv) at 85 °C and HFIP (1.2 mL). After hydrolysis in THF:NaOH (aq., 2M) = 1:1, the aqueous phase was extracted with CH_2Cl_2 (3 x 20 mL) before acidifying with HCl (1M) until pH = 1 and extracting again with CH_2Cl_2 (3 x 20 mL). The organic layer was dried over anhydrous Na_2SO_4, filtered, and directly absorbed on silica using a rotary evaporator. Purification by flash column chromatography (pentane:EtOAc:HCOOH = 84.5:15:0.5 → 59.5:40:0.5) afforded the title compound as slightly yellow solid (44.2 mg, 0.177 mmol, 89%, o:m:p = 90:6:4). Spectral data for **4i-o** are in accordance with the literature.[14]

^{1}H NMR (600 MHz, DMSO-d$_6$) δ = 8.01 – 7.93 (m, 0.21H, 4i-m + 4i-p), 7.89 (dd, *J* = 7.9, 1.3 Hz, 1.00H, 4i-o), 7.79 – 7.76 (m, 0.7H, 4i-m), 7.60 (td, *J* = 7.5, 1.3 Hz, 1.01H, 4i-o), 7.57 – 7.51 (m, 0.15H, 4i-p), 7.46 (td, *J* = 7.6, 1.3 Hz, 1.01H, 4i-o), 7.41 – 7.35 (m, 0.9H, 4i-p), 7.21 (dd, *J* = 7.6, 1.3 Hz, 1.00H, 4i-o), 3.53 – 3.42 (m, 3.32H), 1.44 (d, *J* = 6.7 Hz, 6.45H), 1.14 – 0.95 (m, 6.55H) ppm.
^{13}C NMR (151 MHz, DMSO-d$_6$) δ = 168.9, 167.1, 142.9, 140.4, 139.1, 132.5, 132.3, 131.1, 130.1, 129.6, 129.3, 129.0, 128.5, 128.0, 127.6, 126.0, 125.8, 125.5, 50.6, 44.4, 20.6, 20.3, 20.1, 19.6, 19.3 ppm.
HRMS (ESI-pos): Calcd. for $C_{14}H_{20}O_3N$ = 250.14377, found = 250.14360.
IR (neat): 2982, 1719, 1594, 1447, 1371, 1350 cm^{-1}.

(1,1,1,3,3,3-hexafluoropropan-2-yl) 4-methyl 1-methyl-1H-pyrrole-dicarboxylate (2j)

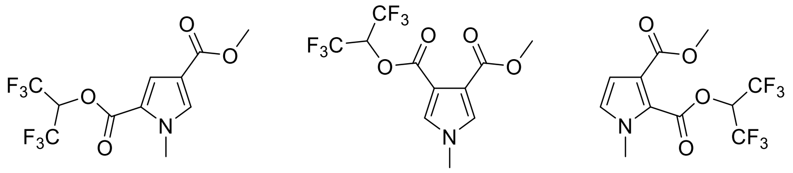

2j-α **2j-others**

Following the general procedure using methyl 1-methyl-1H-pyrrole-3-carboxylate (27.8 mg, 0.200 mmol, 1.0 equiv). Purification by flash column chromatography (pentane:EtOAc 8:2) afforded the title compound as a slightly orange oil (44.5 mg, containing 41.8 mg (0.126 mmol, 63%) of **2j-α** and 2.7 mg (0.008 mmol, 4%) of **2j-others**, 0.134 mmol, 67%, α:others = 94:6).

^{1}H NMR (500 MHz, CDCl$_3$) δ = 8.16 (d, *J* = 8.2 Hz, 0.03H, 2j-others), 7.74 (s, 0.03H, 2j-others), 7.62 (d, *J* = 0.6 Hz, 0.03H, 2j-others), 7.49 (d, *J* = 8.2 Hz, 0.03H, 2j-others), 7.38 (d, *J* = 2.5 Hz, 1H, 2j-α), 7.28 (d, *J* = 2.5 Hz, 1H, 2j-α), 5.94 (hept, *J* = 6.3 Hz, 1H, 2j-α), 3.82 (s, 3H, 2j-α), 3.72 (s, 3H, 2j-α) ppm.
^{13}C NMR (151 MHz, CDCl$_3$) δ = 163.4, 158.8, 131.3, 130.3, 120.8 (q, *J* = 283.1 Hz), 117.1, 111.7, 66.2 (p, *J* = 34.7 Hz), 51.8, 37.2 ppm.
^{19}F NMR (471 MHz, CDCl$_3$) δ = –73.2(0), −73.2(1) ppm.
HRMS (ESI-pos): Calcd. for $C_{11}H_{10}F_6NO_4$ = 334,05085, found = 334.05019.
IR (neat): 2965, 1764, 1719, 1551, 1454, 1390, 1353, 1260, 1221, 1185, 1167, 1089 cm^{-1}.

(1,1,1,3,3,3-hexafluoropropan-2-yl) 5-methyl thiophene-dicarboxylate (2k)

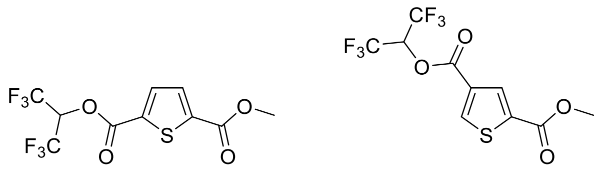

Following the general procedure using methyl thiophene-2-carboxylate (28.4 mg, 0.200 mmol, 1.0 equiv). Purification by flash column chromatography (pentane:CH_2Cl_2 = 70:30) afforded the title compound as colorless solid (36.7 mg, 0.109 mmol, 55%, α:β = 82:18).

^{1}H NMR (600 MHz, $CDCl_3$) δ = 8.46 (d, *J* = 1.4 Hz, 0.22H, 2k-β), 8.22 (d, *J* = 1.4 Hz, 0.23H, 2k-β), 7.91 (d, *J* = 4.0 Hz, 1.00H, 2k-α), 7.80 (d, *J* = 4.0 Hz, 1.01H, 2k-α), 6.96 – 5.90 (m, 1.32H, 2k-α + 2k-β), 4.00 – 3.89 (m, 3.85H, 2k-α + 2k-β) ppm.
^{13}C NMR (151 MHz, $CDCl_3$) δ = 161.6, 158.4, 141.7, 140.9, 135.8, 135.7, 134.4, 133.5, 133.4, 130.2, 120.6 (q, *J* = 281.5 Hz), 120.5 (q, *J* = 282.4 Hz), 68.9 – 66.0 (m), 53.0, 52.9 ppm.
^{19}F NMR (471 MHz, $CDCl_3$) δ = −73.2, −73.2 ppm.
HRMS (EI): Calcd. for $C_{10}H_6F_6O_4S$ = 335.98910, found = 335.98917.
IR (neat): 2966, 1755, 1725, 1530, 1434, 1385, 1358, 1288, 1239, 1193, 1094 cm^{-1}.

(1,1,1,3,3,3-hexafluoropropan-2-yl) 5-methyl furan-dicarboxylate (2l)

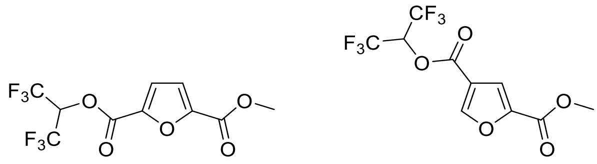

Following the general procedure using methyl furan-2-carboxylate (25.2 mg, 0.200 mmol, 1.0 equiv). Purification by flash column chromatography (pentane:CH_2Cl_2 = 70:30) afforded the title compound as colorless solid (28.1 mg, 0.0878 mmol, 44%, α:β = 88:12).

^{1}H NMR (600 MHz, $CDCl_3$) δ = 8.27 (d, *J* = 0.9 Hz, 0.13H, 2l-β), 7.53 (d, *J* = 0.9 Hz, 0.12H, 2l-β), 7.43 (d, *J* = 3.7 Hz, 1.00H, 2l-α), 7.28 (d, *J* = 3.7 Hz, 0.98H, 2l-α), 6.00 – 5.89 (m, 1.14H, 2l-α + 2l-β), 3.96 (s, 2.87H, 2l-α), 3.94 (s, 0.35H, 2l-β) ppm.
^{13}C NMR (151 MHz, $CDCl_3$) δ = 158.2, 158.1, 154.5, 151.9, 148.9, 146.5, 145.2, 143.3, 120.5 (q, *J* = 283.4 Hz), 116.8, 67.1 (hept, *J* = 35.0 Hz), 52.8, 52.7 ppm.
^{19}F NMR (471 MHz, $CDCl_3$) δ = −73.6, −73.7 ppm.
HRMS (EI): Calcd. for $C_{10}H_6F_6O_5$ = 320.01194, found = 320.01189.
IR (neat): 2976, 1766, 1727, 1580, 1445, 1382, 1359, 1296, 1261, 1230, 1197, 1109 cm^{-1}.

3,4-dimethylbenzoic acid (4m)

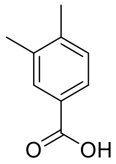

4m

Following the general procedure using o-xylene (21.2 mg, 0.200 mmol, 1.0 equiv). After hydrolysis in MeOH:NaOH (aq., 2M) = 1:1, the aqueous phase was acidified with HCl (1M) until pH = 1 and extracted with CH_2Cl_2 (3 x 20 mL). The organic layer was dried over anhydrous Na_2SO_4, filtered and directly absorbed on silica using a rotary evaporator. Purification by flash column chromatography (pentane:EtOAc:HCOOH = 94.5:5:0.5) afforded the title compound as a white solid (25.6 mg, 0.171 mmol = 85 %). The observed analytical data are in accordance with the ones reported in literature.[10]

^{1}H NMR (600 MHz, $CDCl_3$) δ = 7.91 – 7.88 (m, 1H), 7.85 (dd, *J* = 7.8, 1.9 Hz, 1H), 7.24 – 7.22 (m, 1H), 2.34 (s, 3H), 2.33 (s, 3H) ppm.
^{13}C NMR (151 MHz, $CDCl_3$) δ = 172.5, 143.4, 136.9, 131.2, 129.8, 127.8, 126.9, 20.1, 19.7 ppm.
HRMS (ESI-pos): Calcd. for $C_9H_{11}O_2$= 151,07536, found = 151.07515.

1,1,1,3,3,3-hexafluoropropanyl dimethylbenzoate (2m)

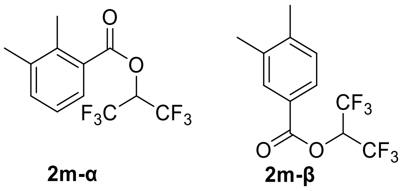

2m-α **2m-β**

Following the general procedure using *o*-xylene (21.2 mg, 0.200 mmol, 1.0 equiv). Purification by flash column chromatography (pentane) afforded the title compound as a colorless solid (44.9 mg,0.150 mmol, 75 %, β:α = 99:1).

^{1}H NMR (600 MHz, $CDCl_3$) δ = 7.88 – 7.85 (m, 1H), 7.85 (dd, *J* = 7.8, 2.0 Hz, 1H), 7.28 – 7.25 (m, 1H), 2.35 (s, 1H), 2.34 (s, 2H) ppm.
^{13}C NMR (151 MHz, $CDCl_3$) δ = 163.6, 144.8, 137.6, 131.5, 130.3, 128.3, 124.5, 120.8 (q, *J* = 283.3 Hz), 67.7 – 66.0 (m), 20.3, 19.8 ppm.
^{19}F NMR (471 MHz, $CDCl_3$) δ = −73.28, -73.16 ppm.
HRMS (EI-neg): Calcd. for $C_{12}H_{10}F_6O_2$= 300.05850, found = 300.05834.
IR (neat): 2982, 1737, 1614, 1388, 1361, 1284, 1262, 1228, 1189, 1173, 1103 cm-1.

2-Fluoromethylbenzoic acid (4n)

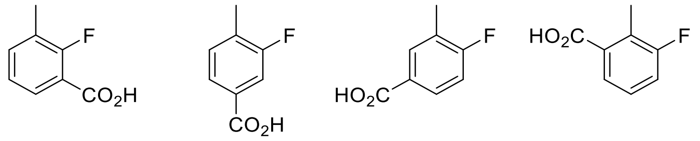

4n-α **4n-β** **4n-β'** **4n-α'**

Following the general procedure using 2-fluorotoluene (22.0 mg, 0.200 mmol, 1.0 equiv). After hydrolysis in MeOH:NaOH (aq., 2M) = 1:1, the aqueous phase was extracted with CH_2Cl_2 (3 x 20 mL) before acidifying with HCl (1M) until pH = 1 and extracting again with CH_2Cl_2 (3 x 20 mL). The organic layer was dried over anhydrous Na_2SO_4, filtered, and directly absorbed on silica using a rotary evaporator. Purification by flash column chromatography (pentane:EtOAc:HCOOH = 94.5:5:0.5) afforded the title compound as slightly yellow solid (16.0 mg, 0.104 mmol, 52%, α:β:β':α' = 26:40:31:2). Assignment was done with the help of literature.[15]

^{1}H NMR (600 MHz, DMSO-d$_6$) δ = 7.90 – 7.86 (m, 0.82H, 4n- β'), 7.82 – 7.79 (m, 0.78H, 4n-β'), 7.78 – 7.74 (m, 0.05H, 4n- α'), 7.68 – 7.67 (m, 1.13H, 4n-β), 7.66 – 7.64 (m, 0.53H, 4n-α), 7.59 – 7.57 (m, 1.00H, 4n-β), 7.53 – 7.48 (m, 0.65H, 4n-α), 7.42 (m, 1.00H, 4n-β), 7.38 – 7.34 (m, 0.06H, 4n- α'), 7.34 – 7.30 (m, 0.06H, 4n- α'), 7.26 – 7.23 (m, 0.77H, 4n- β'), 7.17 (m, 0.66H, 4n-α), 2.40 (d, *J* = 2.4 Hz, 0.15H, 4n- α'), 2.34 – 2.22 (m, 7H, 4n-α + 4n-β + 4n- β') ppm.

^{13}C NMR (151 MHz, DMSO-d$_6$) δ = 166.6, 166.3, 165.3, 163.5 (d, *J* = 249.6 Hz), 160.4 (d, *J* = 243.8 Hz), 159.4 (d, *J* = 255.7 Hz), 135.7 (d, *J* = 5.8 Hz), 133.1 (d, *J* = 6.4 Hz), 130.7 (d, *J* = 7.4 Hz), 130.1, 129.8 (d, *J* = 17.0 Hz), 129.4 (d, *J* = 9.4 Hz), 129.3, 127.0, 125.9 (d, *J* = 17.3 Hz), 125.2 (d, *J* = 3.1 Hz), 124.8 (d, *J* = 17.9 Hz), 123.8 (d, *J* = 4.5 Hz), 122.2, 119.2 (d, *J* = 11.4 Hz), 115.5, 115.3, 115.3, 115.2, 14.4 (d, *J* = 3.3 Hz), 14.2 (d, *J* = 4.7 Hz), 14.0 (d, *J* = 3.3 Hz) ppm.
^{19}F NMR (471 MHz, DMSO-d$_6$) δ = −111.7, −115.7, −116.5, −117.5 ppm.
HRMS (ESI-pos): Calcd. for $C_8H_8O_2F$ = 155.05028, found = 155.05017.
IR (neat): 3681, 2982, 2865, 2844, 1682, 1618, 1581, 1441, 1325, 1303, 1275, 1262 cm^{-1}.

1,2-dichlorobenzoic acid (4o)

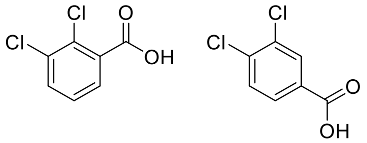

4o-α **4o-β**

Following the general procedure using 1,2-dichlorobenzene (29.4 mg, 0.200 mmol, 1.0 equiv). After hydrolysis in MeOH:NaOH (aq., 2M) = 1:1, the aqueous phase was acidified with HCl (1M) until pH = 1 and extracted with CH_2Cl_2 (3 x 20 mL). The organic layer was dried over anhydrous Na_2SO_4, filtered, and directly absorbed on silica using a rotary evaporator. Purification by flash column chromatography (pentane:EtOAc:HCOOH = 94.5:5:0.5) afforded the title compound as a white solid (19.5 mg, containing 2.9 mg (0.015mmol, 8%) of **4o-α** and 16.6 mg (0.087 mmol, 43%) of **4o-β**, 0.102 mmol, 52% α:β = 15:85).The observed analytical data are in accordance with the ones reported in literature.[16]

^{1}H NMR (600 MHz, DMSO-d$_6$) δ = 8.06 (d, *J* = 1.6 Hz, 1H, 4o-β), 7.88 (dd, *J* = 8.3, 1.8 Hz, 1H, 4o-β), 7.78 (d, *J* = 8.4 Hz, 1.20H, 4o-α + 4o-β), 7.69 (dt, *J* = 7.7, 1.4 Hz, 0.17H, 4o-α), 7.44 (td, *J* = 7.9, 1.2 Hz, 0.17H, 4o-α) ppm.
^{13}C NMR (151 MHz, DMSO-d$_6$) δ = 165.4, 135.8, 132.8, 131.5, 131.5, 131.1, 131.0, 130.1, 129.3, 128.7, 128.4 ppm.
HRMS (ESI-pos): Calcd. for $C_7H_5Cl_2O_2$ = 190,9661, found = 190,9659.

1,3-dimethylbenzoic acid (4p)

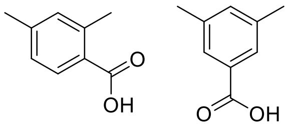

4p-α **4p-β**

Following the general procedure using *m*-xylene (21.2 mg, 0.200 mmol, 1.0 equiv). After hydrolysis in MeOH:NaOH (aq., 2M) = 1:1, the aqueous phase was acidified with HCl (1M) until pH = 1 and extracted with CH_2Cl_2 (3 x 20 mL). The organic layer was dried over anhydrous Na_2SO_4, filtered, and directly absorbed on silica using a rotary evaporator. Purification by flash column chromatography (pentane:EtOAc:HCOOH = 94.5:5:0.5) afforded the title compound as a white solid (21.5 mg, containing 5.2 mg (0.034 mmol, 17%) of **4p-α** and 16.3 mg (0.109 mmol, 55 %) of **4p-β**, 0.143 mmol, 72%, α:β = 24:76). The observed analytical data are in accordance with the ones reported in literature.[17]

^{1}H NMR (600 MHz, CDCl$_3$) δ = 7.98 (d, *J* = 8.4 Hz, 0.23H, 4p-α), 7.74 (dd, *J* = 1.1, 0.6 Hz, 2H, 4p-β), 7.25 (s, 1H, 4p-β), 7.10 – 7.08 (m, 0.46H, 4p-α), 2.63 (s, 0.68H, 4p-α), 2.38 (6H, 4p-β), 2.38 (s, 0.67H, 4p-α) ppm.
^{13}C NMR (151 MHz, CDCl$_3$) δ = 173.2, 172.6 (2-m), 143.8, 141.7, 138.3 (2-m), 135.6 (2-m), 132.8, 131.9, 129.3 (2-m), 128.1 (2-m), 126.8, 125.5, 22.3, 21.6, 21.3 (2-m) ppm.
HRMS (ESI-pos): Calcd. for $C_9H_{11}O_2$ = 190.96611, found = 190.96586.

3,5-di-tert-butylbenzoic acid (4q)

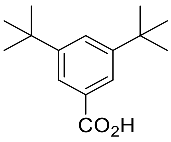

4q

Following the general procedure using 1,3-di-tert-butylbenzene (38.1 mg, 0.200 mmol, 1.0 equiv). After hydrolysis in THF:NaOH (aq., 2M) = 1:1, the aqueous phase was acidified with HCl (1M) until pH = 1 and extracted with CH_2Cl_2 (3 x 20 mL). The organic layer was dried over anhydrous Na_2SO_4, filtered, and directly absorbed on silica using a rotary evaporator. Purification by flash column chromatography (pentane:EtOAc:HCOOH = 94.5:5:0.5) afforded the title compound as a white solid (35.3 mg, 0.151 mmol, 75%). Spectroscopic data match those previously reported in the literature.[18]

^{1}H NMR (600 MHz, CDCl$_3$) δ = δ 7.99 (d, *J* = 1.9 Hz, 1H), 7.69 (t, *J* = 1.9 Hz, 1H), 1.37 (s, 9H) ppm.
^{13}C NMR (151 MHz, CDCl$_3$) δ = 172.9, 151.4, 128.8, 128.2, 124.6, 35.1, 31.5 ppm.
HRMS (EI-pos): Calcd. for $C_{15}H_{22}O_2$ = 234.16198, found = 234.16187.

3-Methyl-1-methoxybenzoic acid (4r)

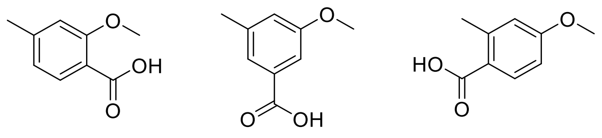

Following the general procedure using 3-methylanisole (24.4mg, 0.200 mmol, 1.0 equiv) and quinoxaline (5.2 mg, 0.040 mmol, 0.2 equiv) as monodentate ligand. After hydrolysis in THF:NaOH (aq., 2M) = 1:1, the aqueous phase was acidified with HCl (aq., 1M) until pH = 1 and extracted with CH_2Cl_2 (3 x 20 mL). The organic layer was dried over anhydrous Na_2SO_4, filtered, and directly absorbed on silica using a rotary evaporator. Purification by flash column chromatography (pentane:EtOAc:HCOOH = 94.5:15:0.5 → 89.5:10:0.5) afforded the title compound as yellow solids (Fraction 1 (8.7 mg, 0.052 mmol of a mixture of **4r-β** and **4r-γ**) and fraction 2 (14.6 mg, 0.088 mmol of **4r-α**), total = 0.140 mmol, 70%, α:β:γ = 63:29:8).

4r-α:

^{1}H NMR (600 MHz, $CDCl_3$) δ = 8.06 (dd, *J* = 8.0, 0.5 Hz, 1H), 6.86 – 6.83 (m, 1H), 6.86 – 6.83 (m, 1H), 4.06 (s, 3H), 2.42 (s, 3H) ppm.
^{13}C NMR (151 MHz, $CDCl_3$) δ = 165.6, 158.1, 146.6, 133.9, 123.3, 115.0, 112.4, 56.7, 22.1 ppm.
HRMS (ESI-pos): Calcd. for $C_9H_{11}O_3$ = 167.07027, found = 167.07022.
IR (neat): 3681, 3007, 2973, 2922, 2864, 2845, 1663, 1610, 1570, 1500, 1458, 1413, 1302, 1239, 1260 cm^{-1}.

4r-β and 4r-γ:

^{1}H NMR (600 MHz, $CDCl_3$) δ = 8.08 (d, *J* = 8.6 Hz, 0.20H, 4r-γ), 7.55 – 7.54 (m, 1.00H, 4r-β), 7.47 – 7.40 (m, 1.02H, 4r-β), 6.98 – 6.97 (m, 0.99H, 4r-β), 6.81 – 6.74 (m, 0.44H, 4r-γ), 3.87 – 3.82 (m, 3.57H, 4r-β + 4r-γ), 2.65 (s, 0.59H, 4r-γ), 2.39 (s, 3.01H, 4r-β) ppm.
^{13}C NMR (151 MHz, $CDCl_3$) δ = 172.4, 171.9, 163.3, 159.7, 144.5, 139.9, 134.3, 130.4, 123.7, 121.3, 120.5, 117.3, 111.6, 111.2, 55.6, 55.5, 22.8, 21.5 ppm.
HRMS (ESI-pos): Calcd. for $C_9H_{11}O_3$ = 167.07027, found = 167.07030.
IR (neat): 3681, 2924, 2865, 2843, 2629, 2540, 1686, 1609, 1595, 1569, 1465, 1410, 1331, 1299, 1247 cm^{-1}.

1,1,1,3,3,3-hexafluoropropan-2-yl 3-(tert-butyldimethylsilyl)-5-((tert-butyldimethylsilyl)oxy)benzoate (2s)

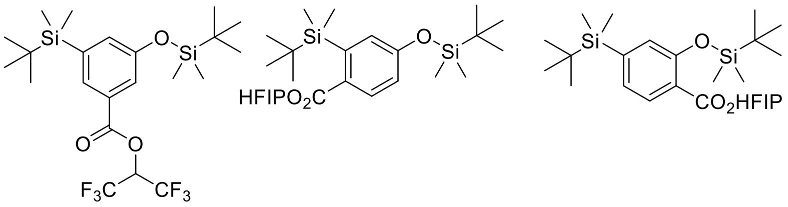

2s-β **2s-others**

Following the general procedure using *tert*-butyl(3-((tert-butyldimethylsilyl)oxy)phenyl)dimethylsilane (64.5 mg, 0.200 mmol, 1.0 equiv). Purification by flash column chromatography (pentane) afforded the title compound as colorless oil (80.2 mg, 0.155 mol, 78%, β:others ≥ 95:5).

^{1}H NMR (600 MHz, CDCl$_3$) δ =7.82-7.81 (m, 1H), 7.52-7.51 (m, 1H), 7.28 – 7.24 (m, 1H), 5.99 (hept, J = 6.1 Hz, 1H), 1.00 (s, 9H), 0.89 (s, 9H), 0.30 (s, 6H), 0.23 (s, 6H) ppm.
^{13}C NMR (151 MHz, CDCl$_3$) δ = 163.5, 141.2, 132.5, 129.2, 127.4, 121.9, 120.8 (q, J = 281.7 Hz), 67.1 (hept, 34.4 Hz), 26.5, 25.8, 18.4, 17.0, -4.3, -6.1 ppm.
^{19}F NMR (471 MHz, CDCl$_3$) δ = −73.1, −73.3, −73.6 ppm.
HRMS (EI): Calcd. for $C_{22}H_{34}F_6O_3Si_2$ = 516.19507, found = 516.19509.

1,1,1,3,3,3-hexafluoropropan-2-yl (1-methoxy-3-oxopropyl)-3-methylbenzoate (2t)

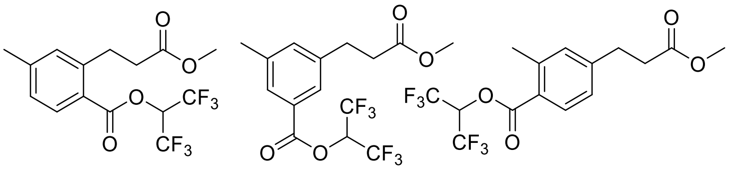

2t-α **2t-β** **2t-γ**

Following the general procedure using methyl 3-(3-methylphenyl)propanoate (35.6 mg, 0.200 mmol, 1.0 equiv). Purification by flash column chromatography (pentane:EtOAc = 90:10) afforded the title compound as colorless oil (45.9 mg, containing **ML1** (1.3 mg, 5 mol%) and **2t** (44.6 mg, 0.120 mmol 95 mol%), 0.120 mmol (**2t**), 60%, β:others = 88:12).

^{1}H NMR (500 MHz, CDCl$_3$) δ = 8.01 – 7.93 (m, 0.15H, 2t- α + 2t- γ), 7.76 (s, 1H, 2t- β), 7.74 (s, 1H, 2t- β), 7.33 (dd, J = 1.9, 1.1 Hz, 1H, 2t- β), 7.18 – 7.13 (m, 0.21H, 2t- α + 2t- γ), 6.01 (m, 1.14H), 3.68 (s, 3.41H, 2t- α + 2t- β + 2t- γ), 2.99 (t, J = 7.7 Hz, 2.28H), 2.66 (t, J = 7.7 Hz, 2.30H), 2.61 (s, 0.28H), 2.41 (s, 3.17H) ppm.
^{13}C NMR (126 MHz, CDCl$_3$) δ = 173.0, 163.5, 147.3, 142.6, 141.6, 139.2, 135.9, 132.4, 132.1, 129.2, 127.1, 126.3, 119.6 (q, J = 283.8 Hz), 67.0 (hept, J = 34.9 Hz), 51.8, 35.5, 35.1, 30.9, 30.6, 22.1, 21.3 ppm.
^{19}F NMR (471 MHz, CDCl$_3$) δ = –73.6, –73.6, –73.7 ppm.
HRMS (EI): Calcd. for $C_{15}H_{14}F_6O_4$= 372.07963, found = 372.07962.
IR (neat): 2958, 1740, 1609, 1454, 1438, 1385, 1357, 1282, 1265, 1224, 1202, 1182, 1105 cm^{-1}.

(2-methoxyethyl)-3-methylbenzoic acid (4u)

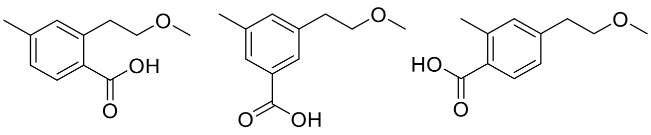

4u-α **4u-β** **4u-γ**

Following the general procedure using 1-(2-methoxyethyl)-3-methylbenzene (30.0 mg, 0.200 mmol, 1.0 equiv). After hydrolysis in THF:NaOH (aq., 2M) = 1:1, the aqueous phase was extracted with CH_2Cl_2 (3 x 20 mL) before acidifying with HCl (1M) until pH = 1 and extracting again with CH_2Cl_2 (3 x 20 mL). The organic layer was dried over anhydrous Na_2SO_4, filtered, and directly absorbed on silica using a rotary evaporator. Purification by flash column chromatography (pentane:EtOAc:HCOOH = 94.5:5:0.5) afforded the title compound as colorless solid (23.1 mg, 0.119 mmol, 60%, α:β:γ = 7:83:10).

^{1}H NMR (600 MHz, $CDCl_3$) δ = 8.01 (d, *J* = 7.9 Hz, 0.12H, 4u-γ), 7.91 (d, *J* = 7.8 Hz, 0.08H, 4u-α), 7.81 – 7.76 (m, 2.00H, 4u-β), 7.29 – 7.28 (m, 1.00H, 4u-β), 7.17 – 7.10 (m, 0.38H, 4u-α + 4u-γ), 3.68 (t, *J* = 6.6 Hz, 0.14H), 3.64 (t, *J* = 7.0 Hz, 2.23H), 3.40 – 3.35 (m, 3.54H), 3.27 – 3.20 (m, 0.14H), 2.93 – 2.89 (m, 2.22H), 2.64 (s, 0.34H), 2.40 (s, 3.20H) ppm.
^{13}C NMR (151 MHz, $CDCl_3$) δ = 172.8, 172.1, 144.7, 143.5, 141.7, 139.5, 138.5, 135.4, 132.7, 132.5, 132.0, 131.9, 129.5, 128.9, 127.8, 127.6, 126.5, 73.7, 73.4, 73.1, 58.8, 36.2, 36.0, 34.9, 22.3, 21.6, 21.4 ppm.
HRMS (EI): Calcd. for $C_{11}H_{14}O_3$ = 194.09429, found = 194.09433.
IR(neat): 3681, 2982, 2923, 2867, 1718, 1686, 1607, 1455, 1412, 1383, 1299, 1261, 1276 cm^{-1}.

3-Fluoro-1-methylbenzoic acid (4v)

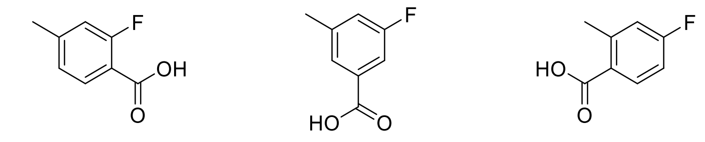

4v-α **4v-β** **4v-γ**

Following the general procedure using 3-fluorotoluene (22.0 mg, 0.200 mmol, 1.0 equiv). After hydrolysis in THF:NaOH (aq., 2M) = 1:1, the aqueous phase was extracted with CH_2Cl_2 (3 x 20 mL) before acidifying with HCl (1M) until pH = 1 and extracting again with CH_2Cl_2 (3 x 20 mL). The organic layer was dried over anhydrous Na_2SO_4, filtered and directly absorbed on silica using a rotary evaporator. Purification by flash column chromatography (pentane:EtOAc:HCOOH = 94.5:5:0.5) afforded the title compound as colorless solid (16.8 mg, 0.109 mmol, 54%, α:β:γ = 48:48:4).

^{1}H NMR (600 MHz, CDCl$_3$) δ = 8.12 – 8.10 (m, 0.09H, 4v-γ), 7.94 – 7.91 (m, 1H, 4v-α), 7.73 – 7.72 (m, 1H, 4v-β), 7.63 – 7.56 (m, 1H, 4v-β), 7.15 – 7.12 (m, 1H, 4v-β), 7.05 – 7.02 (m, 1H, 4v-α), 7.01 – 6.94 (m, 1H, 4v-α + 4v-γ), 2.66 (s, 0.17H, 4v-γ), 2.46 – 2.39 (m, 6H, 4v-β + 4v-α) ppm.
^{13}C NMR (151 MHz, CDCl$_3$) δ = 171.1, 169.4, 162.8 (d, *J* = 261.2 Hz), 162.7 (d, *J* = 246.9 Hz), 147.5 (d, *J* = 9.2 Hz), 141.0 (d, *J* = 7.5 Hz), 134.6 (d, *J* = 10.1 Hz), 132.8, 131.3, 126.8, 125.2, 121.7, 118.9 (d, *J* = 21.3 Hz), 117.7 (d, *J* = 22.1 Hz), 114.3 (d, *J* = 23.0 Hz), 113.2 (d, *J* = 21.8 Hz), 22.5, 21.8, 21.4 ppm.
^{19}F NMR (471 MHz, CDCl$_3$) δ = −106.0, −109.1, −113.2 ppm.
HRMS (ESI-pos): Calcd. for $C_8H_8O_2F$ = 155.05028, found = 155.05013.
IR (neat): 2924, 2659, 1688, 1626, 1590, 1418, 1318, 1302, 1267, 1239 cm^{-1}.

3-Methyl-5-(trifluoromethyl)benzoic acid (4w)

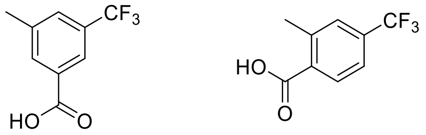

4w-β **4w-γ**

Following the general procedure using 1-methyl-3-trifluoromethyl-benzene (32.0 mg, 0.200 mmol, 1.0 equiv). After hydrolysis in THF:NaOH (aq., 2M) = 1:1, the aqueous phase was extracted with CH_2Cl_2 (3 x 20 mL) before acidifying with HCl (1M) until pH = 1 and extracting again with CH_2Cl_2 (3 x 20 mL). The organic layer was dried over anhydrous Na_2SO_4, filtered, and directly absorbed on silica using a rotary evaporator. Purification by flash column chromatography (pentane:EtOAc:HCOOH = 94.5:5:0.5) afforded the title compound as a slightly yellow solid (14.1 mg, 0.069 mmol, 35%, β: γ = 93:7). Assignment was done with the help of literature.[19]

^{1}H NMR (600 MHz, CDCl$_3$) δ = 8.19 – 8.18 (m, 1H, α), 8.16 (d, *J* = 8.6 Hz, 0.07H, β), 8.12 – 8.10 (m, 1H, α), 7.56 – 7.54 (m, 0.14H. β), 2.72 (s, 0.18H, β), 2.50 (s, 3H, α) ppm.
^{13}C NMR (151 MHz, CDCl$_3$) δ = 170.7, 139.8, 134.1, 131.1 (q, *J* = 32.8 Hz), 131.2 – 131.0 (m), 130.1, 128.8, 124.5 – 124.5 (m), 123.8 (q, *J* = 272.4 Hz), 22.2, 21.4 ppm.
^{19}F NMR (471 MHz, CDCl$_3$) δ = -62.9 (m) ppm.
HRMS (EI): Calcd. for $C_9H_7O_2F_3$ = 204.03981, found = 204.03984.
IR (neat): 2925, 2600, 1694, 1610, 1424, 1385, 1349, 1283, 1268, 1210, 1168, 1122 cm^{-1}.

4-Chloro-1-methylbenzoic acid (4x)

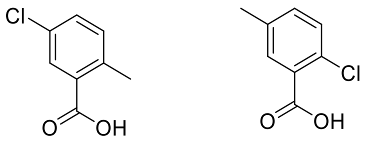

4x-α **4x-β**

Following the general procedure using 4-chlorotoluene (25.3 mg, 0.200 mmol, 1.0 equiv). After hydrolysis in MeOH:NaOH (aq., 2M) = 1:1, the aqueous phase was extracted with CH_2Cl_2 (3 x 20 mL) before acidifying with HCl (1M) until pH = 1 and extracting again with CH_2Cl_2 (3 x 20 mL). The organic layer was dried over anhydrous Na_2SO_4, filtered, and directly absorbed on silica using a rotary evaporator. Purification by flash column chromatography (pentane:EtOAc:HCOOH = 94.5:5:0.5) afforded the title compound as colorless solid (10.1 mg, 0.059 mmol, 30%, α:β =9:91).

^{1}H NMR (600 MHz, CDCl$_3$) δ = 8.06 – 8.02 (m, 0.8H, 4x-α), 7.83 – 7.82 (m, 0.98H, 4x-β), 7.44 – 7.40 (m, 0.08H, 4x-α), 7.38 – 7.36 (m, 0.99H, 4x-β), 7.29 – 7.27 (m, 1.00H, 4x-β), 7.23 – 7.20 (m, 0.09H, 4x-α), 2.62 (s, 0.17H, 4x-α), 2.38 (s, 2.96H, 4x-β) ppm.
^{13}C NMR (151 MHz, CDCl$_3$) δ = 170.5, 136.9, 134.5, 133.4, 133.0, 131.8, 131.7, 131.5, 131.4, 130.8, 129.1, 128.1, 21.7, 20.8 ppm.
HRMS (ESI-pos): Calcd. for $C_8H_8O_2{}^{35}Cl$ = 171.02073, found = 171.02069.
IR (neat): 3006, 2987, 1699, 1677, 1301, 1275, 1260 cm^{-1}.

1,3-diisopropyl-2-methoxybenzoic acid (4y)

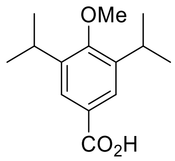

4y

Following the general procedure using 1,3-diisopropyl-2-methoxybenzene (38.5 mg, 0.200 mmol, 1.0 equiv). After hydrolysis in MeOH:NaOH (aq., 2M) = 1:1, the aqueous phase was extracted with CH_2Cl_2 (3 x 20 mL) before acidifying with HCl (1M) until pH = 1 and extracting again with CH_2Cl_2 (3 x 20 mL). The organic layer was dried over anhydrous Na_2SO_4, filtered, and directly absorbed on silica using a rotary evaporator. Purification by flash column chromatography (pentane:EtOAc:HCOOH = 92:7.5:0.5) afforded the title compound as colorless solid (23.9 mg, 0.101 mmol, 51%).

^{1}H NMR (600 MHz, CDCl$_3$) δ = 7.89 (s, 2H), 3.79 (s, 3H), 3.34 (h, *J* = 6.9 Hz, 2H), 1.27 (d, *J* = 6.9 Hz, 12H) ppm.
^{13}C NMR (151 MHz, CDCl$_3$) δ = 172.1, 159.7, 142.4, 126.9, 125.5, 62.4, 26.8, 24.0 ppm.
HRMS (ESI-neg): Calcd. for $C_{14}H_{19}O_3$ = 235.13397, found = 235.13376.
IR (neat): 2963, 2868, 1677, 1603, 1584, 1460, 1438, 1414, 1291, 1260, 1200, 1168 cm^{-1}.

3,4-dimethyl-5-(((1,3,3-trimethylbicyclo[2.2.1]heptan-2-yl)oxy)methyl)benzoic acid (4z)

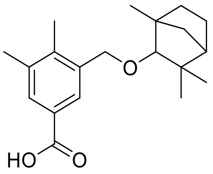

4z-β

Following the general procedure using 2-((2,3-dimethylbenzyl)oxy)-1,3,3-trimethylbicyclo[2.2.1]heptane (54.5 mg, 0.200 mmol, 1.0 equiv). After hydrolysis in THF:NaOH (aq., 2M) = 1:1, the aqueous phase was extracted with CH_2Cl_2 (3 x 20 mL) before acidifying with HCl (1M) until pH = 1 and extracting again with CH_2Cl_2 (3 x 20 mL). The organic layer was dried over anhydrous Na_2SO_4, filtered, and directly absorbed on silica using a rotary evaporator. Purification by flash column chromatography (pentane:EtOAc:HCOOH = 94.5:5:0.5) afforded the title compound as colorless solid (23.3 mg, 0.0736 mmol, 37%, β:others ≥ 96:4).

^{1}H NMR (600 MHz, CDCl$_3$) δ = 7.97 – 7.96 (m, 1H), 7.85 (m, 1H), 4.58 (d, *J* = 11.8 Hz, 1H), 4.49 (d, *J* = 11.8 Hz, 1H), 3.09 – 3.02 (m, 1H), 2.35 (s, 3H), 2.30 (s, 3H), 1.82 – 1.77 (m, 1H), 1.74 – 1.67 (m, 1H), 1.65 – 1.63 (m, 1H), 1.50 – 1.45 (m, 1H), 1.42 – 1.36 (m, 1H), 1.13 – 0.94 (m, 11H) ppm.
^{13}C NMR (151 MHz, CDCl$_3$) δ = 172.4, 142.2, 137.8, 137.4, 130.9, 128.4, 126.2, 93.2, 72.4, 49.4, 49.0, 41.7, 39.8, 31.9, 26.3, 26.2, 21.0, 20.4, 20.3, 15.6 ppm.
HRMS (ESI-pos): Calcd. for $C_{20}H_{29}O_3$ = 317.21112, found = 317.21097.
IR(neat): 3707, 2951, 2868, 1687, 1455, 1421, 1304, 1276, 1261 cm^{-1}.

2-Fluoro-1,3-dimethylbenzoic acid (4aa)

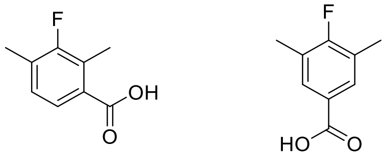

4aa-α **4aa-β**

Following the general procedure using 2-fluoro-1,3-dimethylbenzene (24.8 mg, 0.200 mmol, 1.0 equiv). After hydrolysis in THF:NaOH (aq., 2M) = 1:1, the aqueous phase was extracted with CH_2Cl_2 (3 x 20 mL) before acidifying with HCl (1M) until pH = 1 and extracting again with CH_2Cl_2 (3 x 20 mL). The organic layer was dried over anhydrous Na_2SO_4, filtered, and directly absorbed on silica using a rotary evaporator. Purification by flash column chromatography (pentane:EtOAc:HCOOH = 94.5:5:0.5) afforded the title compound as colorless solid (20.1 mg, 0.120 mmol, 60%, β:α = 83:17).

^{1}H NMR (500 MHz, $CDCl_3$) δ = 7.80 (d, *J* = 7.0, 2.00H, 4aa-β), 7.75 (d, *J* = 9.3 Hz, 0.26H, 4aa-α), 7.09 (t, *J* = 7.7 Hz, 0.21H, 4aa-α), 2.55 (d, *J* = 2.5 Hz, 0.57H, 4aa-α), 2.33 (d, *J* = 2.3 Hz, 0.65H, 4aa-α), 2.31 (d, *J* = 1.8, 5.88H, 4aa-β) ppm.
^{13}C NMR (151 MHz, $CDCl_3$) δ = 172.4 (d, *J* = 13.9 Hz), 171.6 (d, *J* = 12.0 Hz), 163.5 (d, *J* = 252.7 Hz), 159.9 (d, *J* = 242.6 Hz), 131.4 (d, *J* = 6.5 Hz), 130.6, 130.3 (d, *J* = 19.5 Hz), 128.0 (d, *J* = 5.4 Hz), 127.8, 126.7 (d, *J* = 4.1 Hz), 124.9 (d, *J* = 18.8 Hz), 124.4, 15.2 (d, *J* = 4.3 Hz), 14.6 (d, *J* = 4.1 Hz), 12.1 (d, *J* = 7.1 Hz) ppm.
^{19}F NMR (471 MHz, $CDCl_3$) δ = −112.9, −119.7 ppm.
HRMS (EI): Calcd. for $C_9H_9FO_2$ = 168.05866, found = 168.05864.
IR(neat): 2925, 2632, 2561, 1682, 1618, 1602, 1423, 1318, 1261, 1198 cm^{-1}.

2-Bromo-1,3-dimethylbenzoic acid (4ab)

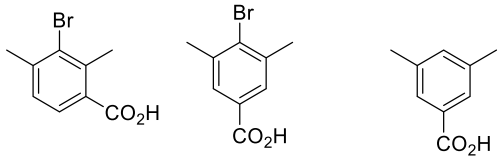

4ab-α **4ab-β** **4ab-β(-Br)**

Following the general procedure using 2-bromo-*m*-xylene (37.0 mg, 0.200 mmol, 1.0 equiv). After hydrolysis in THF:NaOH (aq., 2M) = 1:1, the aqueous phase was extracted with CH_2Cl_2 (3 x 20 mL) before acidifying with HCl (1M) until pH = 1 and extracting again with CH_2Cl_2 (3 x 20 mL). The organic layer was dried over anhydrous Na_2SO_4, filtered, and directly absorbed on silica using a rotary evaporator. Purification by flash column chromatography (pentane:EtOAc:HCOOH = 94.5:5:0.5) afforded the title compound as colorless solid (26.4 mg, containing 1.2 mg (0.008 mmol, 4%) of **4ab-β(-Br)** and 25.2 mg (0.110 mmol, 55%) of **4ab-α** and **4ab-β** (α:β = 6:94), **α:β:β(-Br)** = 6:87:7).

^{1}H NMR (600 MHz, DMSO-d$_6$) δ = 7.72 (m, 2.00H, 4ab-β), 7.63 (d, *J* = 7.9 Hz, 0.07H, 4ab-α), 7.57 – 7.53 (m, 0.17H, 4ab-β(-Br)), 7.28 (d, *J* = 7.9 Hz, 0.07H, 4ab-α), 7.25 – 7.23 (m, 0.08H, 4ab-β(-Br)), 2.58 (s, 0.21H, 4ab-α), 2.41 (m, 6.20H, 4ab-α + 4ab-β), 2.31 (m, 0.51H, 4ab-β(-Br)) ppm.
^{13}C NMR (151 MHz, DMSO-d$_6$) δ = 166.9, 141.4, 138.3, 137.7, 134.2, 131.9, 129.5, 129.0, 128.4, 128.1, 127.0, 24.2, 23.4, 21.0, 20.7 ppm.
HRMS (ESI-pos): Calcd. for $C_9H_{10}O_2{}^{79}Br$ = 228.98587, found = 228.98576.
IR(neat): 3681, 2982, 1687, 1429, 1313, 1276, 1260 cm^{-1}.

5-(1,1,1,3,3,3-hexafluoropropan-2-yl) 3-methyl 2-methyl-4'-(trifluoromethoxy)-[1,1'-biphenyl]-3,5-dicarboxylate (2ac)

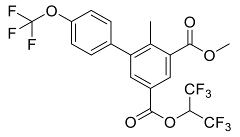

Following the general procedure and using methyl 2-methyl-4'-(trifluoromethoxy)-[1,1'-biphenyl]-3-carboxylate (62.0 mg, 0.200 mmol, 1.0 equiv.) as substrate the target compound **2ac** was obtained as colorless solid (48.4 mg, 0.096 mmol, 48%). The product was purified via silica gel column chromatography using pentane:Et_2O = 99:1 as the eluent.

^{1}H NMR (500 MHz, CDCl$_3$) δ 8.53 (d, *J* = 2.0 Hz, 1H), 8.03 (d, *J* = 2.0 Hz, 1H), 7.32 (s, 4H), 6.32 – 5.73 (m, 1H), 3.98 (s, 3H), 2.49 (s, 3H) ppm.
^{13}C NMR (126 MHz, CDCl$_3$) δ 167.4, 162.7, 149.1, 144.6, 143.6, 138.6, 134.2, 132.7, 131.5, 130.8, 124.4, 121.7, 120.6(3) (q, *J* = 257.3 Hz), 120.6(2) (q, *J* = 283.5 Hz), 67.2 (hept, *J* = 34.8 Hz), 52.7, 19.2 ppm.
^{19}F NMR (471 MHz, CDCl$_3$) δ −58.3, −73.5 ppm.
HRMS (ESI-pos): Calcd. for $C_{20}H_{14}F_9O_5$ = 505.06920, found = 505.06850.
IR(neat): 2969, 1757, 1732, 1509, 1385, 1333, 1358 cm^{-1}.

4.2. Additional substrates

1-(diisopropylcarbamoyl)-2-methylbenzoic acid (4ad)

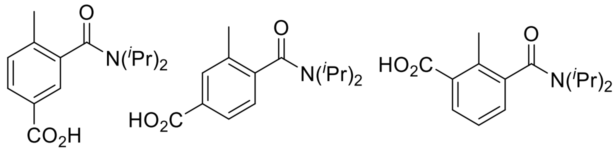

4ad-β **4ad-β'** **4ad-α'**

Following the general procedure at 85 °C and with HFIP (1.2 mL), using *N,N*-diisopropyl-2-methylbenzamide (43.9 mg, 0.200 mmol, 1.0 equiv). After hydrolysis in THF:NaOH (aq., 2M) = 1:1, the aqueous phase was extracted with CH_2Cl_2 (3 x 20 mL) before acidifying with HCl (1M) until pH = 1 and extracting again with CH_2Cl_2 (3 x 20 mL). The organic layer was dried over anhydrous Na_2SO_4, filtered, and directly absorbed on silica using a rotary evaporator. Purification by flash column chromatography (pentane:EtOAc:HCOOH = 79.5:20:0.5) afforded the title compound as colorless, waxy solid (14.7 mg, containing HCOOH (0.2 mg), EtOAc (0.3 mg) and the title compound (14.2 mg, 0.054 mmol, 27%, β: β': α' = 45:49:6).

^{1}H NMR (600 MHz, DMSO-d$_6$) δ = 7.84 – 7.80 (m, 2.06H, 4ad- β' + 4ad-β), 7.78 (ddd, *J* = 7.8, 1.7, 0.7 Hz, 1.10H, 4ad- β'), 7.75 – 7.73 (m, 0.11H, 4ad- α'), 7.60 – 7.56 (m, 1.00H, 4ad-β), 7.38 (dt, *J* = 8.0, 0.6 Hz, 1.00H, 4ad-β), 7.33 – 7.31 (m, 0.12H, 4ad- α'), 7.27 – 7.25 (m, 0.14H, 4ad- α'), 7.21 (d, *J* = 7.8 Hz, 1.09H, 4ad- β'), 3.58 (m), 3.45 (m), 2.38 (s, 0.39H), 2.30 – 2.24 (m, 6H), 1.49 – 1.44 (m, 13H), 1.09 – 1.03 (m, 5H), 1.08 – 1.02 (m, 9H) ppm.
^{13}C NMR (151 MHz, DMSO-d$_6$) δ = 168.7, 168.5, 167.0, 166.9, 142.5, 138.7, 138.5, 133.5, 131.1, 130.7, 130.3, 129.5, 128.9, 128.6, 127.7, 127.0, 126.0, 125.1, 124.8, 50.6, 50.6, 44.8, 44.8, 20.5, 20.4, 20.3, 20.2, 20.2, 20.0, 18.5, 18.2 ppm.
HRMS (ESI-neg): Calcd. for $C_{15}H_{20}O_3N$ = 262.14487, found = 262.14503.
IR (neat): 3681, 2972, 2936, 2873, 2844, 1712, 1629, 1591, 1445, 1371, 1340, 1276, 1259 cm^{-1}.

3-Bromo-1-methylbenzoic acid (4ae)

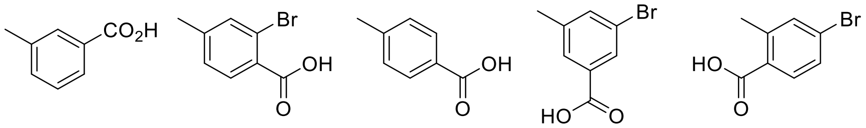

4ae-ipso **4ae-α** **4ae-α(-Br)** **4ae-β** **4ae-γ**

Following the general procedure using 3-bromotoluene (34.2 mg, 0.200 mmol, 1.0 equiv). After hydrolysis in THF:NaOH (aq., 2M) = 1:1, the aqueous phase was extracted with CH_2Cl_2 (3 x 20 mL) before acidifying with HCl (1M) until pH = 1 and extracting again with CH_2Cl_2 (3 x 20 mL). The organic layer was dried over anhydrous Na_2SO_4, filtered, and directly absorbed on silica using a rotary evaporator. Purification by flash column chromatography (pentane:EtOAc:HCOOH = 94.5:5:0.5) afforded the title compound as slightly yellow solid (26.0 mg, containing 2.1 mg (0.016 mmol, 8%) of **4ae-ipso** + **4ae-α(-Br)** and 23.9 mg (0.111 mmol, 56%) of **4ae-α** + **4ae-β** + **4ae-γ** (α:β:γ = 34:61:5), **ipso:α:α(-Br):β:γ** = 9:30:4:53:4). Spectral data of reported isomers are in accordance to the literature.[20–22]

^{1}H NMR (600 MHz, CDCl$_3$) δ = 8.05 (m, 1.00H, 4ae-β), 8.02 – 7.99 (m, 0.16H, 4ae-α(-Br)), 7.96 – 7.90 (m, 0.82H, 4ae-ipso + 4ae-α + 4ae-γ), 7.86 – 7.85 (m, 0.99H, 4ae-β), 7.58 – 7.57

(m, 0.99H, 4ae-β), 7.55 – 7.54 (m, 0.56H, 4ae-α), 7.46 – 7.45 (m, 0.07H, 4ae-γ), 7.44 – 7.41 (m, 0.19H, 4ae-ipso + 4ae-γ), 7.38 – 7.36 (dd, *J* = 7.6 Hz, *J* = 7.6 Hz, 0.16H, 4ae-ipso), 7.29 – 7.27 (m, 0.17H, 4ae-α(-Br)), 7.21 – 7.19f (m, 0.60H, 4ae-α), 2.63 (s, 0.13H, 4ae-γ), 2.44 (s, 0.20H, 4ae-α(-Br)), 2.43 (s, 0.31H, 4ae-ipso), 2.41 (q, *J* = 0.7 Hz, 2.89H, 4ae-β), 2.39 (q, *J* = 0.6 Hz, 1.61H, aew-α) ppm.
^{13}C NMR (151 MHz, CDCl$_3$) δ = 170.9, 144.9, 140.6, 138.5, 137.5, 135.6, 135.0, 134.8, 133.2, 132.7, 131.1, 130.9, 130.8, 130.4, 129.6, 129.4, 128.6, 128.2, 127.5, 127.4, 122.8, 122.5, 22.1, 21.9, 21.4, 21.3, 21.2 ppm.
HRMS (ESI-pos): Calcd. for $C_8H_8O_2{}^{79}Br$ = 214.97022, found = 214.97018.
IR (neat): 2981, 2646, 2560, 1682, 1601, 1573, 1446, 1408, 1293, 1277 cm^{-1}.

Ethylbenzoic acid from a reaction in 2,2,2-trifluoroethanol (4a)

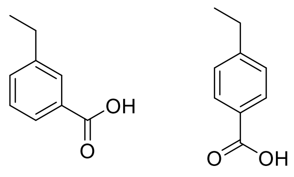

4a-m **4a-p**

Following the general procedure using ethylbenzene (**1a**) (21.2 mg, 0.200 mmol) and 2,2,2-trifluoroethanol (2.0 mL) instead of HFIP. After hydrolysis in THF:NaOH (aq., 2M) = 1:1, the aqueous phase was extracted with CH_2Cl_2 (3 x 20 mL) before acidifying with HCl (1M) until pH = 1 and extracting again with CH_2Cl_2 (3 x 20 mL). The organic layer was dried over anhydrous Na_2SO_4, filtered, and directly absorbed on silica using a rotary evaporator. Purification by flash column chromatography (pentane:EtOAc:HCOOH = 94.5:5:0.5) afforded the title compound as colorless solid (14.6 mg, 0.097 mmol, 49%, m:p = 65:35). Spectral data of reported isomers are in accordance to the literature.[23,24]

^{1}H NMR (500 MHz, CDCl$_3$) δ = 8.07 – 8.00 (m, 1.06H, 4af-p), 7.99 – 7.91 (m, 1.92H, 4af-m), 7.52 – 7.42 (m, 1.04H, 4af-m), 7.42 – 7.36 (m, 1.00H, 4af-m), 7.33 – 7.29 (m, 1.07H, 4af-p), 2.77 – 2.68 (m, 2.95H), 1.34 – 1.22 (m, 5.64H) ppm.
^{13}C NMR (126 MHz, CDCl$_3$) δ = 172.2, 172.1, 150.9, 144.8, 133.6, 130.5, 129.7, 129.5, 128.6, 128.2, 127.8, 29.2, 28.8, 15.6, 15.3 ppm.
HRMS (EI): Calcd. for $C_9H_{10}O_2$ = 150.06808, found = 150.06811.

5. Scalability and Follow-up Transformations

5.1 Scale-up Reactions

General procedure for scale-up reactions: An oven-dried 150 mL Schlenk-flask was charged with a stirring bar, $Pd(MeCN)_2Cl_2$ (31.2 mg, 0.120 mmol, 10 mol%), *N*-(2-((3 -trifluoromethyl)thio)ethyl)acetamide (**BL1**) (126.0 mg, 0.480 mmol, 40 mol%), 3-Trifluoromethylquinoline (**ML1**) (47.4 mg, 0.240 mmol, 20 mol%), $AgNO_3$ (814.8 mg, 4.80 mmol, 4.0 equiv), $Mo(CO)_6$ (104.4 mg, 0.396 mmol, 0.33 equiv), and HFIP (12 mL). The flask was tightly sealed and the mixture was stirred at room temperature for approximately 10 min. The respective substrate (1.20 mmol) was added, the flask was tightly sealed, placed in a pre-heated oil-bath at 80 °C, and the mixture was stirred with 700 rpm for 24 h. After cooling to room-temperature the mixture was filtered through a pad of celite and eluted with CH_2Cl_2 (70 mL). The crude mixture was directly adsorbed on silica and subjected to flash-column chromatography.

1,1,1,3,3,3-hexafluoropropanyl dimethylbenzoate (2m)

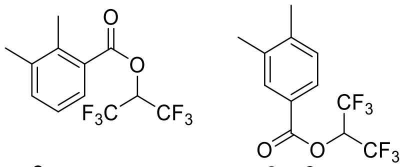

2m-α **2m-β**

Following the general procedure using *o*-xylene (127.4 mg, 1.200 mmol). Purification by flash column chromatography (pentane) afforded the title compound as a colorless solid in two fractions (Fraction 1 (40.8 mg, **2m-α** and **2m-β**) and Fraction 2 (244.0 mg, **2m-β** containing a trace amount of **2m-α**), amounting to a total of 284.8 mg, 0.948 mmol, 79%, α:β = 5:95).

Fraction 1:

^{1}H NMR (500 MHz, $CDCl_3$) δ = 7.89 – 7.82 (m, 2H, 2m-β), 7.77 (d, *J* = 7.2 Hz, 0.37H, 2m-α), 7.40 (d, *J* = 7.5 Hz, 0.38H, 2m-α), 7.31 – 7.24 (m, 1H, 2m-β), 7.21 (t, *J* = 7.7 Hz, 0.43H, 2m-α), 6.05 – 5.98 (m, 1.56H, 2m-α + 2m-β), 2.50 (s, 1.15H, 2m-α), 2.36 (s, 1.23H, 2m-α), 2.35 (s, 3H, 2m-β), 2.34 (s, 3H, 2m-β) ppm.
^{13}C NMR (126 MHz, $CDCl_3$) δ = 164.5, 163.6, 144.8, 139.7, 138.7, 137.6, 135.2, 131.5, 130.3, 128.8, 128.3, 127.1, 125.7, 124.5, 120.9 (q, *J* = 282.0 Hz), 66.9 (m), 20.8, 20.4, 19.8, 16.8 ppm.
^{19}F NMR (471 MHz, $CDCl_3$) δ = −73.1, −73.2 ppm.

Fraction 2:

^{1}H NMR (500 MHz, $CDCl_3$) δ = 7.93 – 7.81 (m, 2H), 7.30 – 7.23 (m, 1H), 6.00 (h, *J* = 6.1 Hz, 1H), 2.35 (s, 3H), 2.34 (s, 3H) ppm.
^{13}C NMR (126 MHz, $CDCl_3$) δ = 163.6, 144.8, 137.6, 131.5, 130.3, 128.3, 124.5, 120.7 (q, *J* = 283.4 Hz), 66.9 (hept, *J* = 34.3 Hz), 20.4, 19.8.
^{19}F NMR (471 MHz, $CDCl_3$) δ = −73.1, -73.2 ppm.

1,1,1,3,3,3-hexafluoropropan-2-yl 3-(tert-butyldimethylsilyl)-5-((tert-butyldimethylsilyl)oxy)benzoate (2s)

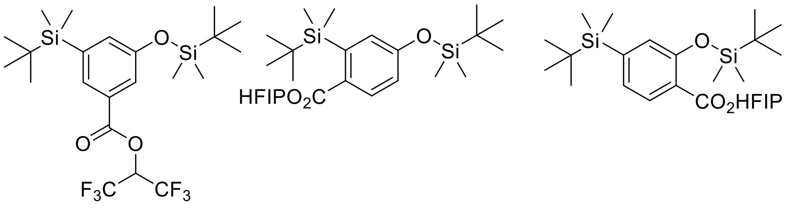

2s-β **2s-others**

Following the general procedure using *tert*-butyl(3-((tert-butyldimethylsilyl)oxy)phenyl)dimethylsilane (392.0 mg, 1.21 mmol). Purification by flash column chromatography (pentane) afforded the title compound as colorless oil which solidified after several days (471.9 mg, 0.913 mmol, 75%, β:others ≥ 91:9).

^{1}H NMR (500 MHz, CDCl$_3$) δ = 7.81 (dd, *J* = 1.6, 1.0 Hz, 1H), 7.51 (dd, *J* = 2.5, 1.6 Hz, 1H), 7.29 – 7.23 (m, 2H), 5.99 (p, *J* = 6.1 Hz, 1H), 1.00 (s, 9H), 0.88 (s, 9H), 0.29 (s, 6H), 0.23 (s, 6H) ppm.
^{13}C NMR (151 MHz, CDCl$_3$) δ = 163.5, 155.4, 141.2, 132.5, 130.7, 129.2, 127.4, 126.8, 121.9, 120.9 (q, *J* = 281.6 Hz), 67.1 (p, *J* = 34.7 Hz), 26.5, 25.8, 18.4, 17.0, -4.3, -6.1.
^{19}F NMR (471 MHz, CDCl$_3$) δ = −73.1, −73.3, −73.6 ppm.
HRMS (EI): Calcd. for $C_{22}H_{34}F_6O_3Si_2$ = 516.19507, found = 516.19531.
IR (neat^{-1}): 2957, 1754, 1275, 1260, 1230 cm^{-1}.

1,1,1,3,3,3-hexafluoropropanyl dimethylbenzoate (2m) using $AgNO_3$ (1.5 equiv)

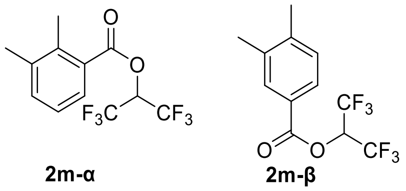

2m-α **2m-β**

Following the general procedure using *o*-xylene (127.4 mg, 1.20 mmol) and $AgNO_3$ (305.6 mg, 1.80 mmol, 1.5 equiv). Purification by flash column chromatography (pentane) afforded the title compound as a colorless solid in two fractions (Fraction 1 (3.8 mg, **2m-α**) and Fraction 2 (279.0 mg, **2m-β** containing a trace amount of **2m-α**), amounting to a total of 282.8 mg, 0.943 mmol, 78%, α:β = 4:96).

Fraction 1:

^{1}H NMR (500 MHz, $CDCl_3$) δ = 7.77 (d, *J* = 7.9 Hz, 1H), 7.40 (d, *J* = 7.5 Hz, 1H), 7.20 (t, *J* = 7.7 Hz, 1H), 6.00 (hept, *J* = 5.9 Hz, 1H), 2.52 – 2.47 (m, 3H), 2.36 (s, 3H) ppm.
^{13}C NMR (151 MHz, $CDCl_3$) δ = 164.5, 139.7, 138.7, 135.2, 131.1, 128.8, 127.1, 125.7, 120.8 (q, *J* = 282.6 Hz), 66.8 (hept, *J* = 34.6 Hz), 20.8, 16.8 ppm.
^{19}F NMR (471 MHz, $CDCl_3$) δ = −73.1 ppm.

Fraction 2:

^{1}H NMR (500 MHz, $CDCl_3$) δ = 7.93 – 7.81 (m, 2H, 2m-β), 7.78 – 7.76 (m, 0.03H, 2m-α), 7.40 (m, 0.3H, 2m-α), 7.31 – 7.24 (m, 1H, 2m-β), 7.23 – 7.18 (m, 0.04H, 2m-α), 6.01 (hept, *J* = 6.2 Hz, 1H), 2.50 (s, 0.08H, 2m-α), 2.35 (s, 3H, 2m-β), 2.34 (s, 3H, 2m-β) ppm.
^{13}C NMR (151 MHz, $CDCl_3$) δ = 163.6, 144.8, 137.6, 135.2, 131.5, 130.3, 128.8, 128.3, 125.7, 124.5, 120.8 (q, *J* = 282.9 Hz), 66.9 (hept, *J* = 34.7 Hz), 20.4, 19.8 ppm.
^{19}F NMR (471 MHz, $CDCl_3$) δ = −73.1, −73.2 ppm.

5.2 Synthetic Transformations of the HFIP-Ester Moiety

(3,4-dimethylphenyl)methanol (5)

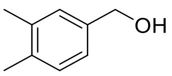

5

Under N_2-atmosphere, $LiAlH_4$ (5.3 mg, 0.14 mmol, 1.0 equiv) was suspended in dry Et_2O (0.7 mL) in a 10 mL Schlenk-flask containing a stirring bar. The solution was cooled to 0 °C using an ice-bath and a solution of 1,1,1,3,3,3-hexafluoropropan-2-yl 3,4-dimethylbenzoate (**2m**) (42.0 mg, 0.140 mmol) in dry Et_2O (0.7 mL) was added dropwise via syringe. After complete addition the ice-bath was removed and the mixture was stirred for another 3h at room temperature. The mixture was carefully quenched by the addition of sat. NH_4Cl solution (1.0 mL) followed by the addition of HCl (1M, 3.0 mL). The mixture was transferred to a separatory funnel containing distilled water (10 mL) and the aqueous phase was extracted with CH_2Cl_2 (4 x 20 mL). The combined organic phases were dried over anhydrous Na_2SO_4, filtered, and the solvent was removed under reduced pressure to obtain the title compound **5** as colorless solid (17.5 mg, 0.128 mmol, 92%). The spectral data are in accordance with those reported in literature.[25]

^{1}H NMR (600 MHz, $CDCl_3$) δ = 7.16 – 7.11 (m, 2H), 7.09 (dd, *J* = 7.7, 1.9 Hz, 1H), 4.62 (s, 2H), 2.27 (s, 3H), 2.26 (s, 3H), 1.66 (s, 1H) ppm.
^{13}C NMR (151 MHz, $CDCl_3$) δ = 138.5, 137.0, 136.2, 129.9, 128.7, 124.8, 124.7, 65.5, 19.9, 19.6 ppm.
HRMS (EI): Calcd. for $C_9H_{12}O$ = 136.08881, found = 136.08888.

***N*-benzyl-3,4-dimethylbenzamide (6)**

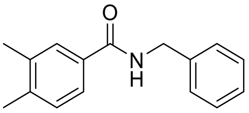

6

This reaction was performed in analogy to a report by Watson *et al.*[26] 1,1,1,3,3,3-hexafluoropropan-2-yl 3,4-dimethylbenzoate (**2m**) (42.0 mg, 0.140 mmol), K_3PO_4 (29.7 mg, 0.140 mmol, 1.0 equiv), dry THF (0.07 mL), and benzylamine (15.0 mg, 0.140 mmol, 1.0 equiv) were added to a 10 mL Schlenk-tube containing a stirring bar. The mixture was heated to 90 °C and stirred for 22 h. After cooling to room temperature, the mixture was transferred to a separatory funnel with the help of EtOAc (20 mL). The organic phase was extracted with NaOH (2M, 2 x 20 mL), HCl (1M, 2 x 20 mL), and brine (20 mL). Afterwards the organic phase was dried over anhydrous Na_2SO_4, filtered, and directly adsorbed on silica using a rotary evaporator. Purification by flash column chromatography (CH_2Cl_2 → CH_2Cl_2:MeOH = 99:1) afforded the title compound **6** as yellow solid (26.2 mg, 0.109 mmol, 78%).

^{1}H NMR (600 MHz, DMSO-d$_6$) = δ 8.91 (t, *J* = 6.0 Hz, 1H), 7.69 (d, *J* = 1.2 Hz, 1H), 7.62 (dd, *J* = 7.8, 2.0 Hz, 1H), 7.35 – 7.28 (m, 4H), 7.27 – 7.20 (m, 2H), 4.46 (d, *J* = 6.0 Hz, 2H), 2.26 (s, 6H) ppm.
^{13}C NMR (151 MHz, DMSO-d$_6$) δ 166.2, 139.9, 139.8, 136.2, 131.9, 129.3, 128.3, 128.3, 127.2, 126.7, 124.7, 42.5, 19.4, 19.4 ppm.
HRMS (EI): Calcd. for $C_{16}H_{17}NO$ = 239.13101, found = 239.13098.
IR (cm^{-1}): 3327, 2921, 1637, 1614, 1542, 1495, 1452, 1417, 1319, 1293, 1274.

(3,4-dimethylphenyl)(morpholino)methanone (7a)

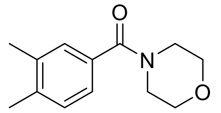

7a

This reaction was performed in analogy to a report by Watson *et al.*[26] 1,1,1,3,3,3-hexafluoropropan-2-yl 3,4-dimethylbenzoate (**2m**) (150.1 mg, 0.500 mmol), K_3PO_4 (106.1 mg, 0.500 mmol, 1.0 equiv), dry THF (0.25 mL), and morpholine (43.6 mg, 0.500 mmol, 1.0 equiv) were added to a 10 mL Schlenk-tube containing a stirring bar. The mixture was heated to 90 °C and stirred for 22 h. After cooling to room temperature, the mixture was transferred to a separatory funnel with the help of EtOAc (20 mL). The organic phase was extracted with NaOH (2M, 2 x 20 mL), HCl (1M, 2 x 20 mL), and brine (20 mL). Afterwards the organic phase was dried over anhydrous Na_2SO_4, filtered, and directly adsorbed on silica using a rotary evaporator. Purification by flash column chromatography (pentane:EtOAc 80:20) afforded the title compound as colorless oil (97.1 mg, 0.443 mmol, 89%). The spectral data are in accordance with those reported in literature.[27]

^{1}H NMR (600 MHz, $CDCl_3$) = δ 7.20 – 7.17 (m, 1H), 7.16 – 7.13 (m, 1H), 7.11 (dd, *J* = 7.6, 1.9 Hz, 1H), 3.87 – 3.33 (m, 1H), 2.27 (s, 6H) ppm.
^{13}C NMR (151 MHz, $CDCl_3$) = δ 170.8, 138.8, 137.1, 132.9, 129.6, 128.5, 124.6, 67.0, 48.4, 42.7, 19.8(4), 19.8(1) ppm.

3,4-Dimethylbenzophenone (7b)

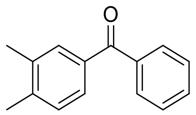

7b

This reaction was performed in analogy to a report by Vilarrasa *et al.*[28] (3,4-dimethylphenyl)(morpholino)methanone (**7a**) (96.5 mg, 0.44 mmol) and dry THF (8 mL) were added to a 25 mL Schlenk-tube containing a stirring bar. The mixture was cooled to 0 °C and over the course of 5 minutes phenylmagnesium bromide (3M in Et_2O, 0.59 mL, 1.76 mmol, 4.0 equiv) was slowly added to the solution. The mixture was stirred for 3 h at 0 °C and for 3 h at room temperature. Afterwards it was quenched with a saturated ammonium chloride solution (5 mL) and extracted with CH_2Cl_2 (3 x 30 mL). The combined organic phases were washed with a saturated ammonium chloride solution (10 mL), dried with Na_2SO_4, filtered, and directly adsorbed on silica using a rotary evaporator. Purification by flash column chromatography (pentane:EtOAc = 97:3) afforded the title compound **7b** as colorless solid (50.7 mg, 0.24 mmol, 55%). The spectral data are in accordance with those reported in literature.[29]

^{1}H NMR (600 MHz, $CDCl_3$) δ = 7.82 – 7.76 (m, 2H), 7.63 – 7.61 (m, 1H), 7.60 – 7.55 (m, 1H), 7.55 – 7.51 (m, 1H), 7.51 – 7.44 (m, 2H), 7.24 – 7.21 (m, 1H), 2.35 (s, 3H), 2.33 (s, 3H) ppm.
^{13}C NMR (151 MHz, $CDCl_3$) = δ 196.9, 142.1, 138.2, 136.9, 135.4, 132.2, 131.3, 130.1, 129.6, 128.3, 128.2, 20.2, 19.9 ppm.

(3,4-dimethylphenyl)diphenylmethanol (8)

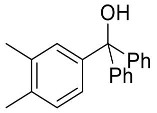

8

1,1,1,3,3,3-hexafluoropropan-2-yl 3,4-dimethylbenzoate (**2m**) (54.3 mg, 0.181 mmol) was added to a 10 mL Schlenk-tube containing a stirring bar. Under N_2-atmosphere dry THF (2.0 mL) followed by PhMgBr (3M in Et_2O, 0.36 mL, 0.11 mmol, 6.0 equiv) were added, the flask was tightly sealed, placed in a pre-heated aluminium block and stirred at 60 °C for 20 h. After cooling to room temperature, the reaction was carefully quenched by the addition of distilled water (2 mL) and saturated NH_4Cl-soluition (1 mL). The mixture was transferred to a separatory funnel with the help of CH_2Cl_2 (20 mL) and H_2O (20 mL) and the aqueous phase was extracted with CH_2Cl_2 (2 x 20 mL). The combined organic phases were dried over anhydrous Na_2SO_4, filtered, and directly absorbed on silica using a rotary evaporator. Column chromatography on silica gel (pentane → pentane:EtOAc = 94:6) afforded a yellow oil containing the title compound and a small amount of residual phenol. For further purification the title compound was re-dissolved in CH_2Cl_2 (20 mL) and transferred to a separatory funnel. The organic layer was extracted with NaOH (2M, 2 x 20 mL) and brine (20 mL), dried over anhydrous Na_2SO_4, and directly absorbed on silica using a rotary evaporator. Column chromatography on silica gel (pentane:EtOAc = 96:4) afforded the title compound as yellow oil (35.7 mg, 0.124 mmol, 69%). The spectral data are in accordance with those reported in literature.[30]

^{1}H NMR (600 MHz, CDCl$_3$) δ = 7.34 – 7.25 (m, 10H), 7.11 – 7.08 (m, 1H), 7.08 – 7.05 (m, 1H), 6.95 – 6.90 (m, 1H), 2.78 (s, 1H), 2.26 (s, 3H), 2.22 (s, 3H) ppm.
^{13}C NMR (151 MHz, CDCl$_3$) δ = 147.2, 144.6, 136.3, 135.8, 129.2, 129.1, 128.0, 128.0, 127.3, 125.6, 82.0, 20.1, 19.5 ppm.

Methyl 3,4-dimethylbenzoate (9)

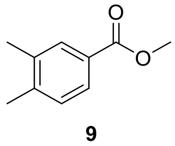

9

The transesterification procedure was inspired by Leadbeater *et al.*[31] An oven-dried 25 mL Schlenk-flask was charged with a stirring bar, $Pd(MeCN)_2Cl_2$ (5.2 mg, 0.020 mmol, 10 mol%), *N*-(2-((3-trifluoromethyl)thio)ethyl)acetamide (**BL1**) (21.0 mg, 0.0800 mmol, 40 mol%), 3-trifluoromethylquinoline (**ML1**) (7.9 mg, 0.040 mmol, 20 mol%), $AgNO_3$ (135.8 mg, 0.8000 mmol, 4.0 equiv), $Mo(CO)_6$ (17.4 mg, 0.066 mmol, 0.33 equiv), and HFIP (2.0 mL). The flask was tightly sealed and the mixture was stirred at room temperature for approximately 10 min. *o*-Xylene (**1m**,21.2 mg, 0.200 mmol, 1.0 equiv) was added, the flask was tightly sealed, placed in a pre-heated oil-bath at 80 °C, and the mixture was stirred with 1000 rpm for 24 h. After cooling to room-temperature the mixture was filtered through a pad of $MgSO_4$ and eluted with CH_2Cl_2 (70 mL). The solvent was removed under reduced pressure and NEt_3 (0.28 mL, 2.0 mmol, 10 equiv) and MeOH (4.0 mL) were added to the residue. The obtained mixture was stirred at 65 °C for 72 h. After cooling to room temperature, the crude reaction mixture was transferred to a separatory funnel with the help of Et_2O (20 mL) and extracted with aq. HCl (1M, 20 mL). The aqueous phase was extracted with Et_2O (2 x 20 mL) and the combined

organic phases were washed with brine (20 mL). The organic phase was dried with anhydrous Na_2SO_4, filtered and directly adsorbed on silica using a rotary evaporator. Purification by flash column chromatography (pentane → pentane:Et_2O = 99:1) afforded the title compound **9** as colorless oil (25.4 mg, 0.154 mmol, 77%). The spectral data are in accordance with those reported in literature.[32]

^{1}H NMR (600 MHz, $CDCl_3$) δ = 7.83 – 7.80 (m, 1H), 7.80 – 7.74 (m, 1H), 7.21 – 7.16 (m, 1H), 3.89 (s, 3H), 2.31 (s, 3H), 2.30 (s, 3H) ppm.
^{13}C NMR (151 MHz, $CDCl_3$) δ = 167.5, 142.4, 136.8, 130.8, 129.8, 127.9, 127.3, 52.1, 20.1, 19.8 ppm.
HRMS (EI): Calcd. for $C_{10}H_{12}O_2$ = 164.08373, found = 164.08380.

5.3 Net meta-Selective Carboxylation of Protected Phenols

3-(tert-butyldimethylsilyl)-5-hydroxybenzoic acid (10)

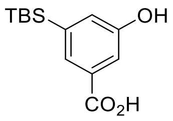

10

1,1,1,3,3,3-Hexafluoropropan-2-yl 3-(tert-butyldimethylsilyl)-5-((tert-butyldimethylsilyl)oxy) benzoate (**2s**) (104.0 mg, 0.201 mmol) was added to a 10 mL round-bottom flask containing a stirring bar. Dry THF (1.4 mL) and tetra-*N*-butylammonium fluoride (0.6 mL of a 1M solution in THF, 0.6 mmol, 3.0 equiv) were added, and the mixture was stirred at room temperature for 1 h. NaOH (2mL of a 2M solution in distilled water) was added and the mixture was stirred for another 24 h at room temperature. HCl (1M) was added until pH = 1 and the mixture was transferred to a separatory funnel. The aqueous phase was extracted with CH_2Cl_2 (4 x 20 mL), the combined organic phases were dried over anhydrous Na_2SO_4, filtered, and directly adsorbed on silica using a rotary evaporator. Purification by flash column chromatography (pentane:EtOAc:HCOOH = 89.5:10:0.5) afforded the title compound as colorless solid (40.7 mg, 0.161 mmol, 80%).

^{1}H NMR (600 MHz, DMSO-d$_6$) δ = 12.84 (s, 1H), 9.67 (s, 1H), 7.50 (dd, *J* = 1.5, 1.0 Hz, 1H), 7.32 (dd, *J* = 2.5, 1.5 Hz, 1H), 7.10 (dd, *J* = 2.6, 1.0 Hz, 1H), 0.84 (s, 9H), 0.24 (s, 6H) ppm.
^{13}C NMR (151 MHz, DMSO-d$_6$) δ = 167.6, 156.7, 138.9, 131.2, 125.5, 125.4, 116.4, 26.3, 16.5, -6.3 ppm.
HRMS (EI): Calcd. for $C_{13}H_{20}O_3Si$ = 252.11817, found = 252.11809.
IR (neat): 3400, 2950, 2925, 2854, 2881, 1654, 1594, 1581, 1471, 1438, 1314, 1277, 1260, 1204 cm^{-1}.

6. Preliminary Mechanistic Studies

To gain further insight into the mechanism of the reaction a series of experiments was performed. (all yields and selectivities were determined by GC-FID against mesitylene as internal standard on a 0.1 mmol scale for catalytic reactions or on a 0.05 mmol scale for stochiometric reactions). Procedures are identical to the reaction optimization above.

Notably, since the reaction is net-oxidative, an oxidant is required to enable catalyst turnover. Other oxidants than Ag-salts did not yield to any product formation, as evidenced by Table S 34.

Table S 34: Screening different oxidants.

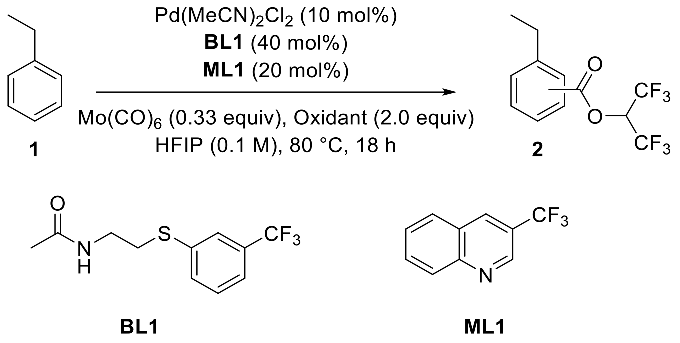

Entry	Oxidant (2.0 equiv)	**1** Conversion [%]	**2** o	m	p	∑ Yield **2** [%]	Conversion/ Yield (**2**)
1	**$AgNO_3$**	**96**	2	65	33	**80**	0,84
2	**$Cu(NO_3)_2 \cdot 3\,H_2O$**	**26**				**n.d.**	
3	**$CuCl_2 \cdot 2\,H_2O$**	**0**				**n.d.**	
4	**CuCl**	**0**				**n.d.**	
5	**CuO**	**0**				**n.d.**	
6	**$Cu(OTf)_2$**	**0**				**n.d.**	
7	**1,4-Benzoquinone**	**0**				**n.d.**	
8	**MnO_2**	**0**				**n.d.**	
9	**CuF_2**	**0**				**n.d.**	

To probe if the silver salt fulfils a role besides serving as terminal oxidant, we conducted stochiometric reactions with varying amounts of $AgNO_3$ (Table S35). Notably, in a stoichiometric reaction, where no catalyst turnover needs to occur, product formation should be possible in the absence of a compound serving only as terminal oxidant. Under these reactions conditions $AgNO_3$ was found to be essential for product formation, indicating a vital role of Ag^+ in the catalytic cycle or catalyst activation beyond the re-oxidation of palladium after product formation.

Table S 35: Stochiometric reactions using $Pd(MeCN)_2Cl_2$.

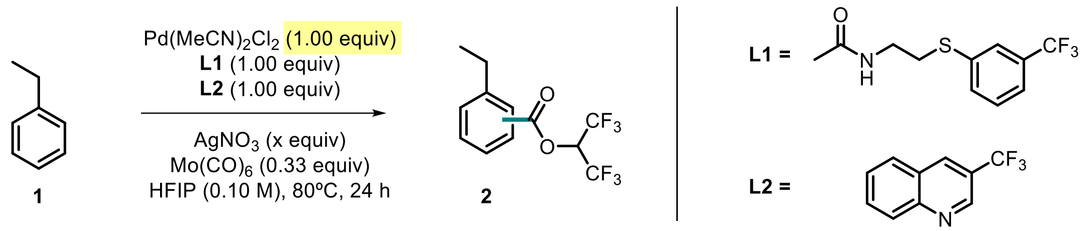

	Conv (1)	Yield (2)	Ratio o:m:p
No $AgNO_3$	0%	0%	-
$AgNO_3$ (0.5 equiv)	10%	4%	7:56:37
$AgNO_3$ (1.0 equiv)	51%	21%	3:59:38
$AgNO_3$ (1.5 equiv)	72%	40%	1:62:36
$AgNO_3$ (2.0 equiv)	87%	47%	1:64:35

Interestingly a stochiometric reaction using $Pd(NO_3)_2$ 2 H_2O resulted in significant formation of product **2** even in the absence of $AgNO_3$ (Table S36). This led us to hypothesize that the silver salt serves as halide scavenger to remove chloride ions from the palladium source.

Interestingly, the reaction with $Pd(NO_3)_2$ 2 H_2O afforded virtually the same regioselectivities as reactions with $Pd(MeCN)_2Cl_2$ and $AgNO_3$. This result indicates that the same catalytically active species/selectivity determining transition states are involved in both systems and thus excludes an active role of Ag^+-ions in the rate- and selectivity-determining C–H activation step.

Table S 36: Stochiometric reaction using $Pd(NO_3)_2$ 2 H_2O.

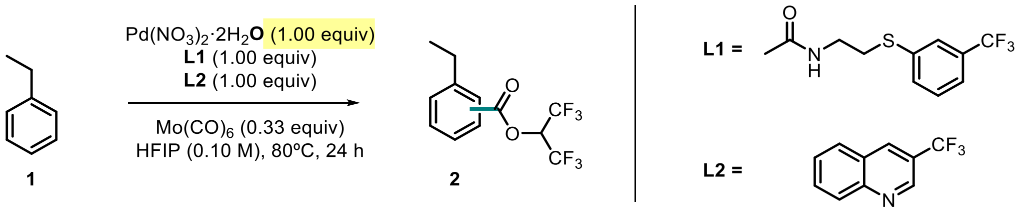

	Conv (1)	Yield (2)	Ratio o:m:p
No $AgNO_3$	67%	28%	3:62:35

Next we were interested to probe the role of $Mo(CO)_6$. At 90 °C, diisopropyl azodicarboxylate (DIAD) was shown to be a suitable source of CO in transition metal catalyzed carbonylation reactions.[33] When exchanging $Mo(CO)_6$ with DIAD under our reaction conditions at 90 °C we obtained up to 14% of product **2** (Table S 37), once more with the same regioselectivity as in the optimized protocol. This indicates that $Mo(CO)_6$ is solely a source of CO under the reaction conditions and otherwise not involved in the catalytic cycle.

Table S 37: Carbonylation reactions with DIAD.

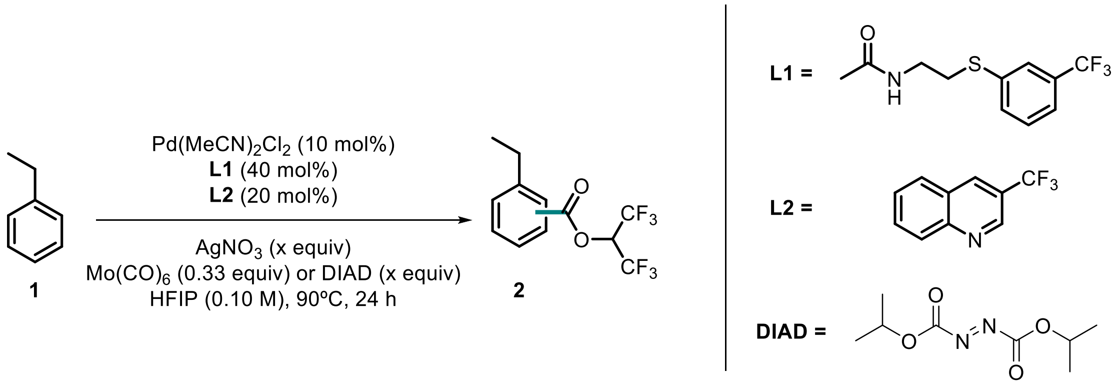

DIAD/Mo(CO)$_6$ (equiv)/ AgNO$_3$ (equiv)	Conv	Yield	Ratio o:m:p
**Mo(CO)$_6$ (0.33) / AgNO$_3$ (4.00)**	**80%**	**66%**	**0:65:35**
**DIAD (1.00) / AgNO$_3$ (4.00)**	**35%**	**7%**	**0:60:40**
**DIAD (1.00) / AgNO$_3$ (1.5)**	**32%**	**6%**	**0:60:40**
**DIAD (1.00) / AgNO$_3$ (1.00)**	**32%**	**8%**	**0:61:39**
**DIAD (1.00) / AgNO$_3$ (0.50)**	**24%**	**8%**	**0:66:34**
**DIAD (1.00) / No AgNO$_3$**	**6%**	**0%**	**-**
**DIAD (2.00) / AgNO$_3$ (1.50)**	**54%**	**14%**	**0:63:37**

Based on the observation that Mo(CO)$_6$ serves solely as CO source, we hypothesized that it should in principle be possible to replace it with gaseous CO. Notably, while Mo(CO)$_6$ is a highly convenient source of CO on a laboratory scale, this information would be highly relevant for practitioners considering to adopt our method on a larger scale. For safety considerations, we first opted to study CO generated chemically ex situ using a two-chamber setup (Figure S1). For CO generation we used a combination of Mo(CO)$_6$ and 1,8-Diazabicyclo[5.4.0]undec-7-en (DBU) in chamber 2, which was already demonstrated for Pd-catalyzed carbonylation reactions.[34] We employed 0.6 equiv of Mo(CO)$_6$ and 1.5 equiv of DBU and added the same amount of HFIP as in the reaction chamber.

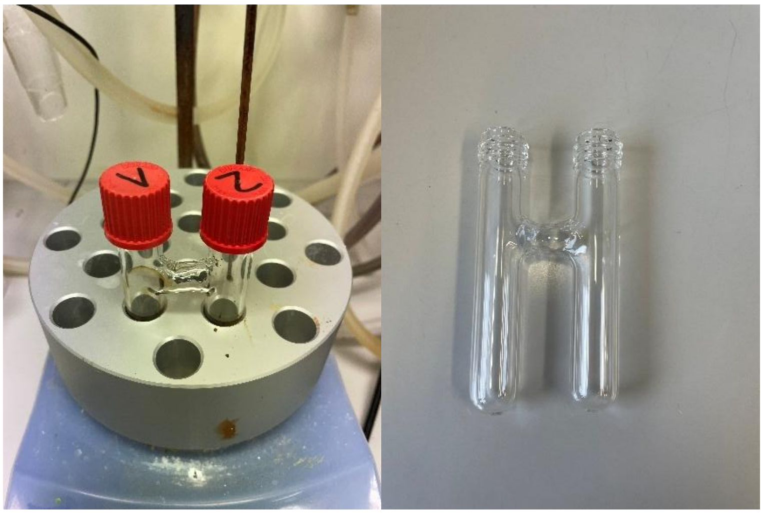

Figure S1: Two-chamber reaction vessel.

First, we performed stochiometric reactions using $Pd(MeCN)_2Cl_2$ and $Pd(NO_3)_2$ $2H_2O$ without the addition of $AgNO_3$ (Scheme S3). Again product formation was only observed when using $Pd(NO_3)_2$ 2 H_2O and the regioselectivity remained unaffected by the modified setup, suggesting that the same catalytic species/selectivity-determining transition states remain involved.

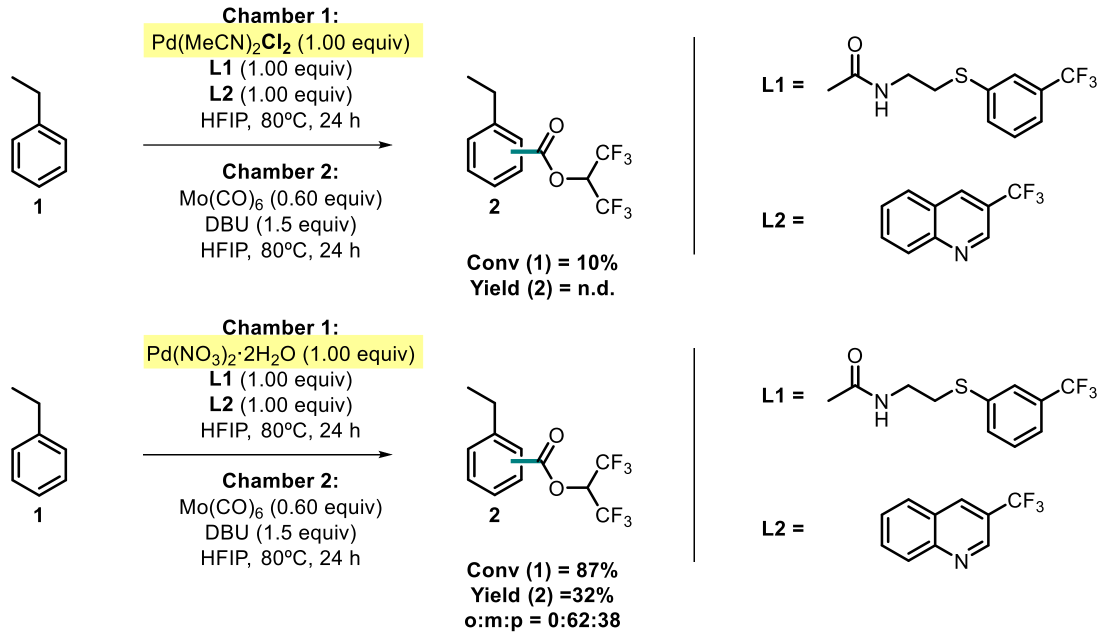

Scheme S3: Stochiometric reactions in a two-chamber setup.

Next, we performed four catalytic reactions using $Pd(MeCN)_2Cl_2$ and $Pd(NO_3)_2$ 2 H_2O in catalytic amounts, each with and without $AgNO_3$ (Scheme S4). While the two reactions in the presence of $AgNO_3$ lead to virtually identical yields and selectivities, the reactions in the absence of $AgNO_3$ differed significantly. While no product formation was overserved when using $Pd(MeCN)_2Cl_2$ as Pd-source, the reaction with $Pd(NO_3)_2$ 2 H_2O **2** in a yield of 17%. These results indicate that a Ag^+-free pathway is in principle feasible and confirm that silver serves two key roles in our system, activating the catalyst and re-oxidizing palladium after product formation. While such a silver-free system is highly attractive, the reaction using other oxidants remains inefficient under the current reaction conditions.

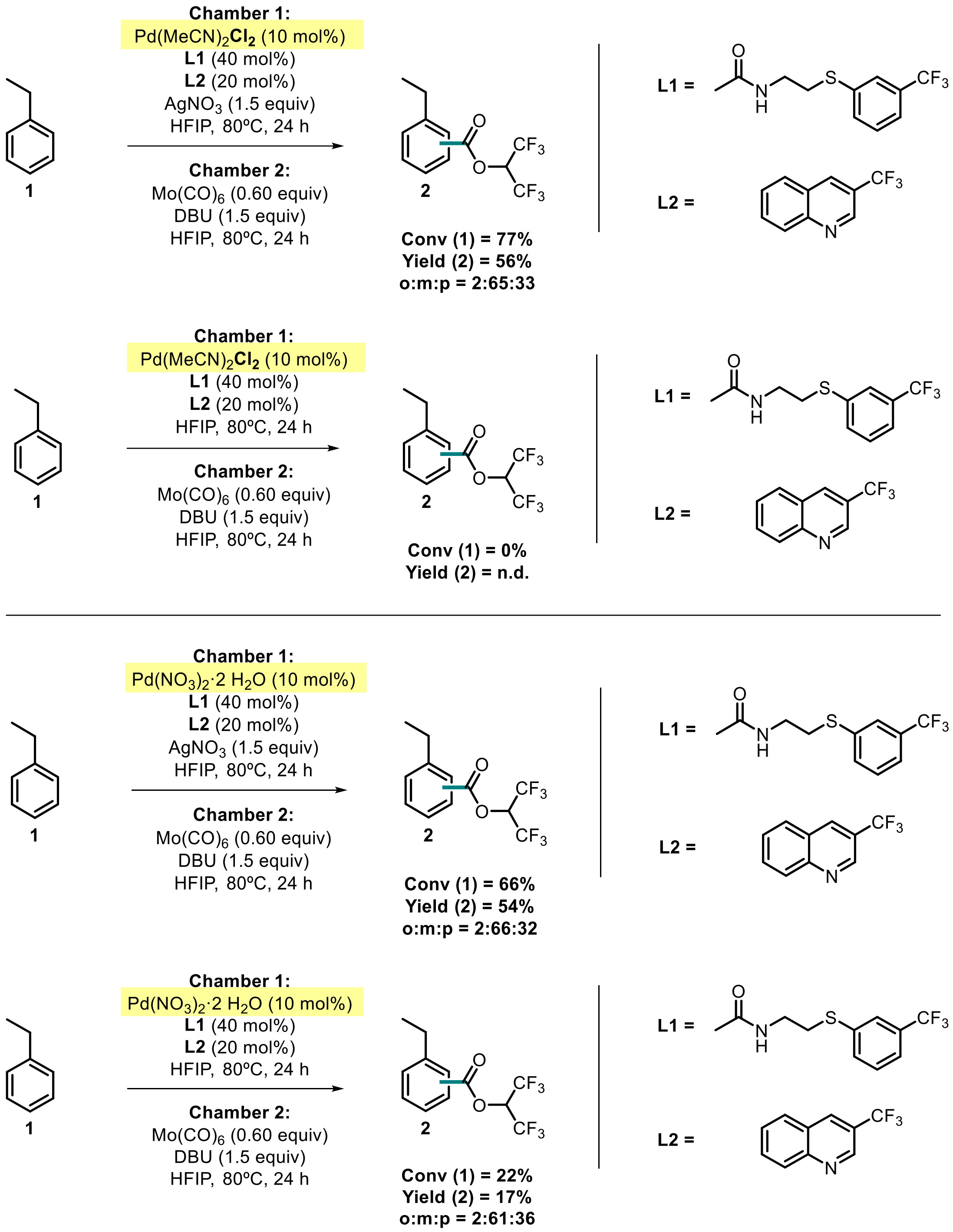

Scheme S4: Catalytic reactions in a two-chamber setup.

We performed two further reactions in a single chamber setup, probing the performance of $Pd(NO_3)_2$ 2 H_2O with and without $AgNO_3$ (Table S 38) when $Mo(CO)_6$ is present in the same solution. In comparison to the reaction with ex situ generated CO gas, the reaction without $AgNO_3$ gives significantly less yield when performed in a single chamber setup, while the reaction with $AgNO_3$ remains effective. This indicates that the silver salt serves a third role in our system, presumably the oxidation of low-valent Mo-species that could be detrimental for the reaction.[35]

Table S 38: Carbonylation reactions with $Pd(NO_3)_2$ 2 H_2O.

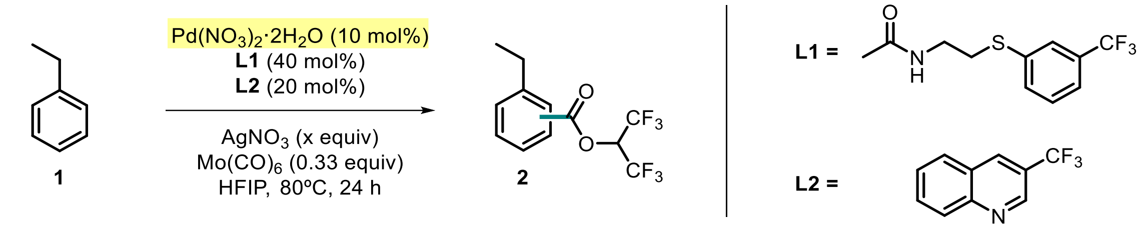

	Conv (1)	Yield (2)	Ratio o:m:p
**No $AgNO_3$**	**3%**	**3%**	**4:58:38**
**$AgNO_3$ (1.5 equiv)**	**93%**	**88%**	**2:65:33**

Lastly, we performed the reaction in a single chamber fashion using gaseous CO. These reactions were performed in 25 mL Schlenk tubes and the headspace was loaded to a pressure of 3 atm with a mixture of 10 vol% CO in He. Note: Using an ideal gas law approximation, this corresponds to the same maximum amount of CO available in the optimized reaction conditions. The reactions show the same trends as observed in the two-chamber reactions described above, albeit with lower yields and conversion rates (Table S 39).

Table S 39: Carbonylation reactions with CO in He

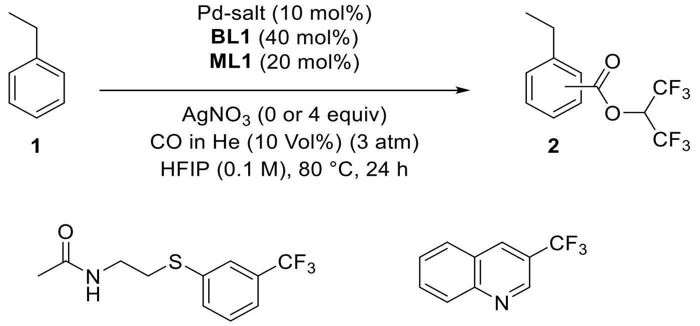

BL1 **ML1**

Entry	Pd-salt / $AgNO_3$ [equiv]	1 Conversion [%]	2 o	m	p	∑ Yield **2** [%]	Conversion/ Yield (**2**)
1	**$Pd(MeCN)_2Cl_2$ / 4**	**56**	1	63	36	**28**	0.50
2	**$Pd(MeCN)_2Cl_2$ / 4**	**55**	0	63	37	**22**	0.41
3	**$Pd(MeCN)_2Cl_2$ / 0**	**0**				**n.d.**	
4	**$Pd(NO_3)_2 \cdot 2H_2O$ / 4**	**61**	1	63	36	**39**	0.64
5	**$Pd(NO_3)_2 \cdot 2H_2O$ / 0**	**15**	3	60	37	**7**	0.49

Taken together, the experiments lead to the following conclusions. Neither Mo-species nor Ag-species are involved in the selectivity determining step. $Mo(CO)_6$ is solely a source of CO under the reaction conditions. $AgNO_3$ is needed to generate the active catalyst by halide abstraction, to re-oxidize low-valent Pd-species to Pd^{II} in the catalytic cycle and presumably to compensate for detrimental effects of low-valent Mo-species in solution.

Based on these observations and the extensive mechanistic studies previously conducted on our dual-ligand based Pd-catalysts,[36] as well as mechanistic knowledge on Pd-catalyzed carbonylation reported in literature,[37–39] a plausible mechanism is shown in scheme S5.

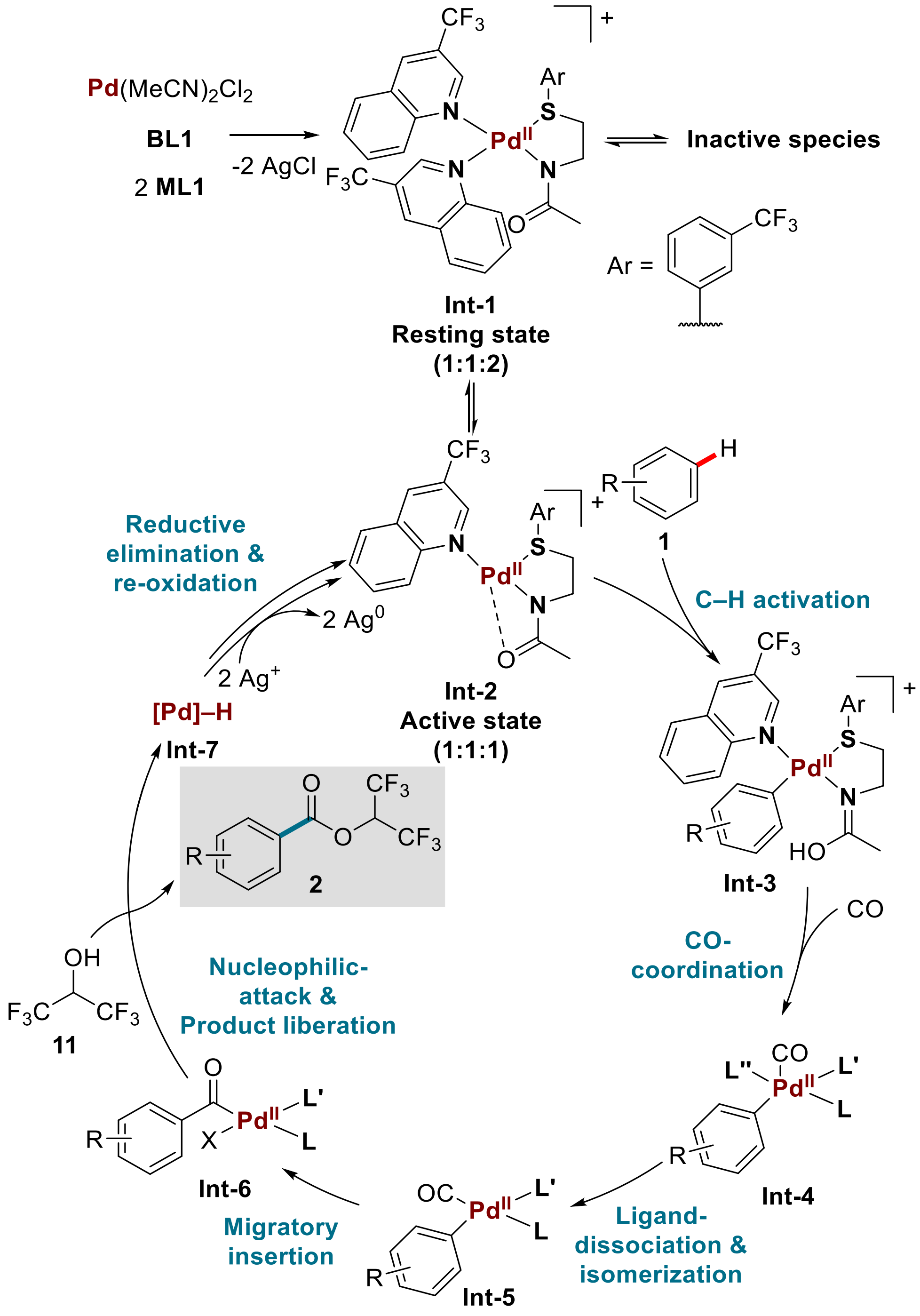

Scheme S5. Plausible catalytic cycle of the reaction.

First the Pd-salt forms a complex with the respective ligands in a stoichiometry of Pd:**BL**:**ML** = 1:1:2 (**Int-1**), as exemplified here with **BL2** and **ML3**. From the inactive resting state **Int-1** the active catalyst is generated via dissociation of one **ML3** to form **Int-2**. This can undergo C–H activation with the starting material **1** to generate the C–H activated species **Int-3**. From here an association of CO to form a penta-coordinated complex **Int-4** followed by dissociation of a ligand and isomerization to form **Int-5** is expected to take place.[37–39] Alternatively, CO could also coordinate to Pd in a dissociation-association pathway, meaning that ligand dissociation

takes place before CO-binding. In this case no penta-coordinated species are involved. Acyl-Pd complex **Int-6** is subsequently formed by migratory insertion. Nucleophilic attack by HFIP (**11**) liberates product **2** and forms **Int-7**.[40] From here, reductive elimination to Pd^0 and reoxidation by Ag^+ regenerates the active catalyst **Int-2**.

Importance of the counteranion in the Ag-salt:

During their studies, Yu *et al.* observed the formation of phthalic-anhydrides (**3**) as main products in sterically non-biased substrates.[41] In this study we found that the formation of **3** can be completely suppressed when the presence of acetate (e.g. from AgOAc or $Pd(OAc)_2$) is avoided. We reasoned that the formation of **3** could potentially occur as outlined in scheme S6. If acetate (**12**) is present in the mixture, **12** could outcompete HFIP (**11**) as nucleophile which would subsequently form anhydride **13**. Such a formation of anhydrides was already described by Fujiwara *et al.* in early studies.[42–44] **13** could react with **11** in two different ways. It could either form HFIP-ester **2** and acetic acid (**15**) or carboxylic acid **4** and HFIP-acetate (**14**). Once carboxylic acid **4** is formed, it would be expected to undergo a directed C–H carbonylation to form product **3** as described by Yu *et al.* in studies on related systems.[45]

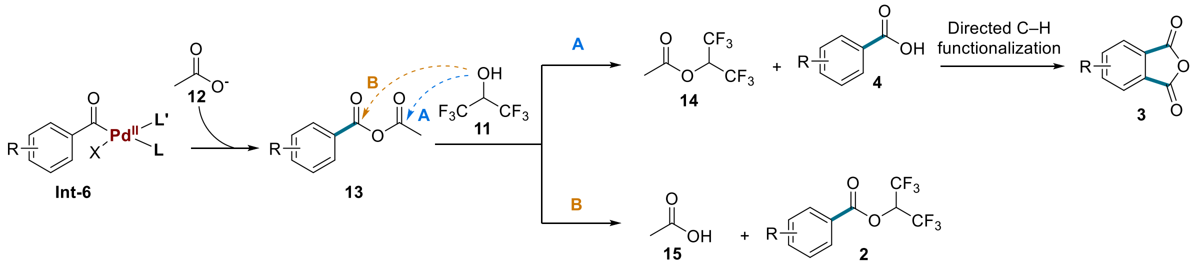

Scheme S6. Plausible pathway to the formation of **3**.

7. NMR-Spectra

7.1 Authentic Samples

1,1,1,3,3,3-Hexafluoropropan-2-yl 2-ethylbenzoate

^{1}H-NMR in $CDCl_3$

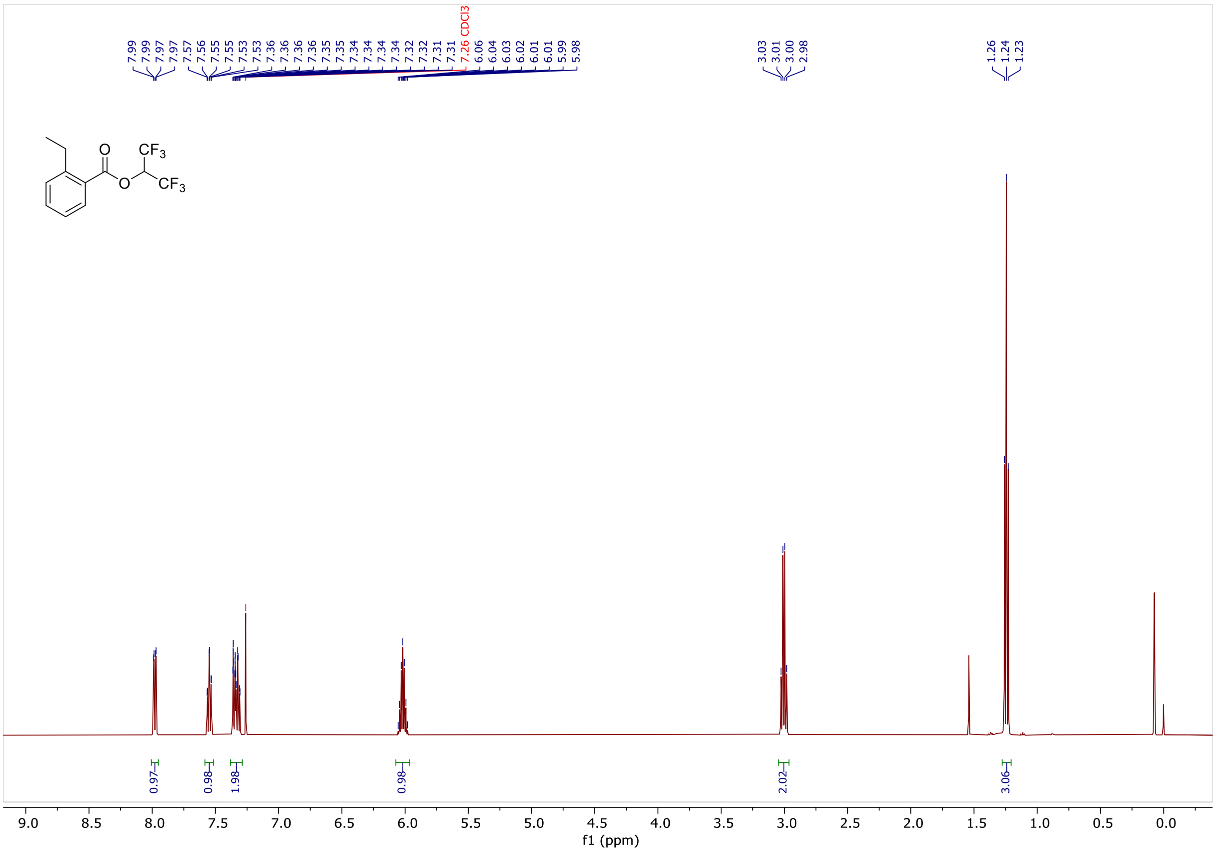

^{13}C-NMR in $CDCl_3$

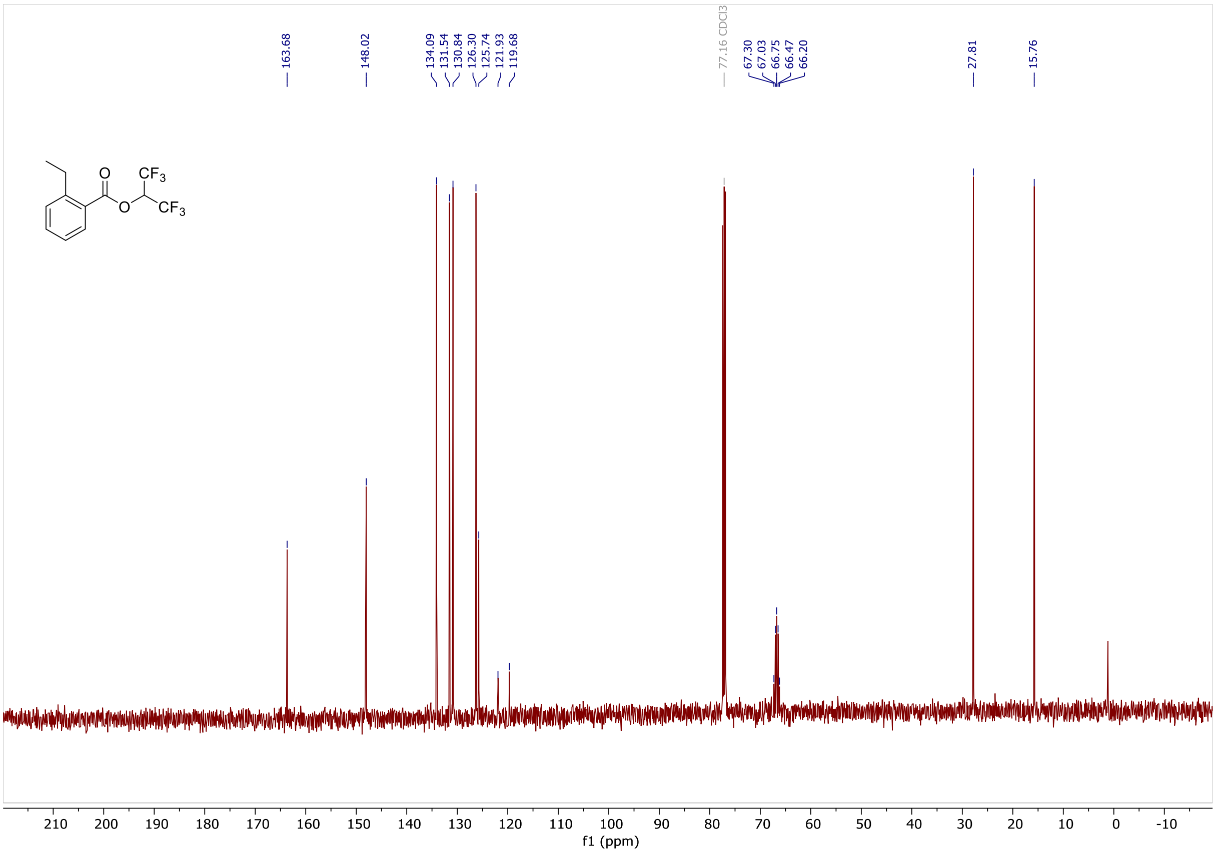

^{19}F-NMR in $CDCl_3$

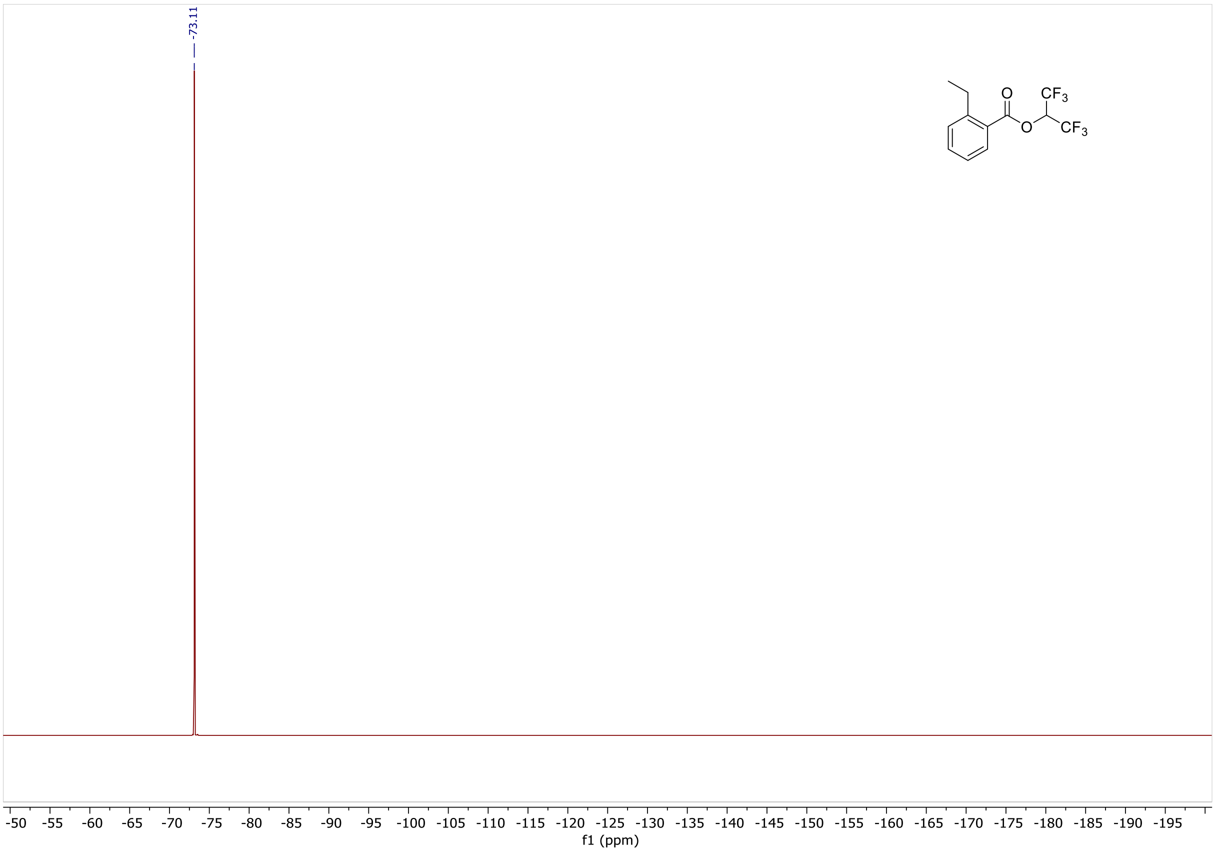

1,1,1,3,3,3-Hexafluoropropan-2-yl 3-ethylbenzoate

^{1}H-NMR in CDCl$_3$

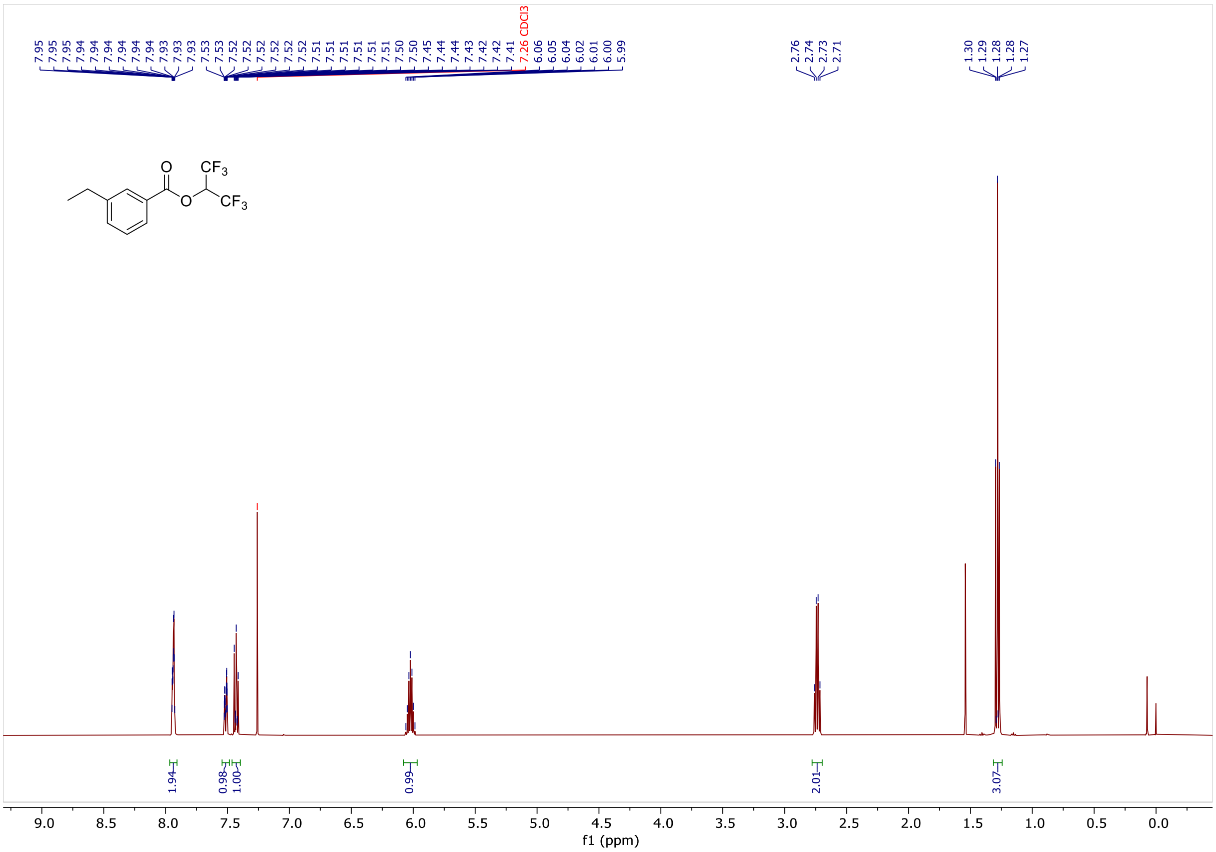

^{13}C-NMR in CDCl$_3$

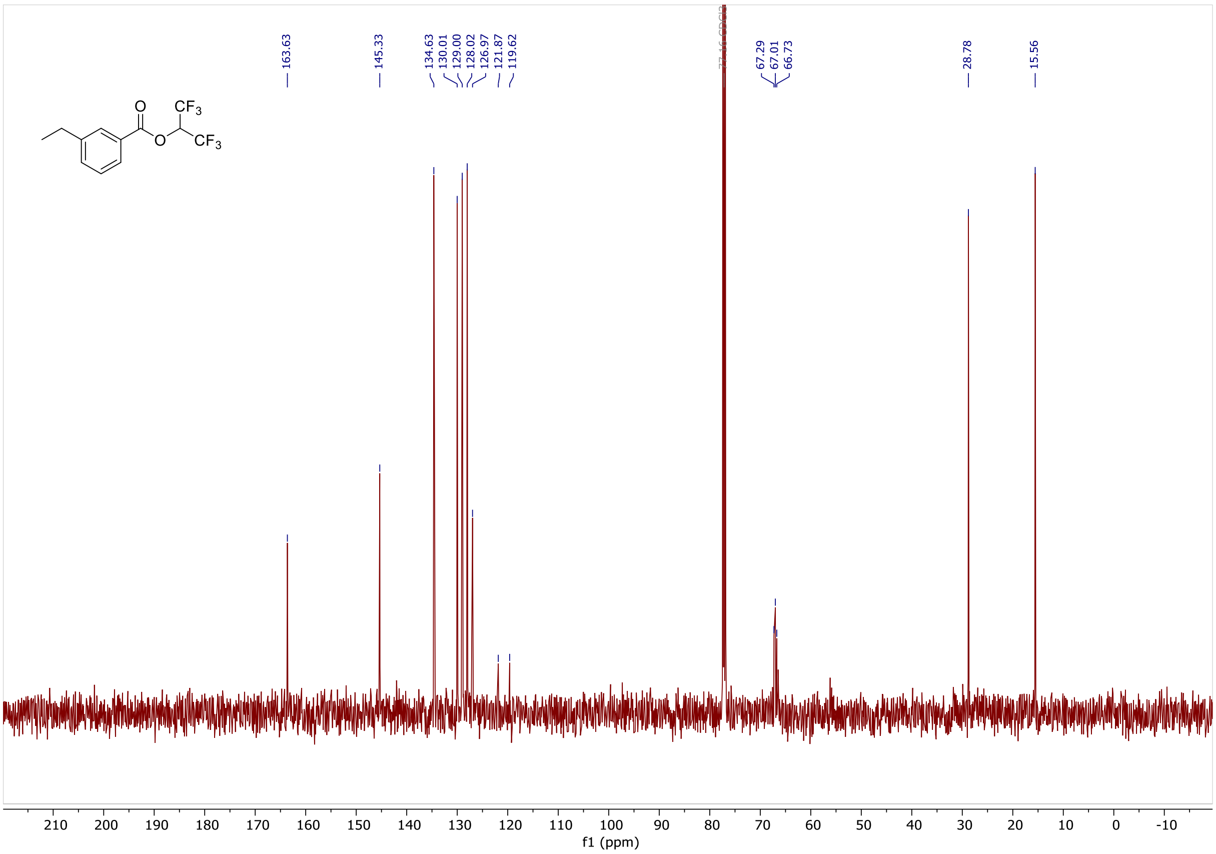

^{19}F-NMR in CDCl$_3$

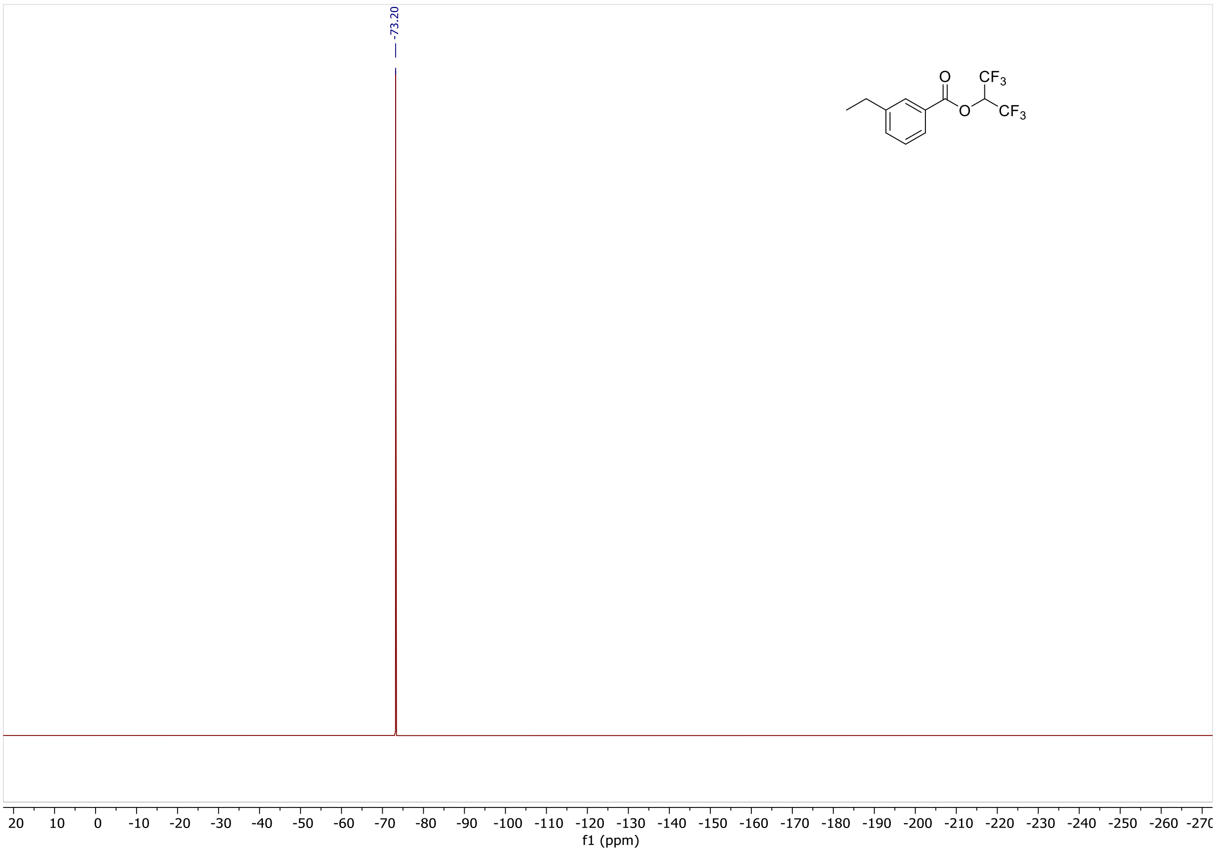

1,1,1,3,3,3-Hexafluoropropan-2-yl 4-ethylbenzoate

^{1}H-NMR in CDCl$_3$

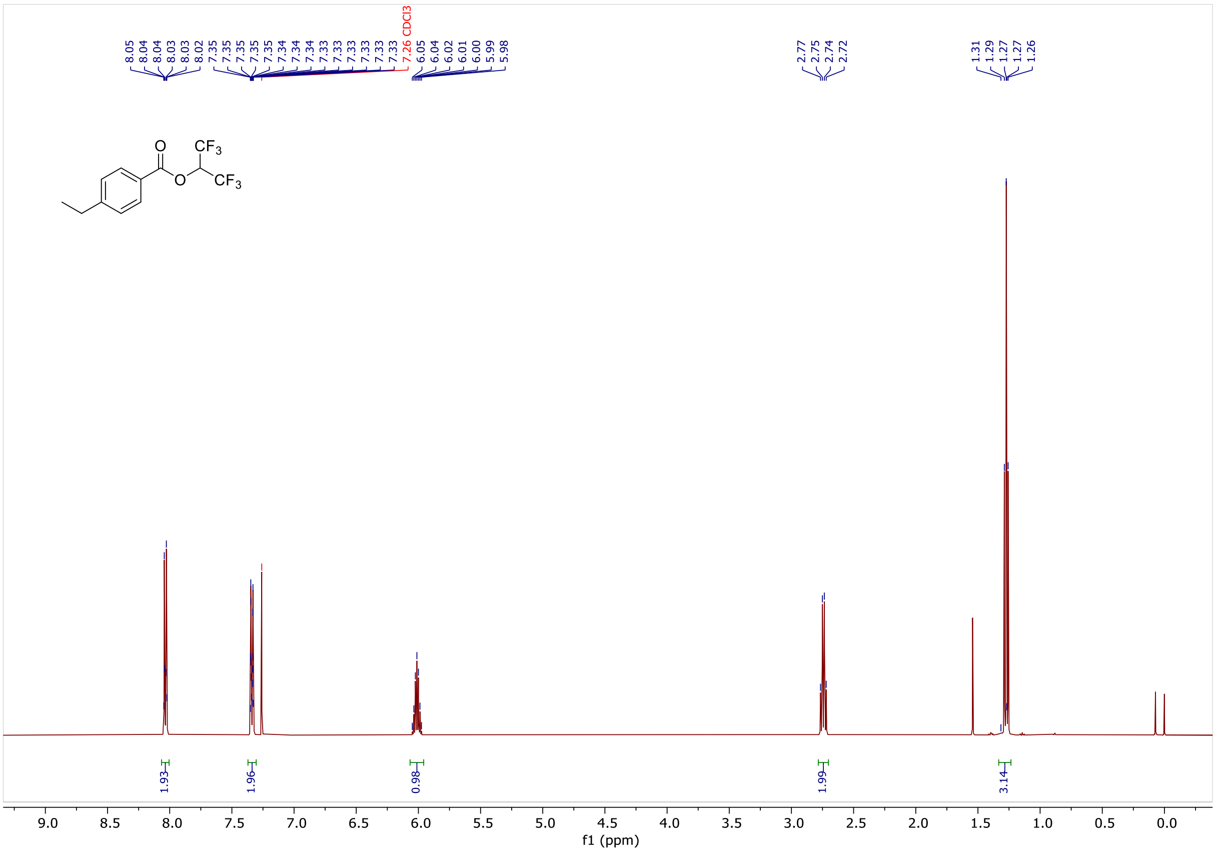

^{13}C-NMR in $CDCl_3$

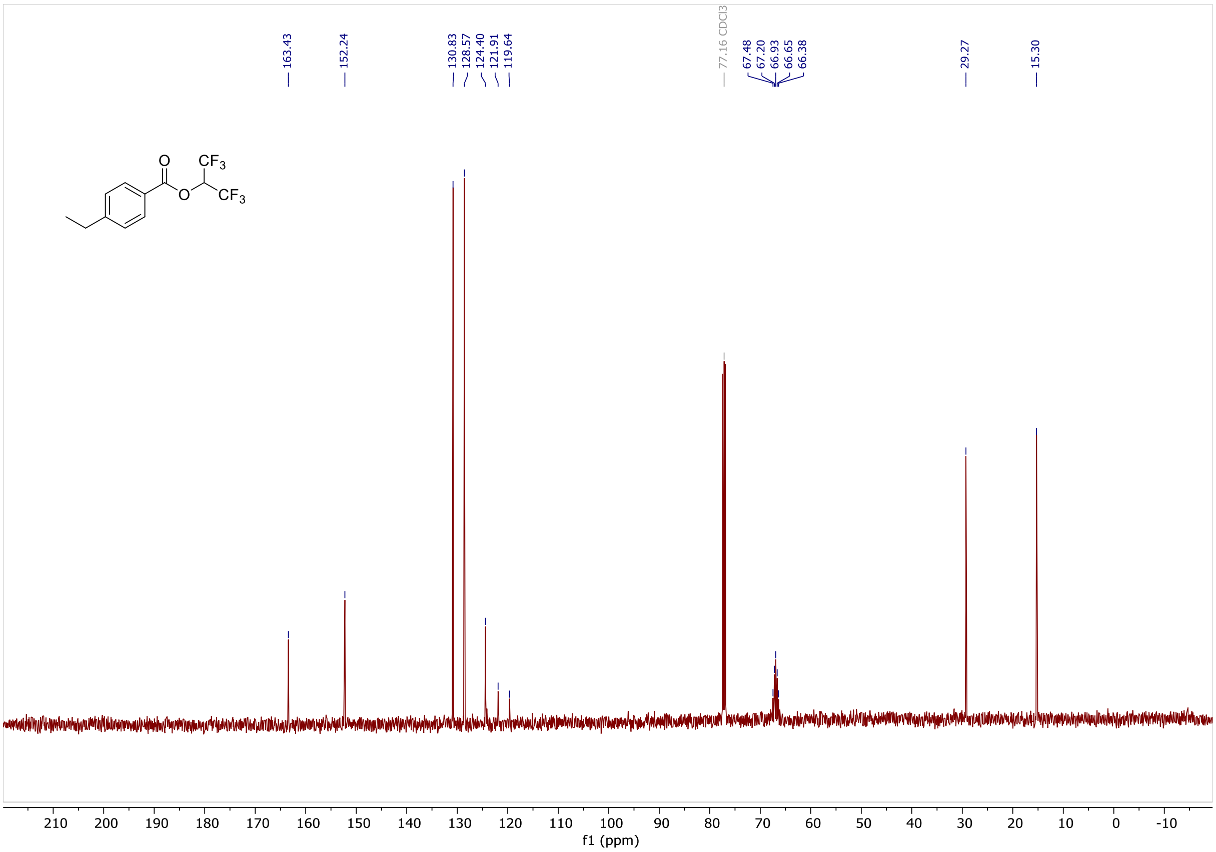

^{19}F-NMR in $CDCl_3$

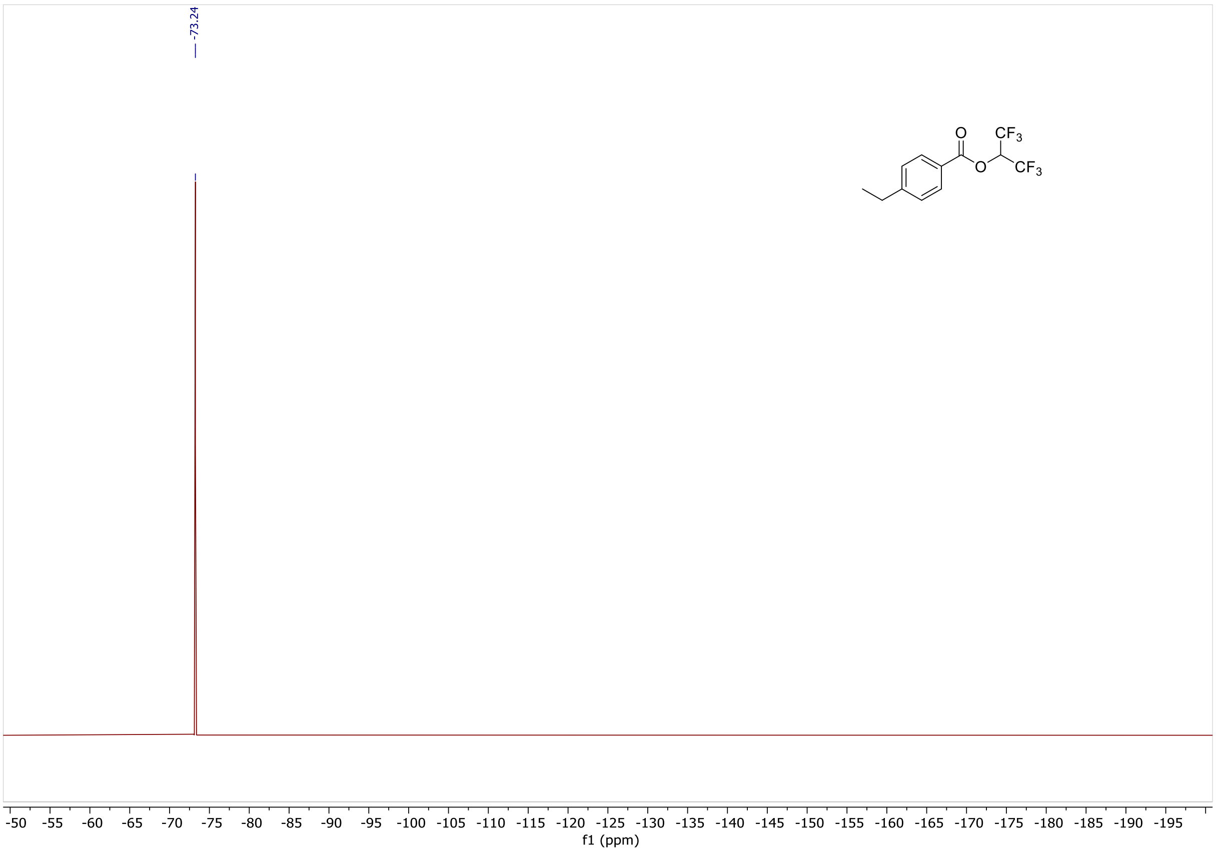

2,2,2-trifluoroethyl 4-ethylbenzoate

^{1}H-NMR in $CDCl_3$

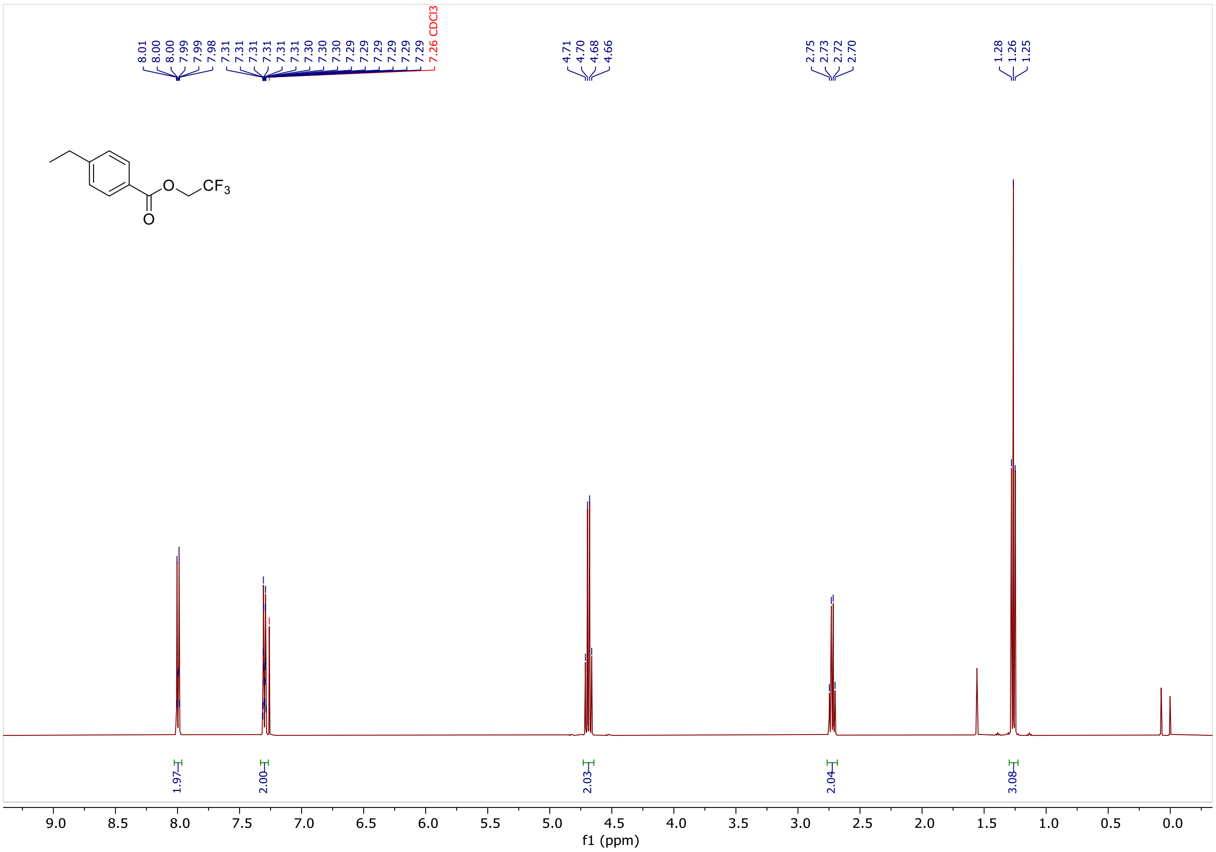

^{13}C-NMR in $CDCl_3$

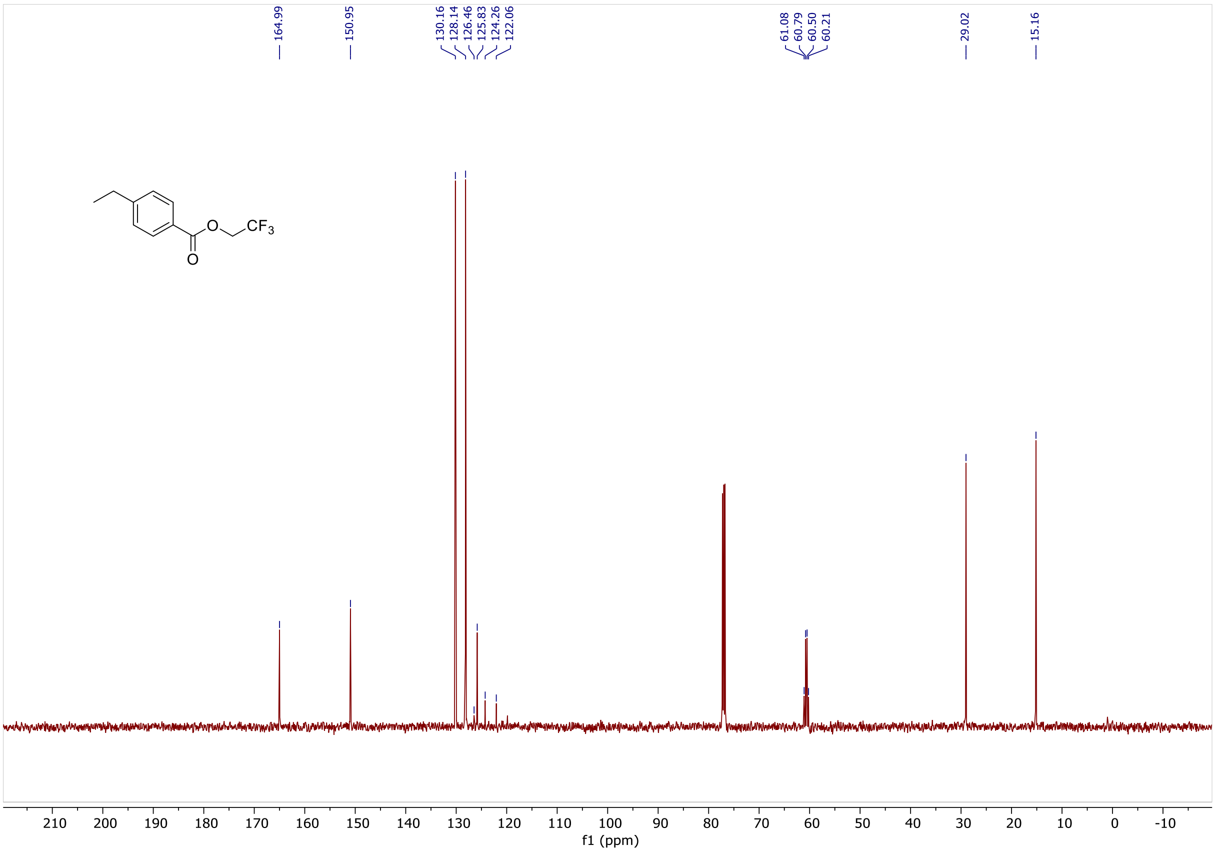

^{19}F-NMR in $CDCl_3$

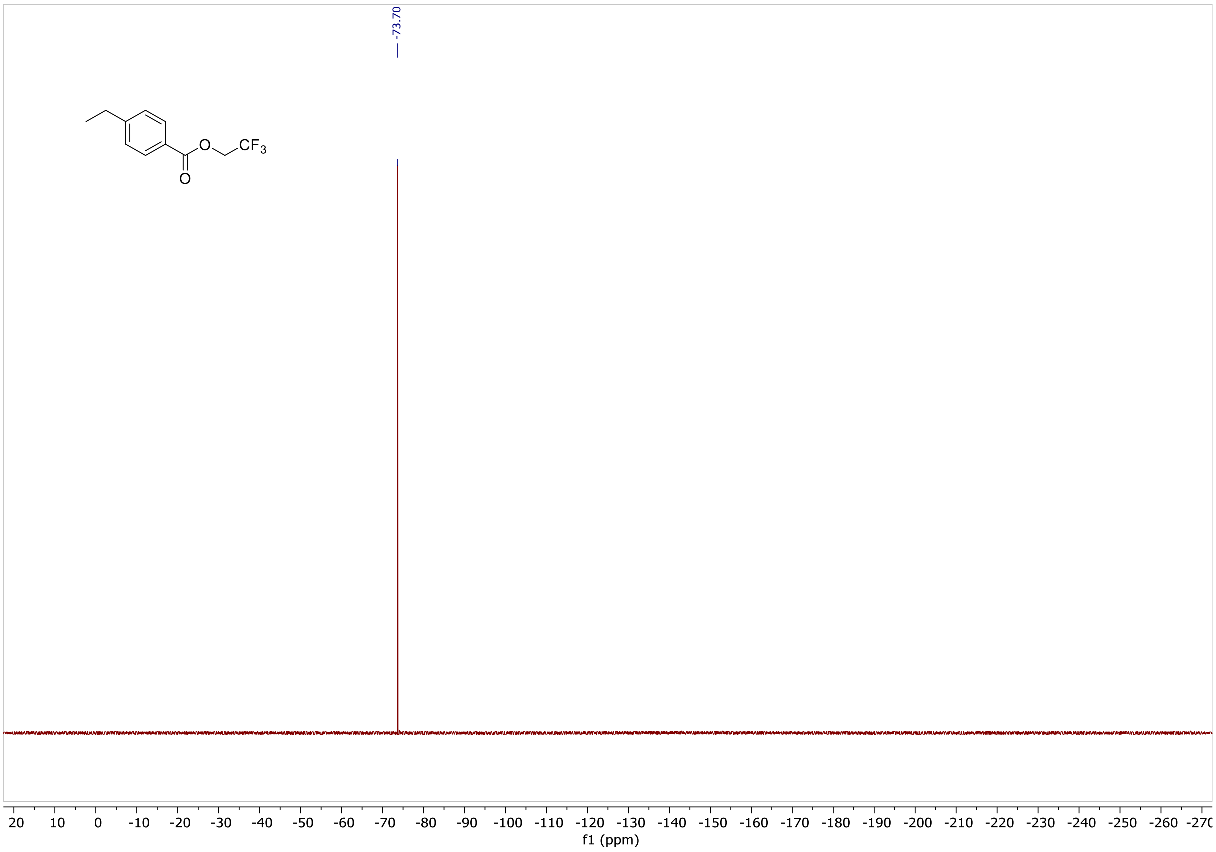

7.2 Ligands

N-(2-((3,5-Dimethylphenyl)thio)ethyl)acetamide

^{1}H-NMR in $CDCl_3$

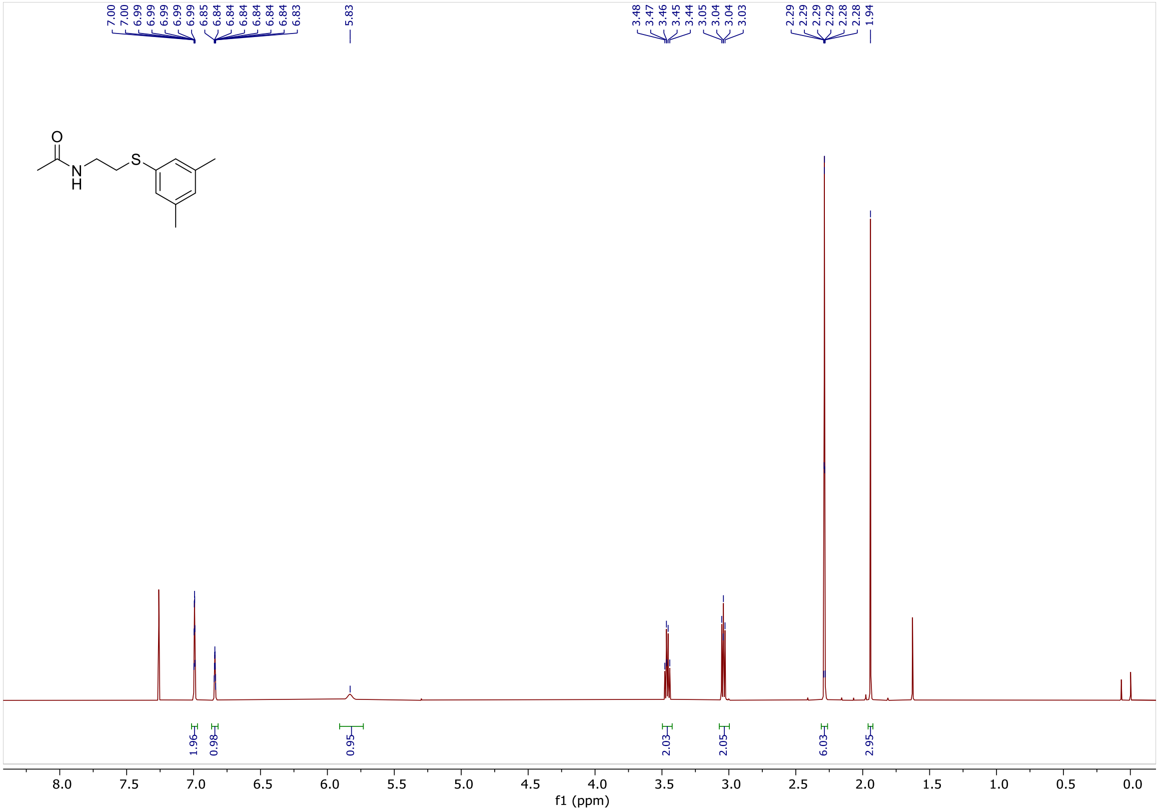

^{13}C-NMR in $CDCl_3$

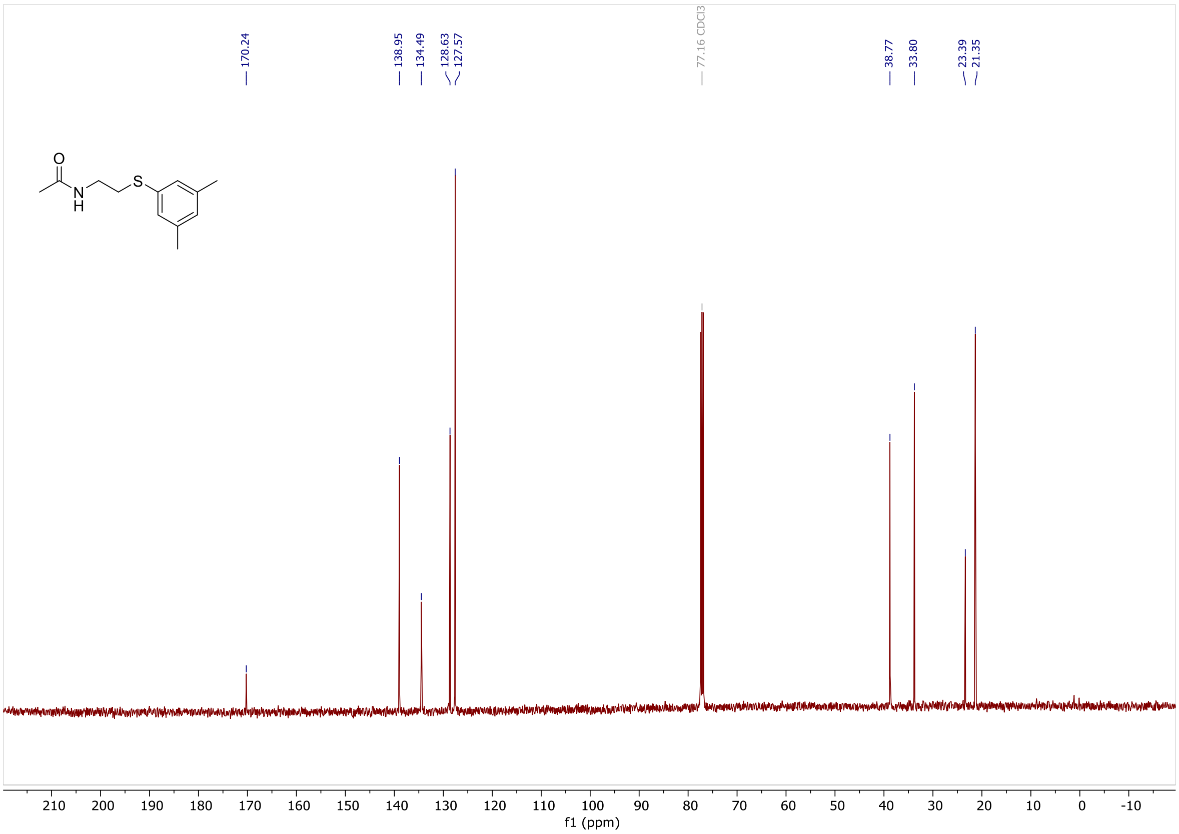

N-(2-((4-Trifluoromethyl)thio)ethyl)acetamide

^{1}H-NMR in $CDCl_3$

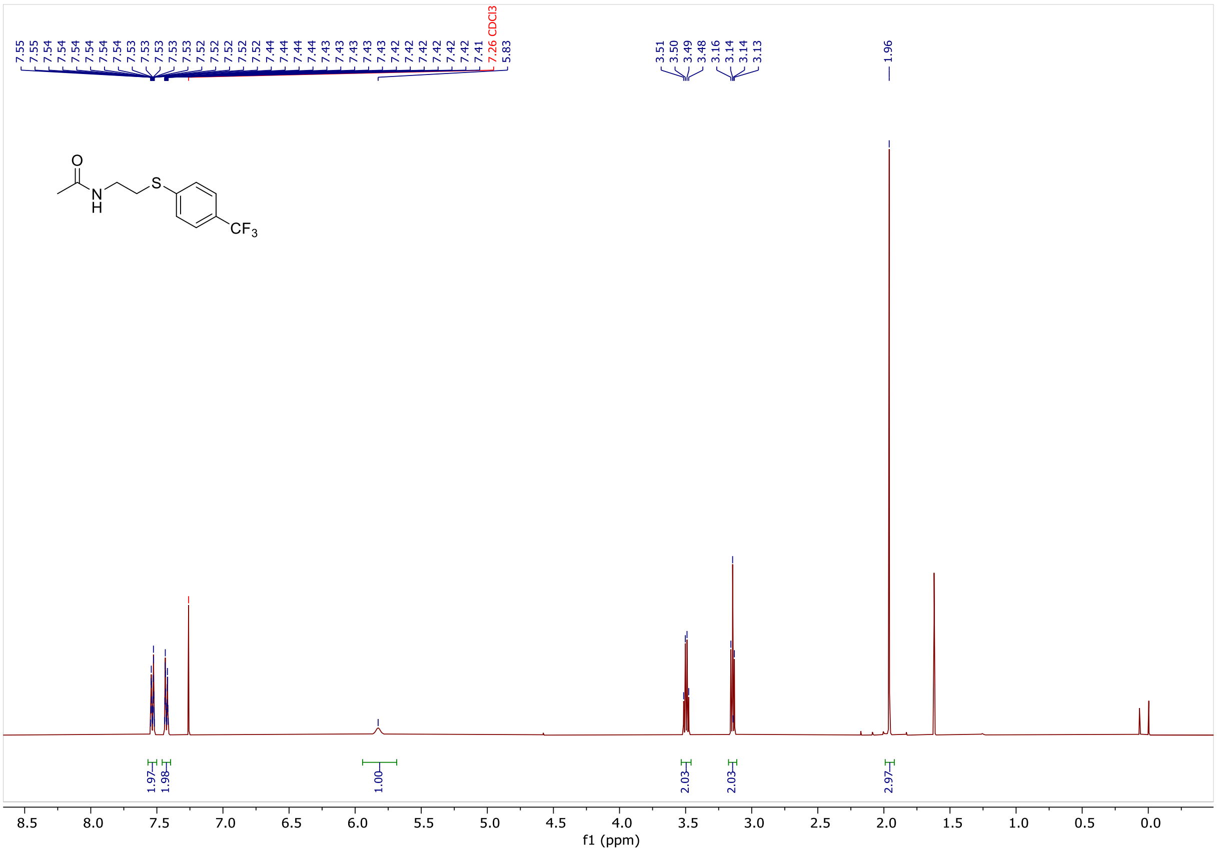

^{13}C-NMR in $CDCl_3$

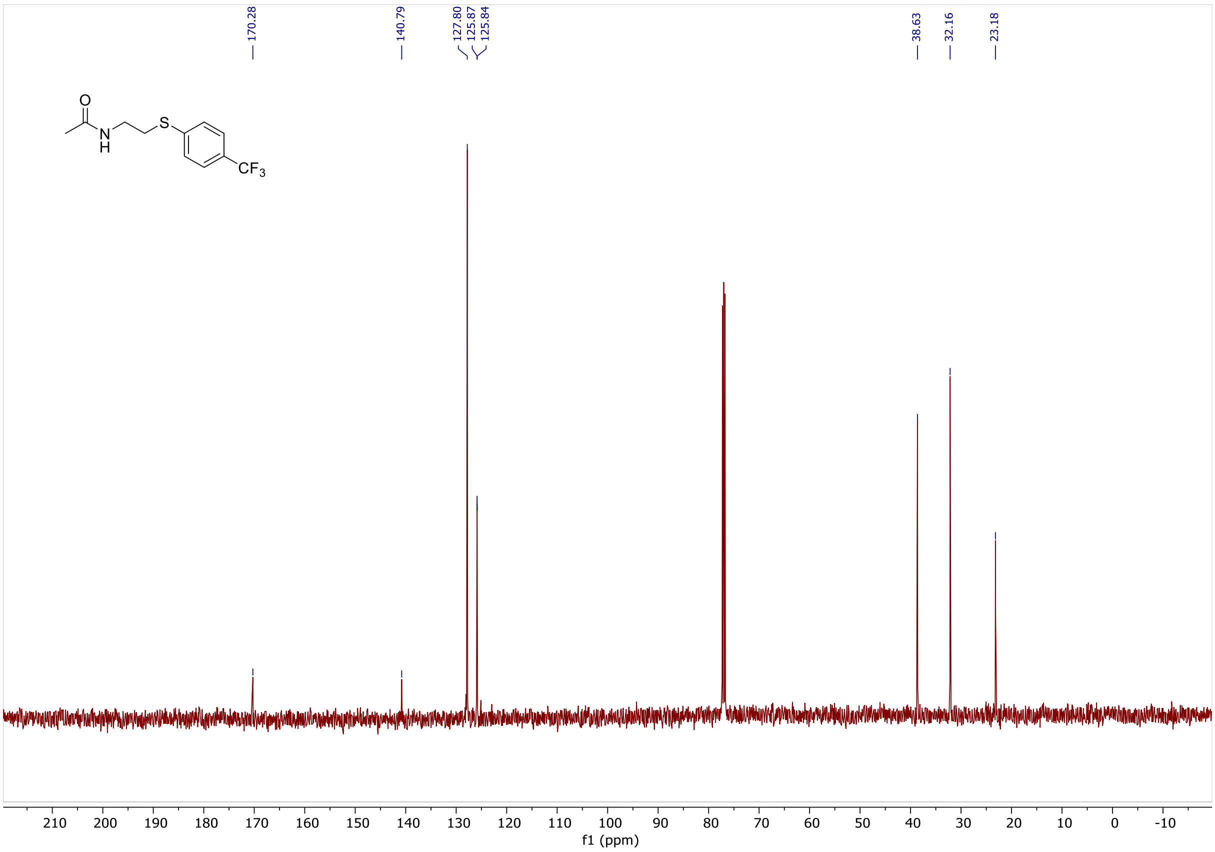

^{19}F-NMR in $CDCl_3$

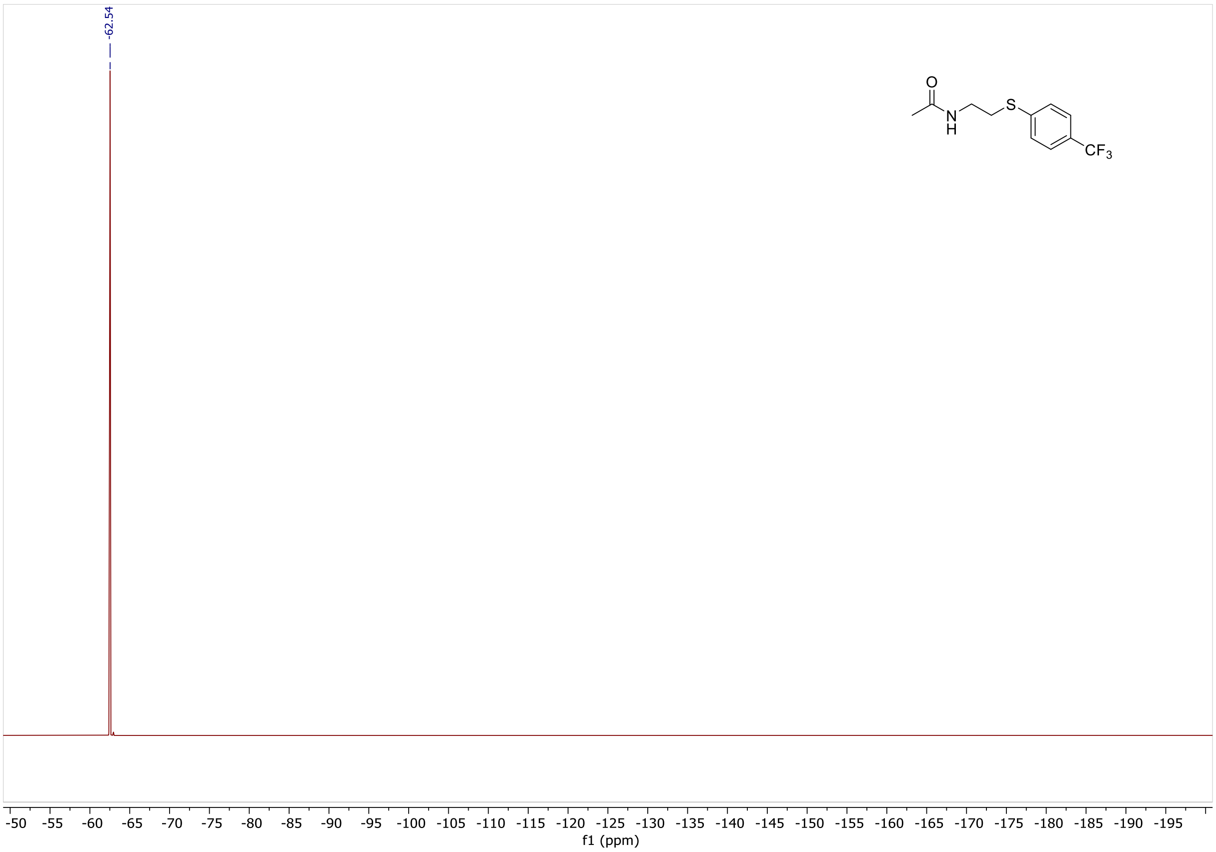

N-(2-((3-Trifluoromethyl)thio)ethyl)acetamide (BL1)

^{1}H-NMR in $CDCl_3$

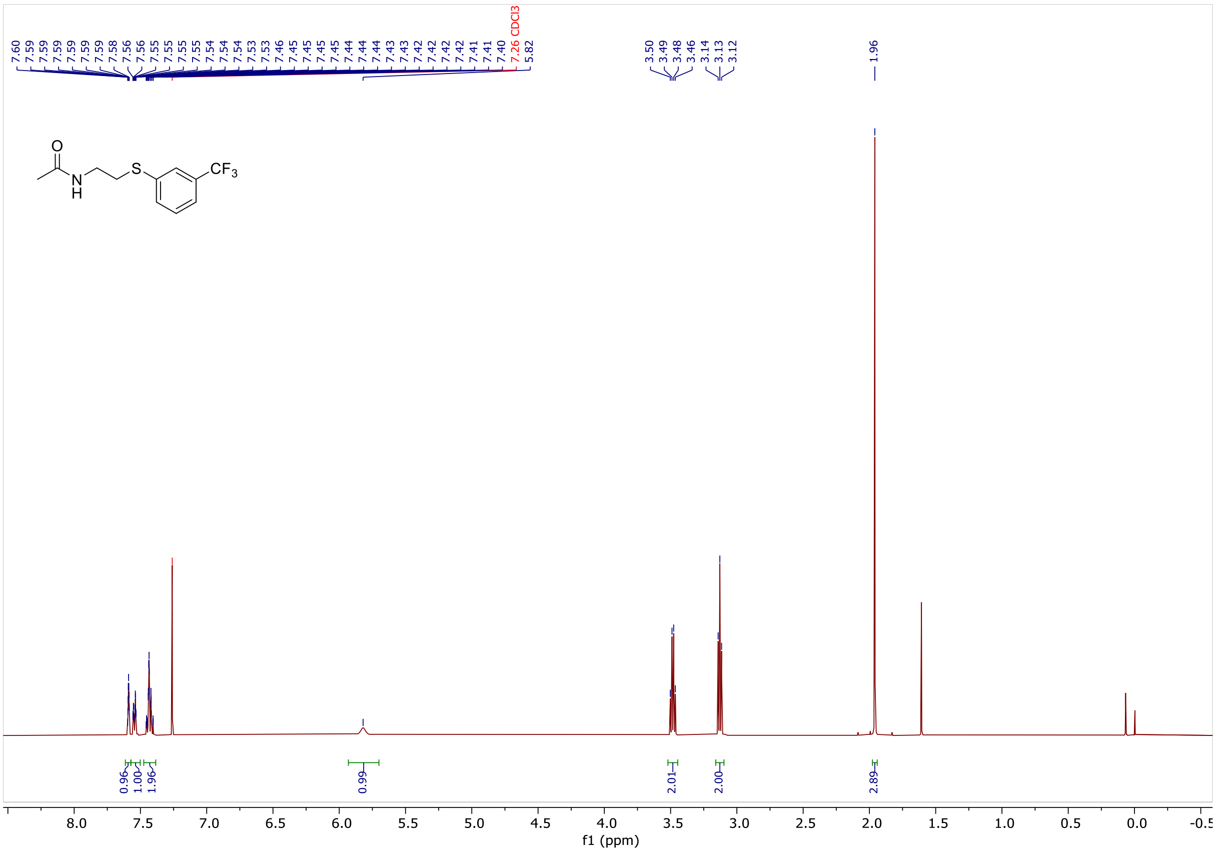

^{13}C-NMR in $CDCl_3$

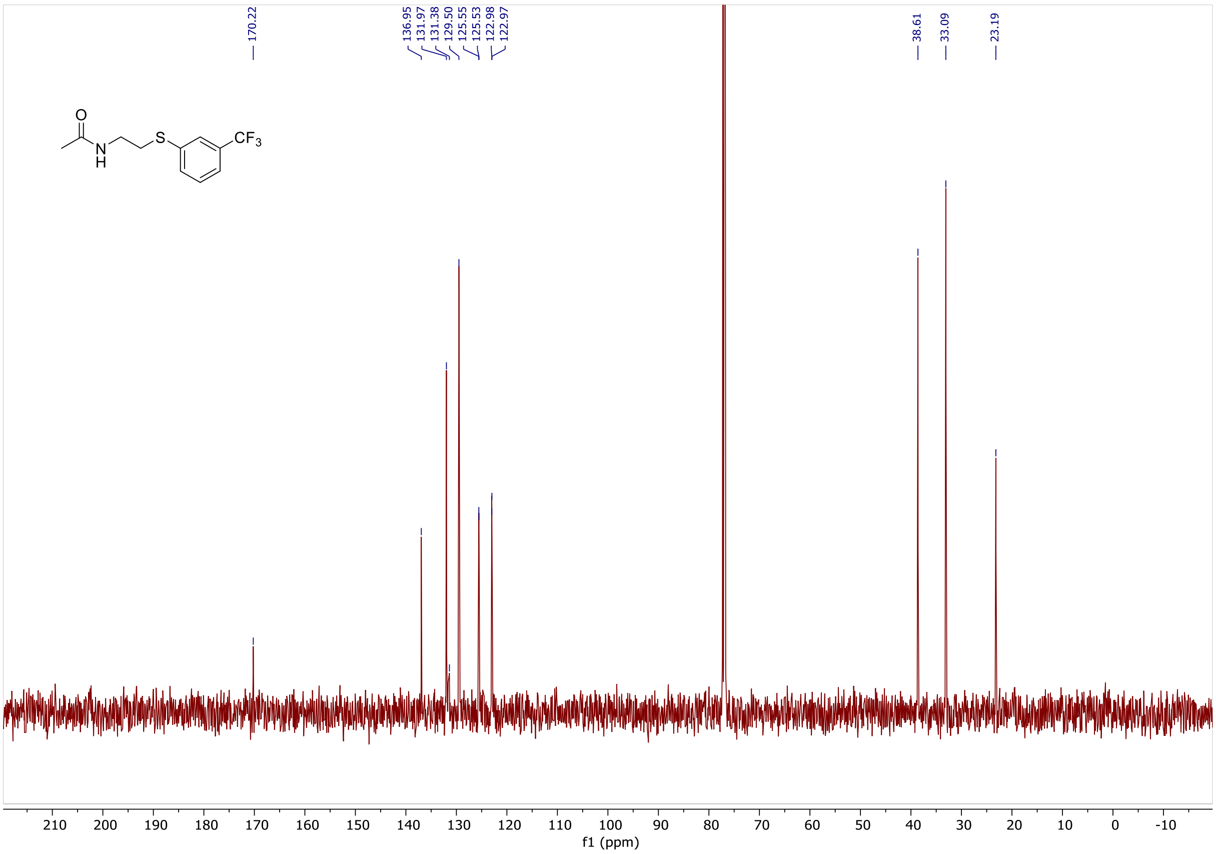

^{19}F-NMR in $CDCl_3$

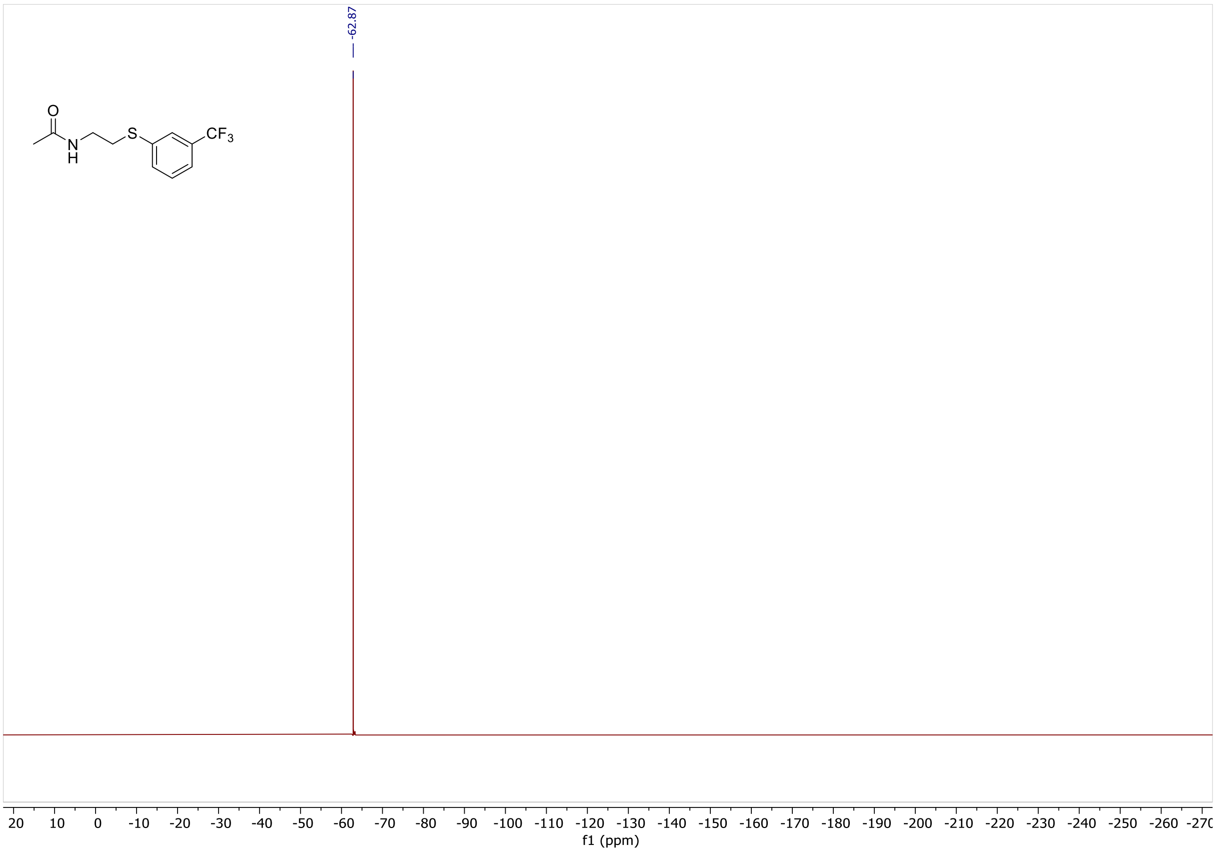

N-(2-((3,5-Bis(trifluoromethyl)phenyl)thio)ethyl)acetamide

^{1}H-NMR in $CDCl_3$

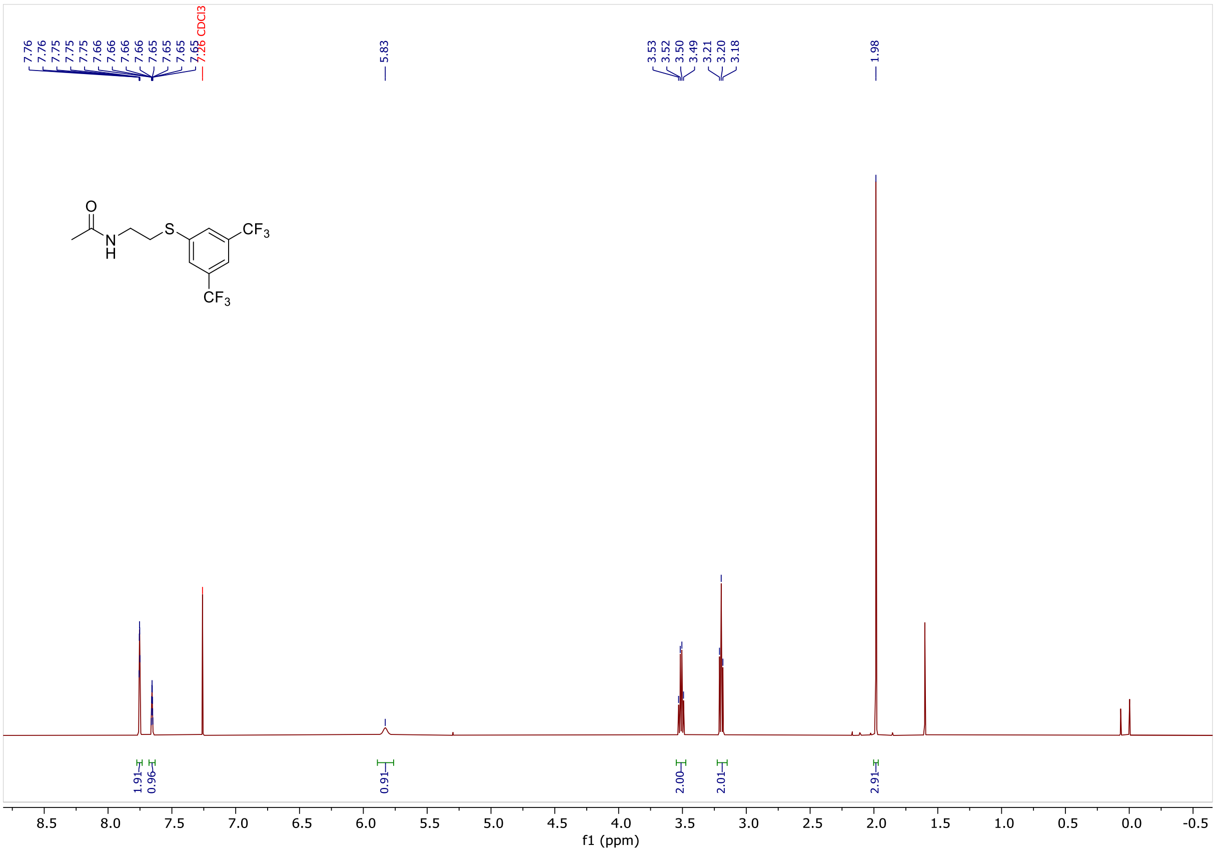

^{13}C-NMR in $CDCl_3$

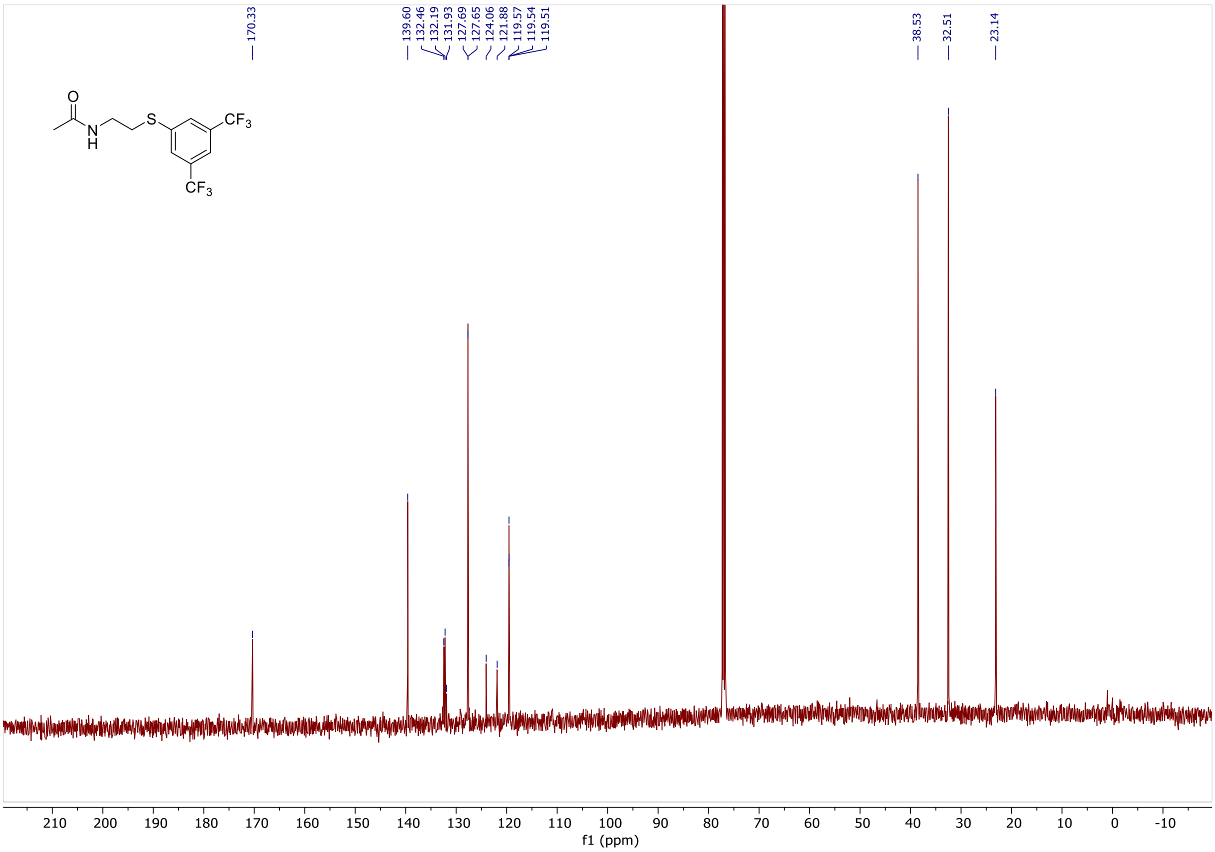

^{19}F-NMR in CDCl$_3$

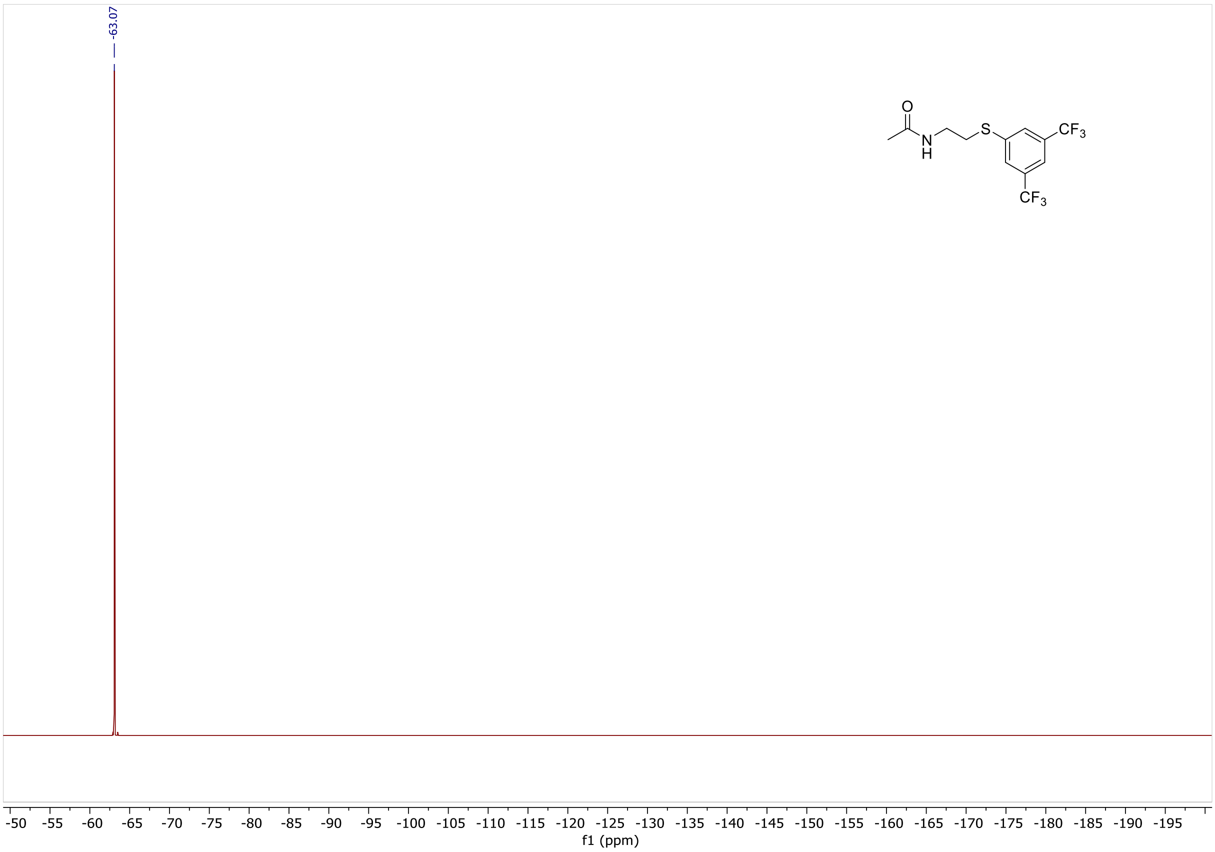

N-(2-((3-(Trifluoromethyl)phenyl)sulfinyl)ethyl)acetamide

^{1}H-NMR in DMSO-d_6

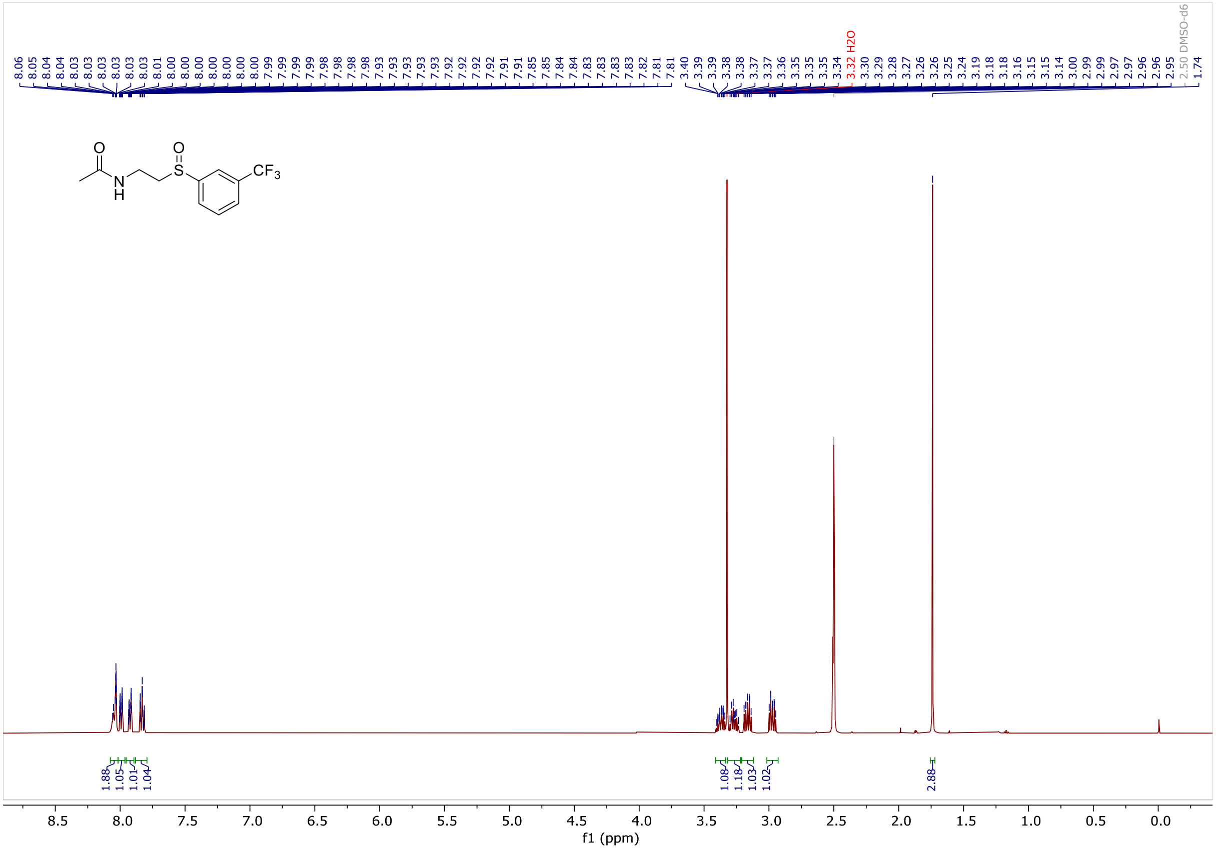

^{13}C-NMR in DMSO-d_6

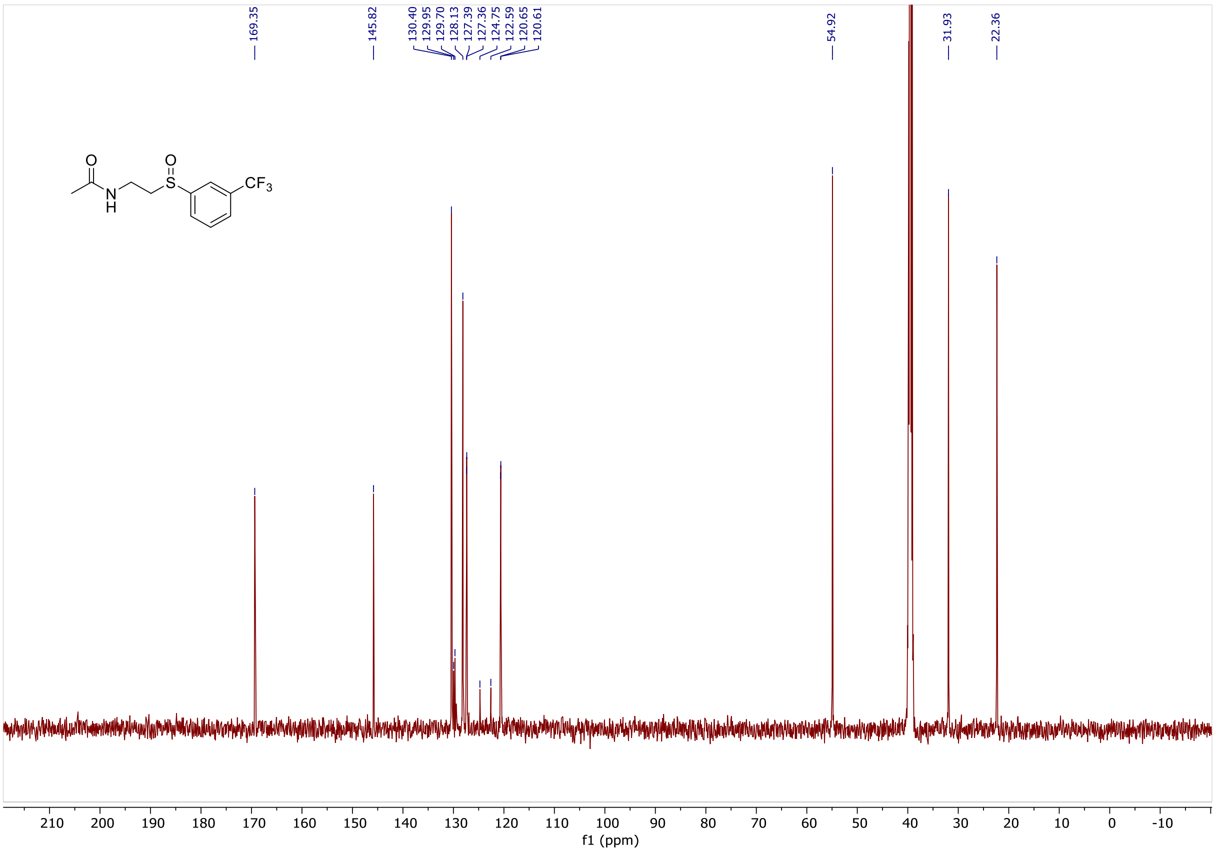

^{19}F-NMR in DMSO-d$_6$

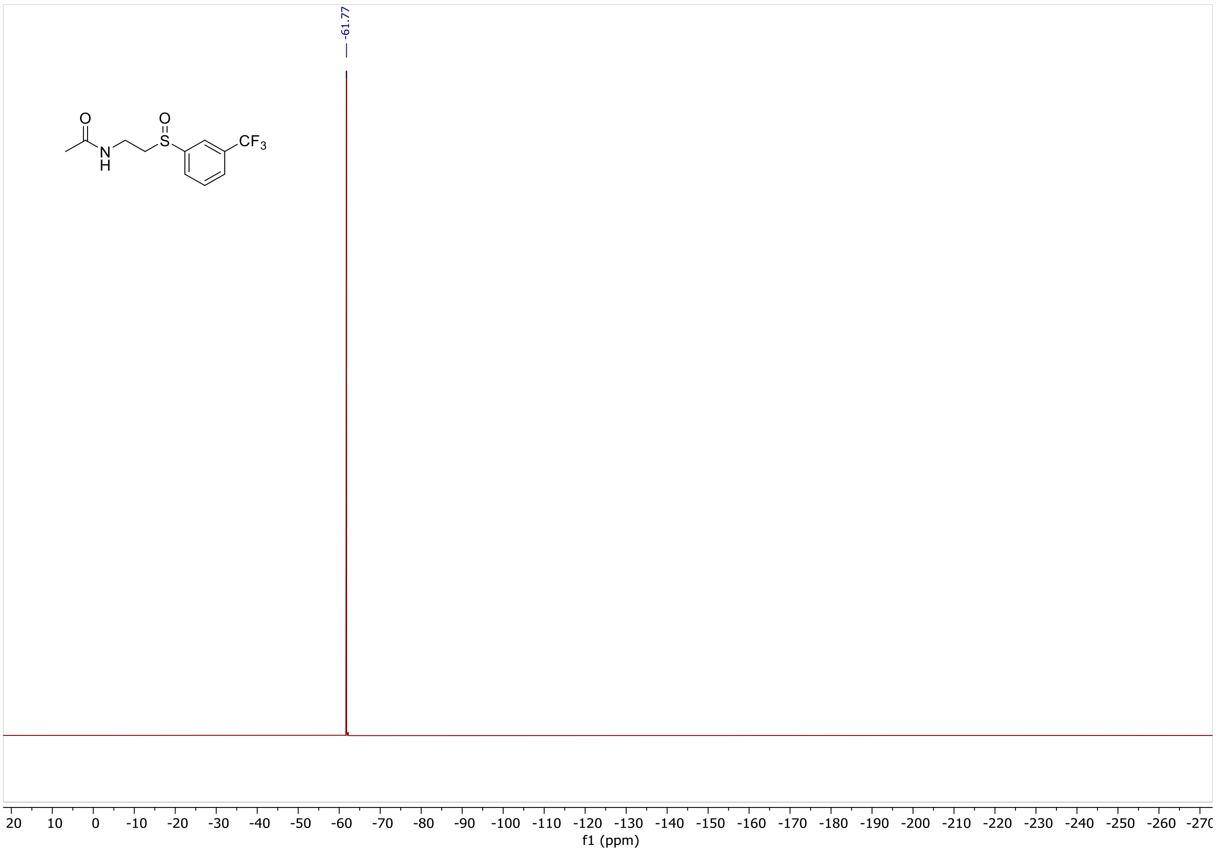

7.3 Starting Materials

***tert*-Butyl(3-((tert-butyldimethylsilyl)oxy)phenyl)dimethylsilane (1s)**

^{1}H-NMR in CDCl$_3$

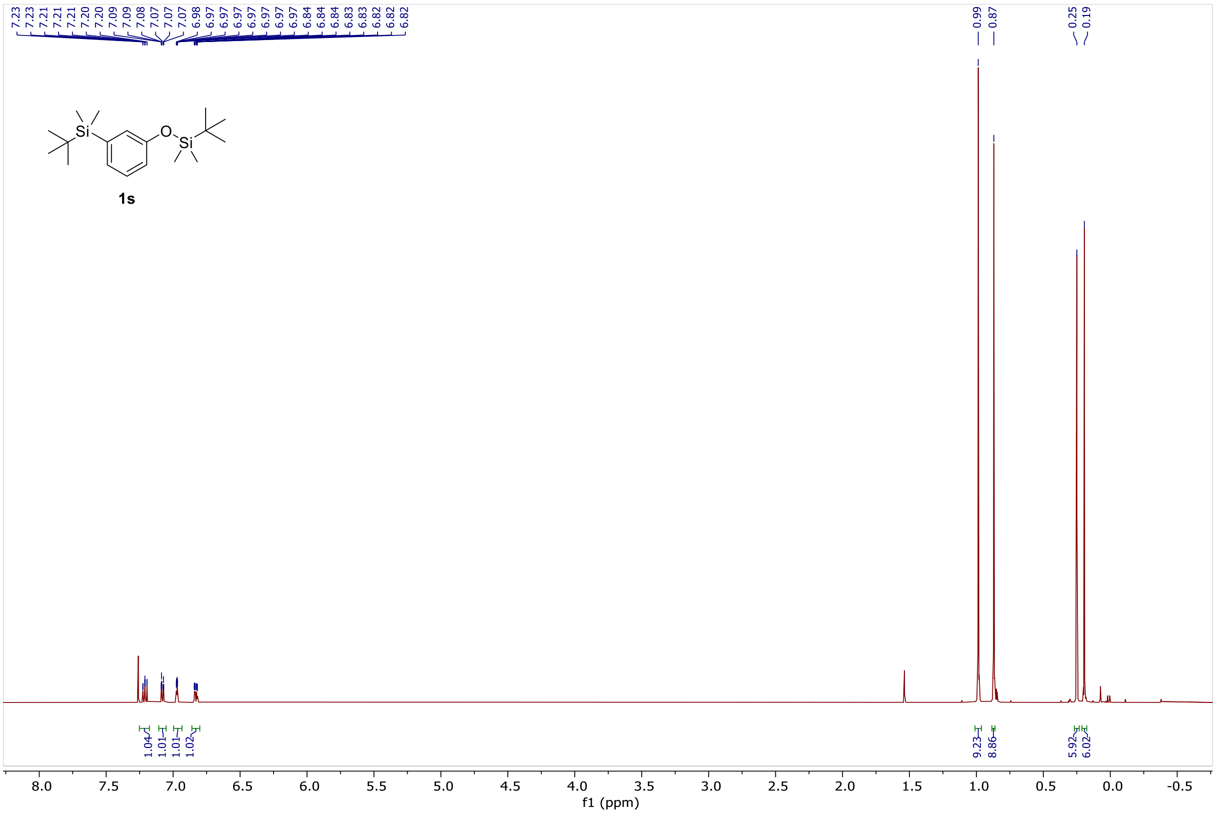

^{13}C-NMR in CDCl$_3$

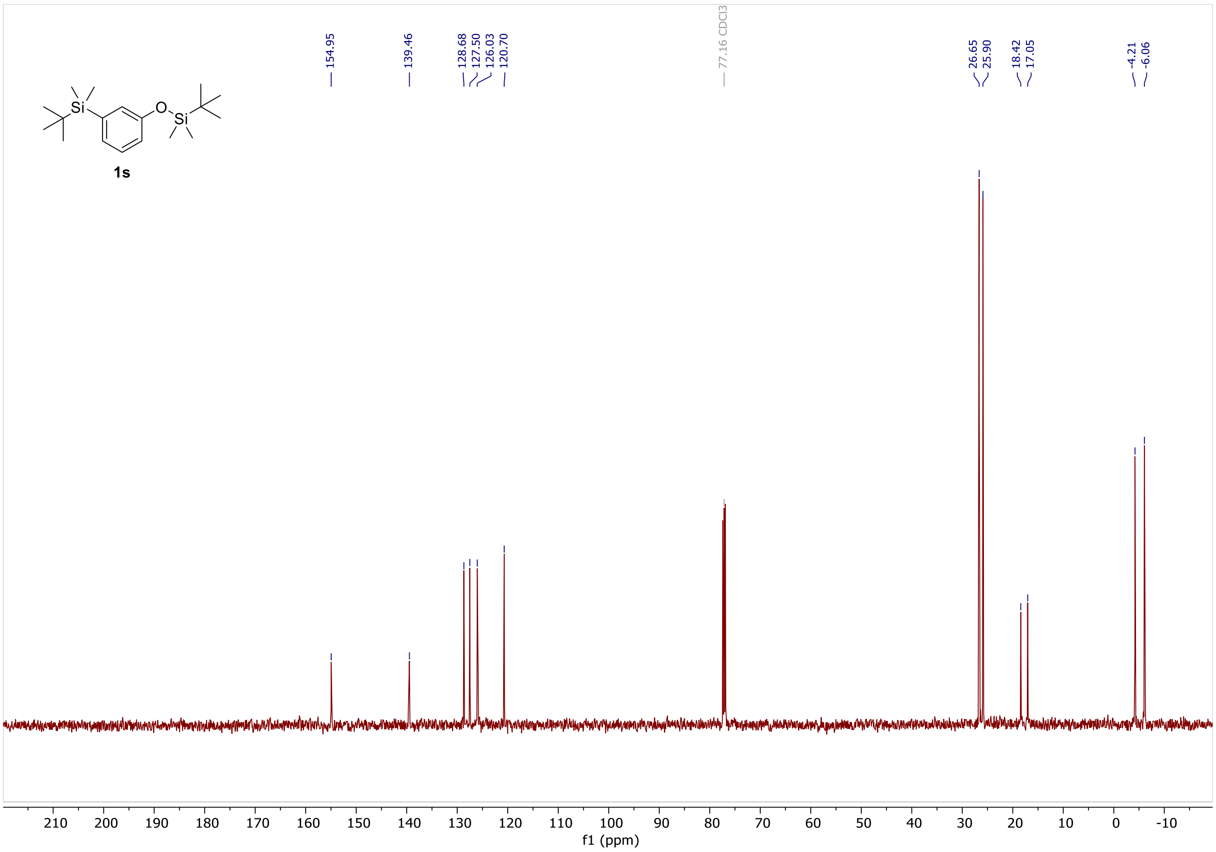

2-((2,3-Dimethylbenzyl)oxy)-1,3,3-trimethylbicyclo[2.2.1]heptane (1z)

^{1}H-NMR in $CDCl_3$

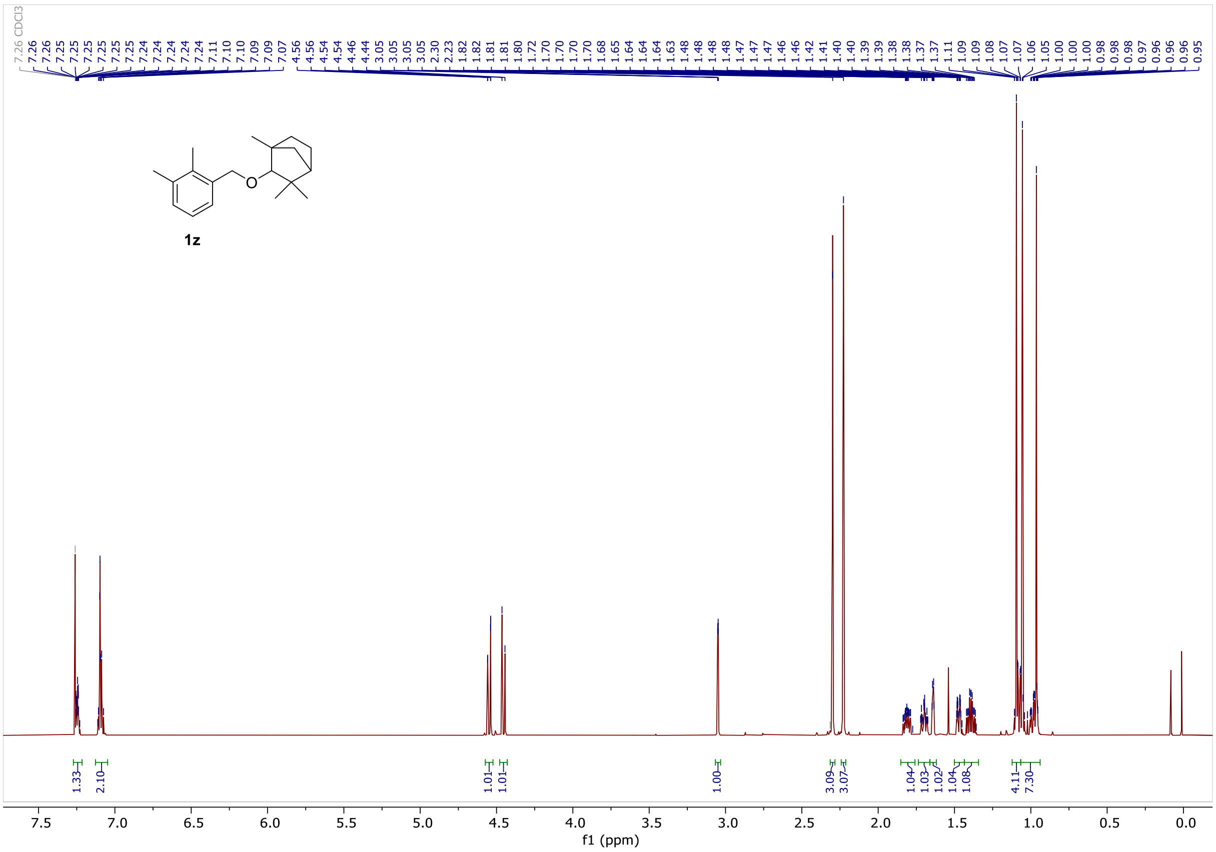

^{13}C-NMR in $CDCl_3$

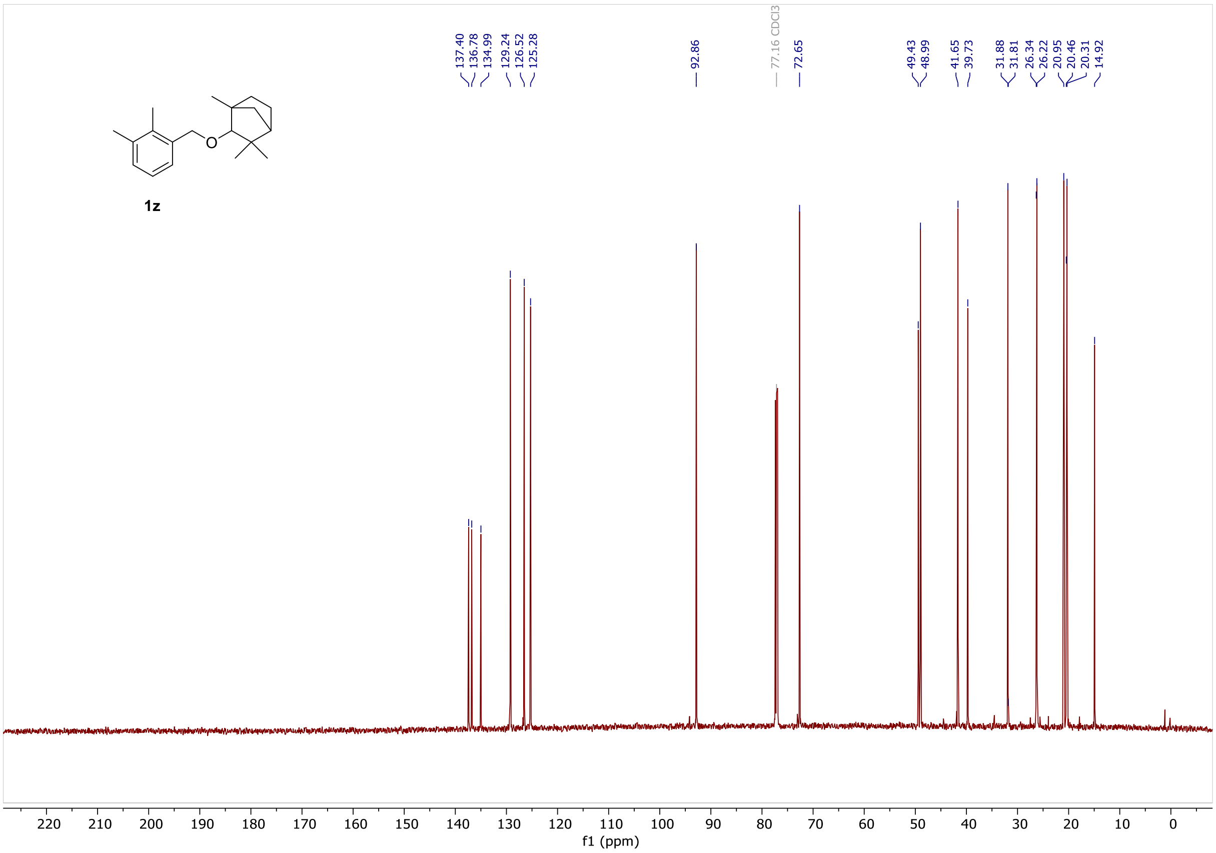

Methyl 2-methyl-4'-(trifluoromethoxy)-[1,1'-biphenyl]-3-carboxylate (1ac)

^{1}H-NMR in Acetone-d$_6$

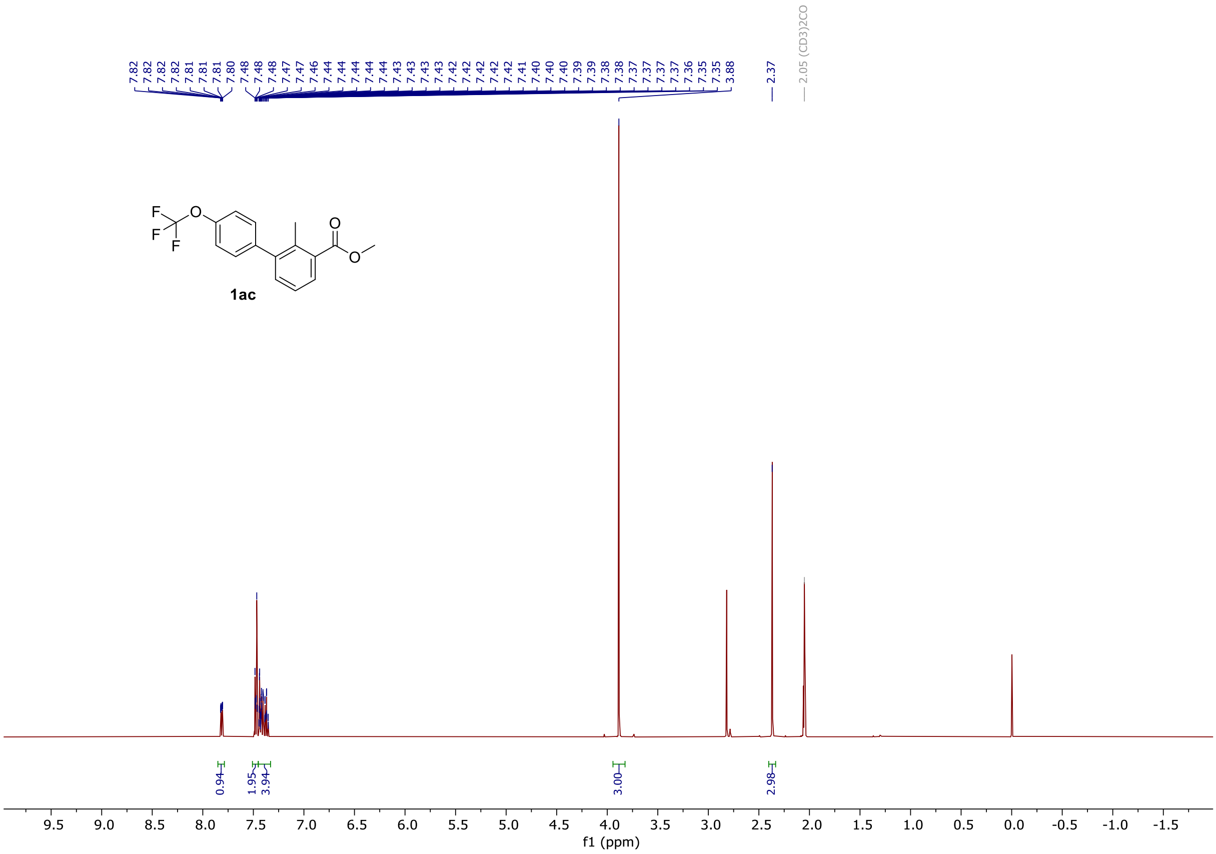

^{13}C-NMR in Acetone-d$_6$

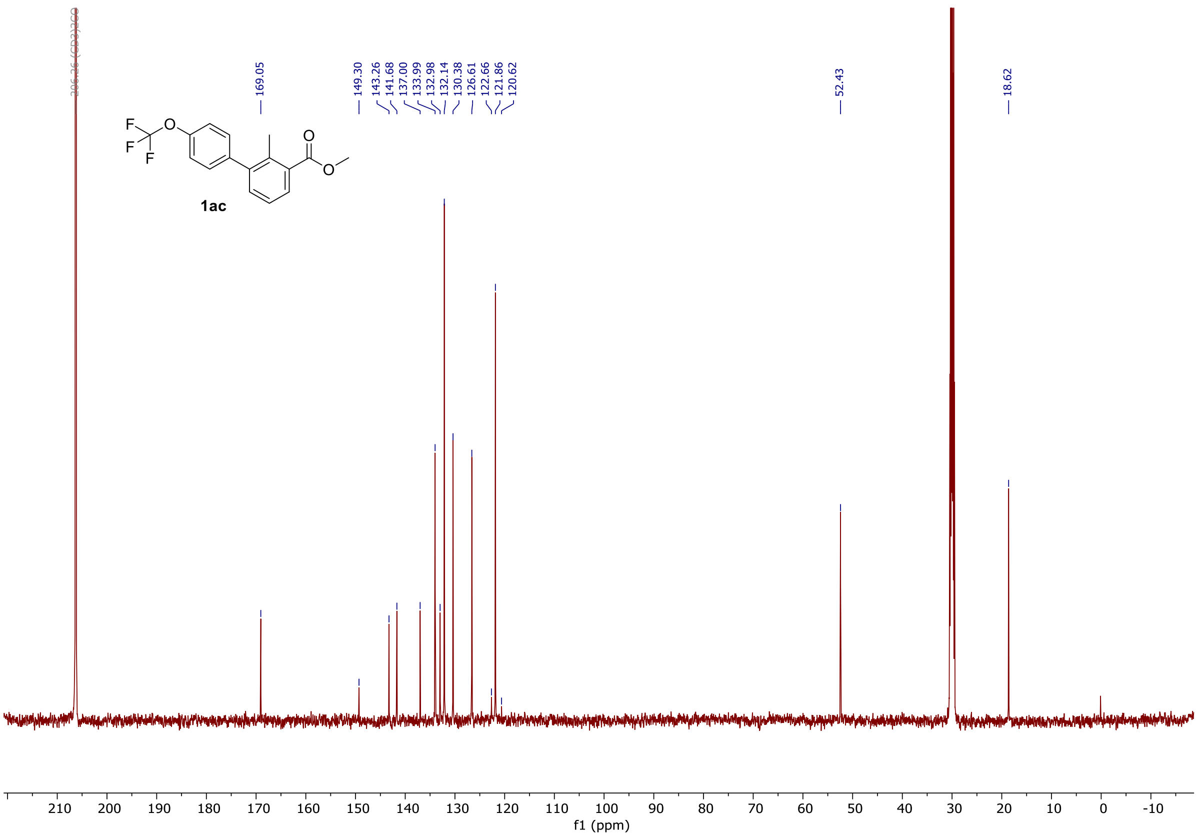

^{19}F-NMR in Acetone-d_6

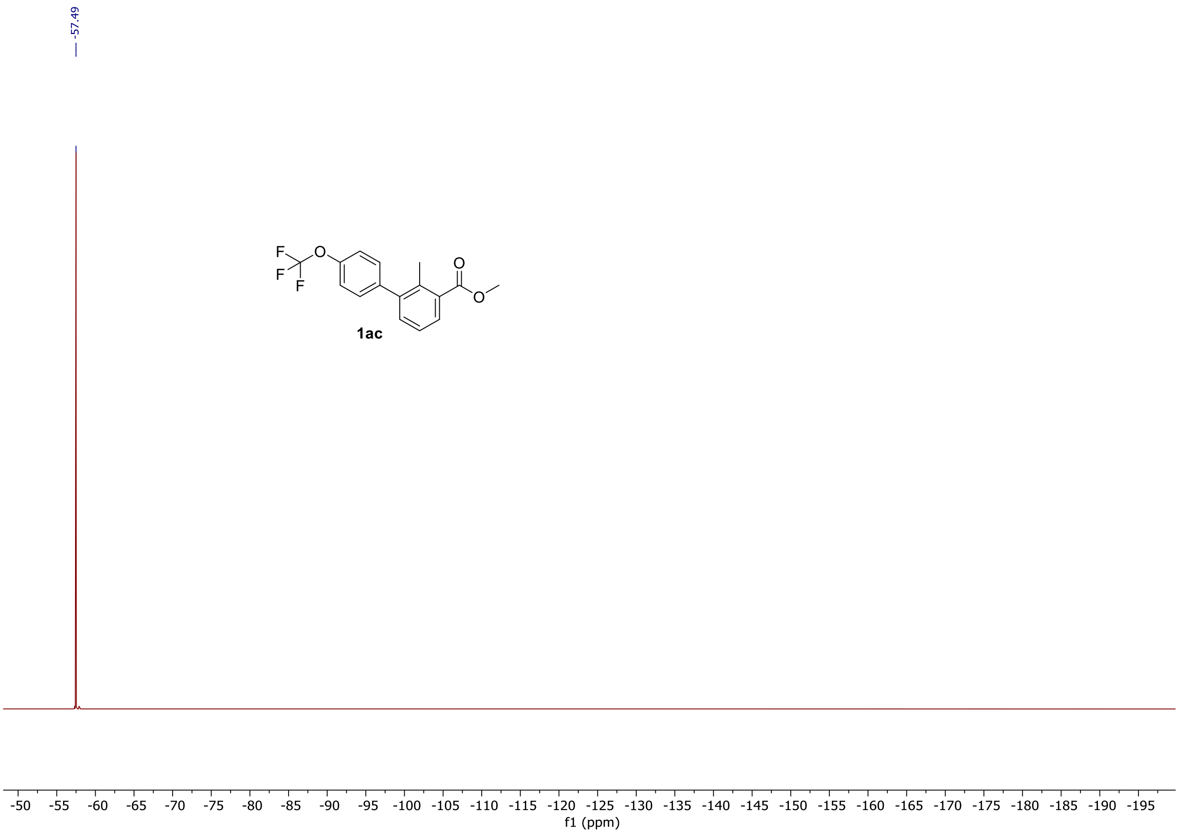

7.4 Scope shown in the main mansucript

Benzoic acid (4b)

^{1}H-NMR in $CDCl_3$

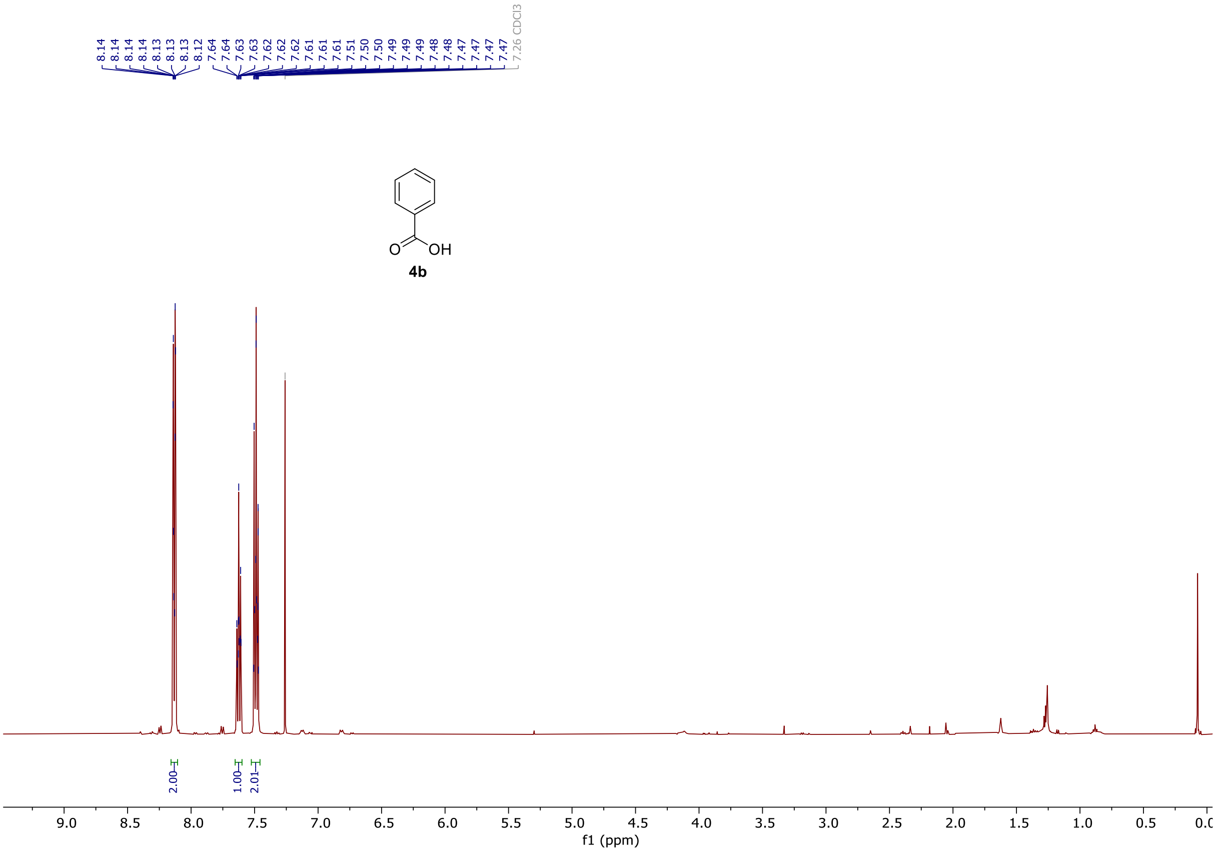

^{13}C-NMR in $CDCl_3$

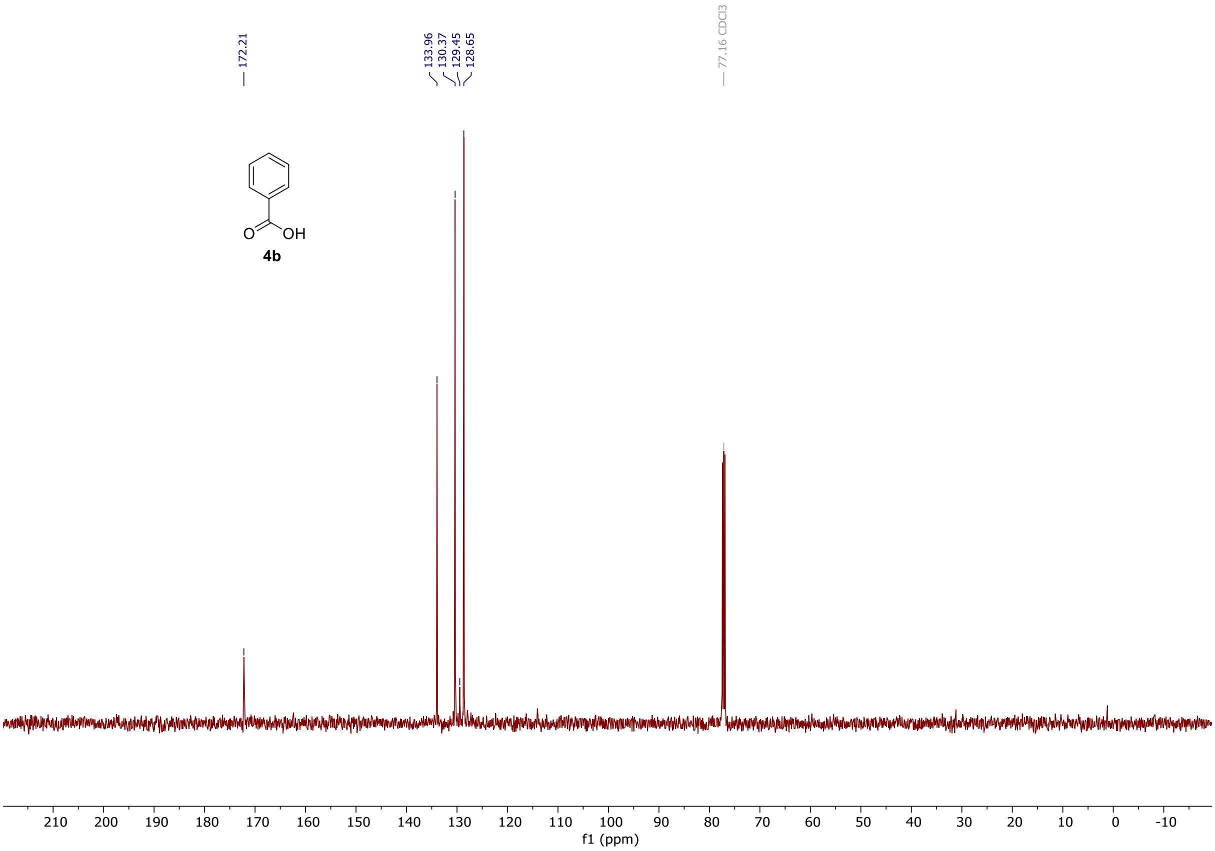

1,1,1,3,3,3-Hexafluoropropan-2-yl ethylbenzoate (2a)

^{1}H-NMR in $CDCl_3$

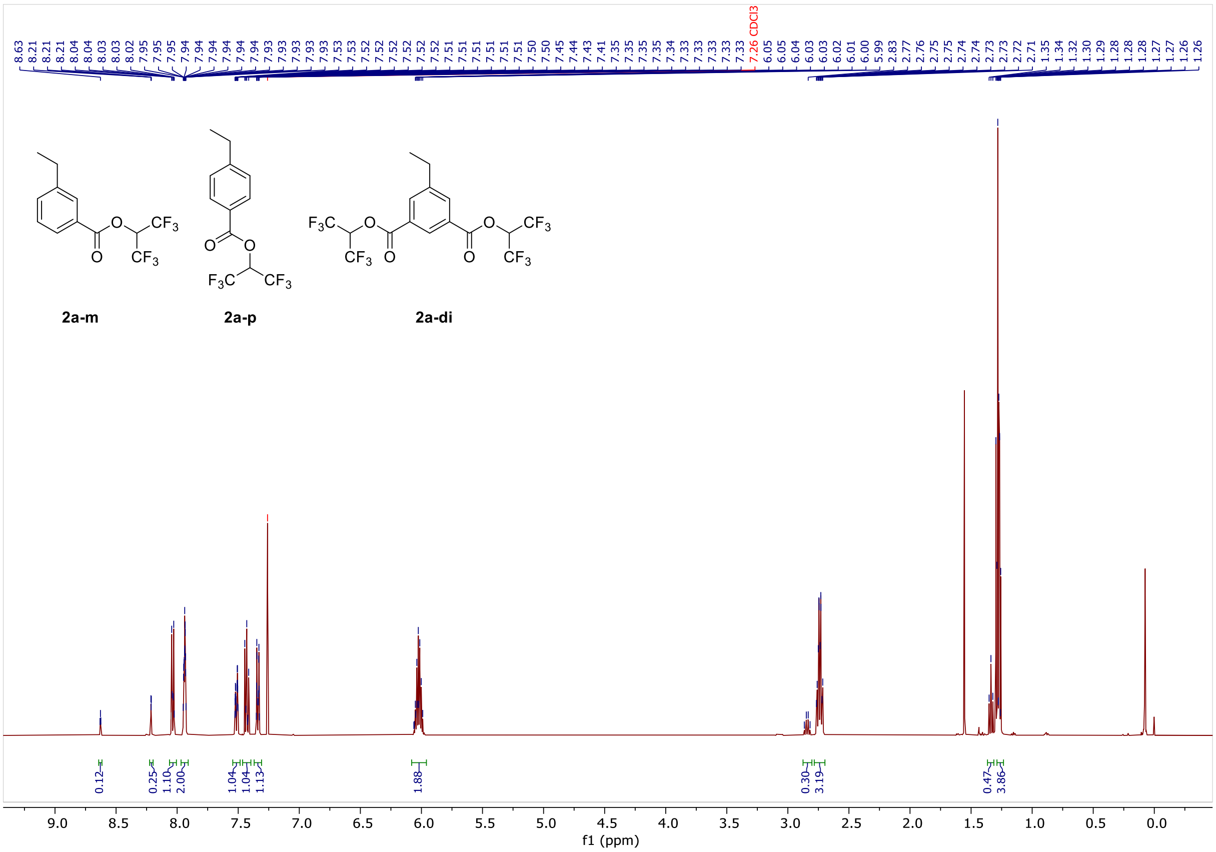

^{13}C-NMR in $CDCl_3$

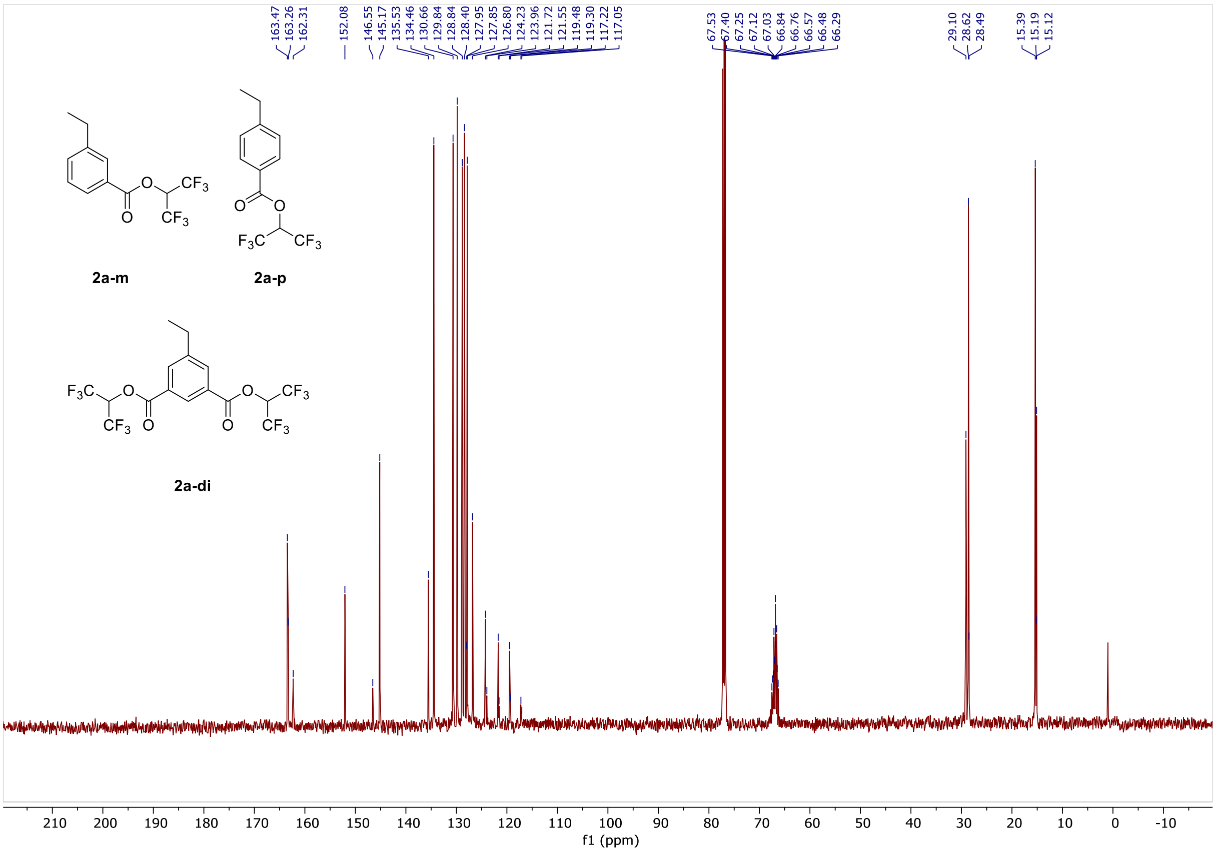

^{19}F-NMR in $CDCl_3$

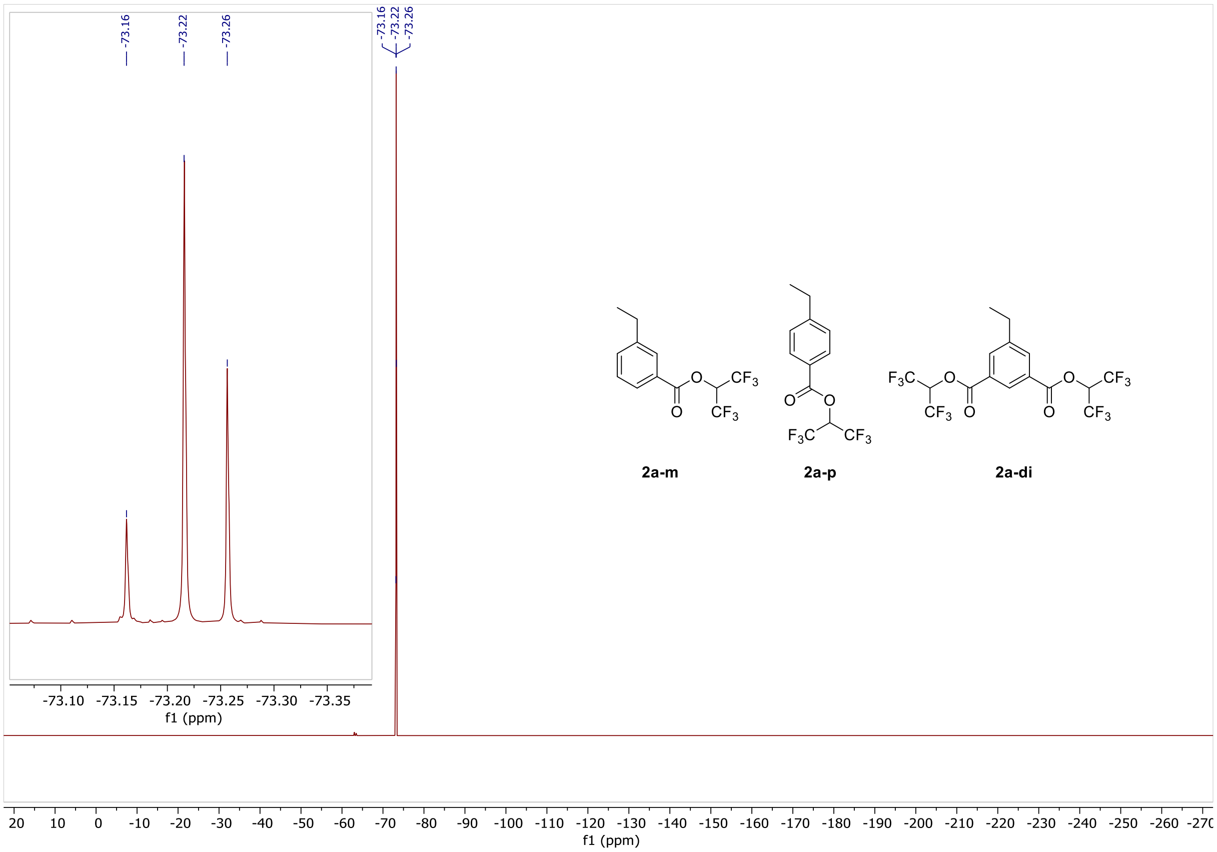

Chlorobenzoic acid (4c)

^{1}H-NMR in DMSO-d$_6$

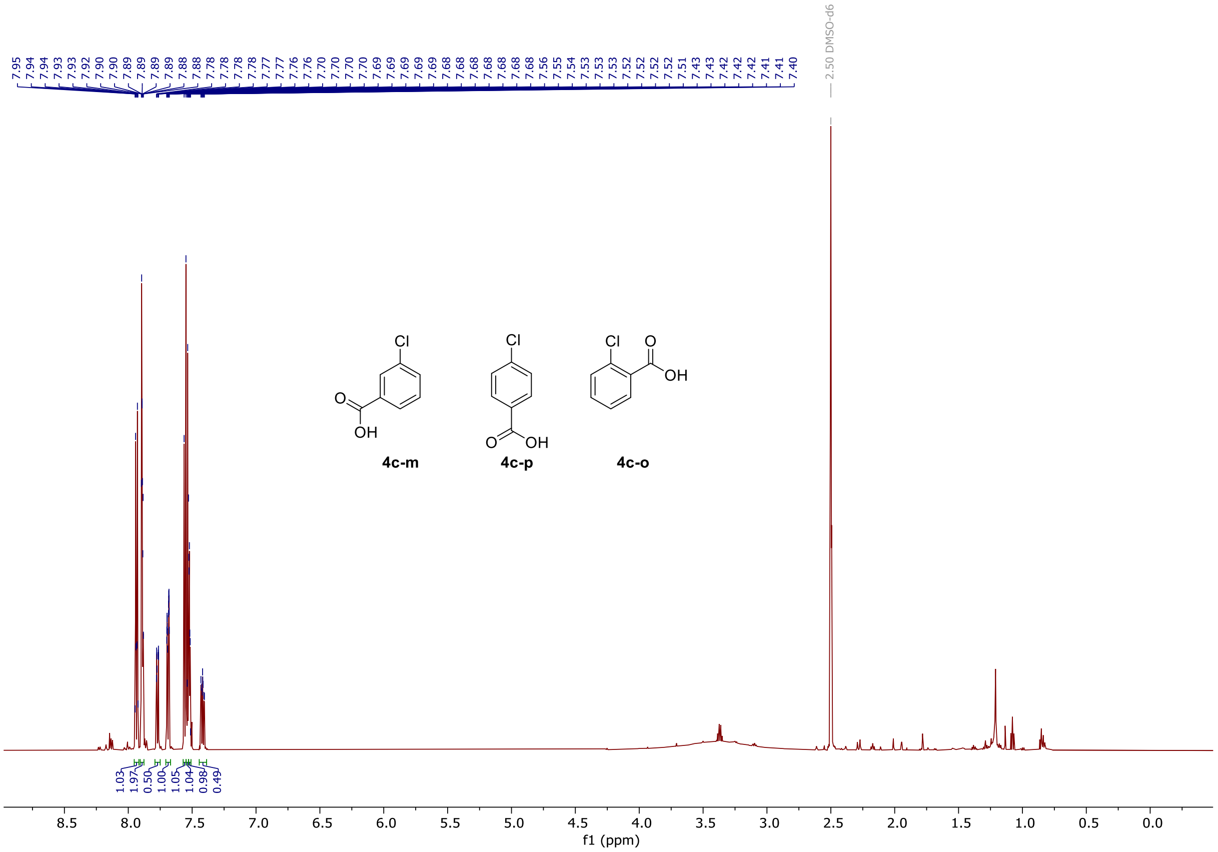

^{13}C-NMR in DMSO-d$_6$

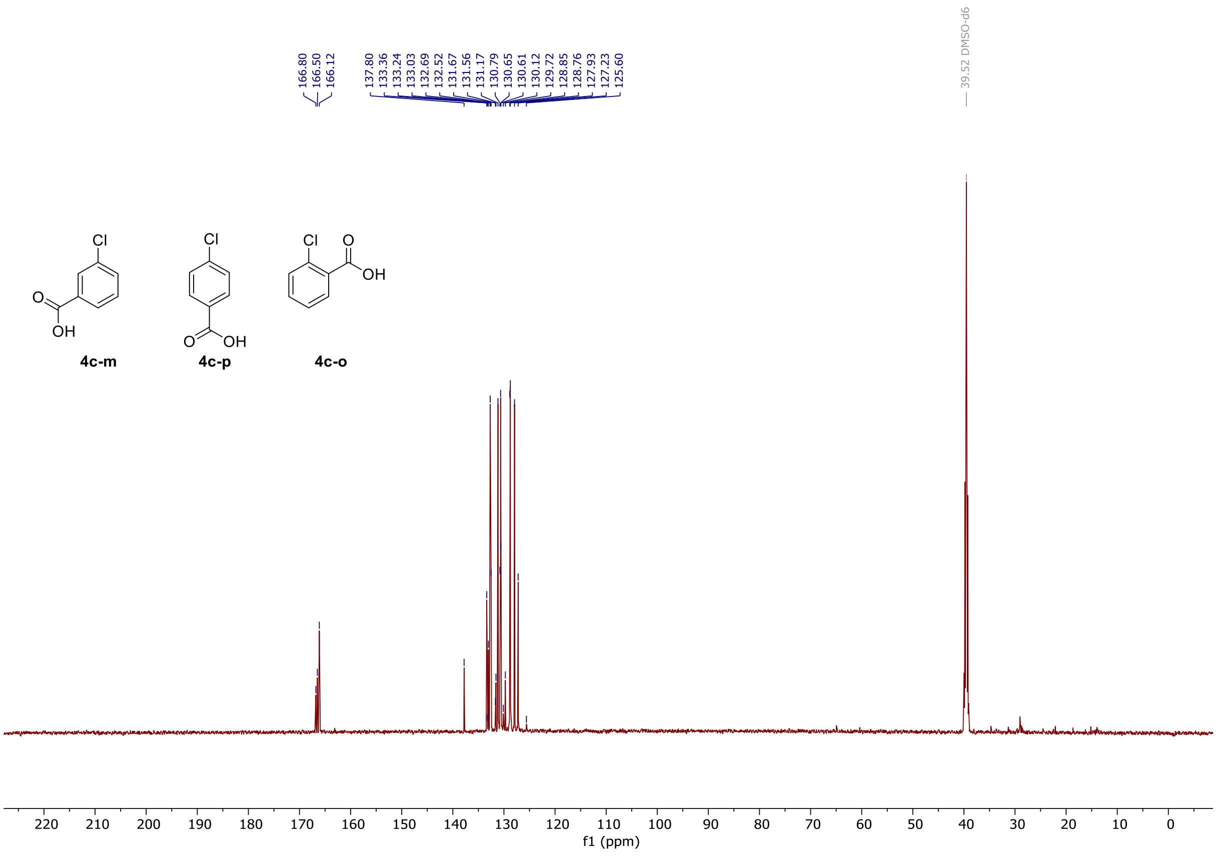

Bromobenzoic acid (4d)

^{1}H-NMR in $CDCl_3$

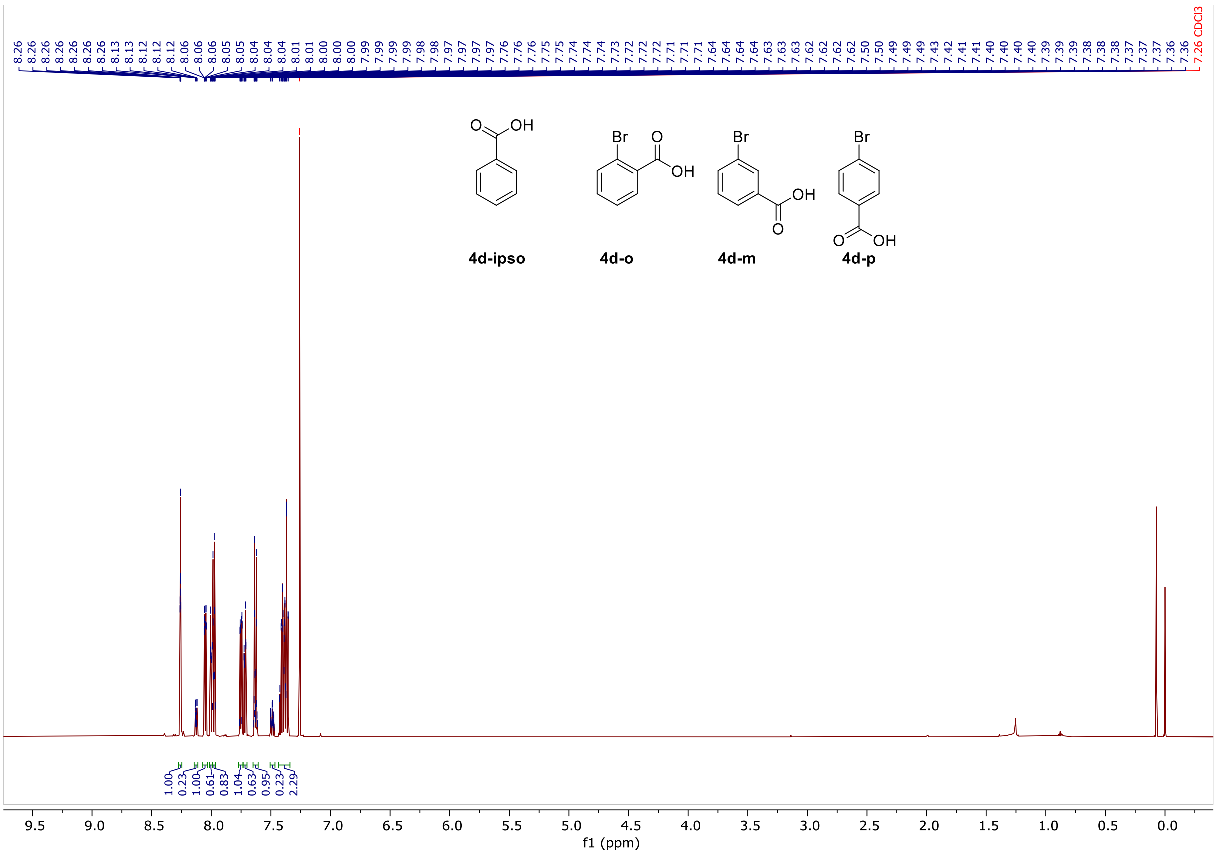

^{13}C-NMR in $CDCl_3$

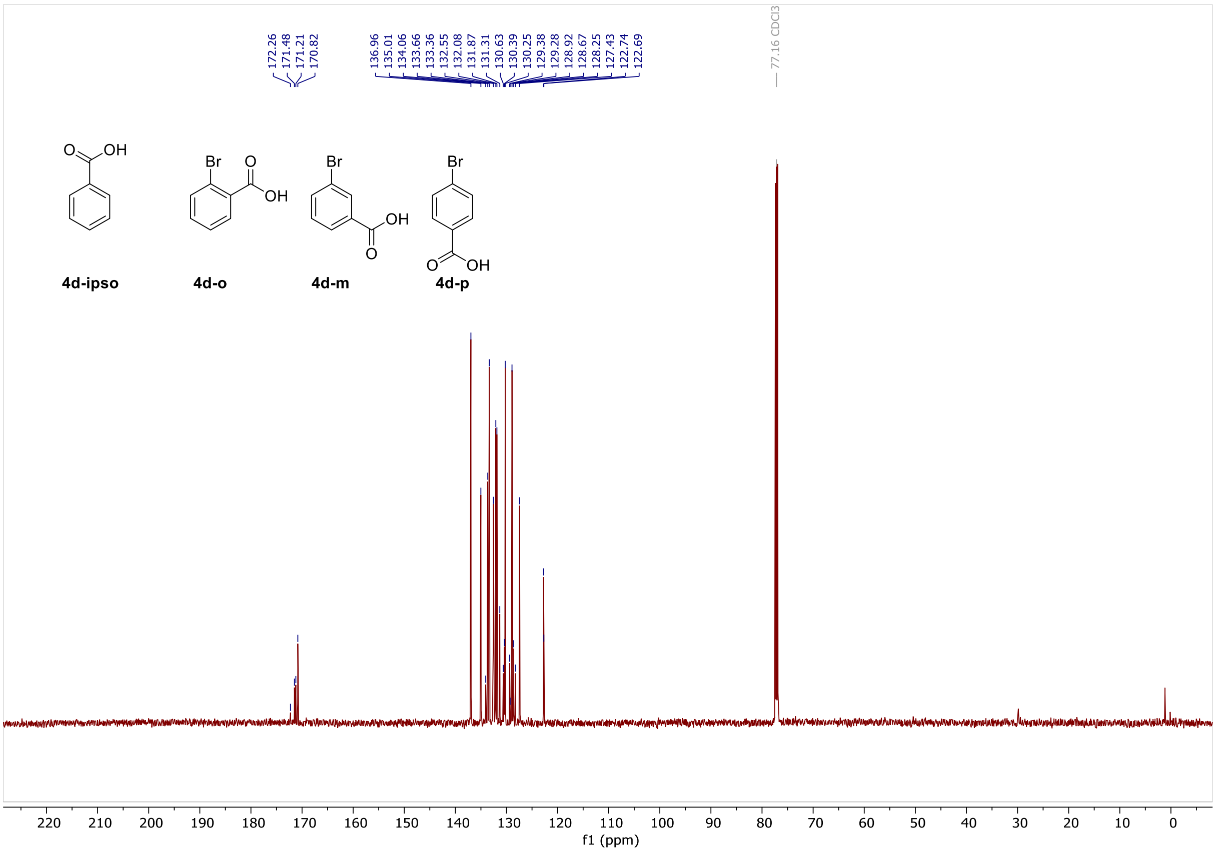

1,1,1,3,3,3-Hexafluoropropan-2-yl ((tert-butyldimethylsilyl)oxy)benzoate (2e)

^{1}H-NMR in $CDCl_3$

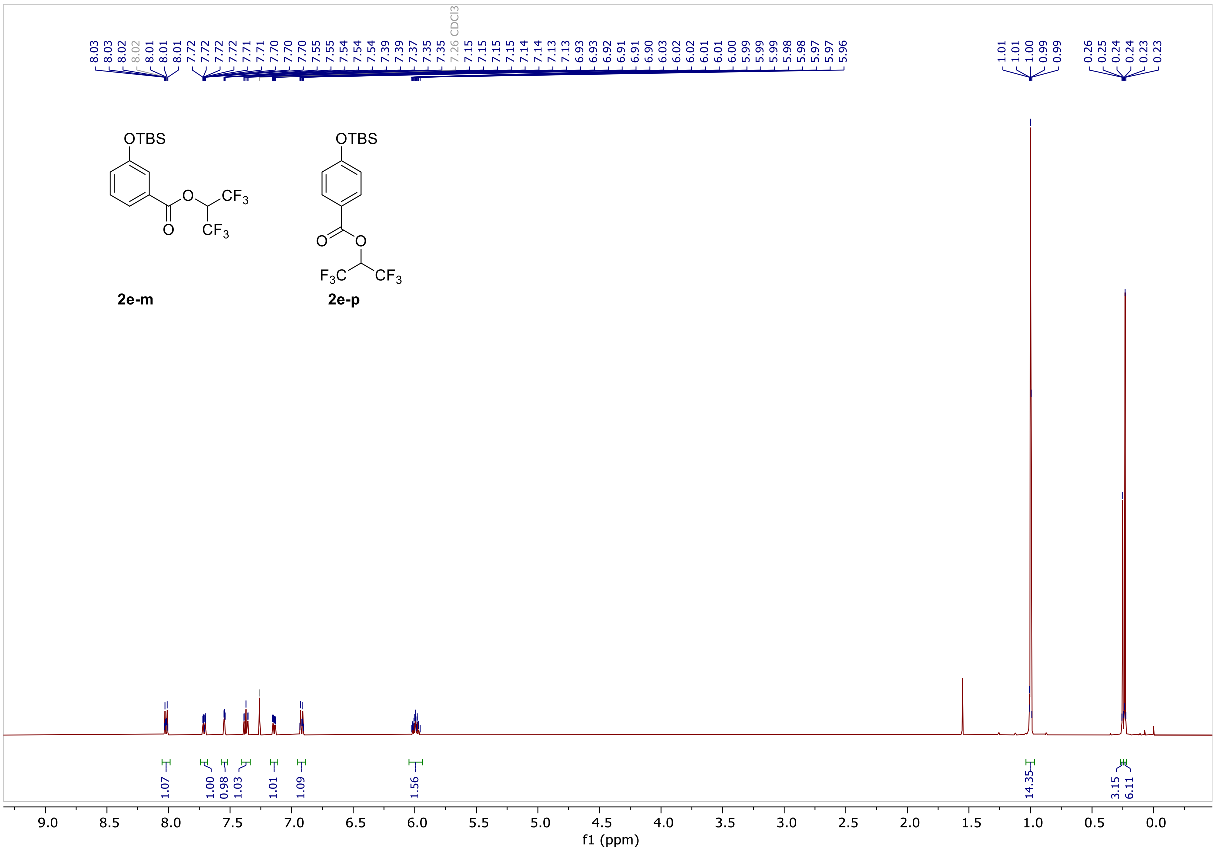

^{13}C-NMR in $CDCl_3$

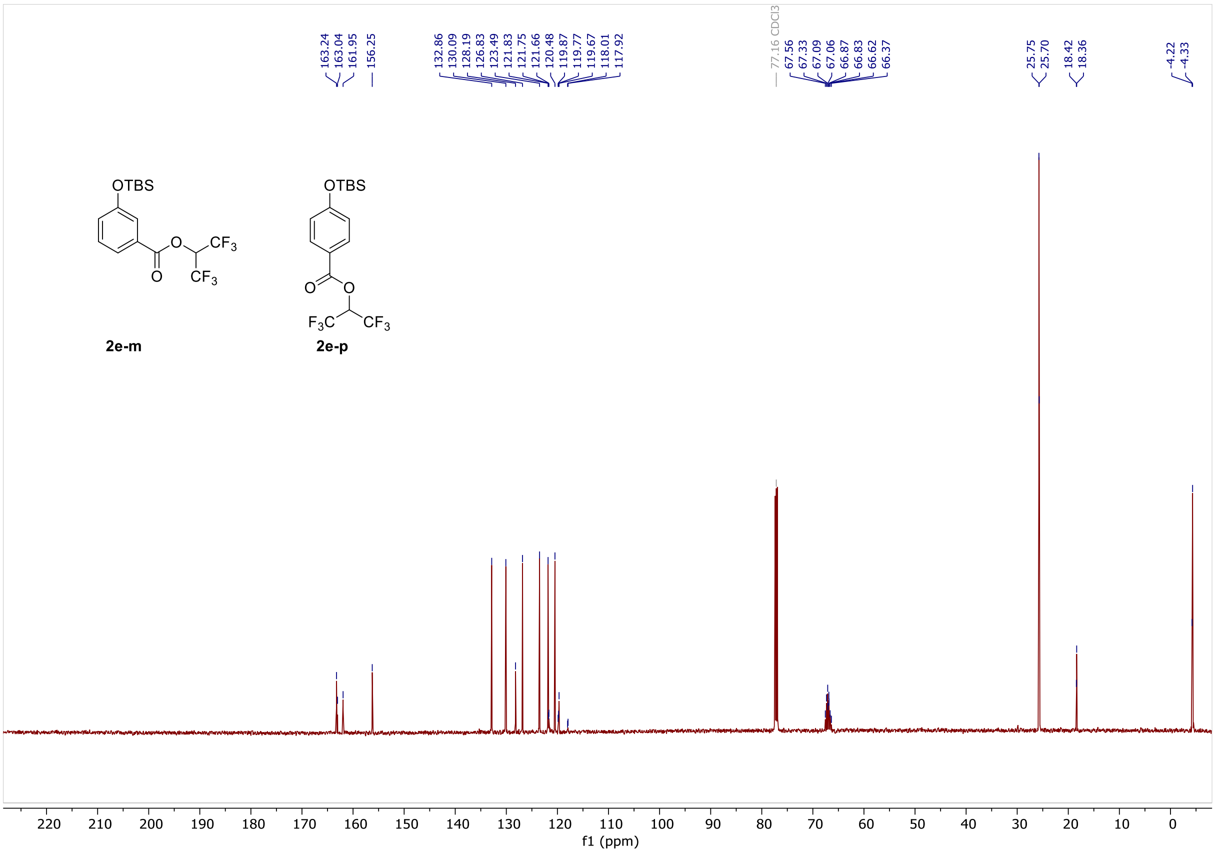

^{19}F-NMR in $CDCl_3$

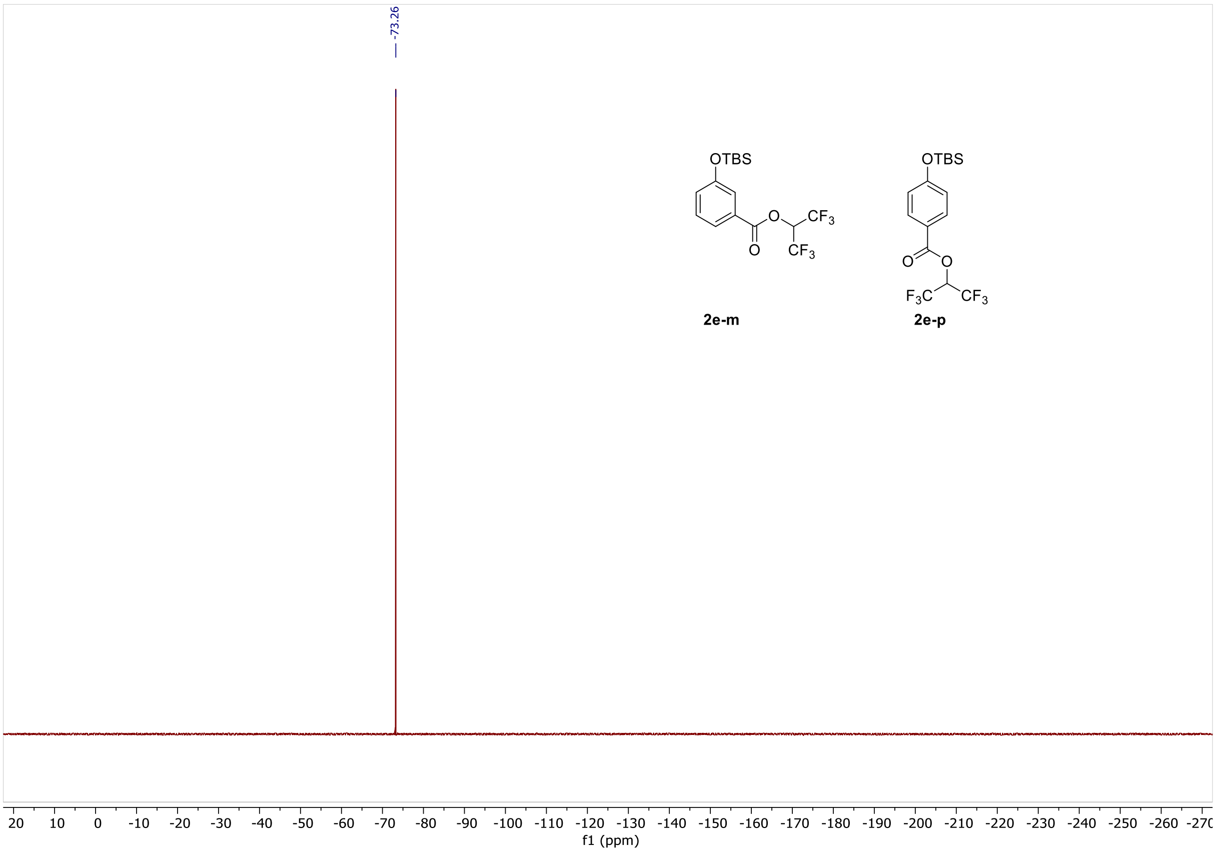

1,1,1,3,3,3-hexafluoropropan-2-yl 3-(trimethylsilyl)benzoate (2f)

^{1}H-NMR in $CDCl_3$

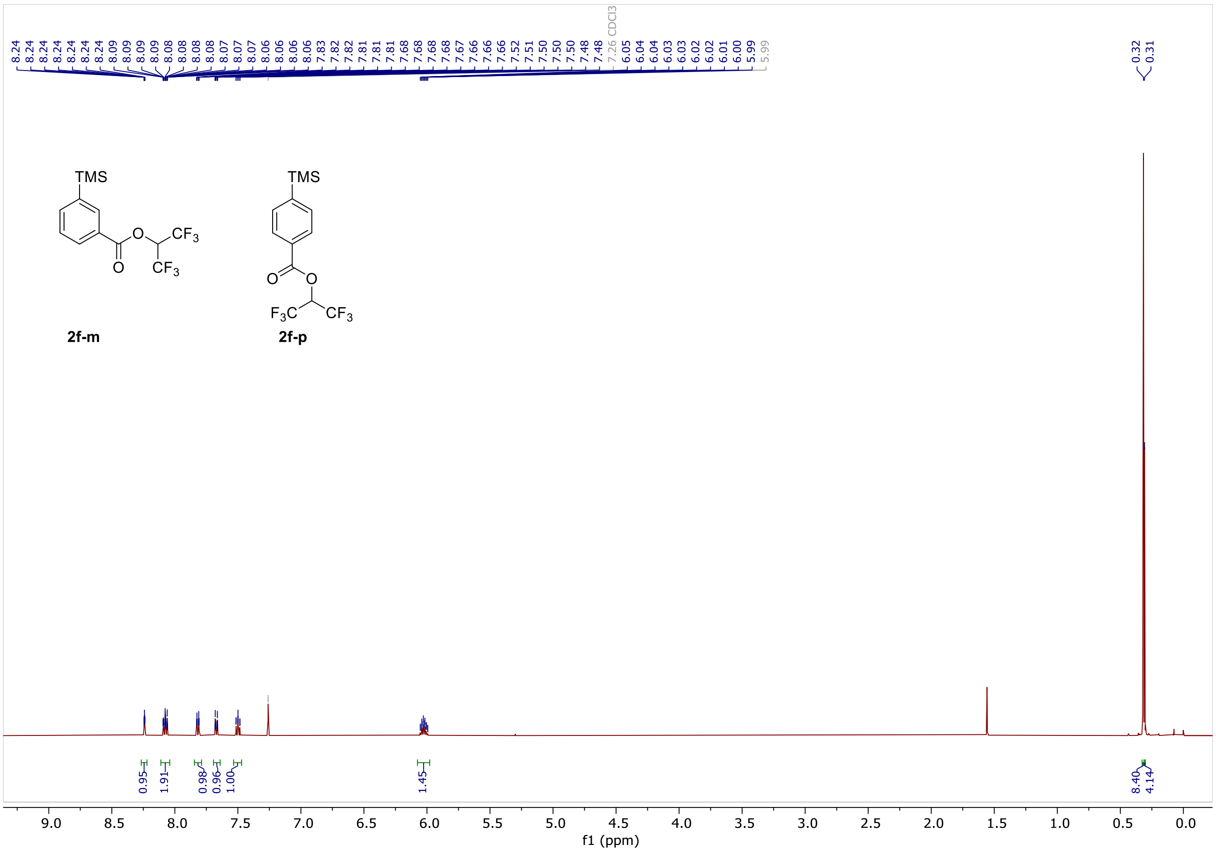

^{13}C-NMR in $CDCl_3$

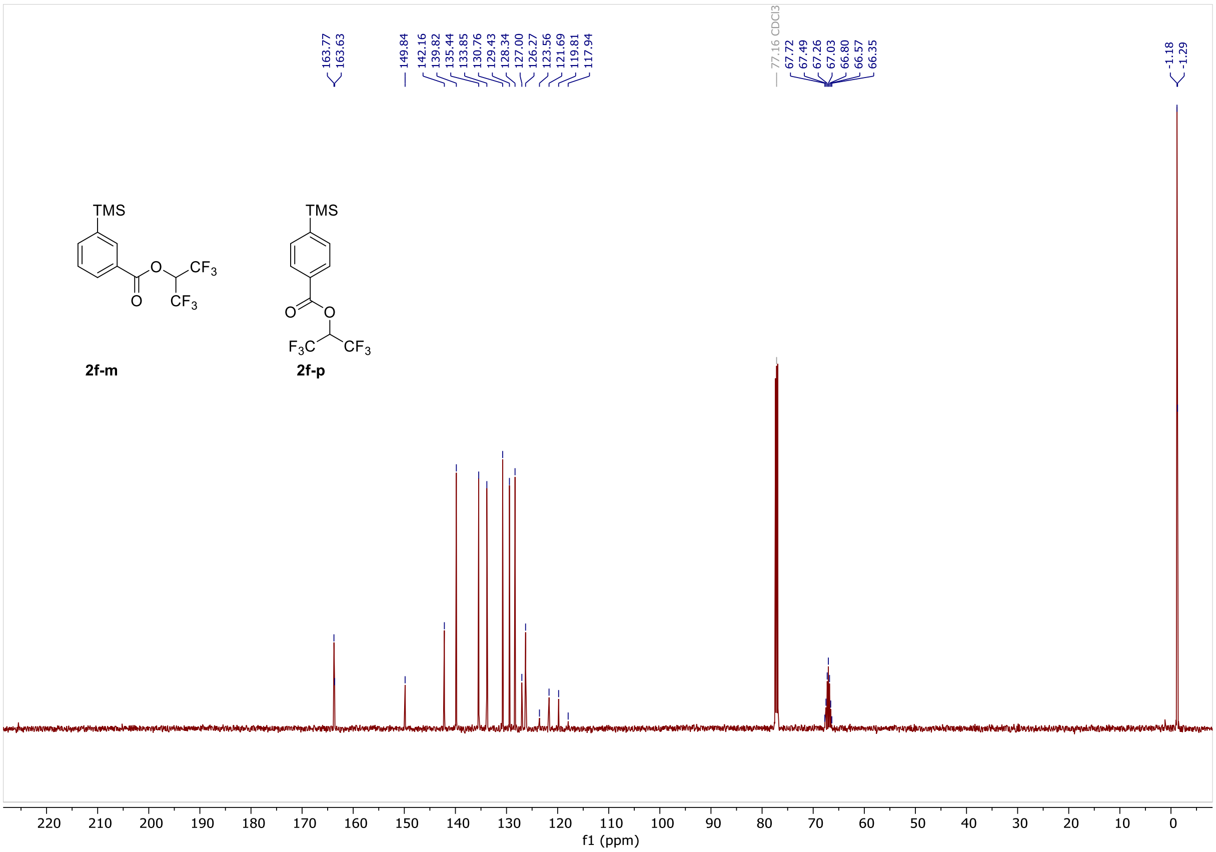

^{19}F-NMR in $CDCl_3$

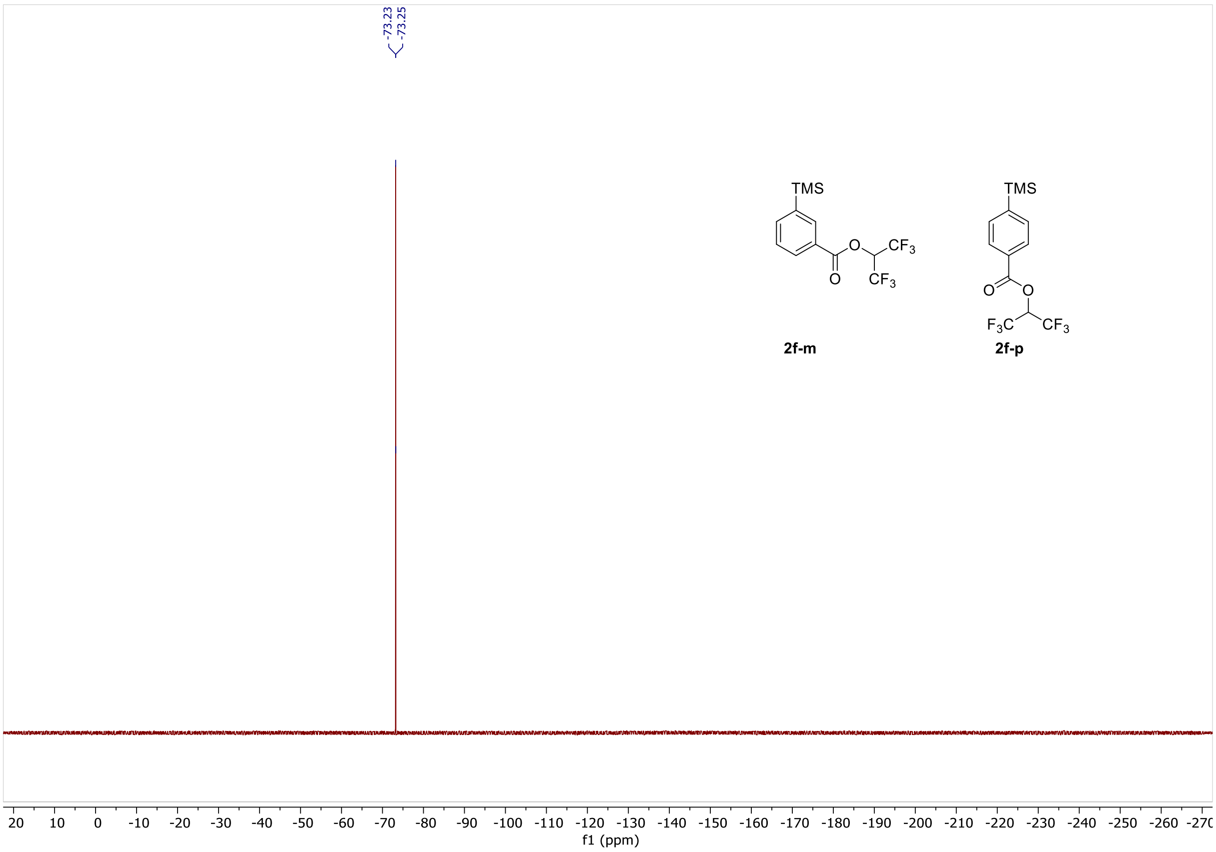

2',3',4',5',6'-Pentafluoro-[1,1'-biphenyl]-carboxylic acid (4g)

^{1}H-NMR in Acetone-d$_6$

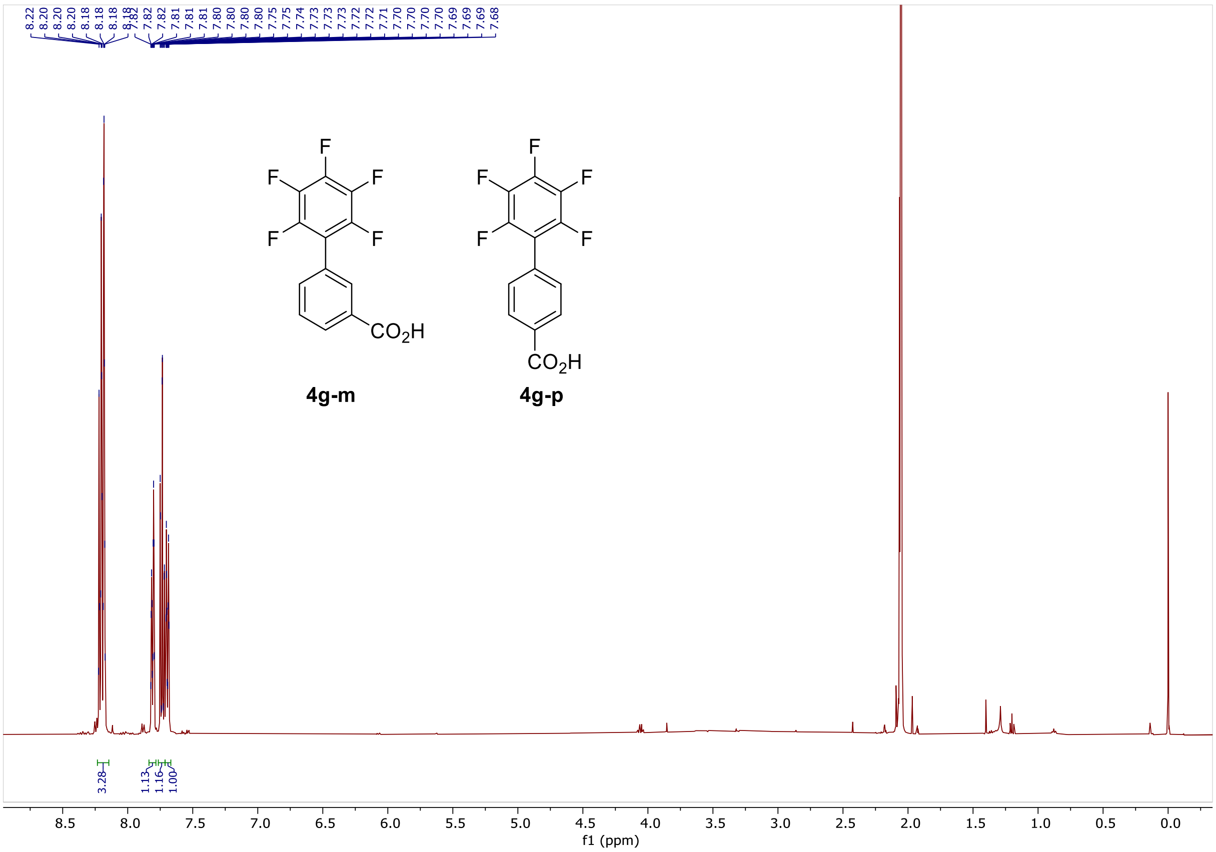

^{13}C-NMR in Acetone-d$_6$

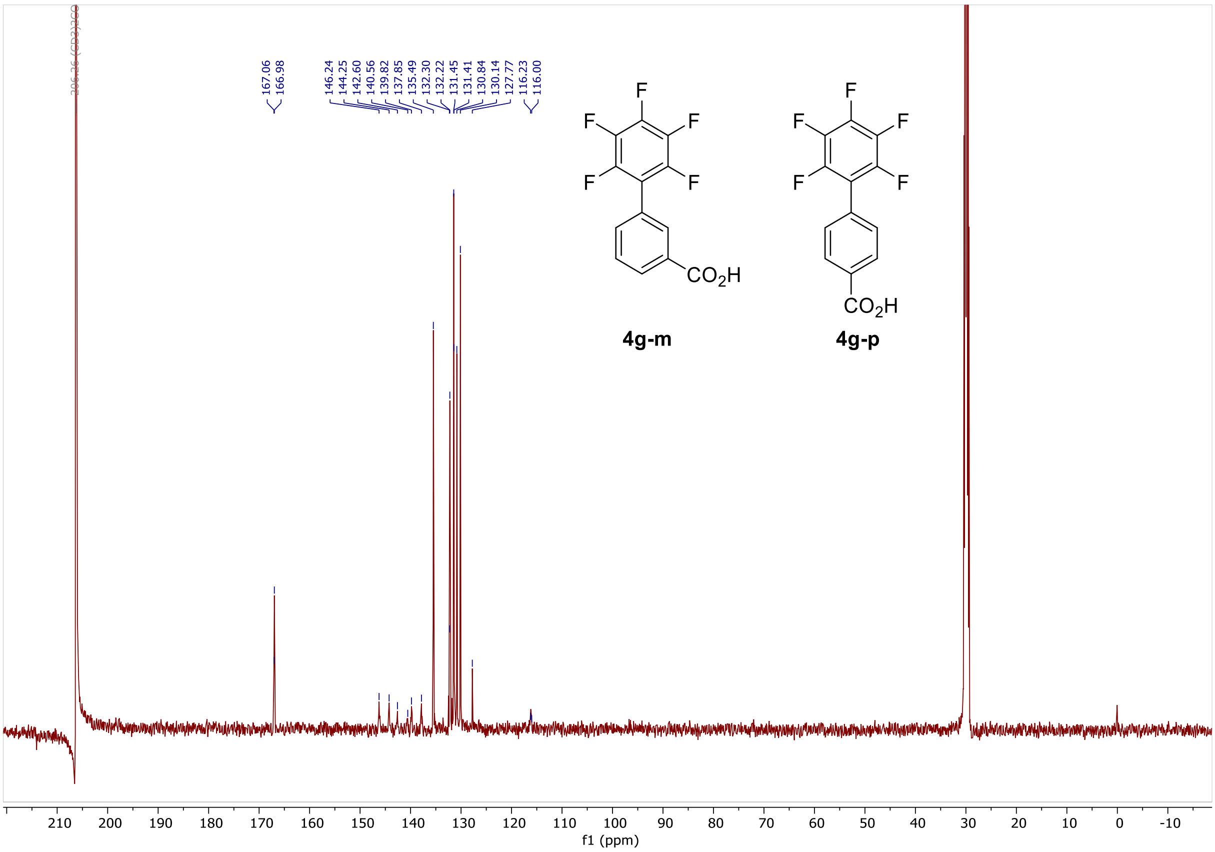

^{19}F-NMR in Acetone-d$_6$

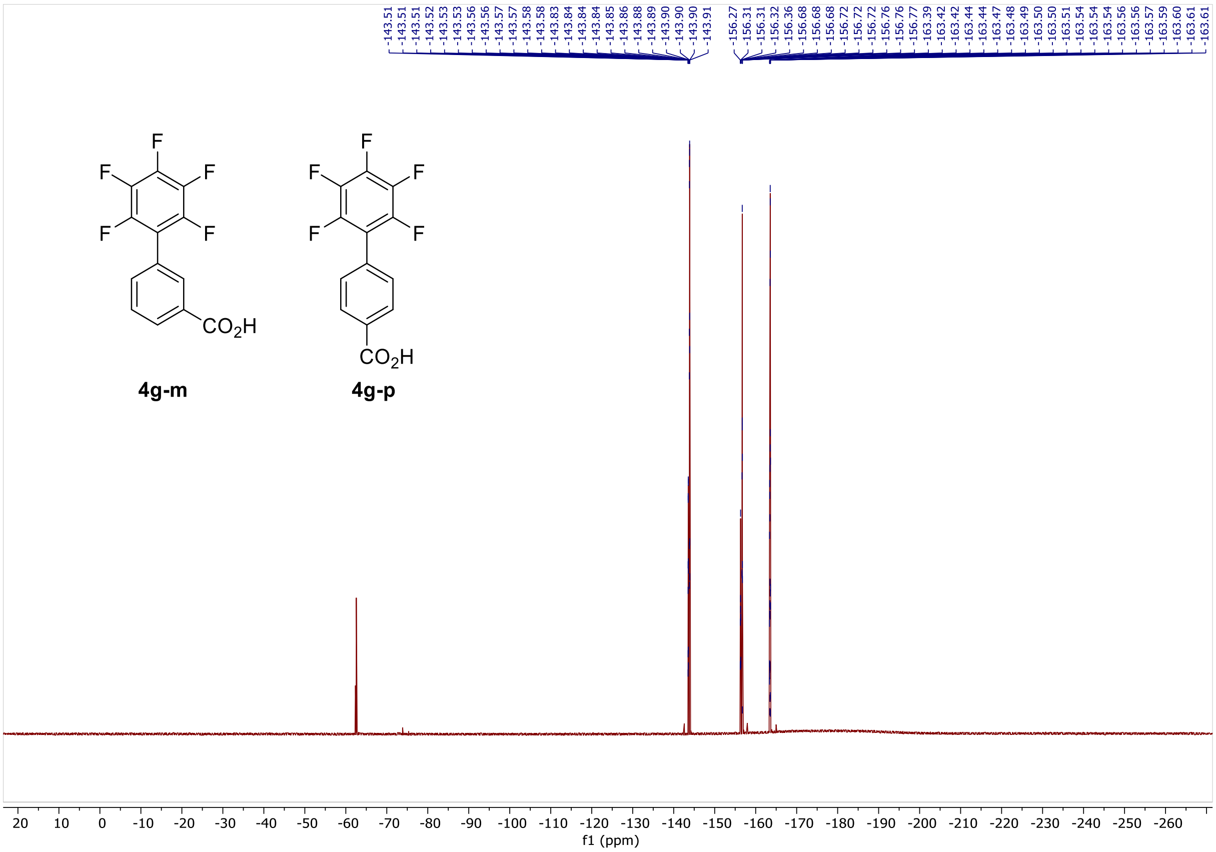

Pivaloylbenzoic acid (4h)

^{1}H-NMR in $CDCl_3$

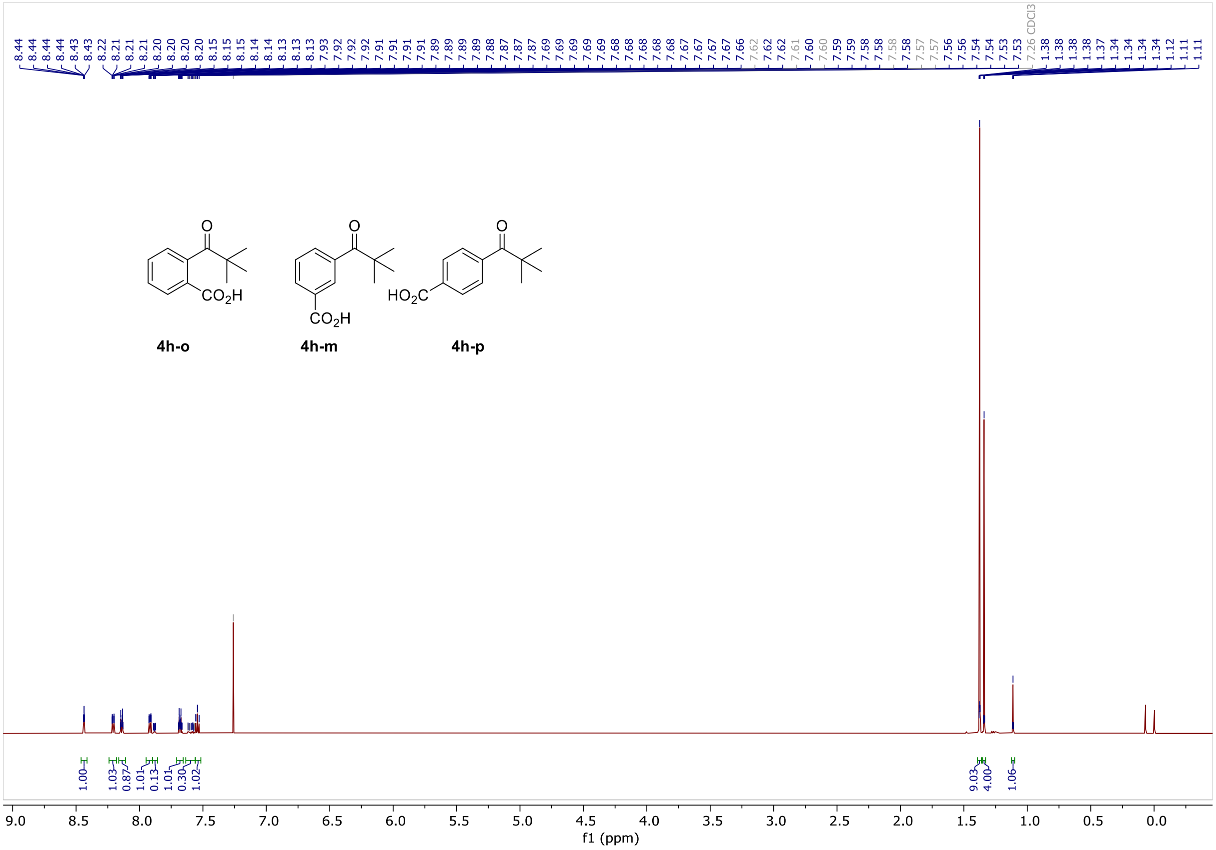

^{13}C-NMR in $CDCl_3$

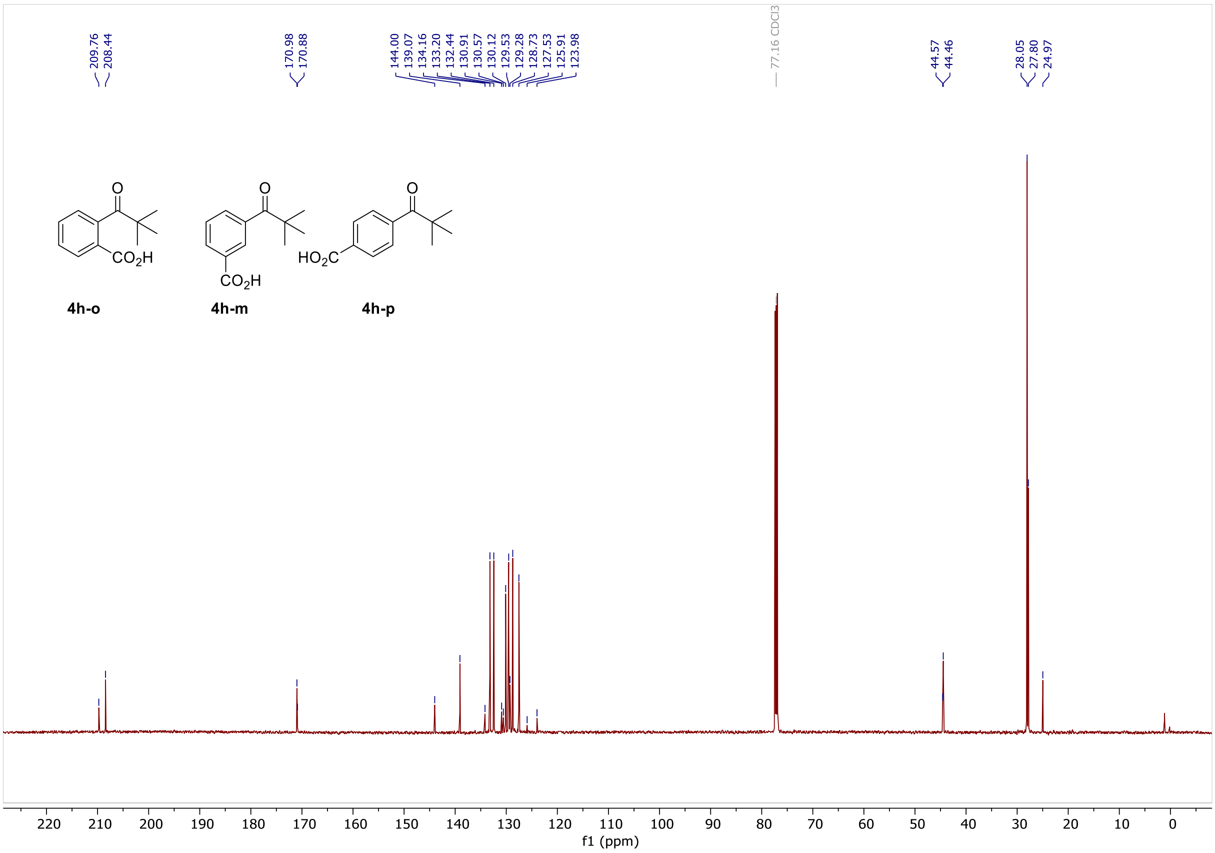

2-(Diisopropylcarbamoyl)benzoic acid (4i)

^{1}H-NMR in DMSO-d$_6$

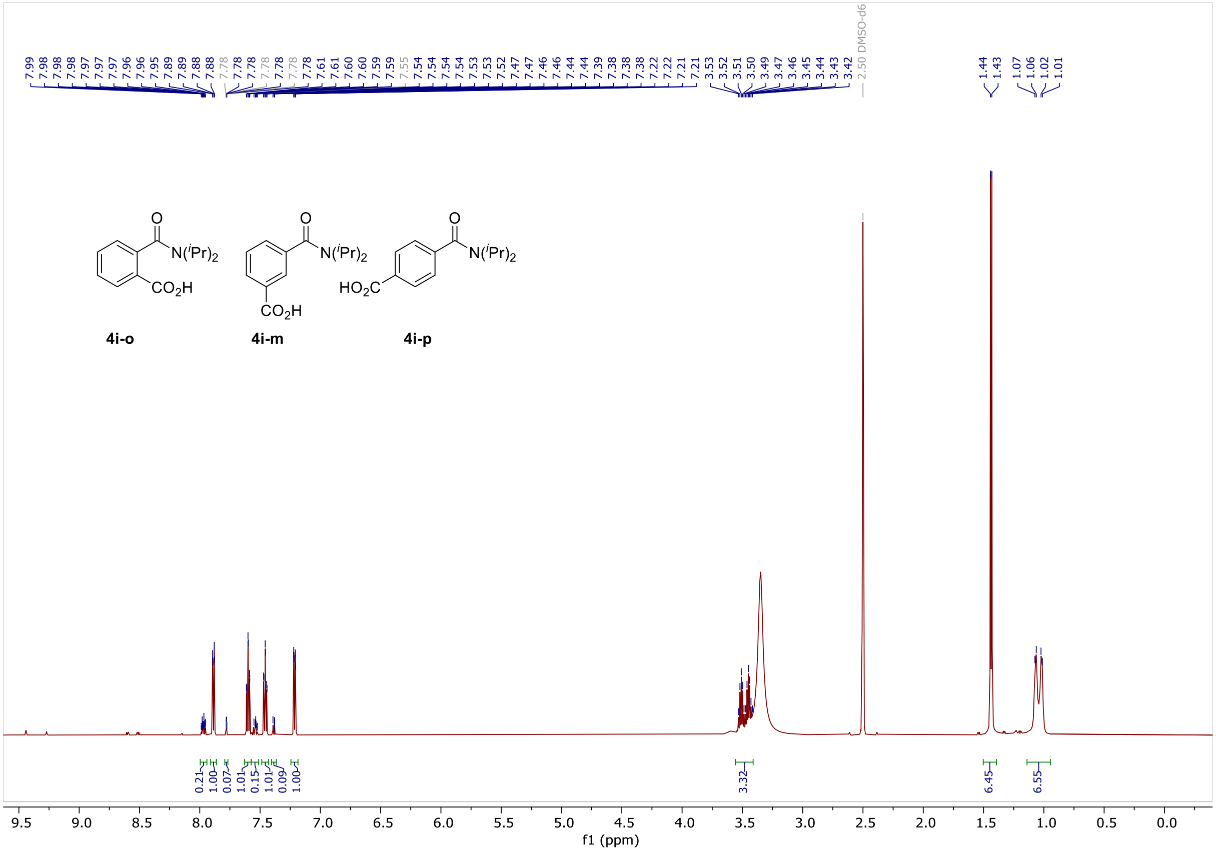

^{13}C-NMR in DMSO-d$_6$

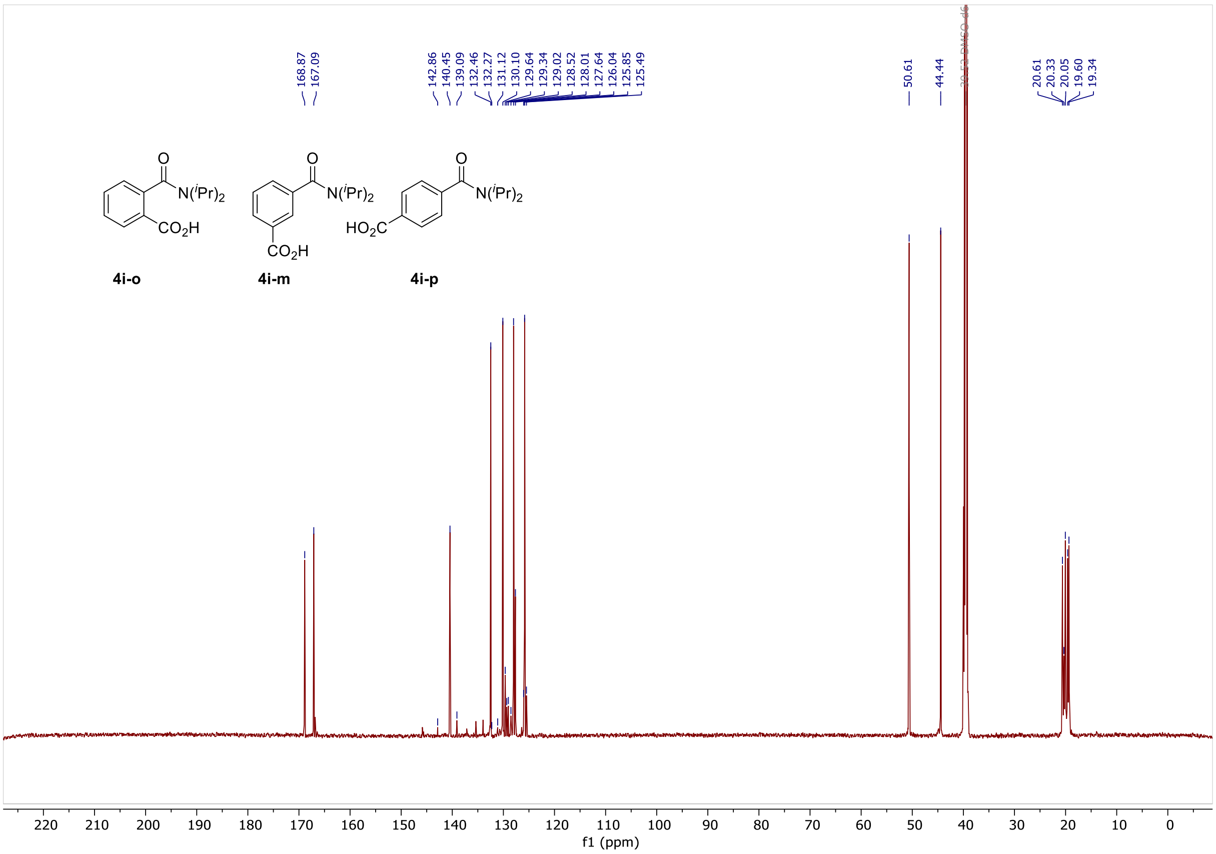

(1,1,1,3,3,3-Hexafluoropropan-2-yl) 4-methyl 1-methyl-1H-pyrrole-dicarboxylate (2j)

^{1}H-NMR in $CDCl_3$

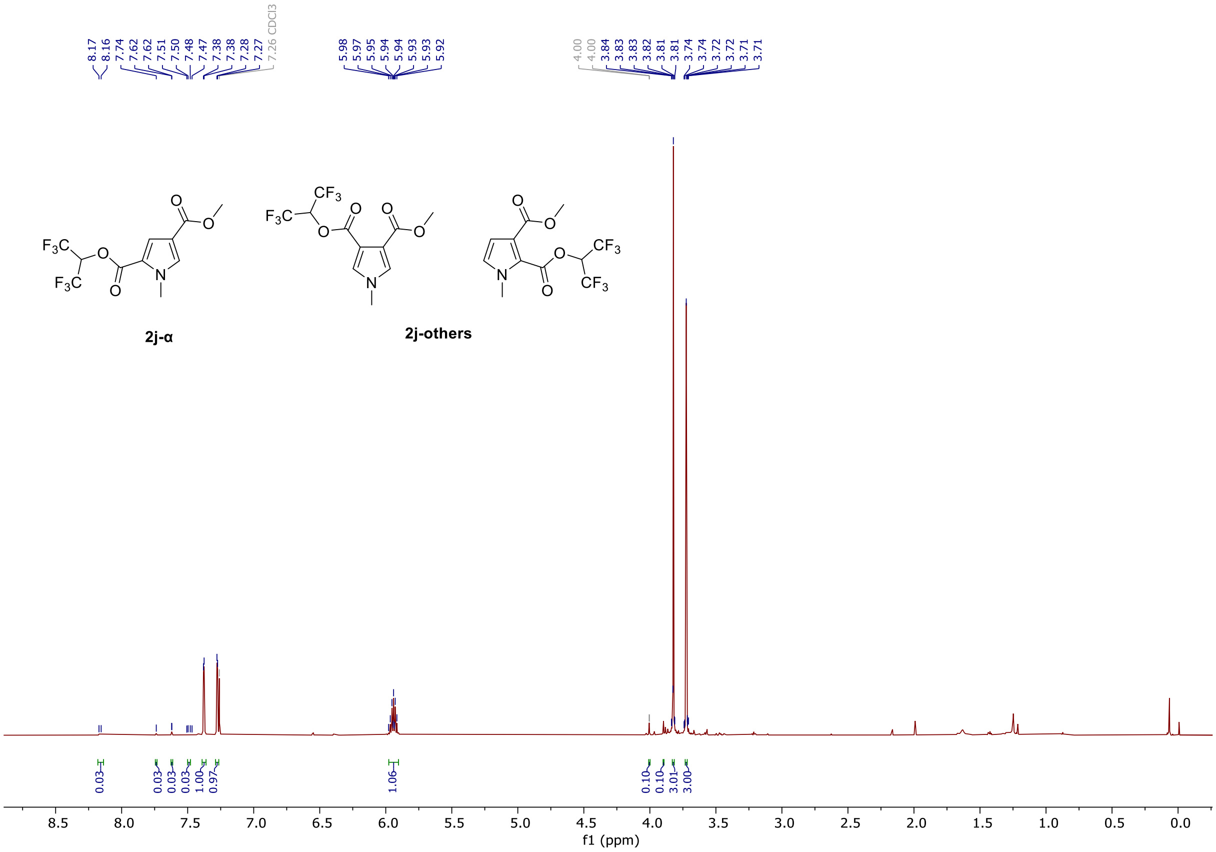

^{13}C-NMR in $CDCl_3$

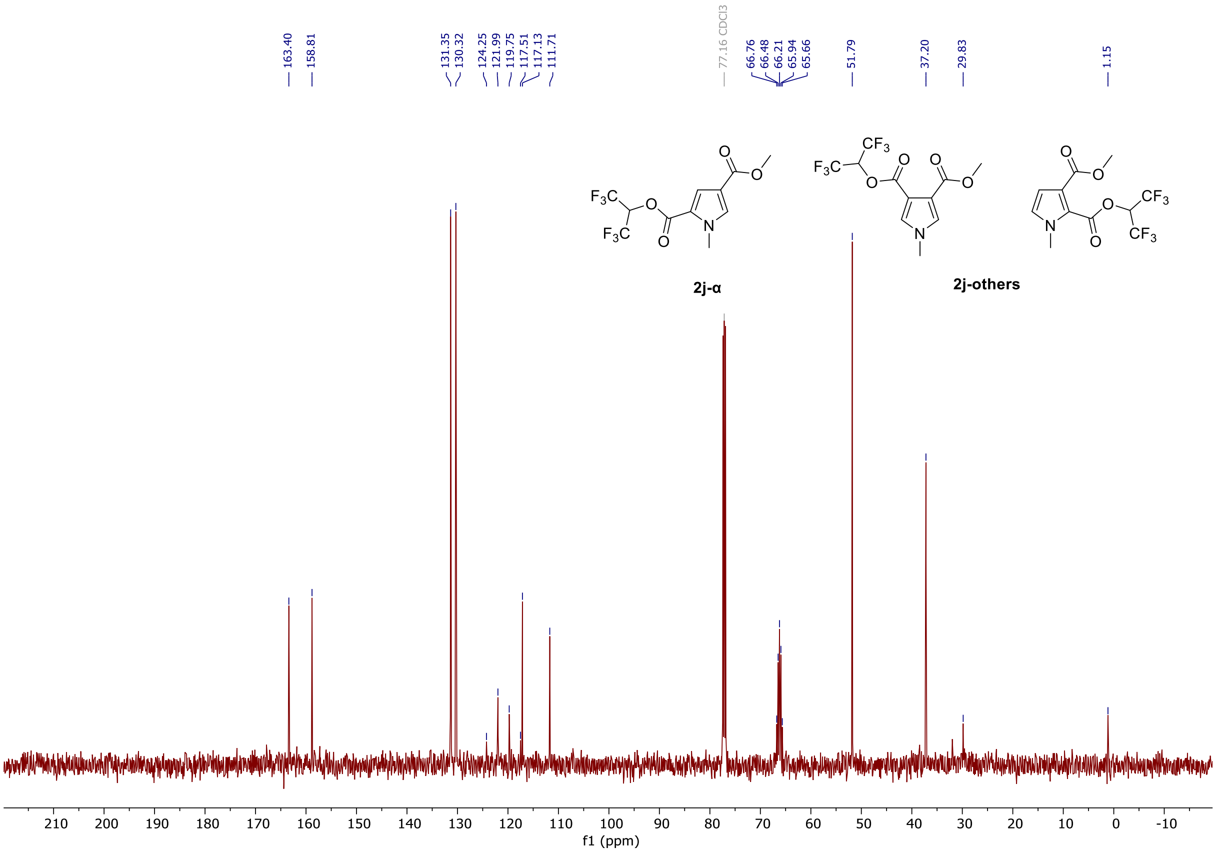

^{19}F-NMR in $CDCl_3$

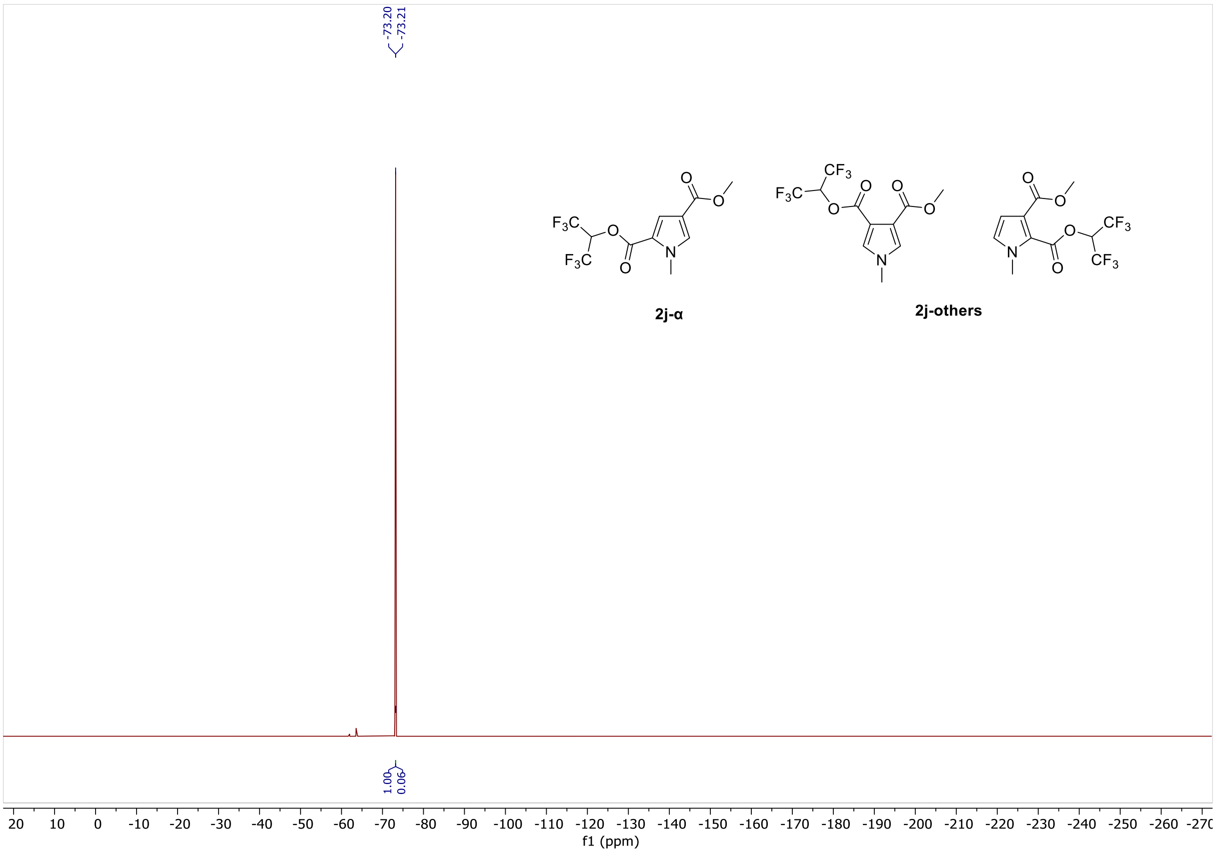

(1,1,1,3,3,3-Hexafluoropropan-2-yl) 5-methyl thiophene-dicarboxylate (2k)

^{1}H-NMR in $CDCl_3$

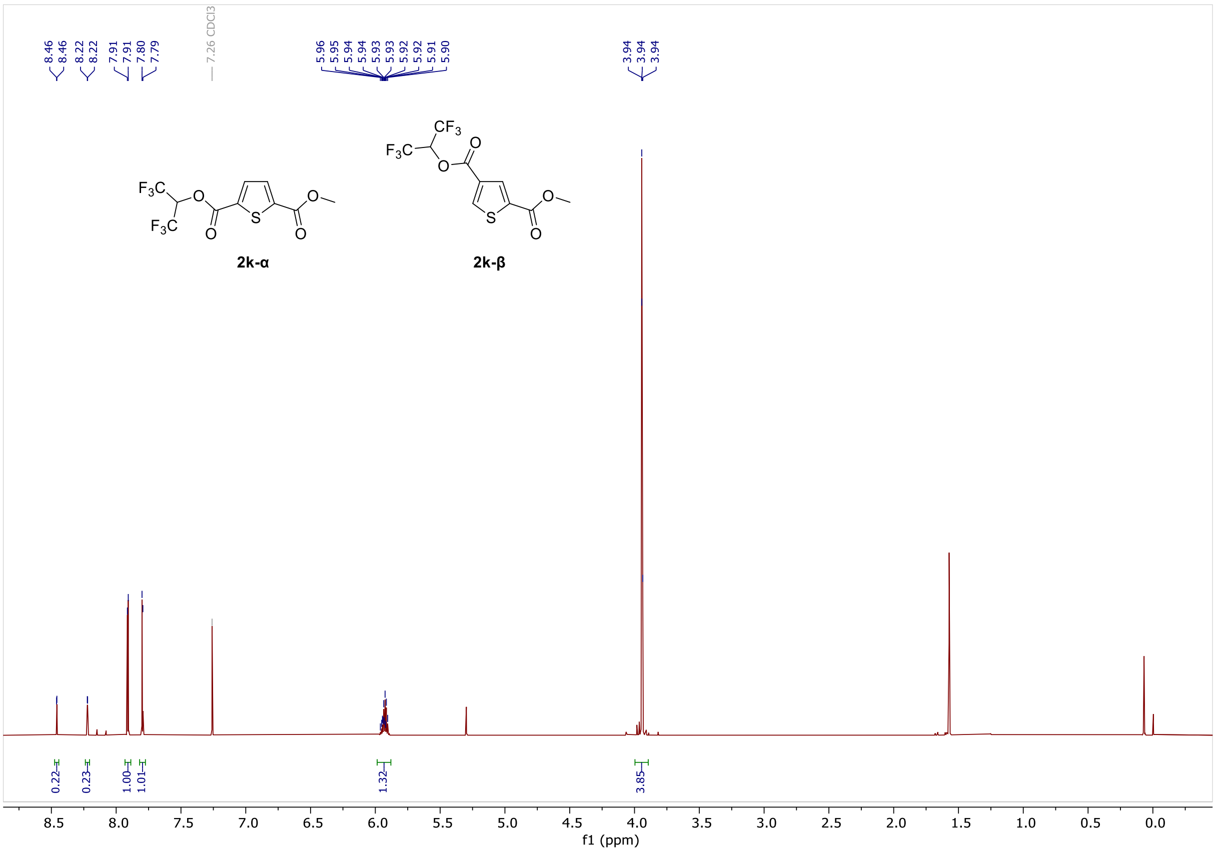

^{13}C-NMR in $CDCl_3$

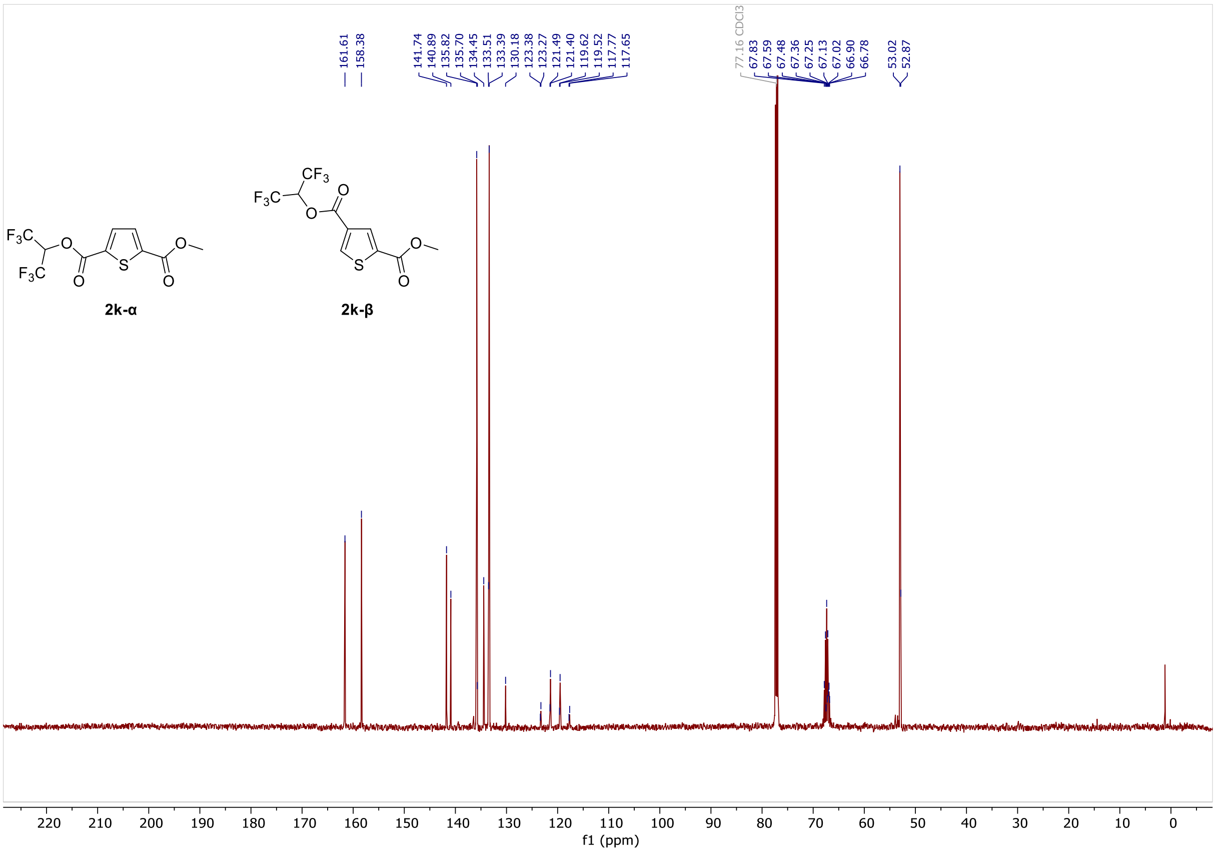

^{19}F-NMR in $CDCl_3$

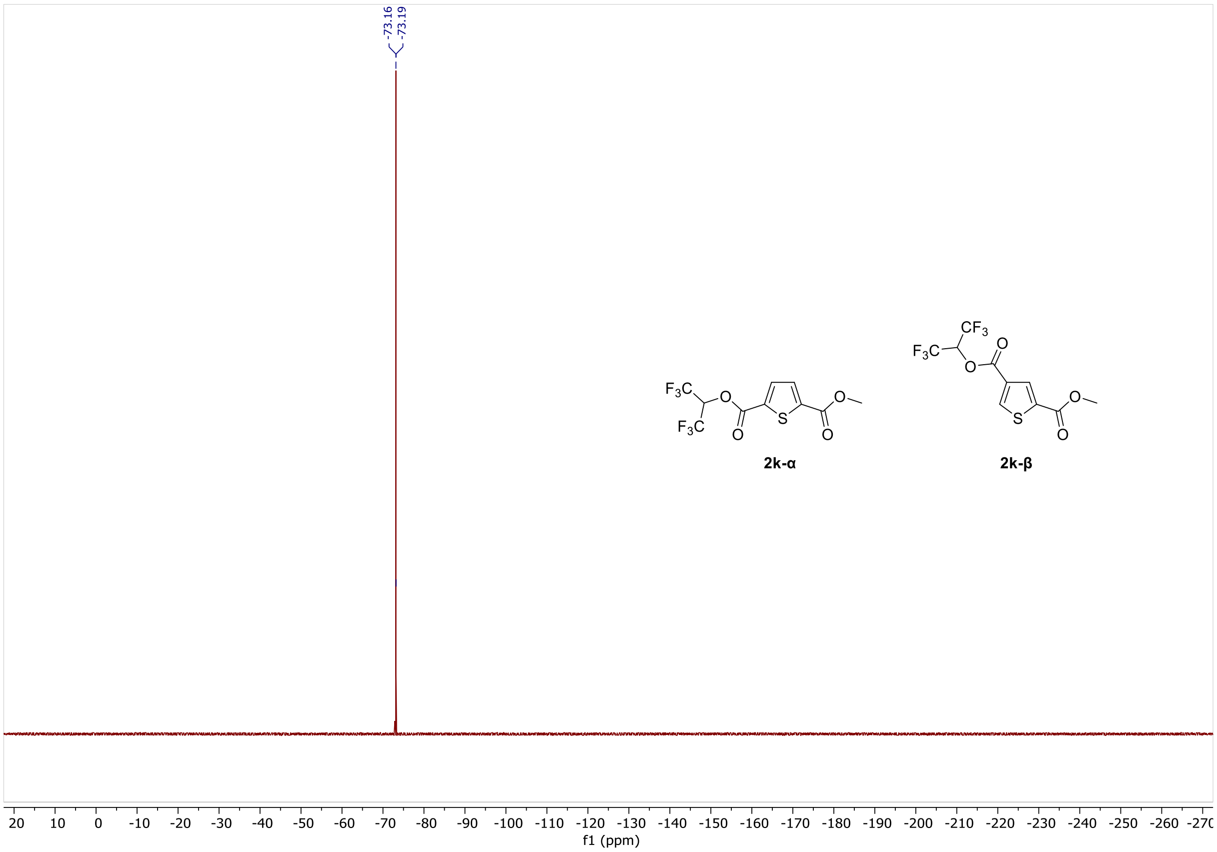

(1,1,1,3,3,3-Hexafluoropropan-2-yl) 5-methyl furan-dicarboxylate (2l)

^{1}H-NMR in $CDCl_3$

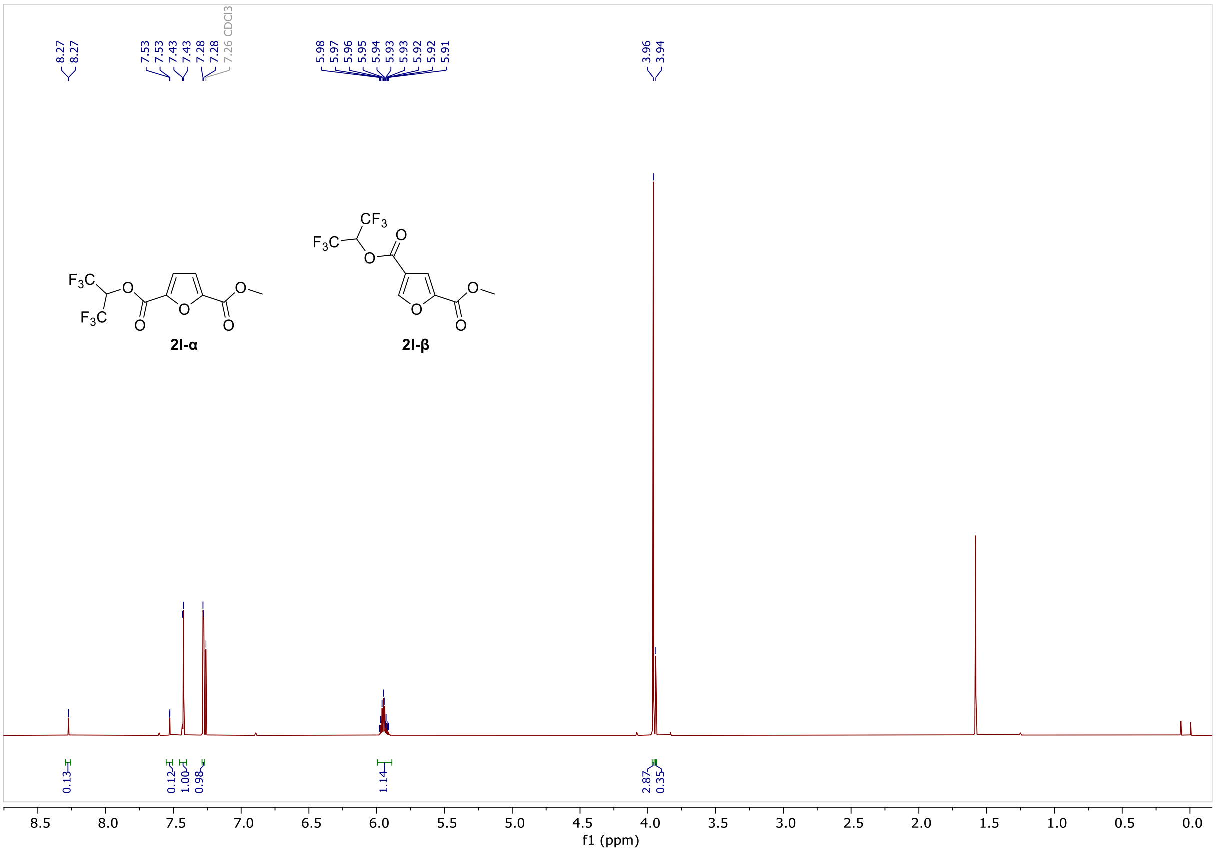

^{13}C-NMR in $CDCl_3$

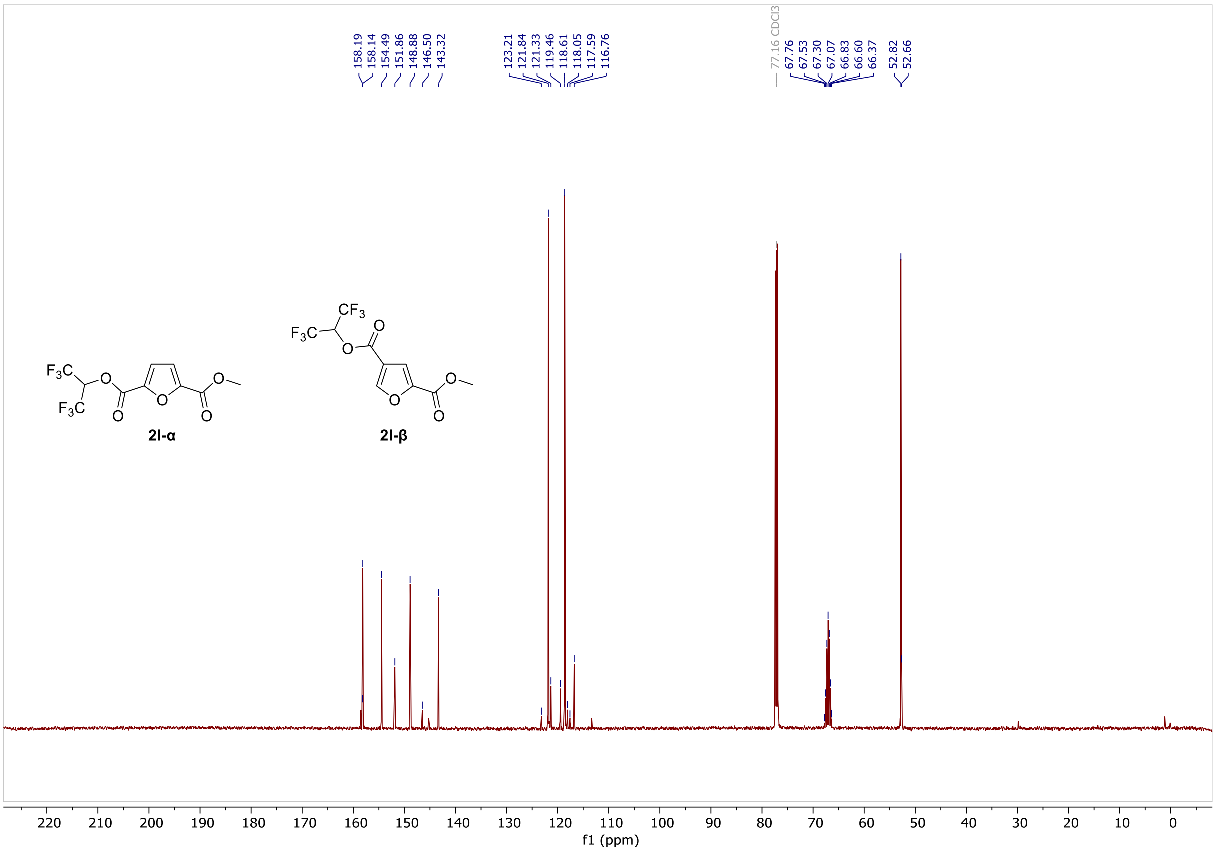

^{19}F-NMR in $CDCl_3$

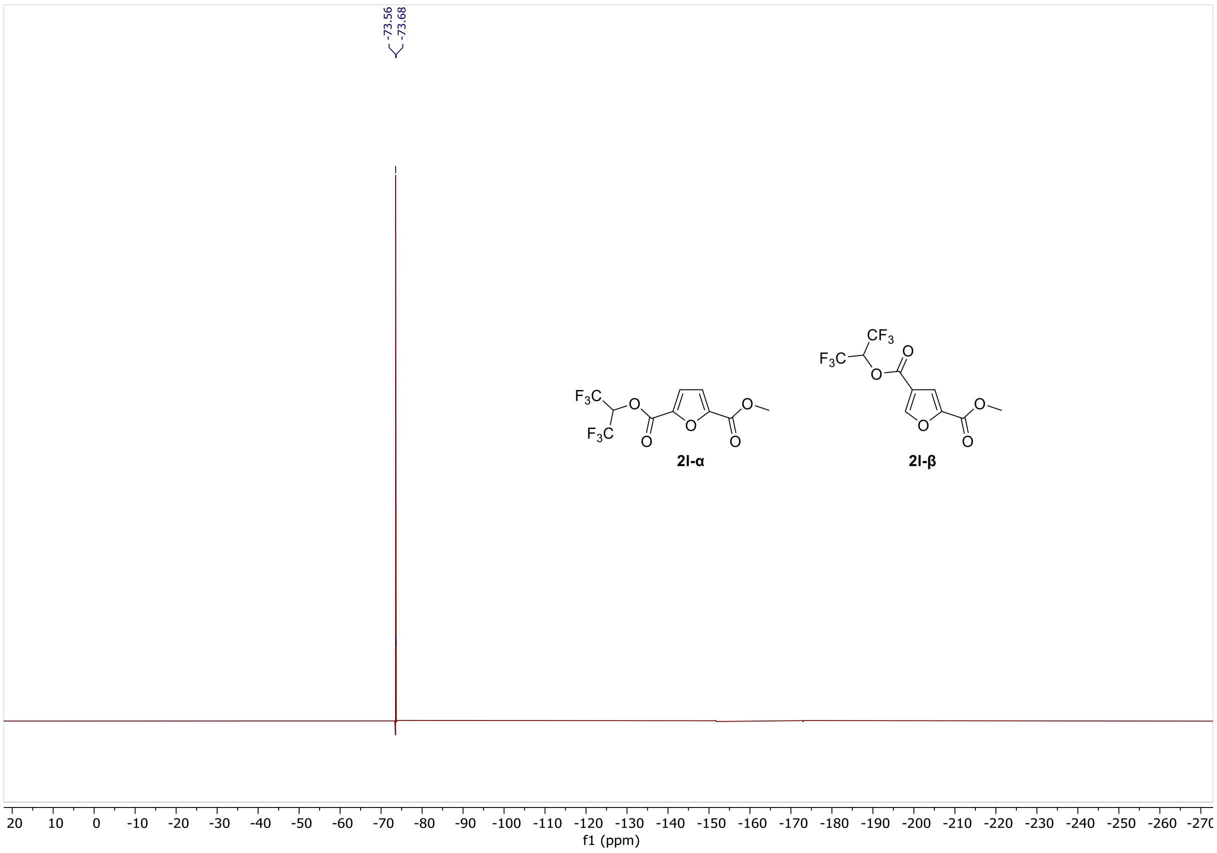

3,4-Dimethylbenzoic acid (4m)

^{1}H-NMR in $CDCl_3$

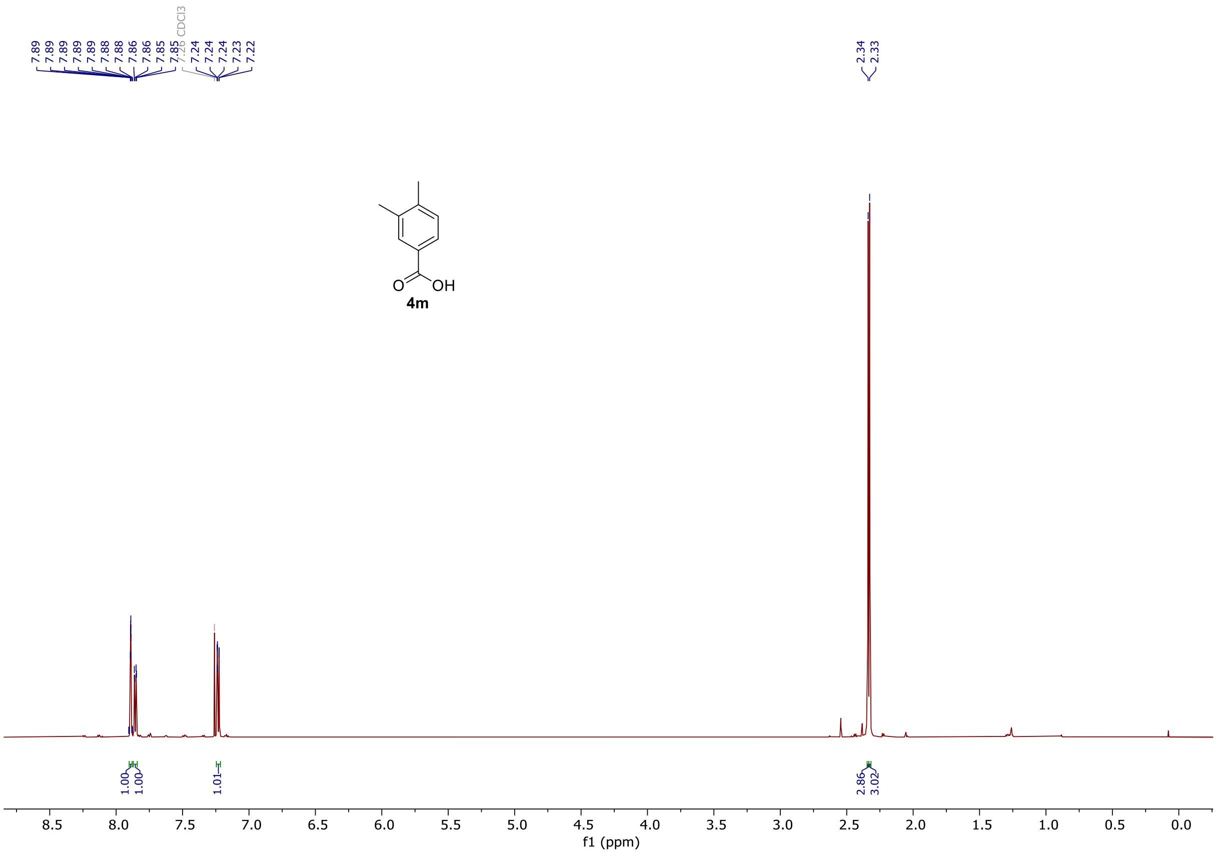

^{13}C-NMR in $CDCl_3$

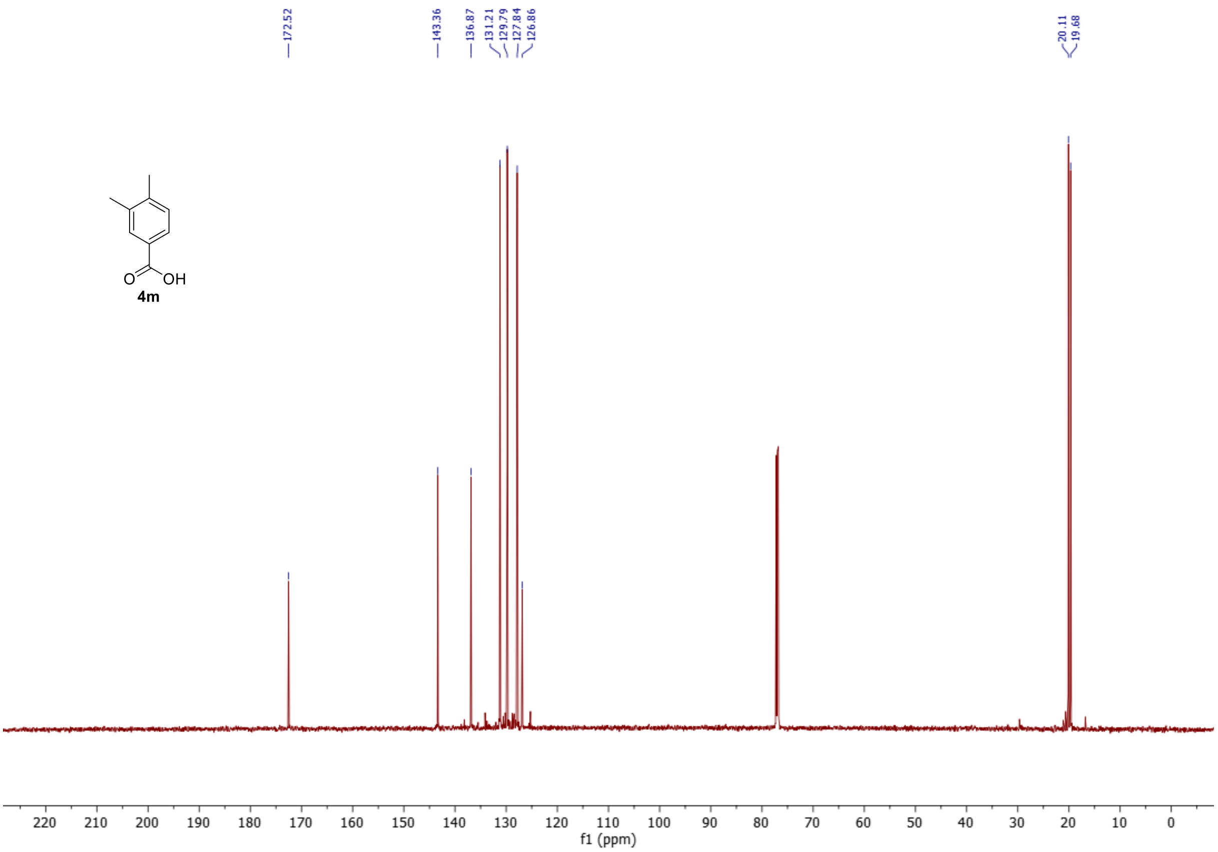

1,1,1,3,3,3-Hexafluoropropanyl dimethylbenzoate (2m)

^{1}H-NMR in $CDCl_3$

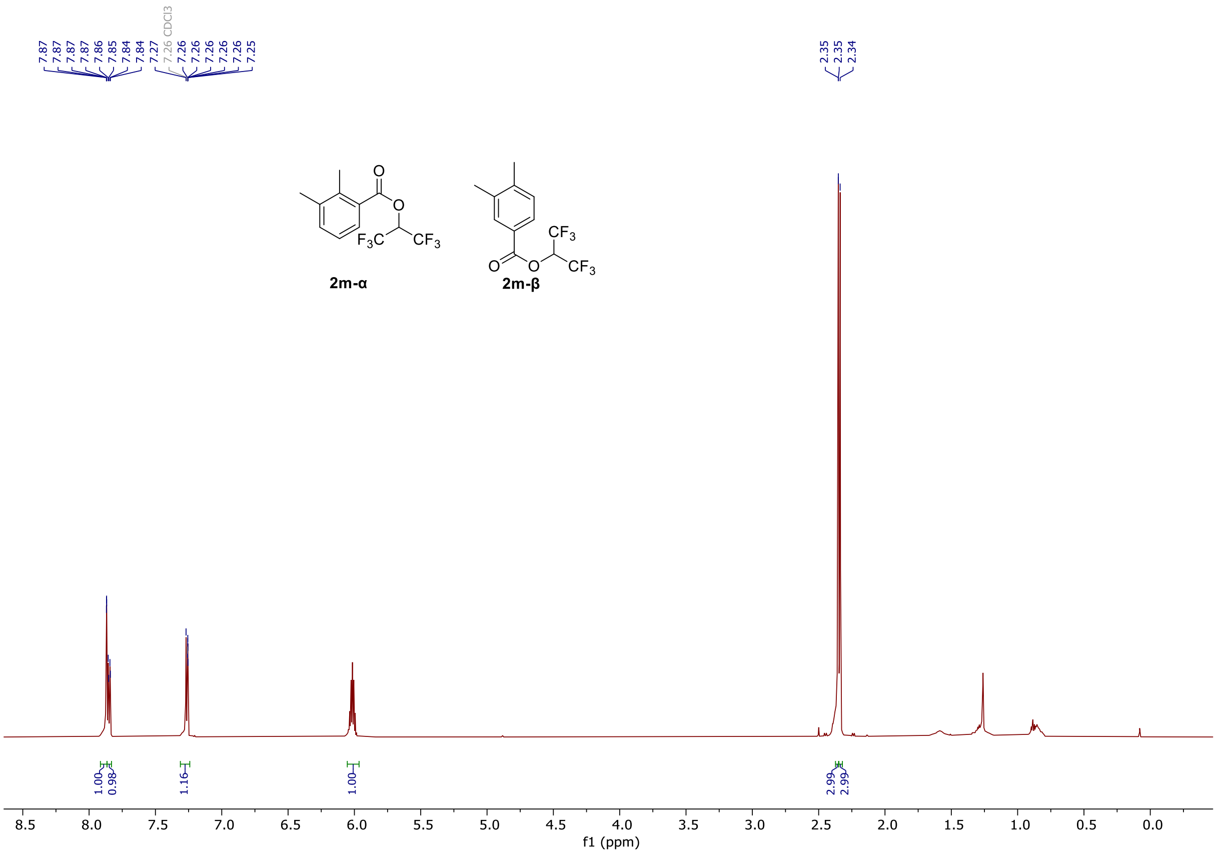

^{13}C-NMR in $CDCl_3$

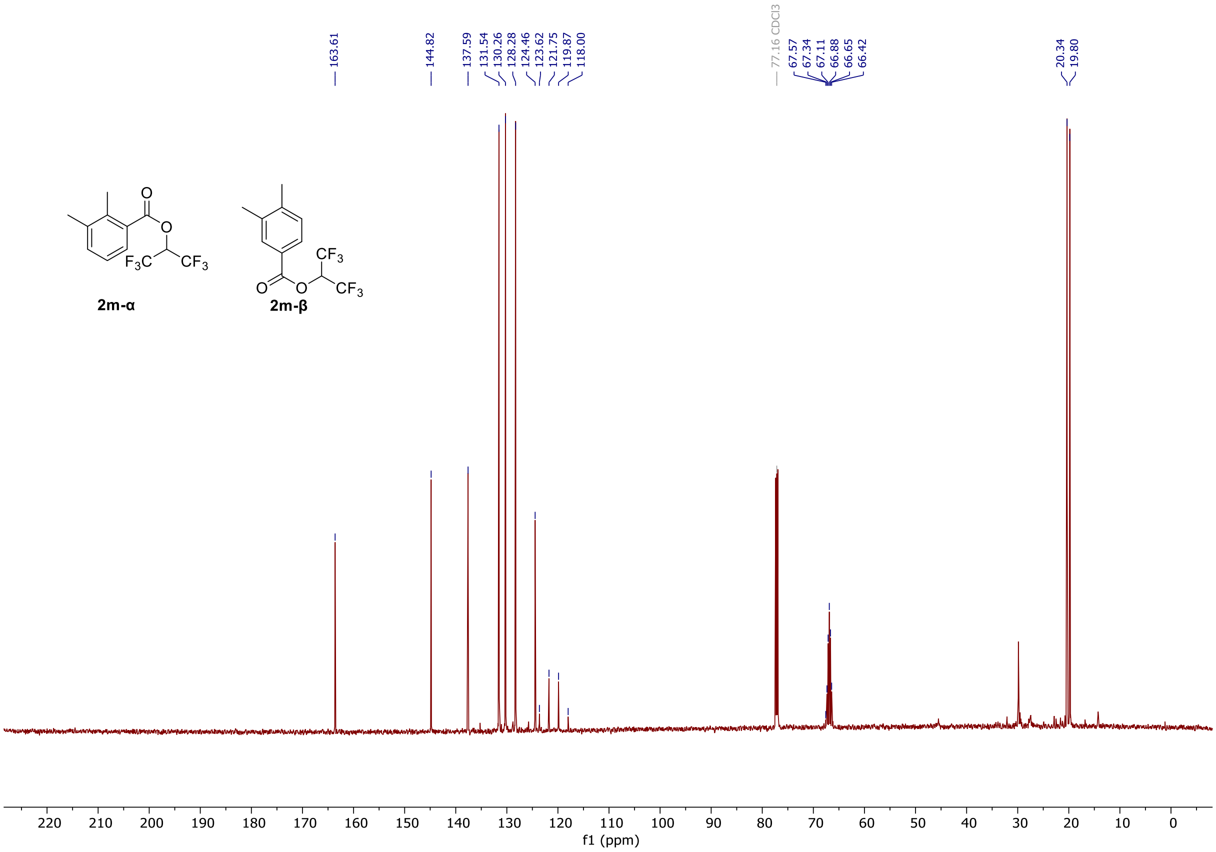

^{19}F-NMR in $CDCl_3$

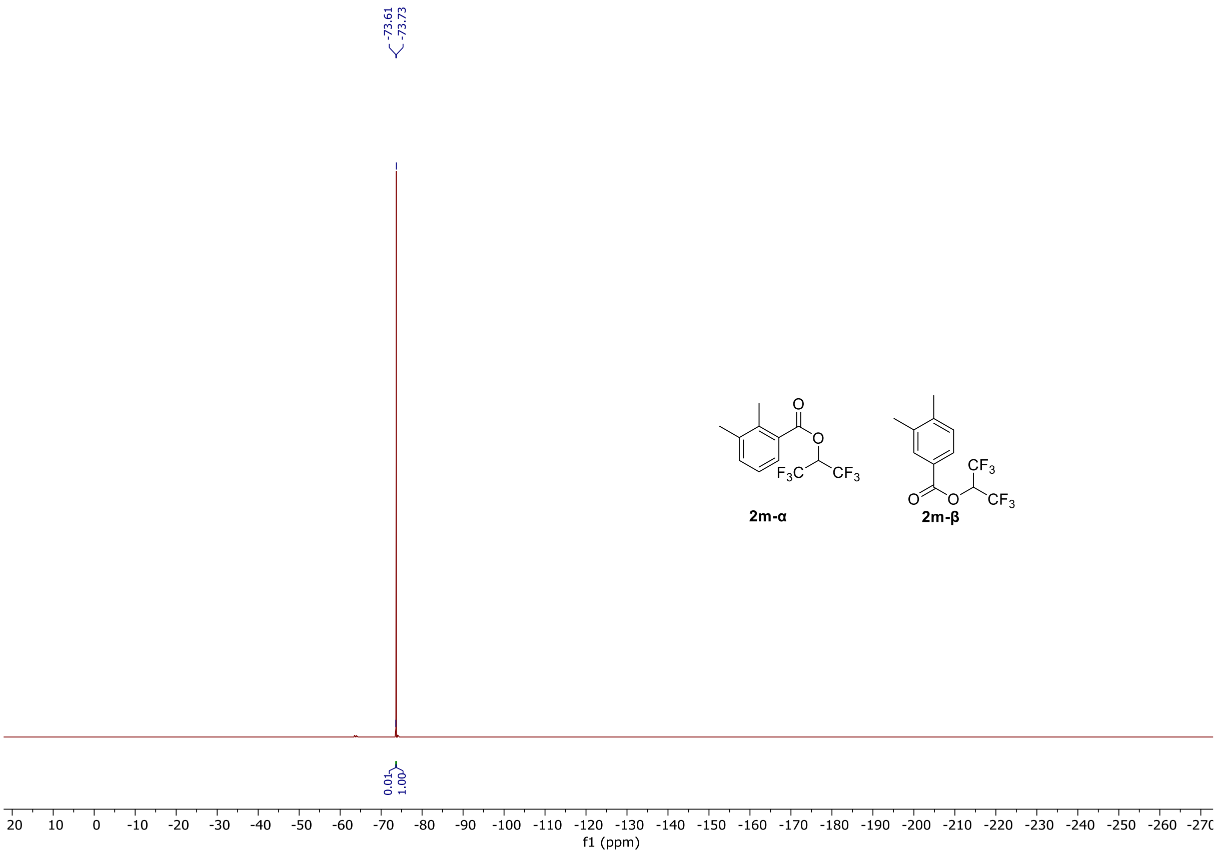

2-Fluoromethylbenzoic acid (4n)

^{1}H-NMR in DMSO-d$_6$

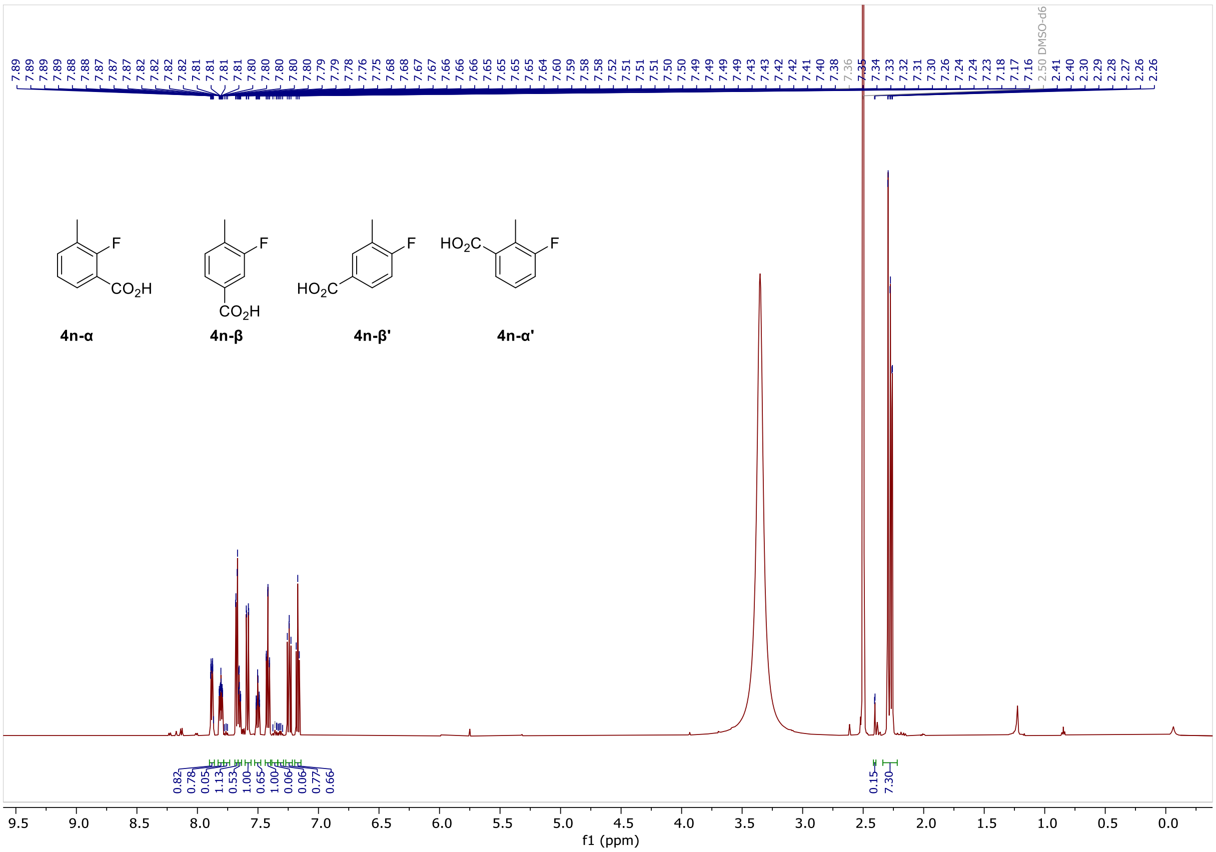

^{13}C-NMR in DMSO-d$_6$

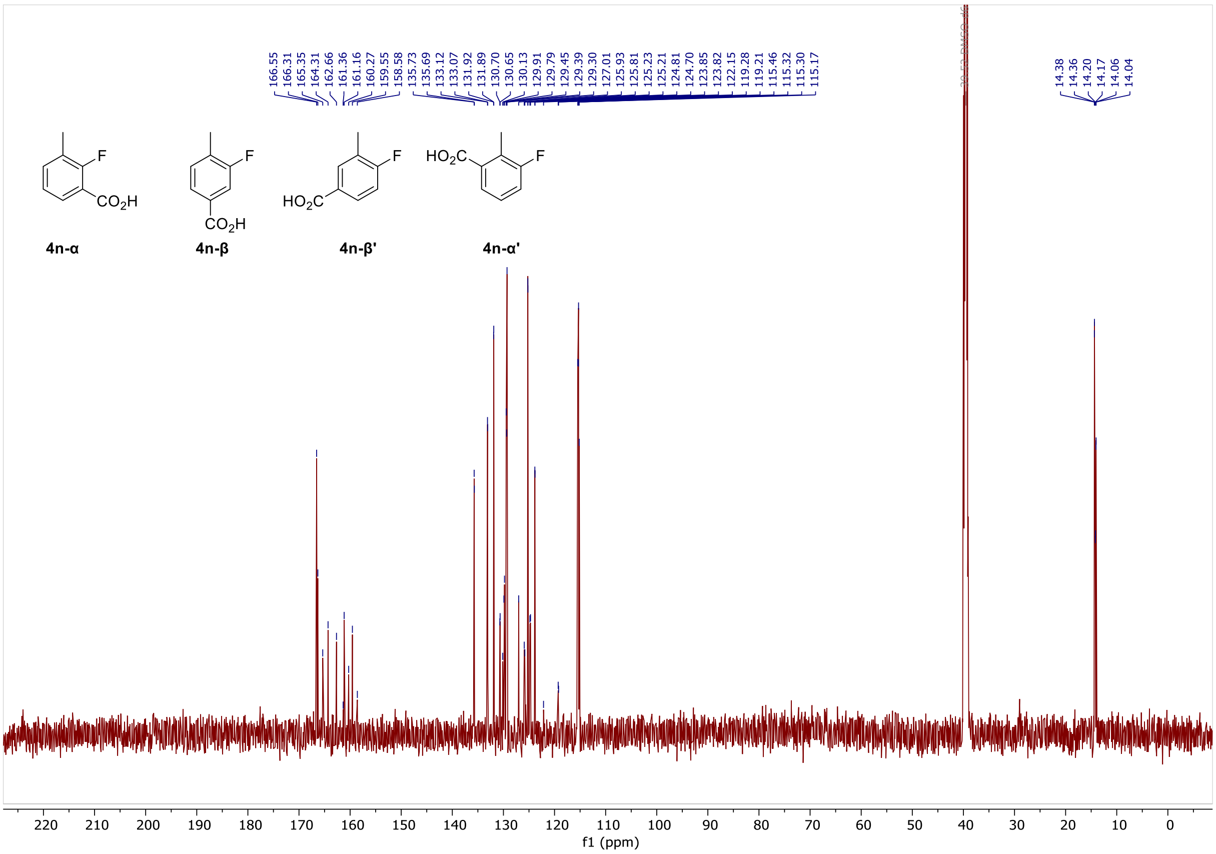

^{19}F-NMR in DMSO-d$_6$

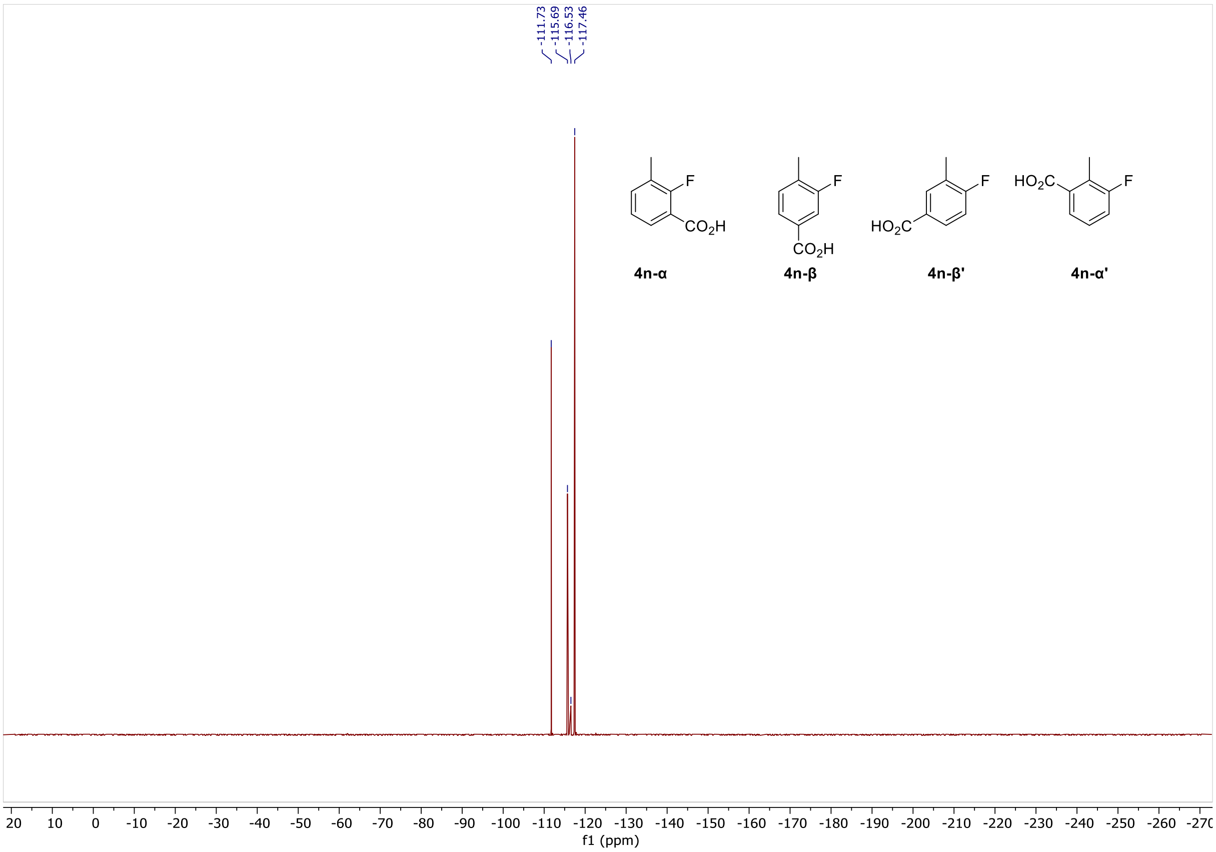

1,2-Dichlorobenzoic acid (4o)
^{1}H-NMR in DMSO-d$_6$

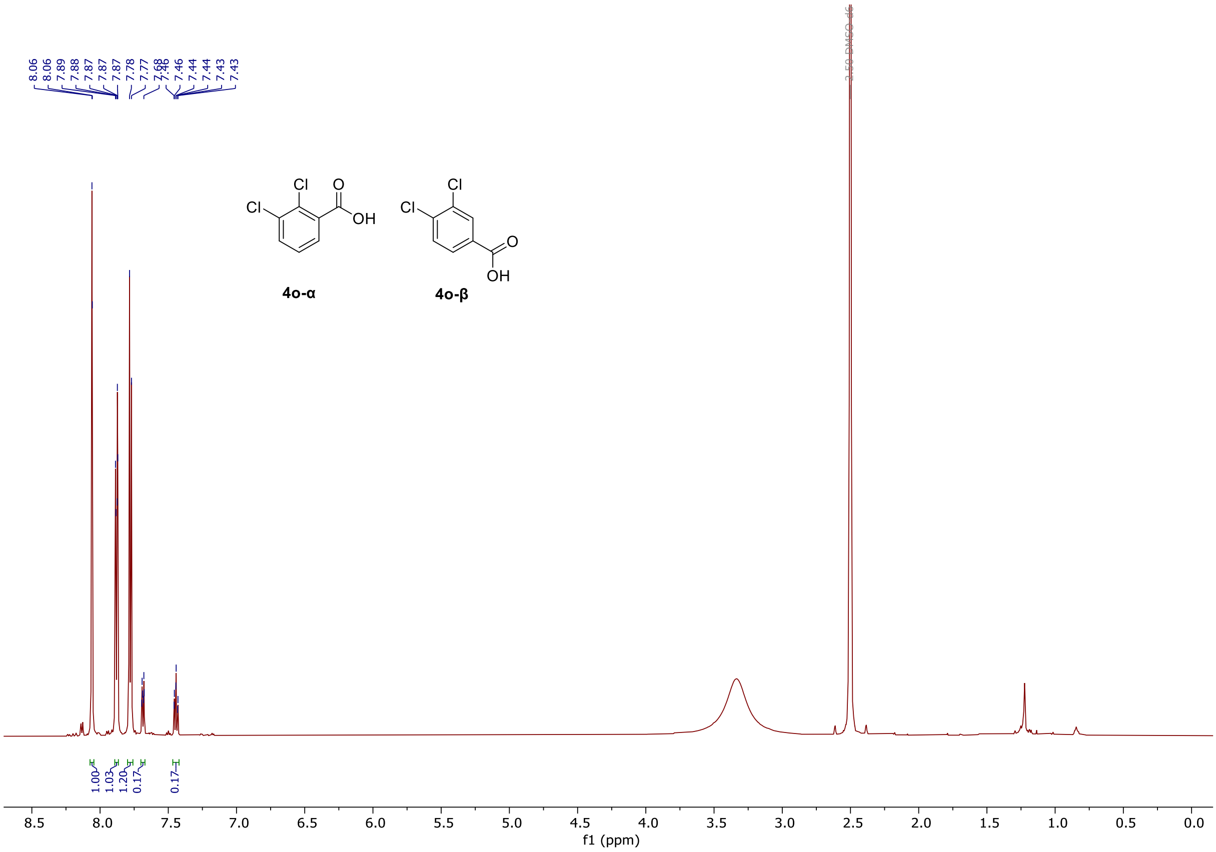

^{13}C-NMR in DMSO-d$_6$

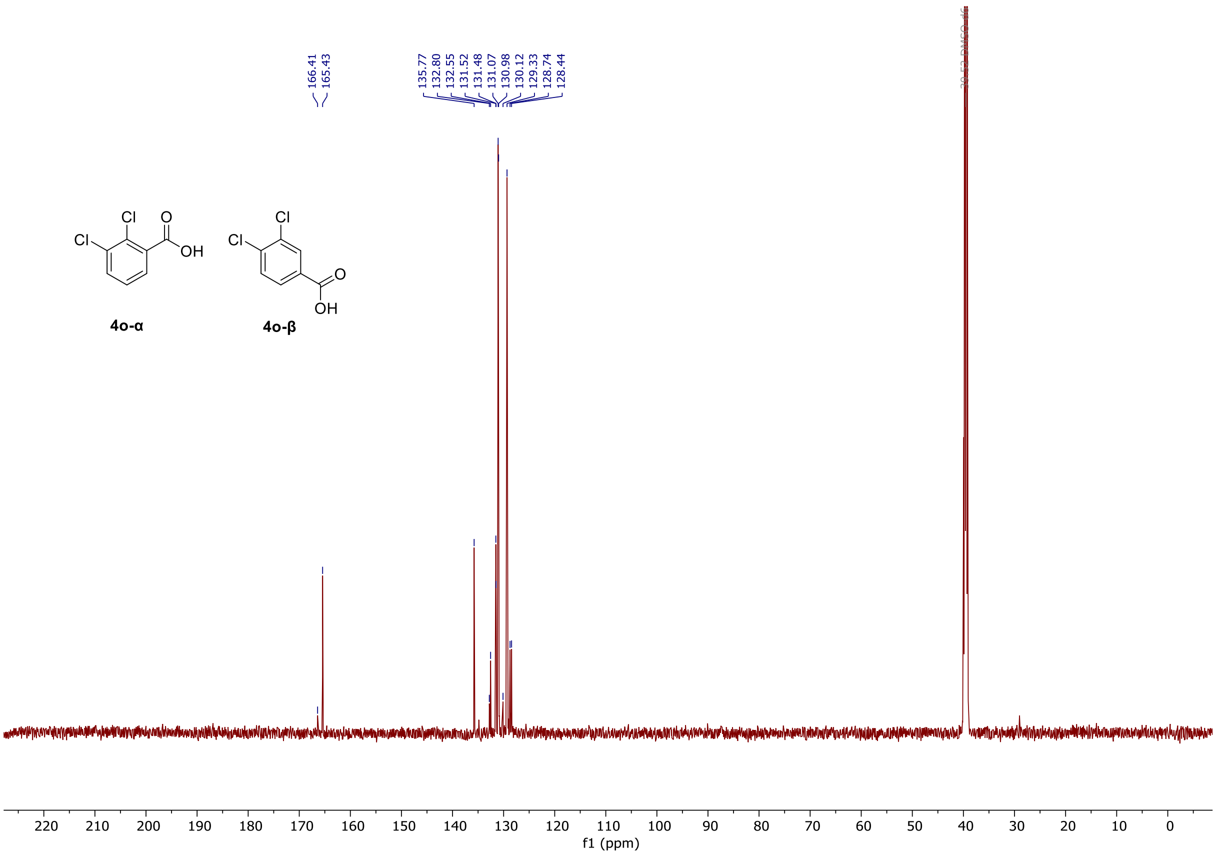

1,3-Dimethylbenzoic acid (4p)

^{1}H-NMR in $CDCl_3$

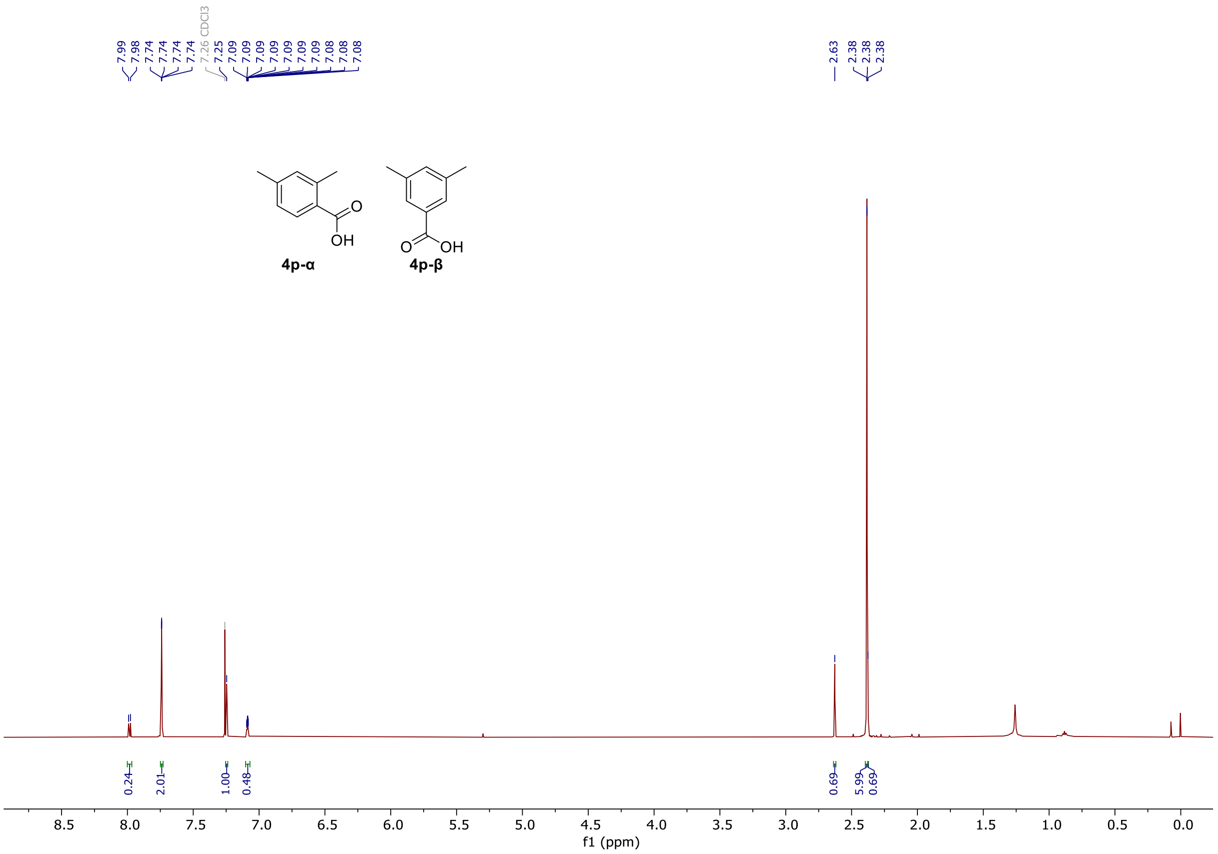

^{13}C-NMR in $CDCl_3$

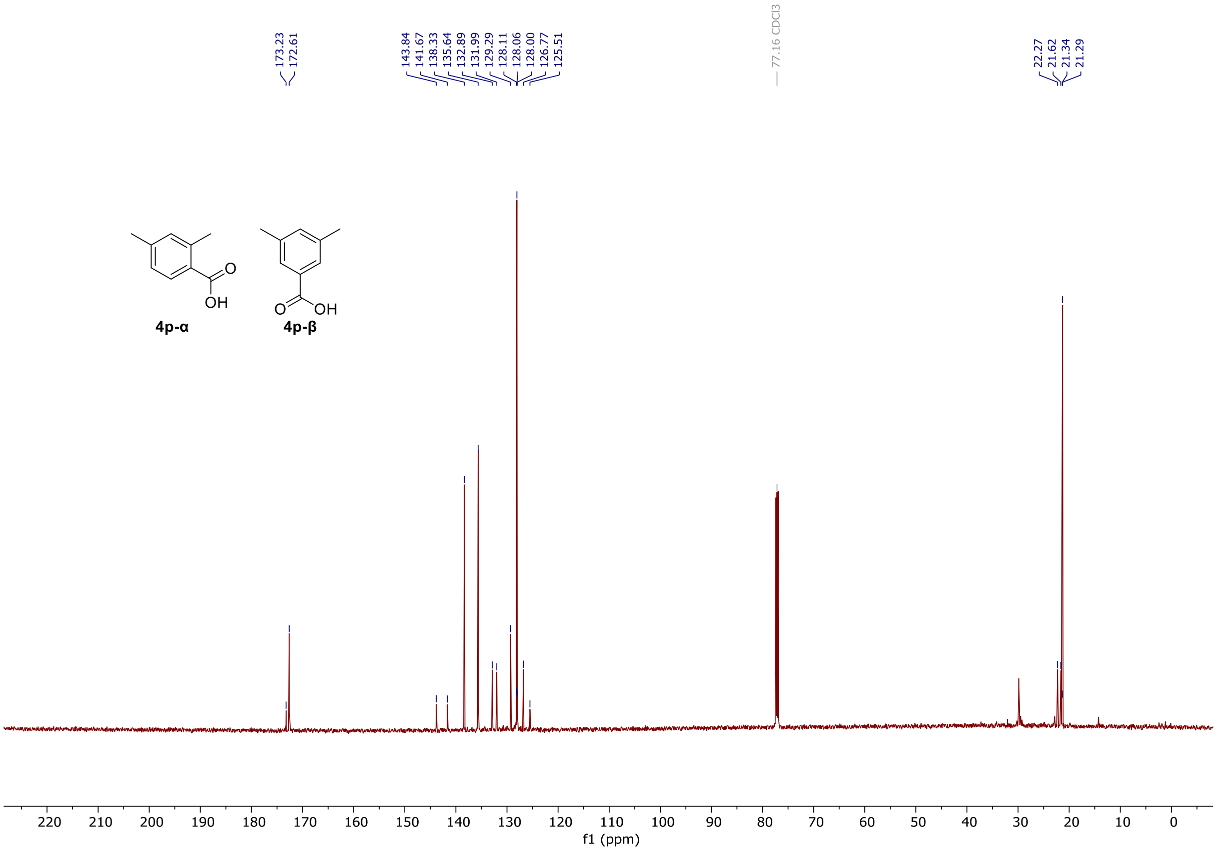

3,5-di-tert-butylbenzoic acid (4q)

^{1}H-NMR in $CDCl_3$

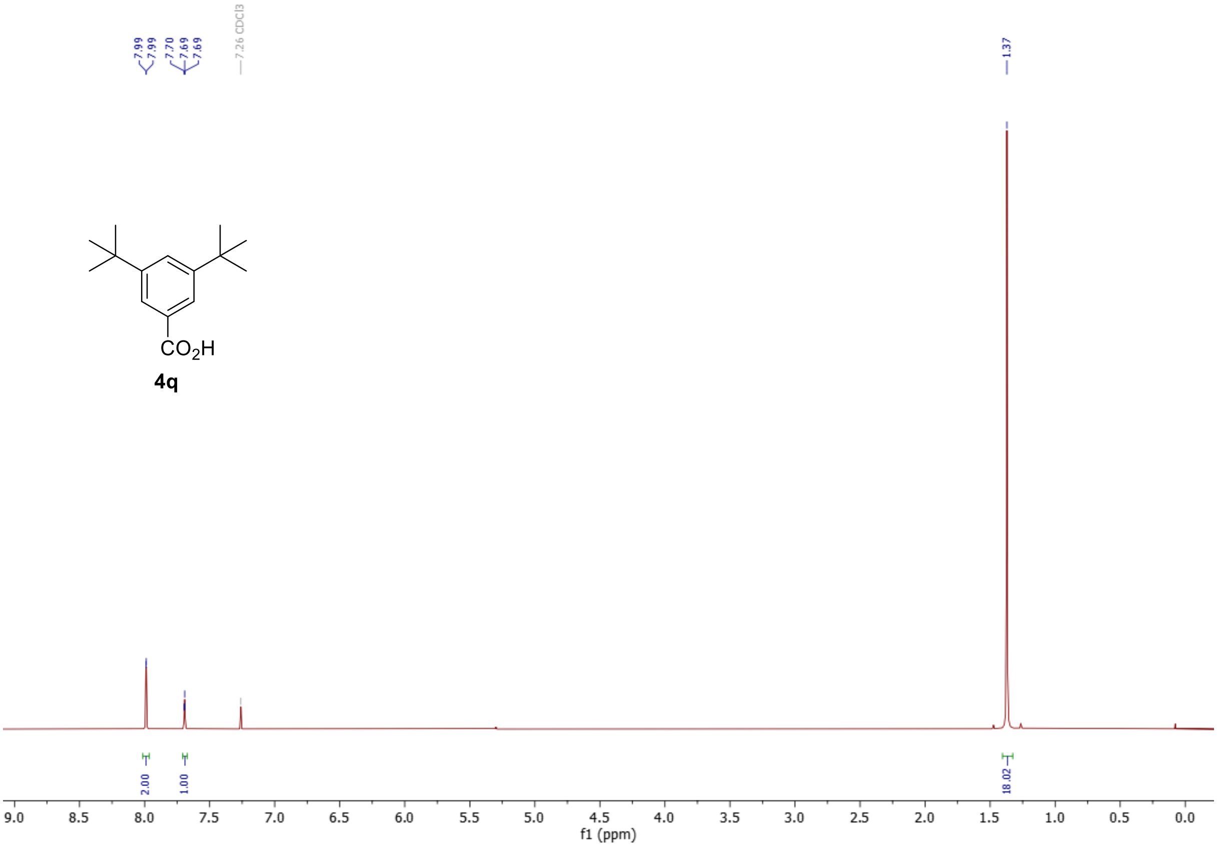

^{13}C-NMR in $CDCl_3$

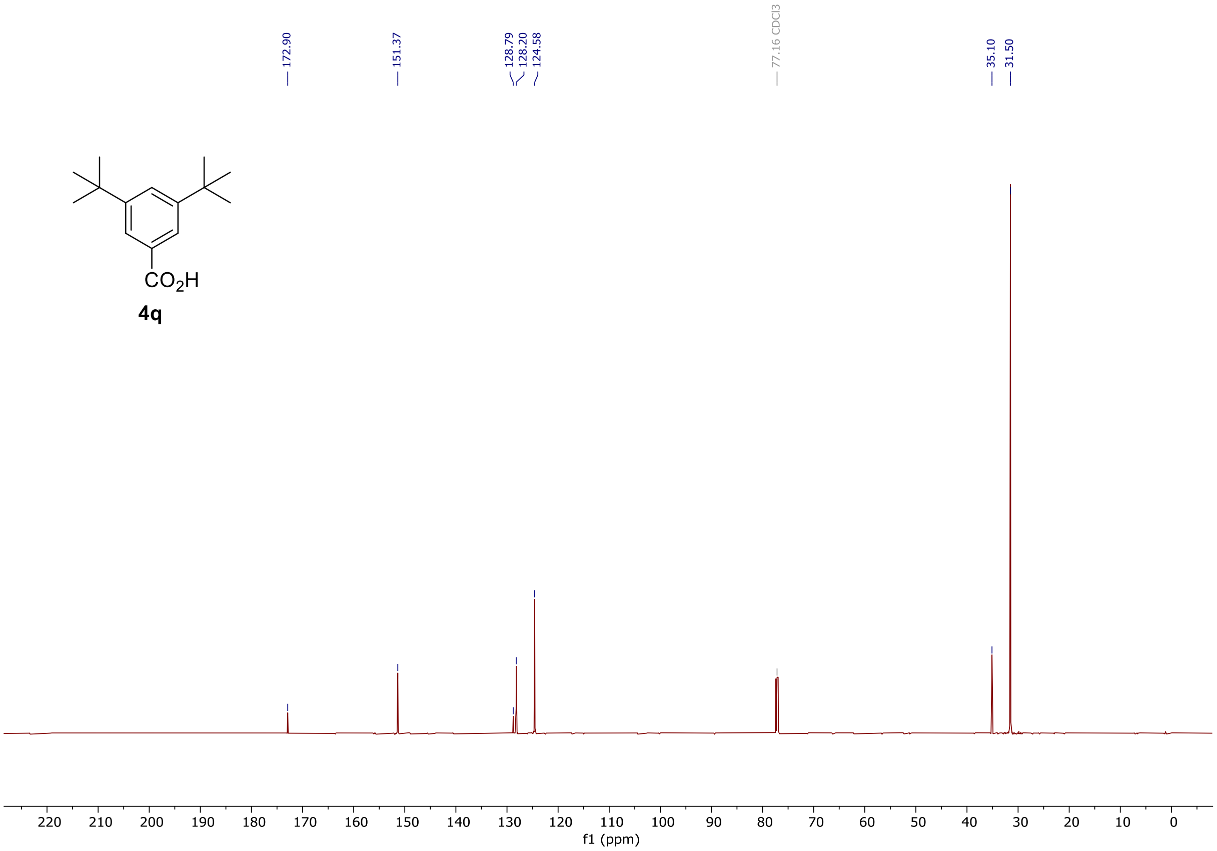

3-Methyl-1-methoxybenzoic acid (4r)

^{1}H-NMR of 4r-α in $CDCl_3$

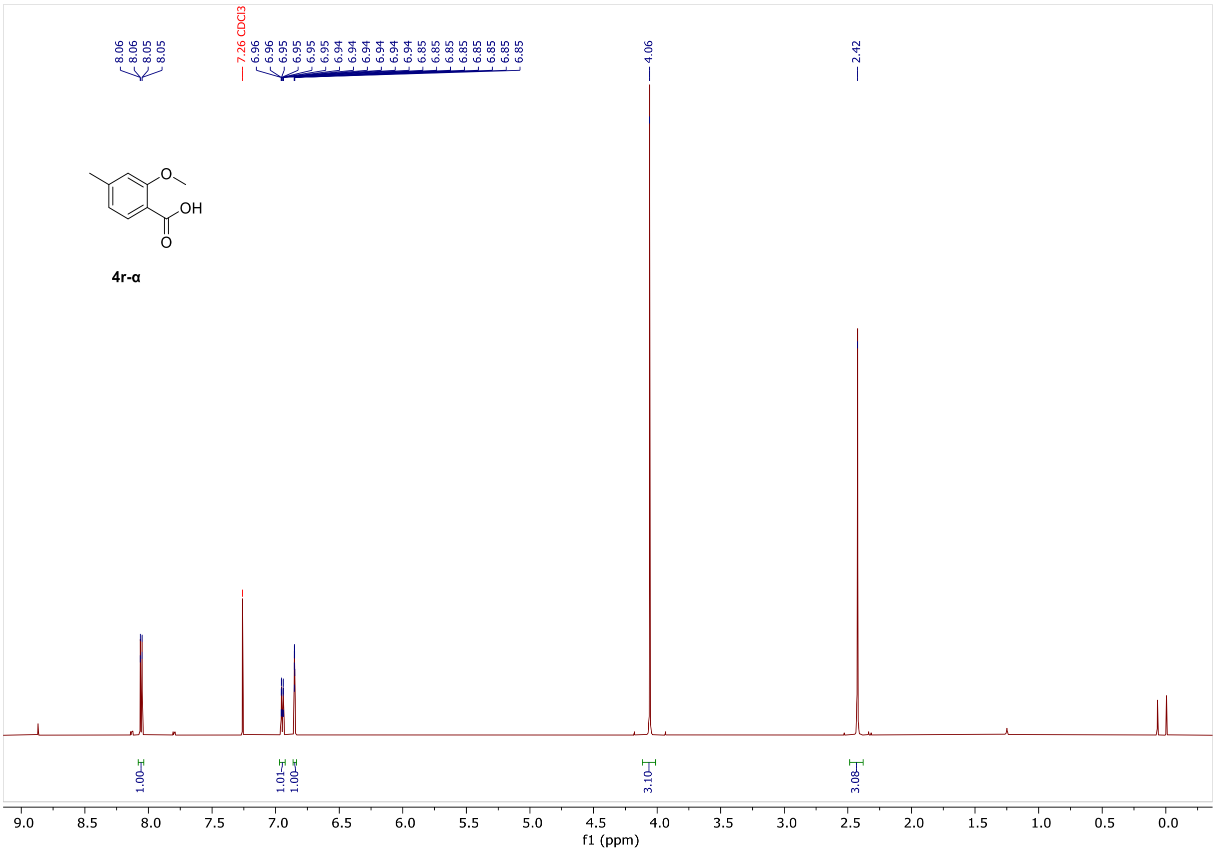

^{13}C-NMR of 4r-α in $CDCl_3$

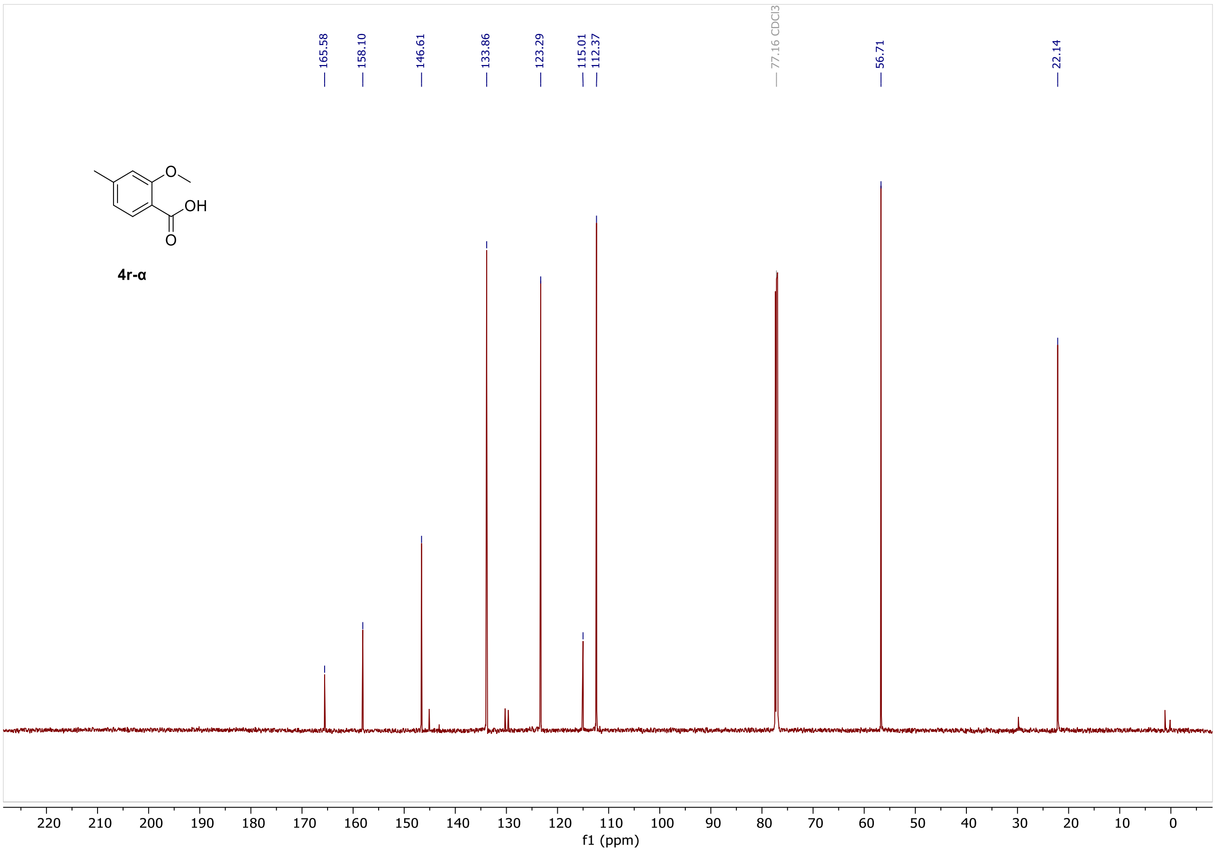

^{1}H-NMR of 4r-β and 4r-γ in $CDCl_3$

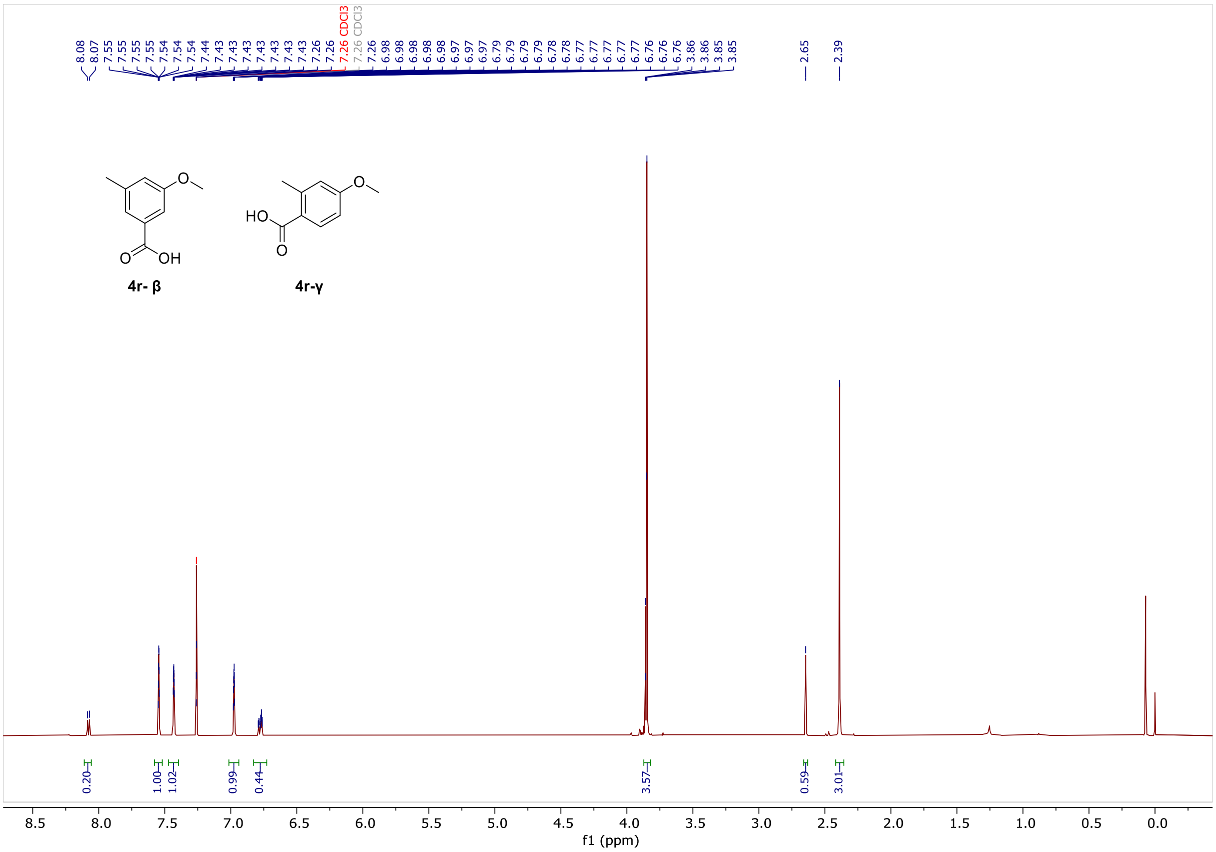

^{13}C-NMR of 4r-β and 4r-γ in $CDCl_3$

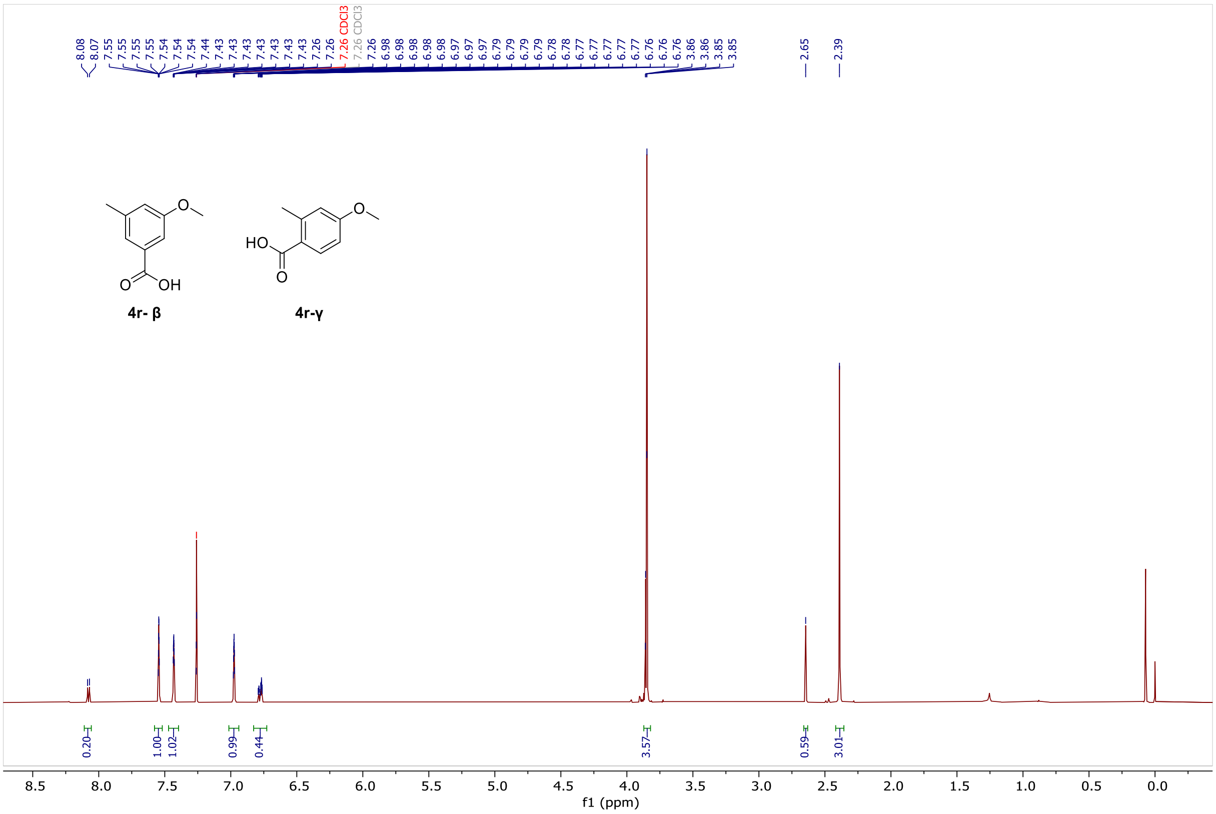

1,1,1,3,3,3-Hexafluoropropan-2-yl 3-(tert-butyldimethylsilyl)-5-((tert-butyldimethylsilyl)oxy)benzoate (2s)

^{1}H-NMR in $CDCl_3$

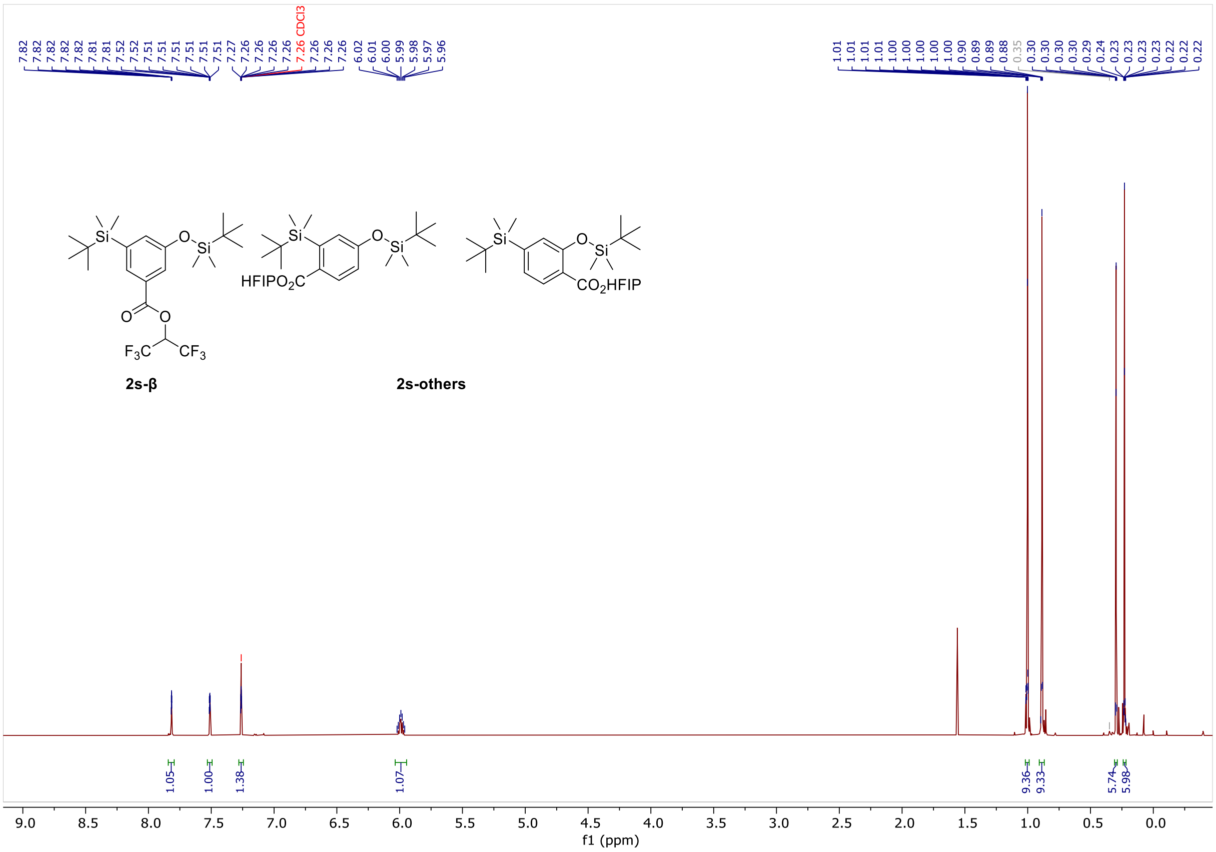

^{13}C-NMR in $CDCl_3$

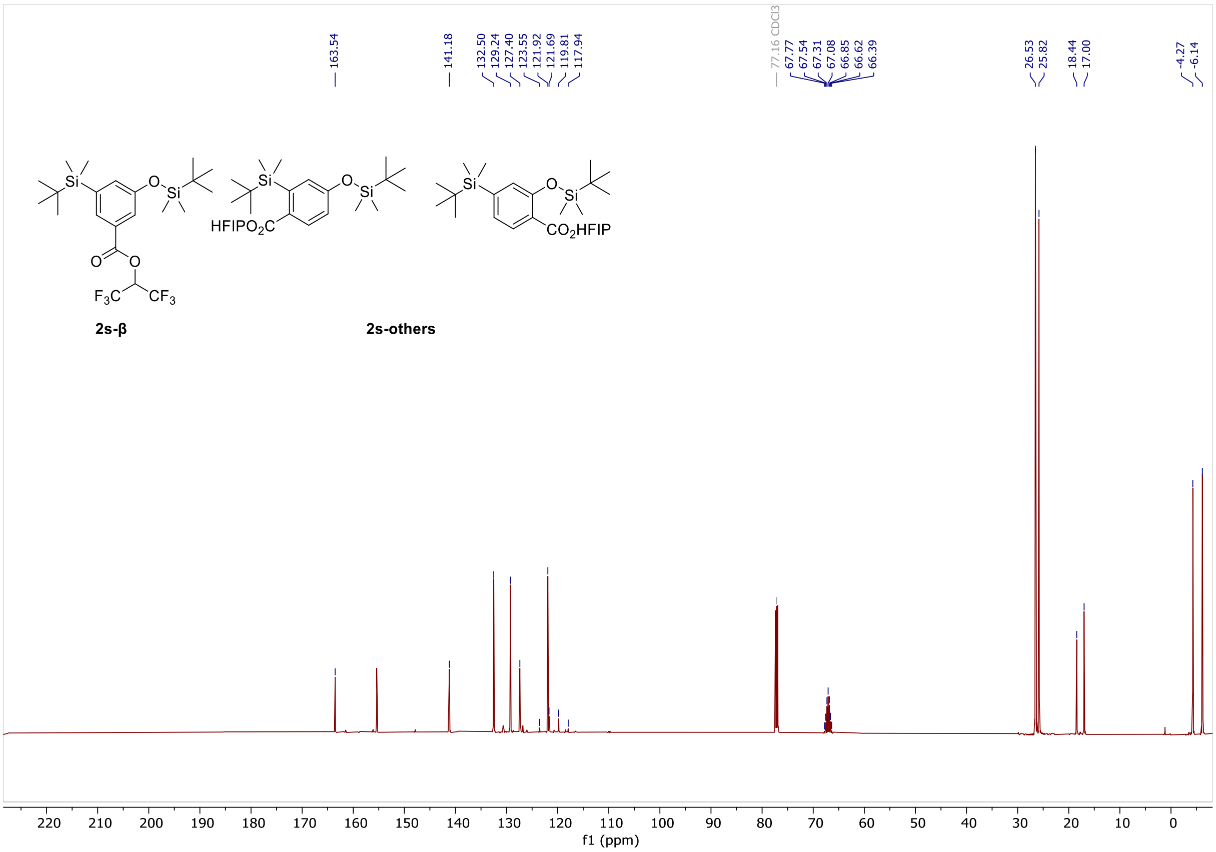

^{19}F-NMR in CDCl$_3$

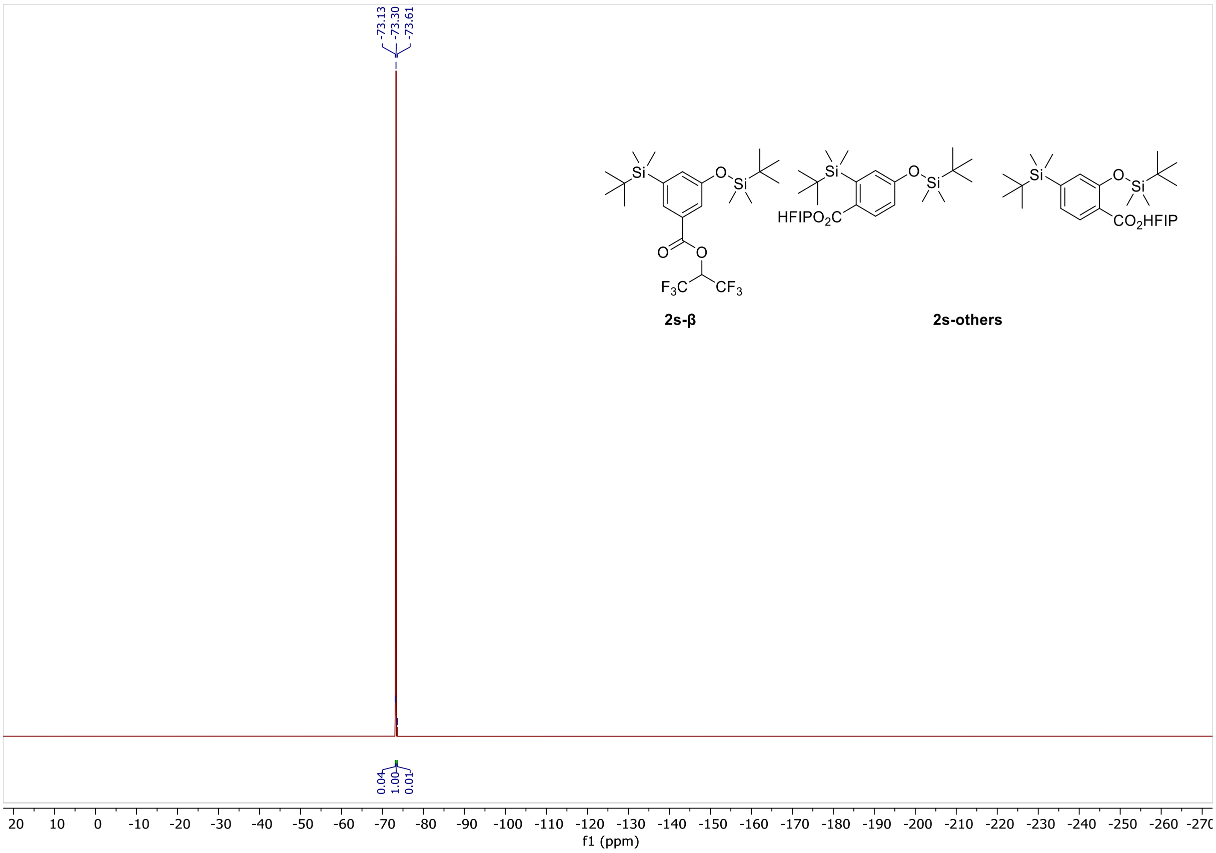

1,1,1,3,3,3-hexafluoropropan-2-yl (1-methoxy-3-oxopropyl)-3-methylbenzoate (2t)

^{1}H-NMR in $CDCl_3$

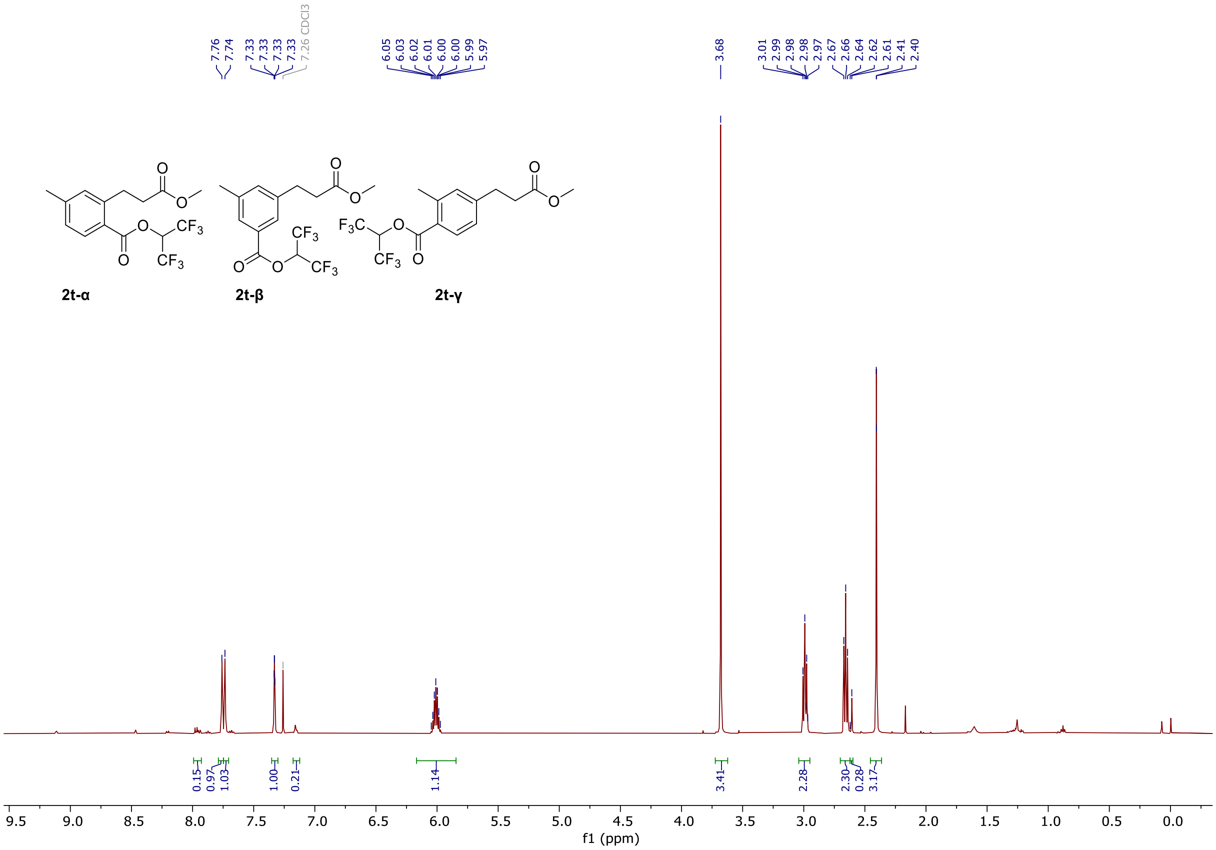

^{13}C-NMR in $CDCl_3$

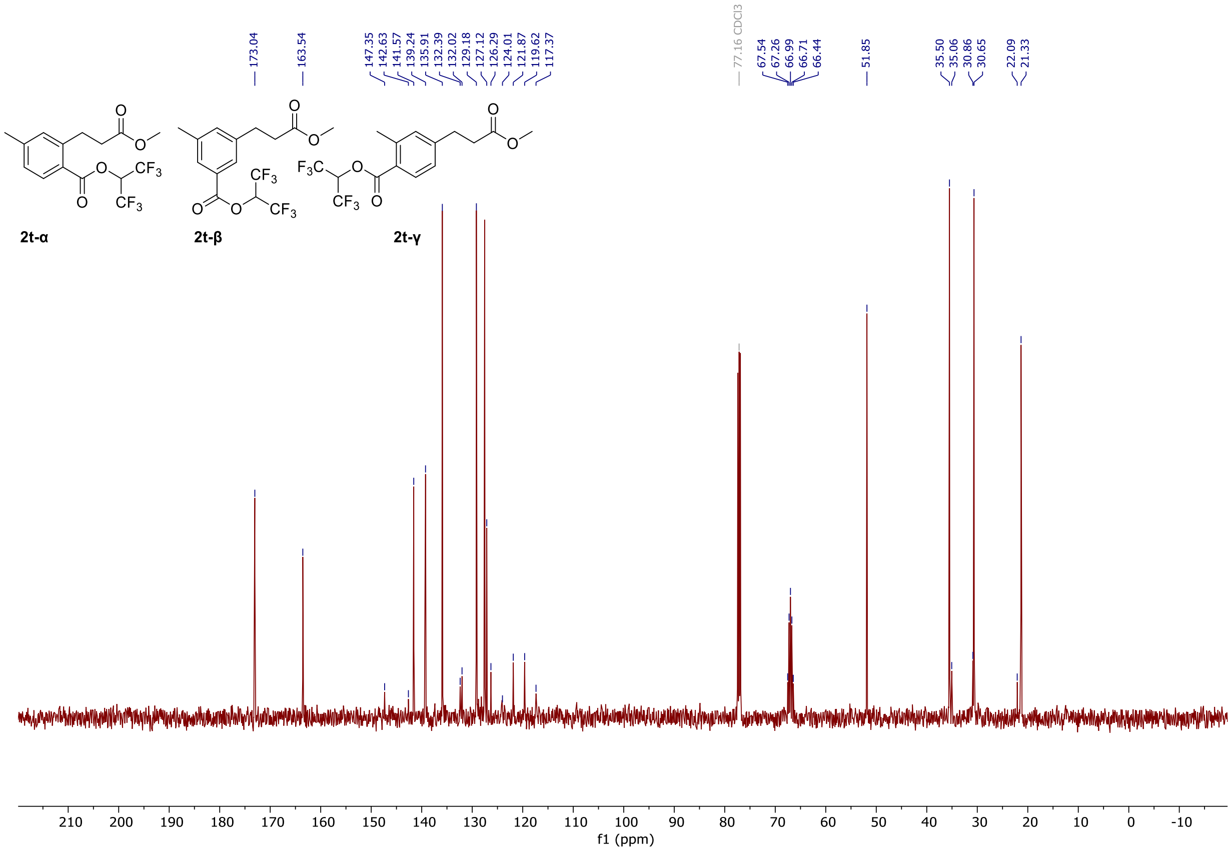

^{19}F-NMR in CDCl$_3$

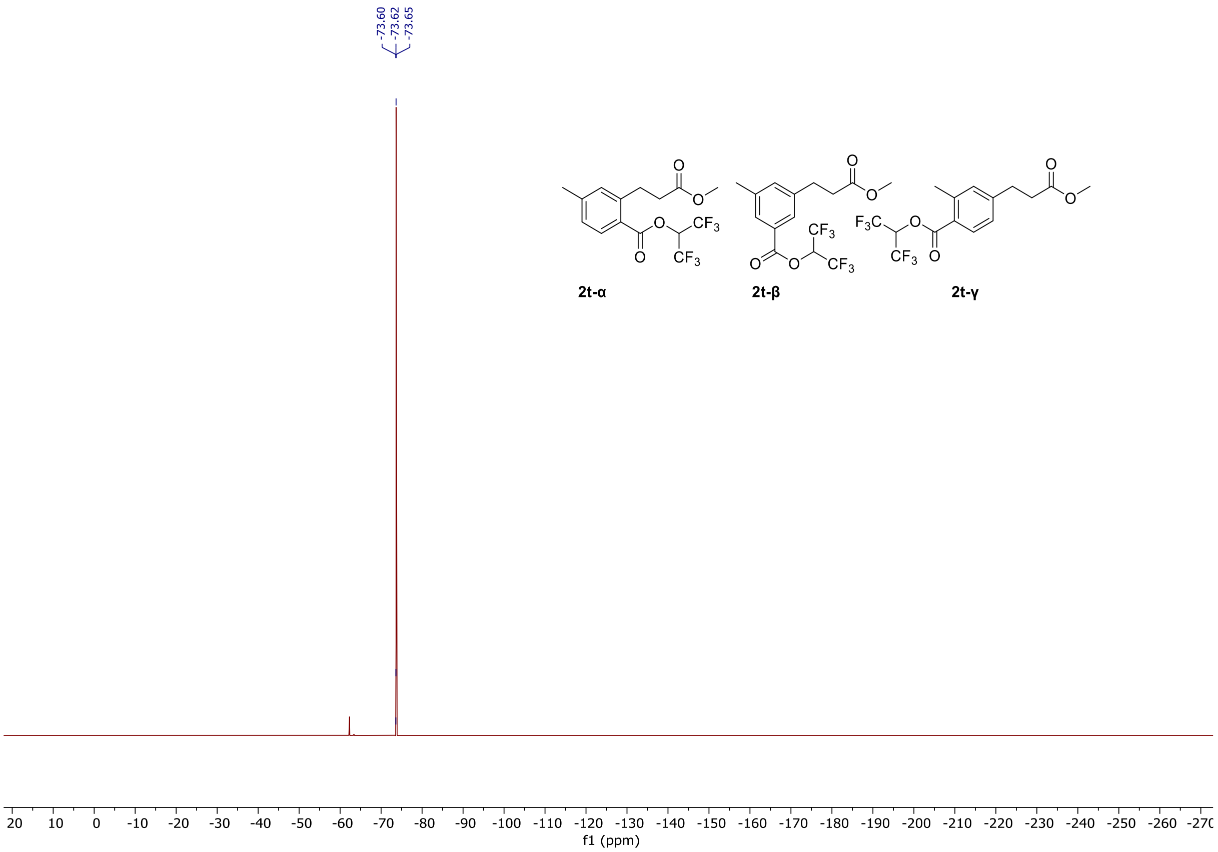

(2-Methoxyethyl)-3-methylbenzoic acid (4u)

^{1}H-NMR in $CDCl_3$

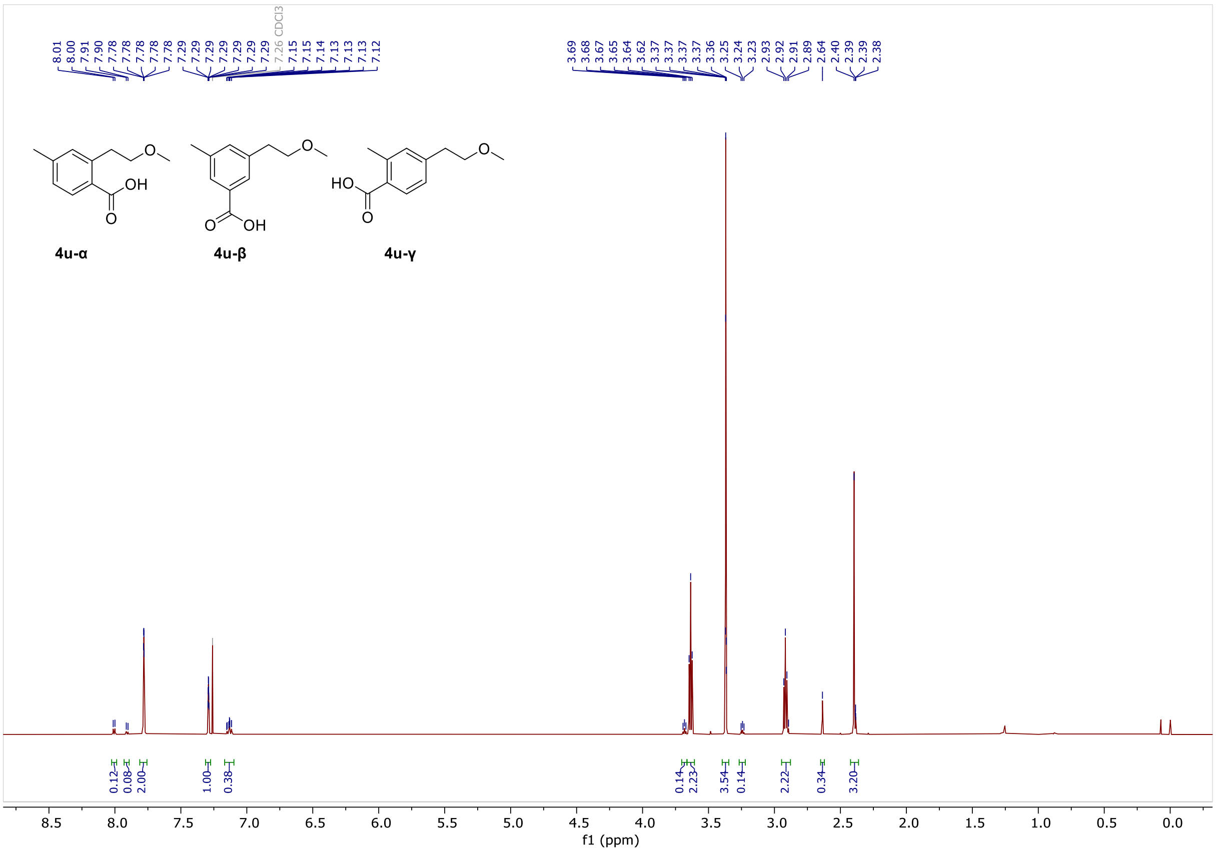

^{13}C-NMR in $CDCl_3$

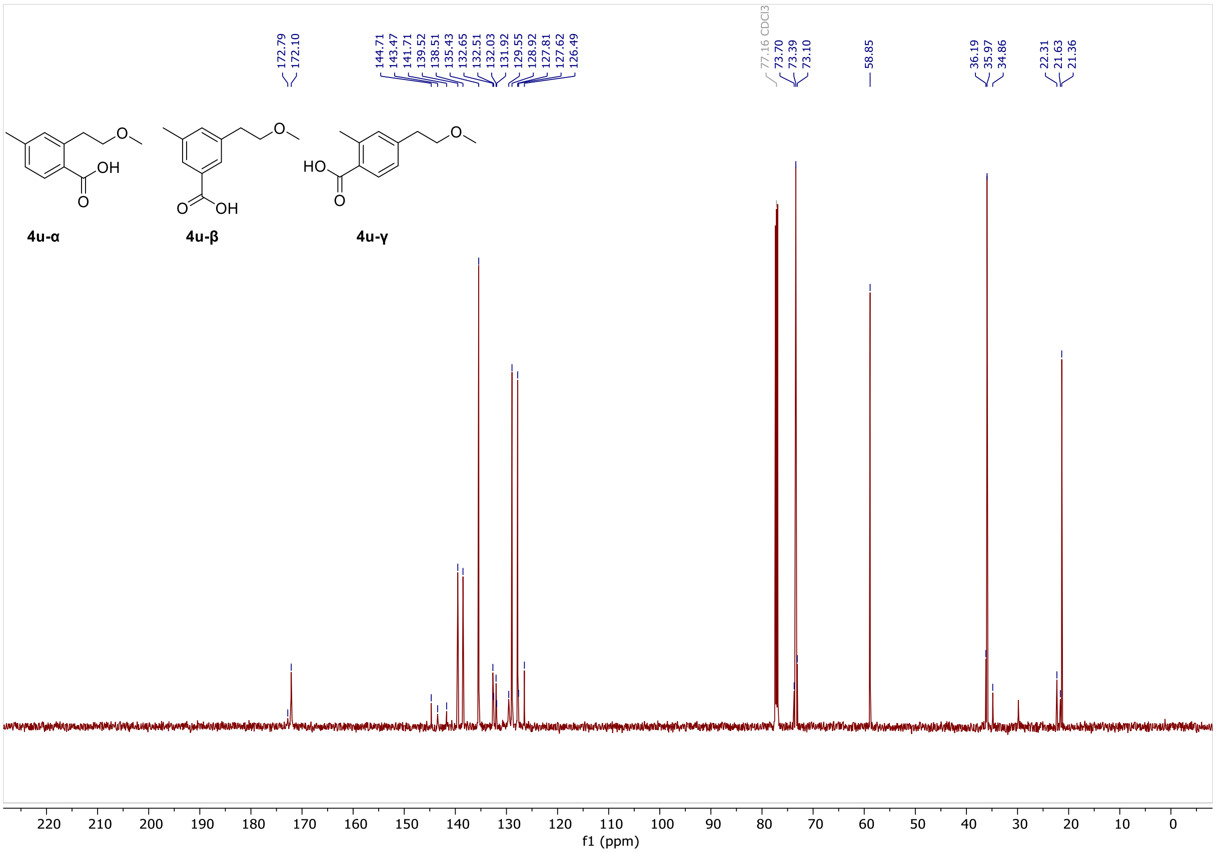

3-Fluoro-1-methylbenzoic acid (4v)

^{1}H-NMR in $CDCl_3$

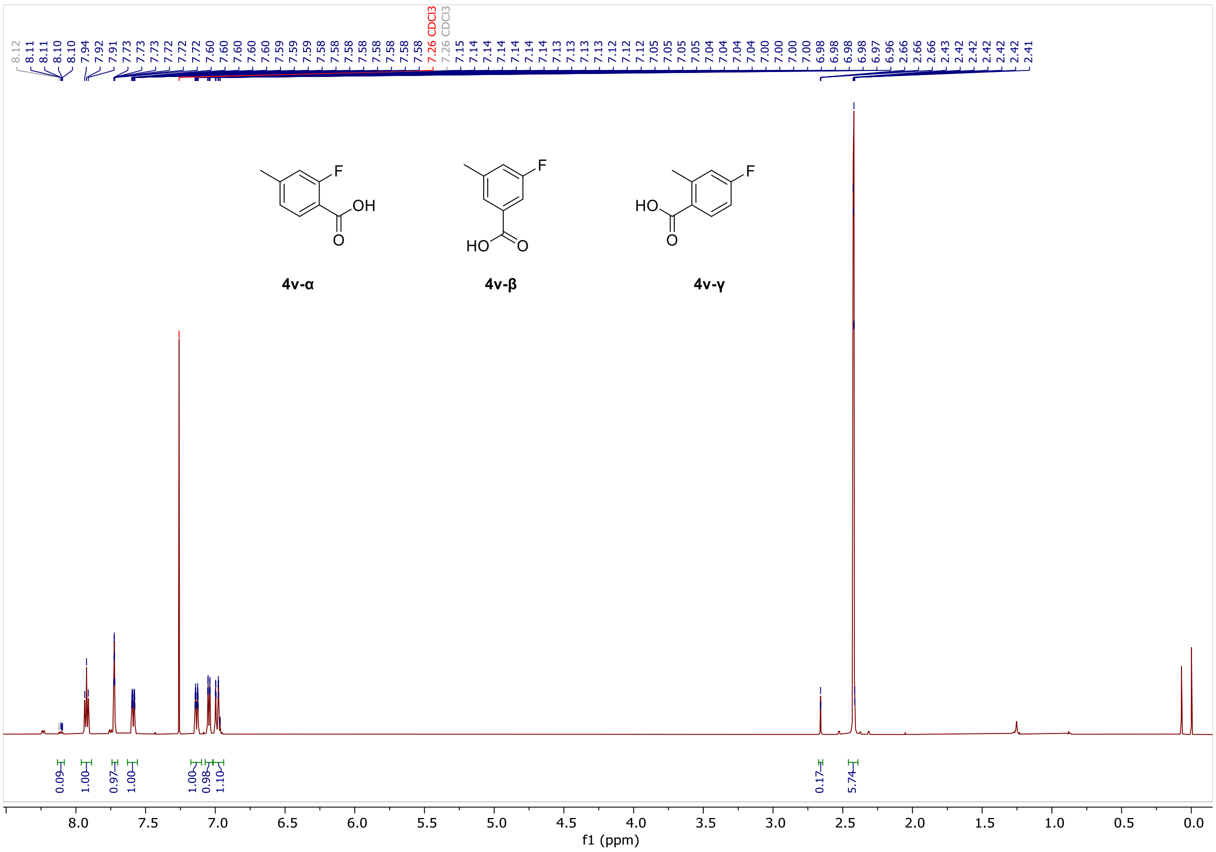

^{13}C-NMR in $CDCl_3$

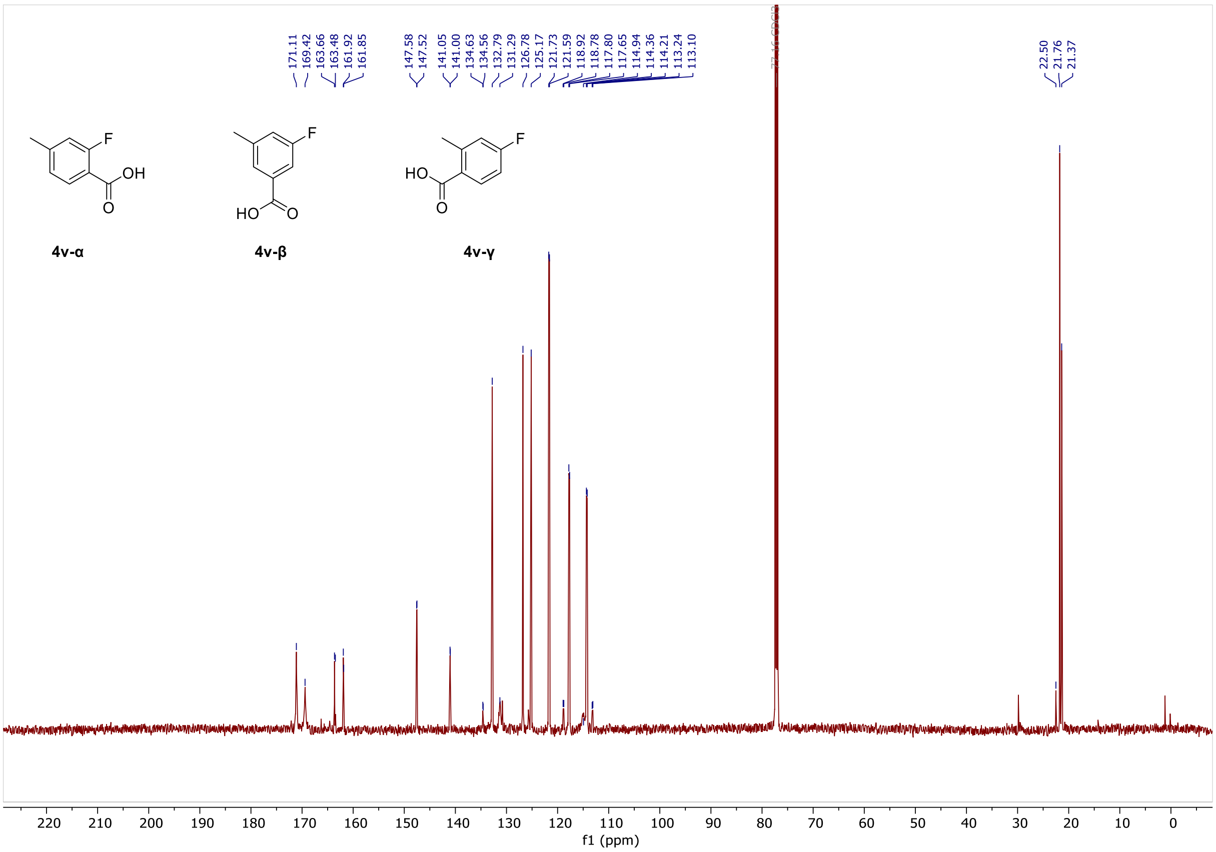

^{19}F-NMR in $CDCl_3$

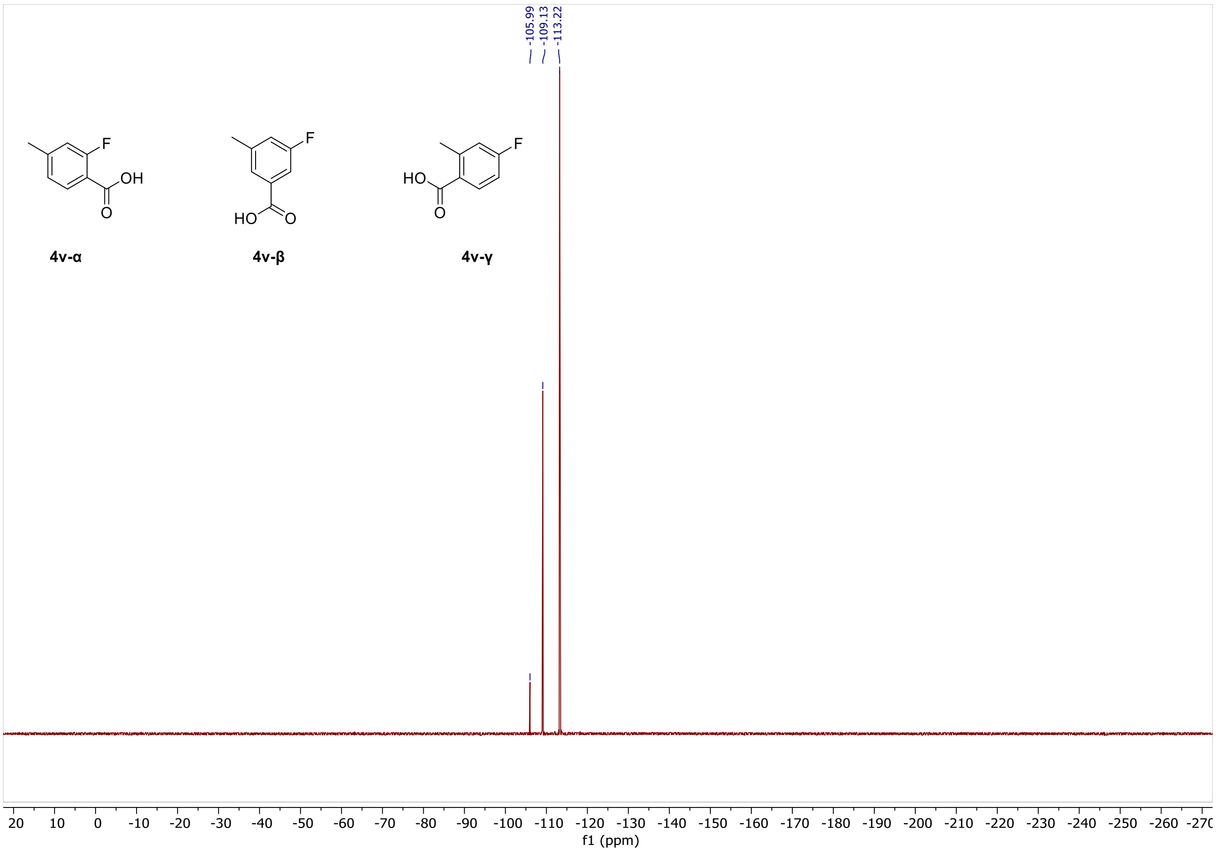

3-methyl-5-(trifluoromethyl)benzoic acid (4w)

^{1}H-NMR in $CDCl_3$

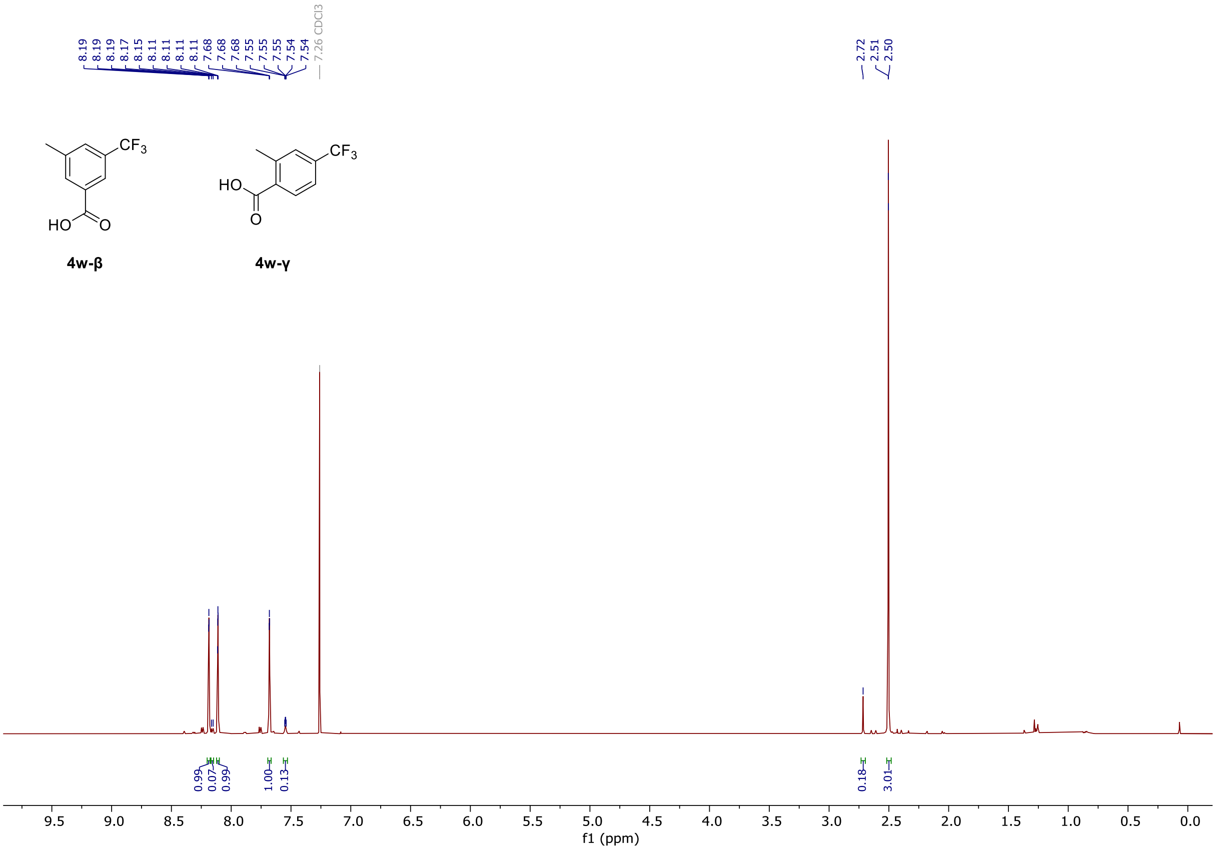

^{13}C-NMR in $CDCl_3$

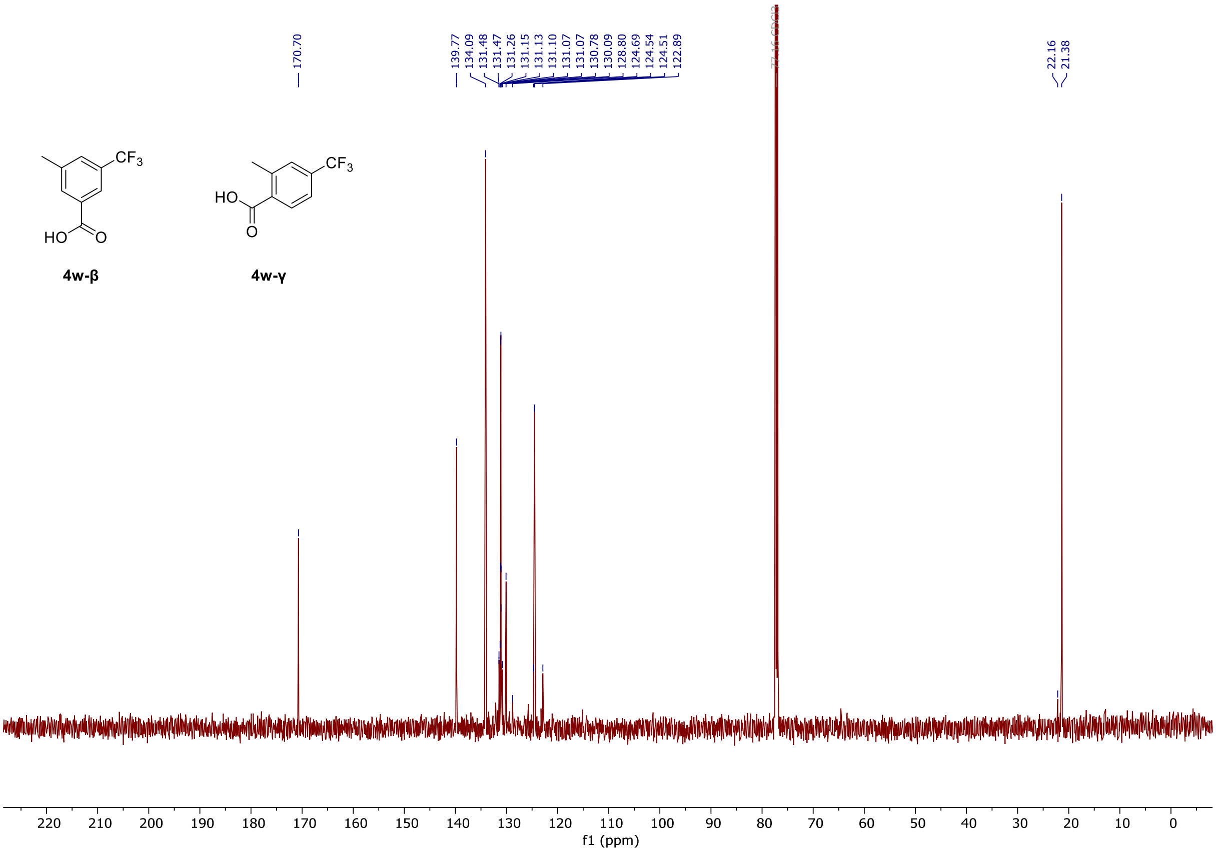

^{19}F-NMR in $CDCl_3$

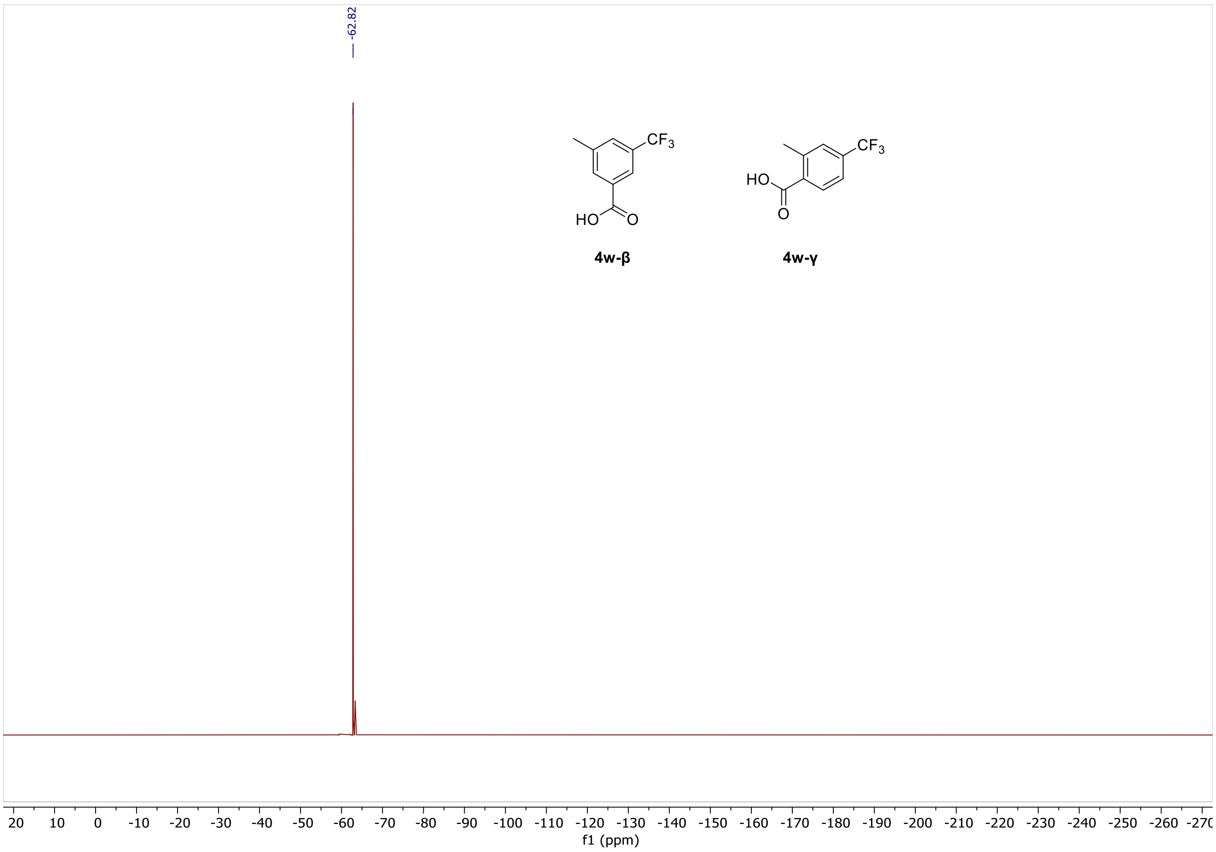

4-Chloro-1-methylbenzoic acid (4x)

^{1}H-NMR in $CDCl_3$

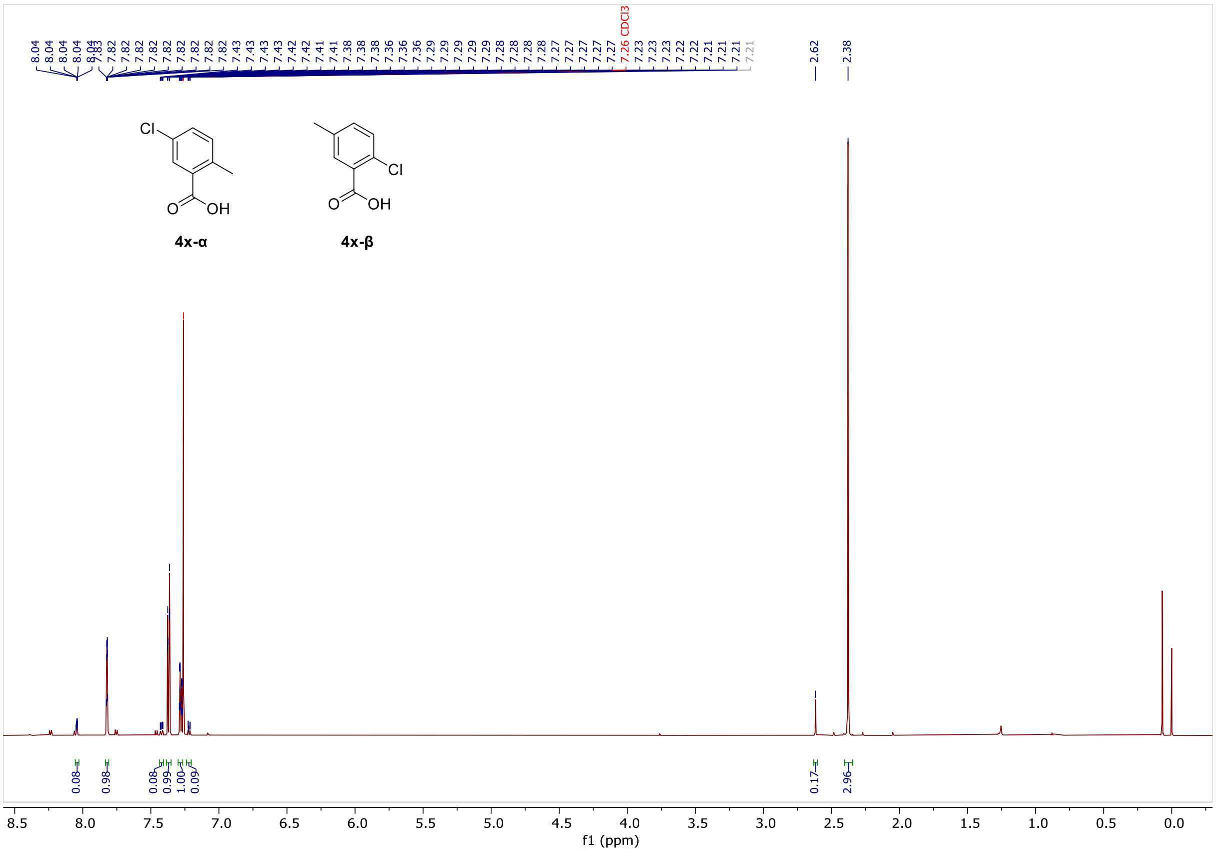

^{13}C-NMR in $CDCl_3$

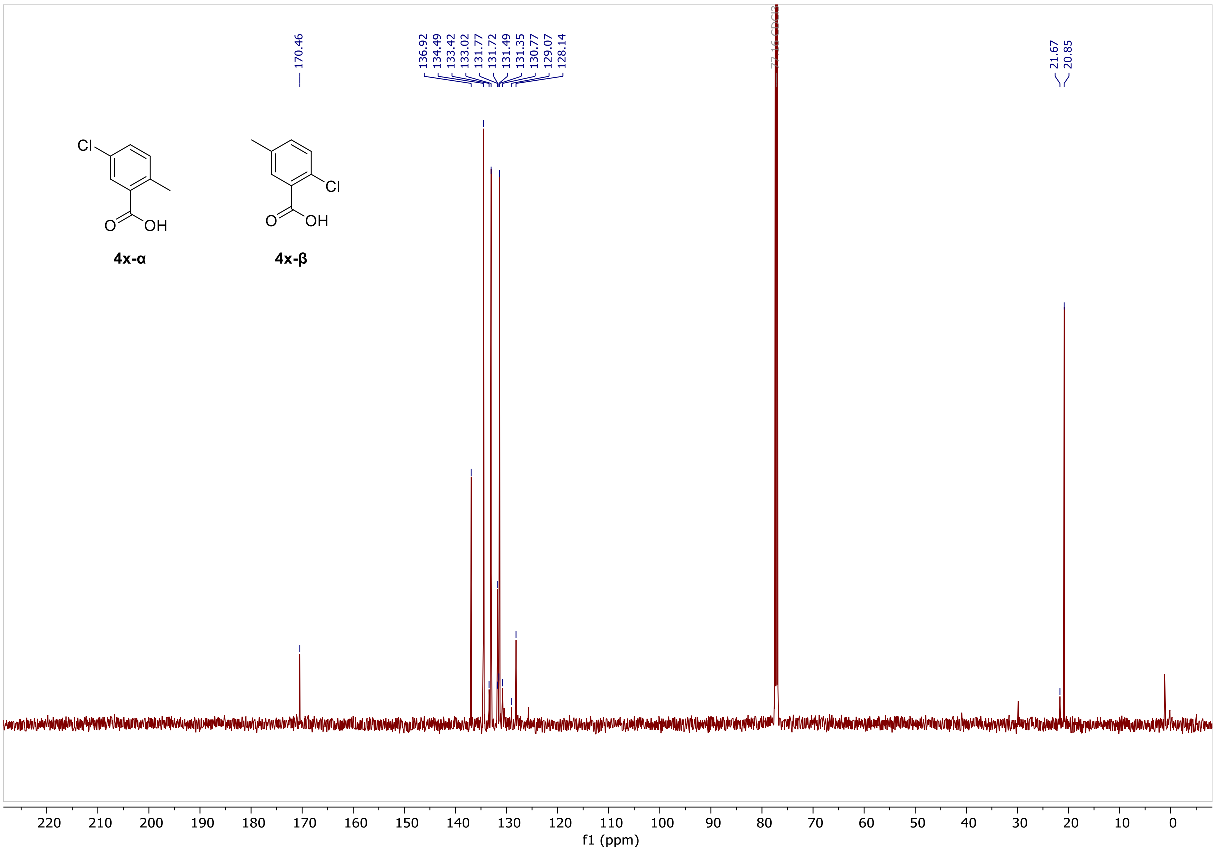

1,3-Diisopropyl-2-methoxybenzoic acid (4y)

^{1}H-NMR in $CDCl_3$

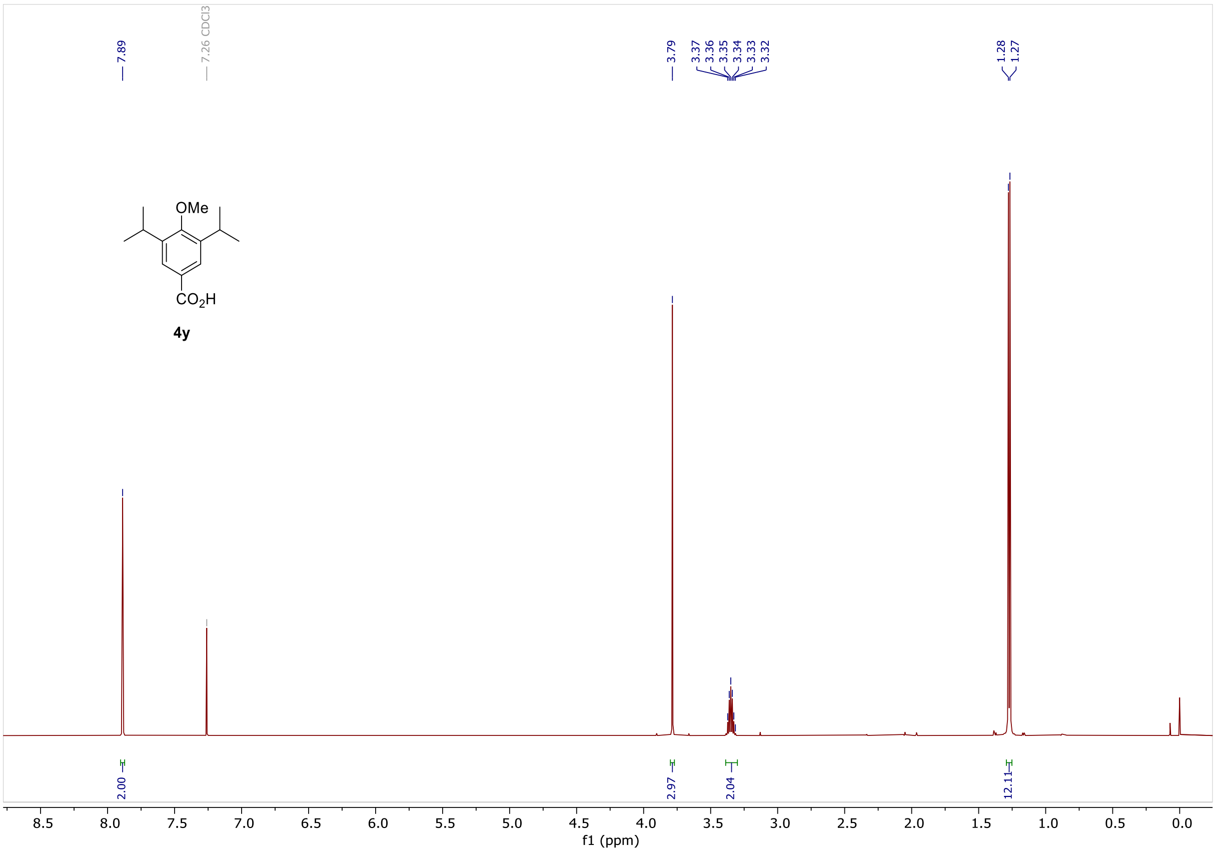

^{13}C-NMR in $CDCl_3$

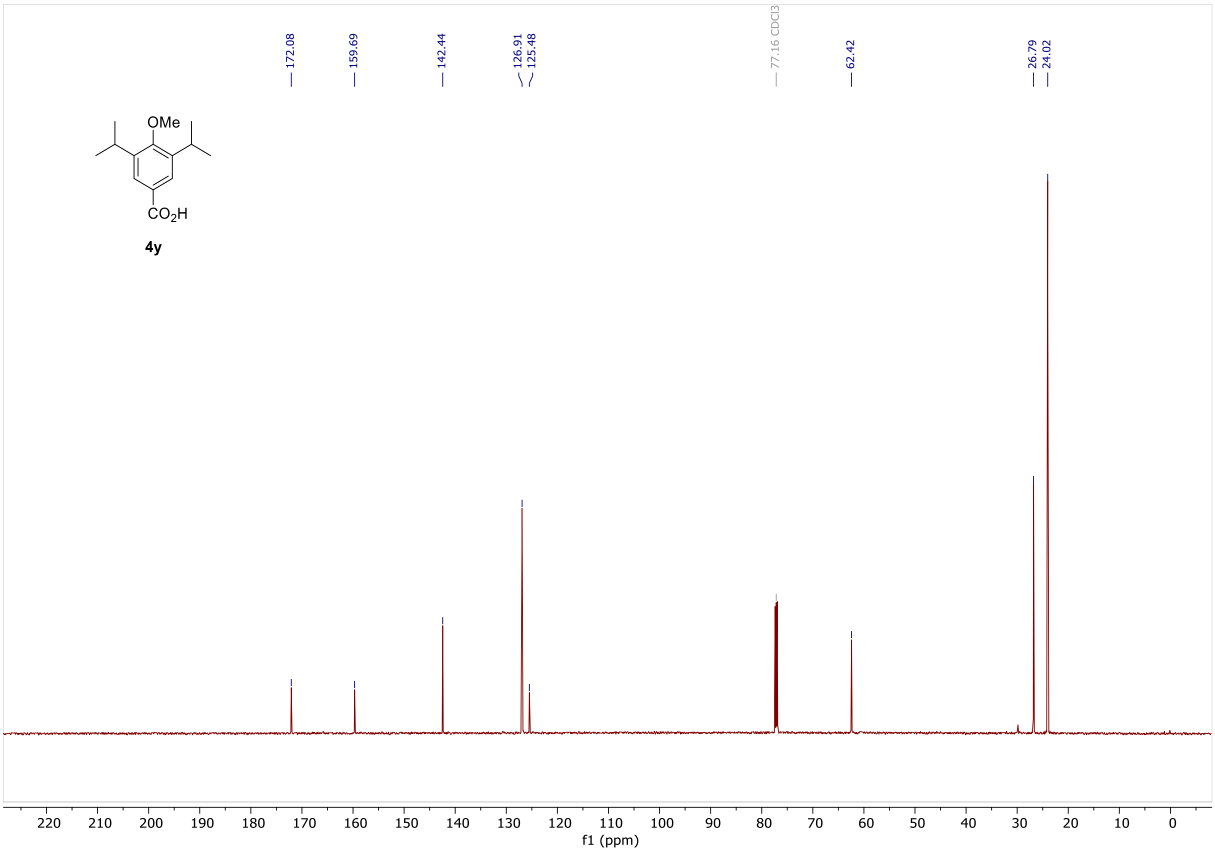

3,4-Dimethyl-5-(((1,3,3-trimethylbicyclo[2.2.1]heptan-2-yl)oxy)methyl)benzoic acid (4z)

^{1}H-NMR in $CDCl_3$

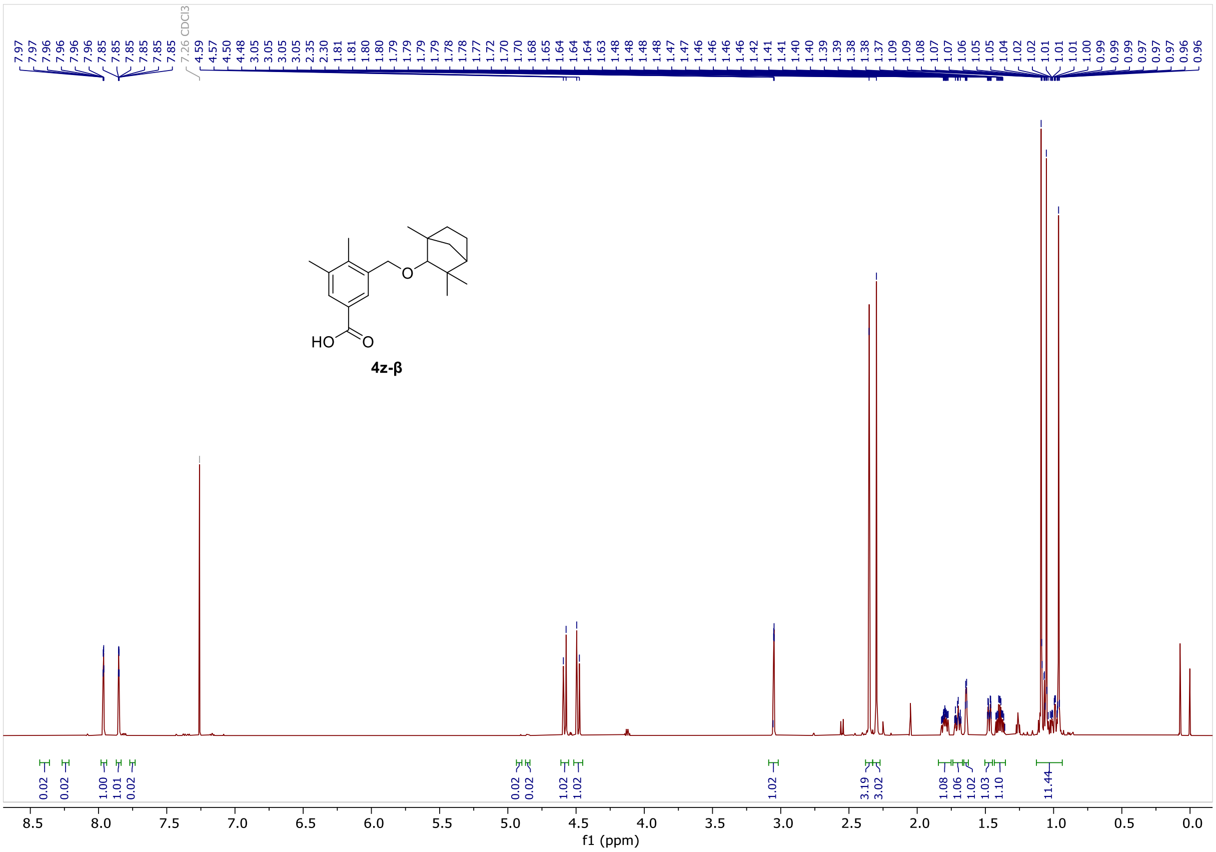

^{13}C-NMR in $CDCl_3$

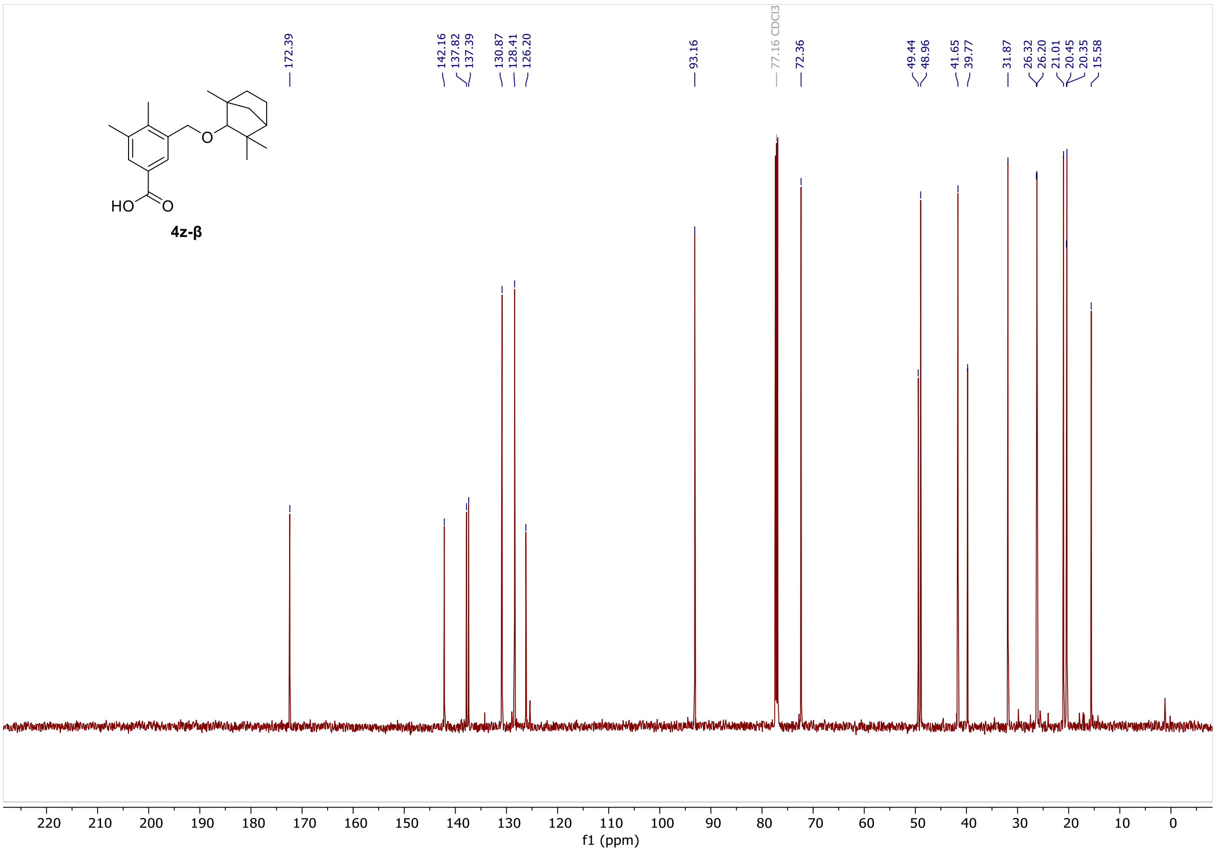

2-Fluoro-1,3-dimethylbenzoic acid (4aa)

^{1}H-NMR in $CDCl_3$

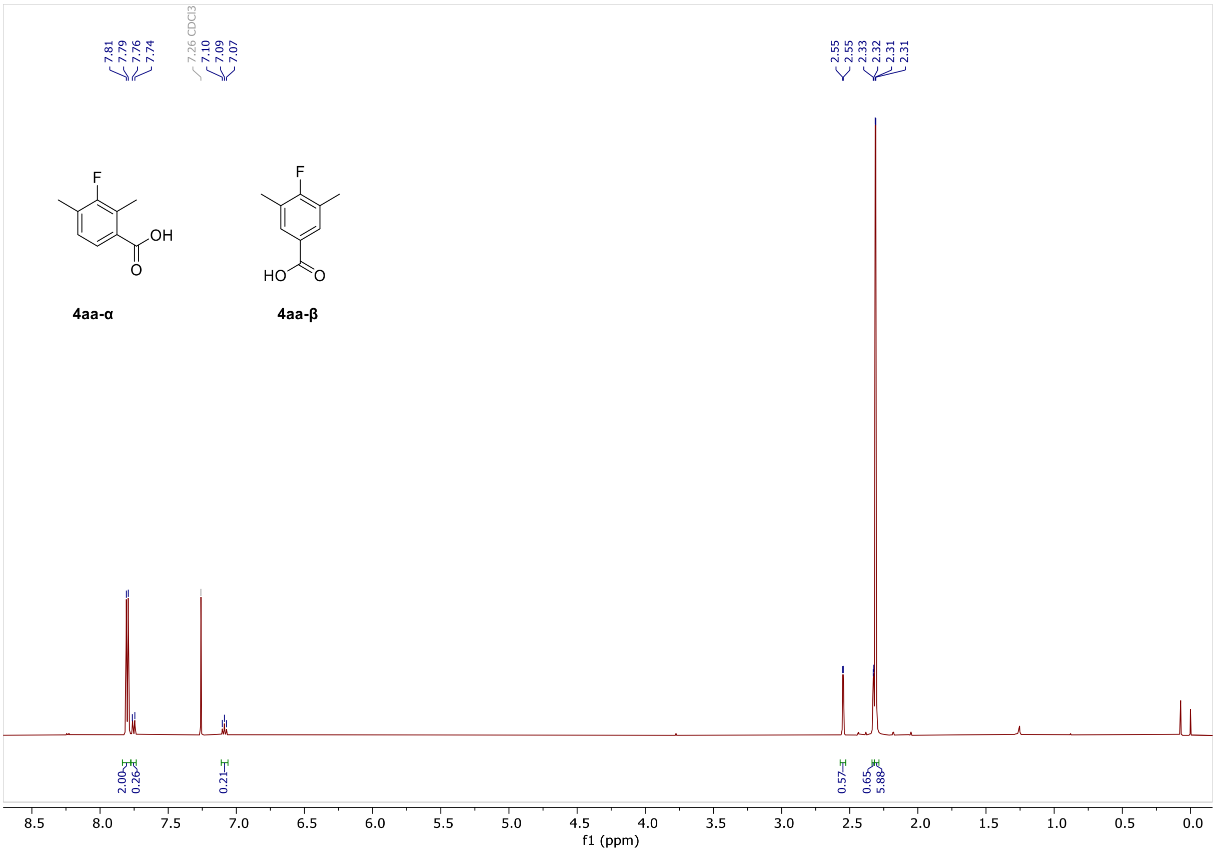

^{13}C-NMR in $CDCl_3$

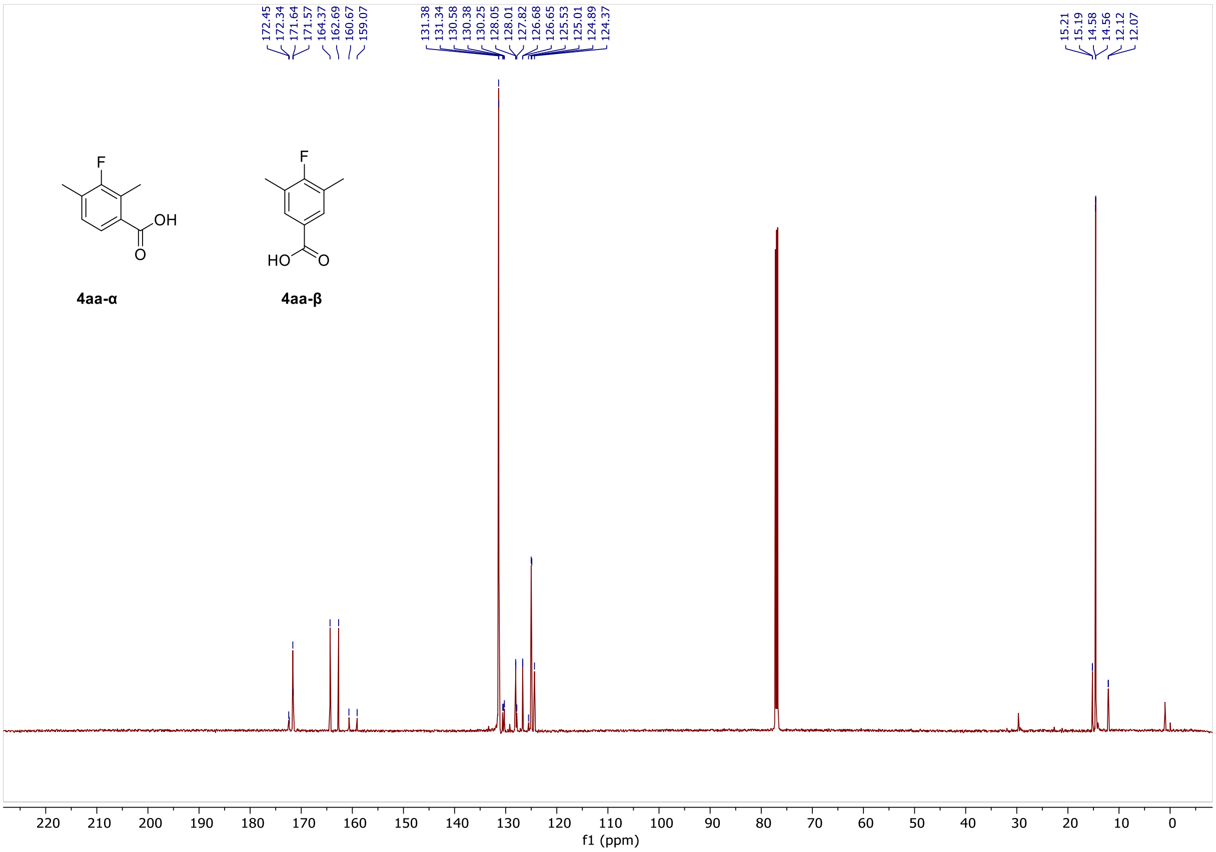

^{19}F-NMR in $CDCl_3$

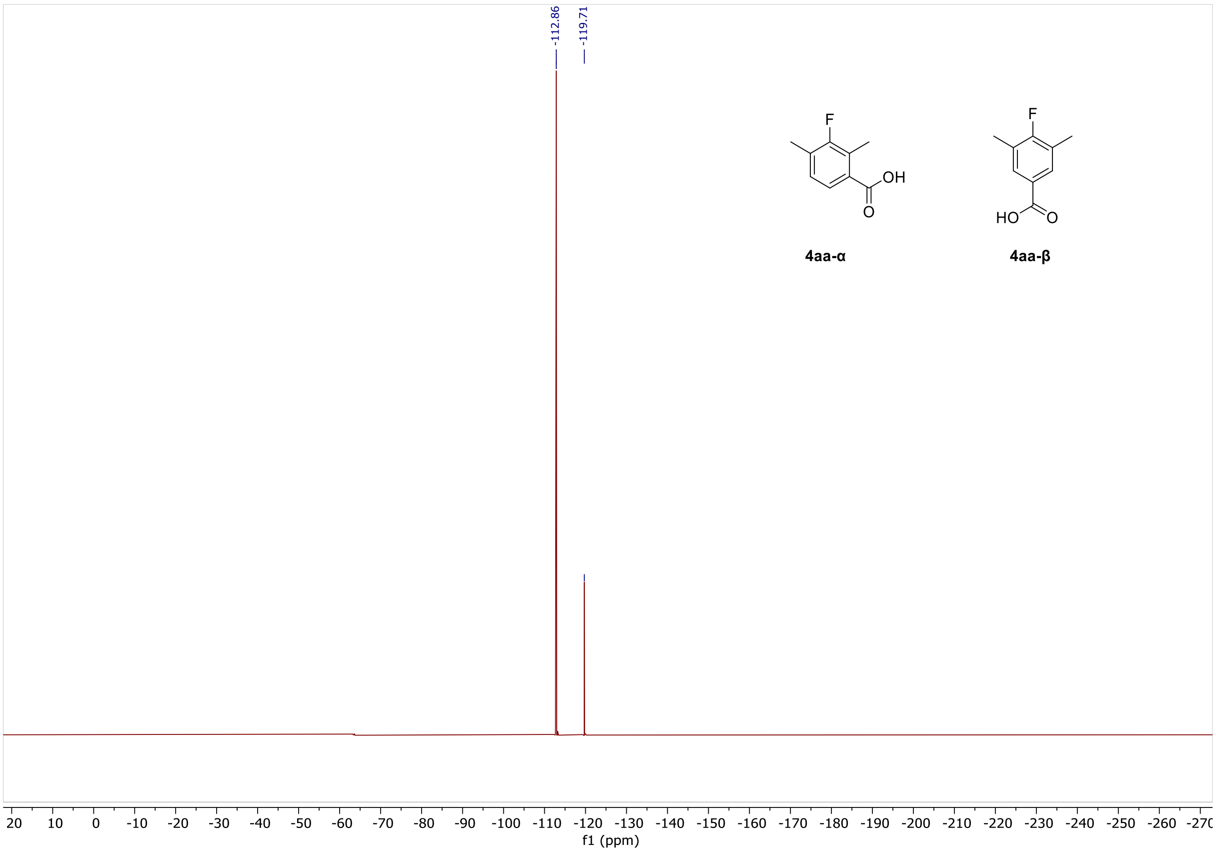

2-Bromo-1,3-dimethylbenzoic acid (4ab)

^{1}H-NMR in DMSO-d_6

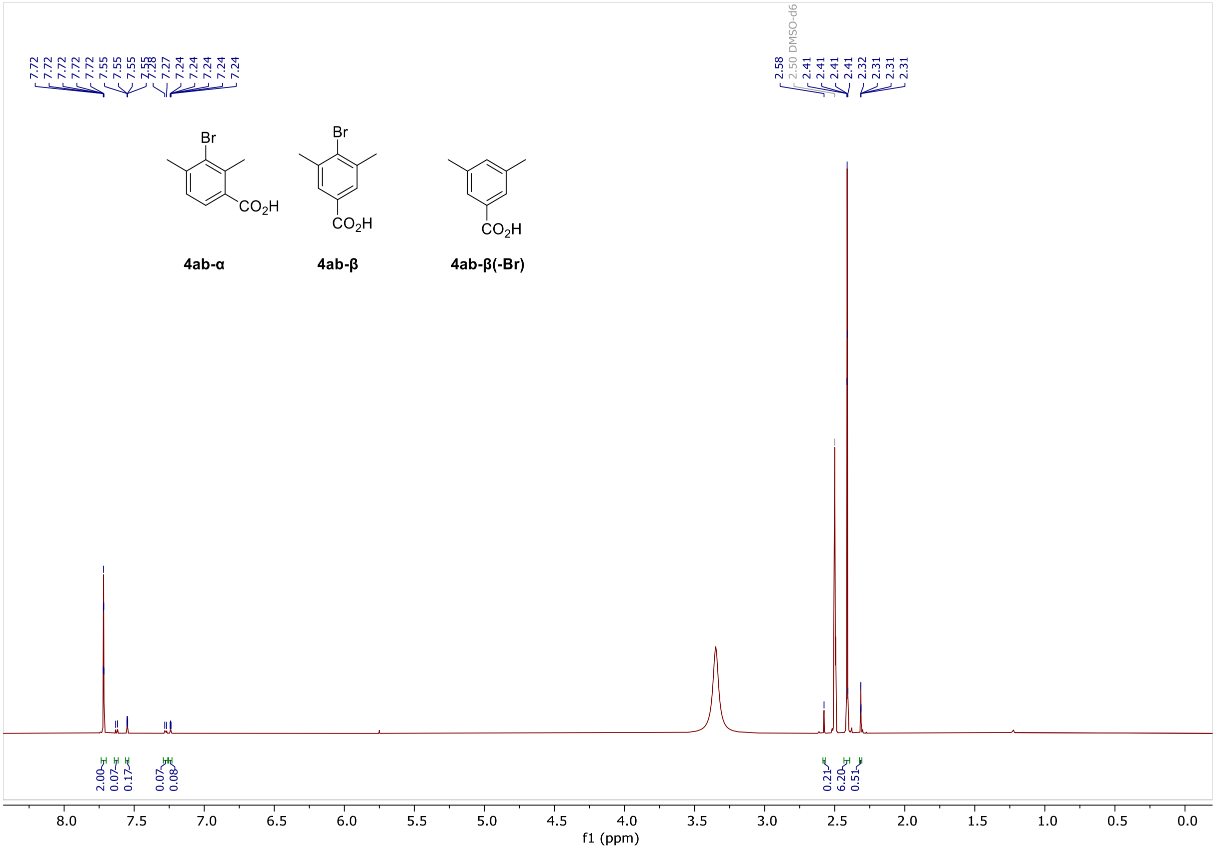

^{13}C-NMR in DMSO-d_6

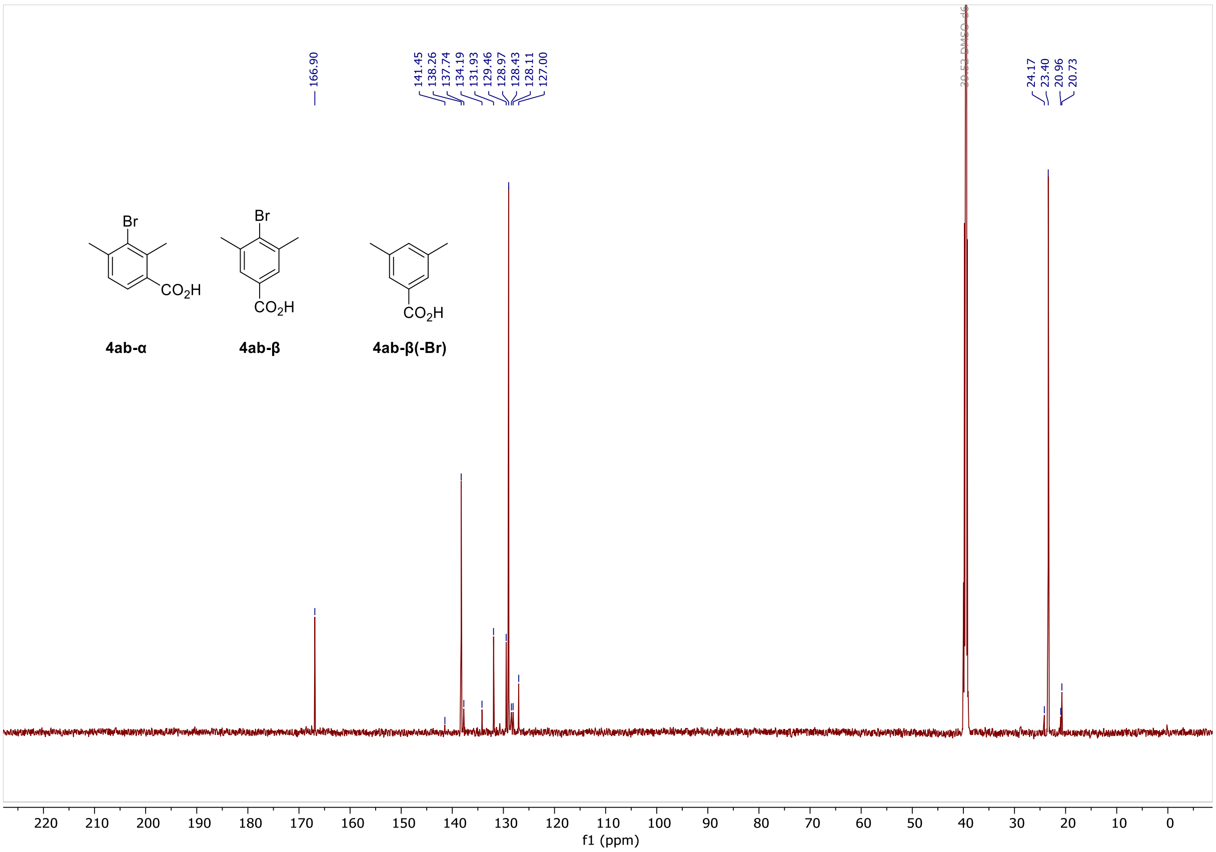

5-(1,1,1,3,3,3-Hexafluoropropan-2-yl) 3-methyl 2-methyl-4'-(trifluoromethoxy)-[1,1'-biphenyl]-3,5-dicarboxylate (2ac)

^{1}H NMR spectrum in $CDCl_3$

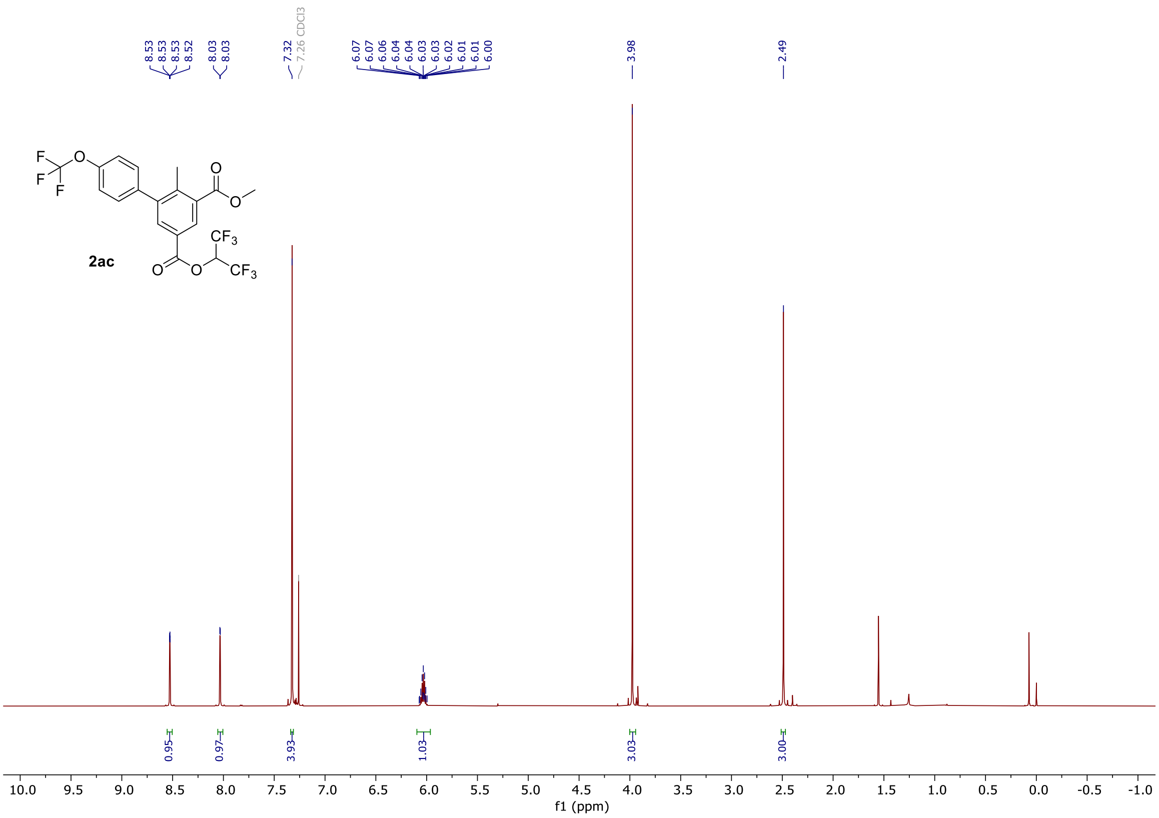

^{13}C NMR spectrum in $CDCl_3$

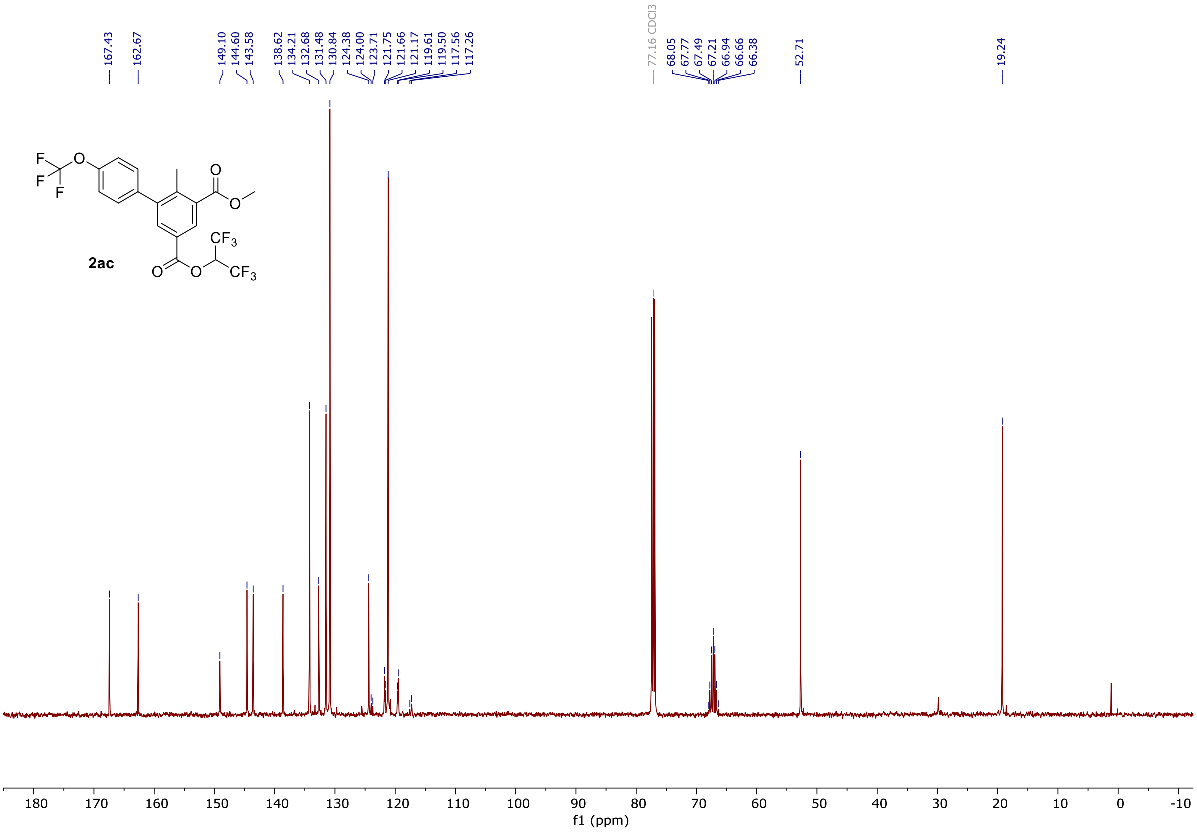

^{19}F NMR spectrum in CDCl$_3$

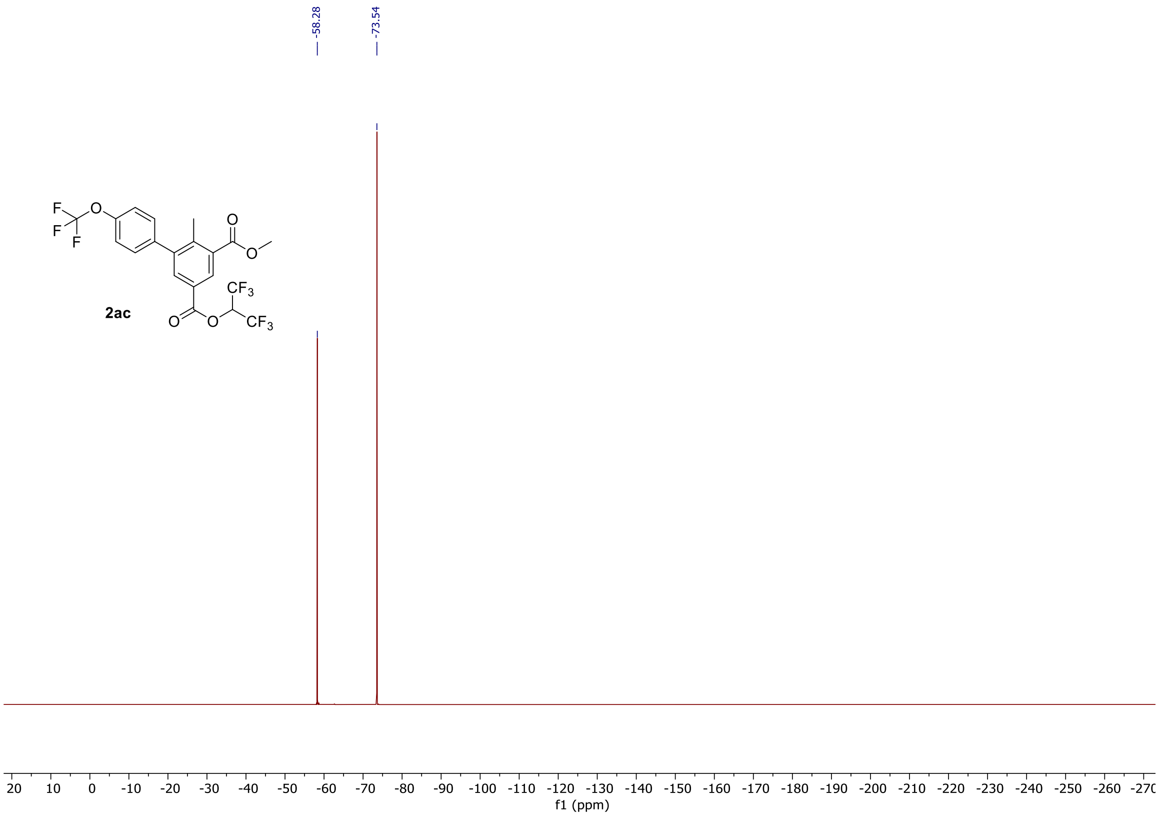

7.5. Additional substrates

1-(Diisopropylcarbamoyl)-2-methylbenzoic acid (4ad)

^{1}H-NMR in DMSO-d$_6$

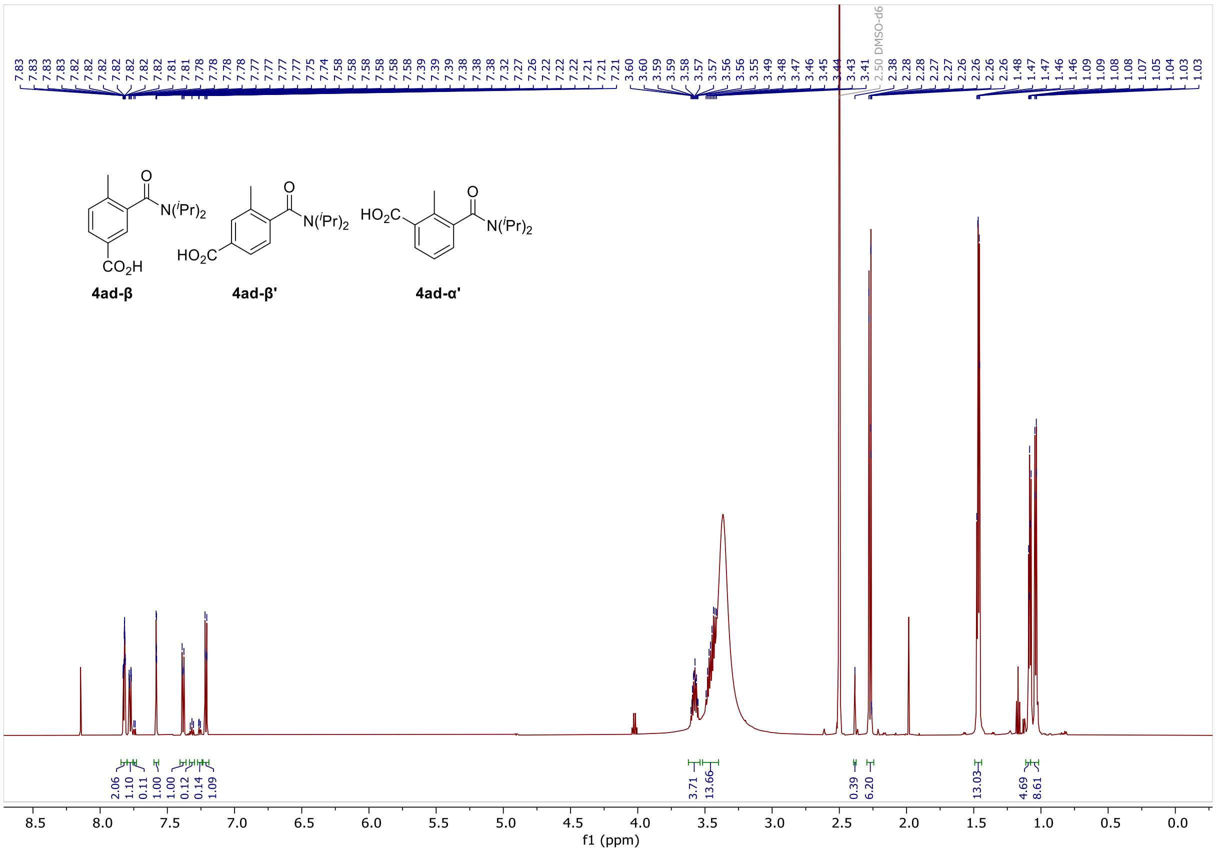

^{13}C-NMR in DMSO-d$_6$

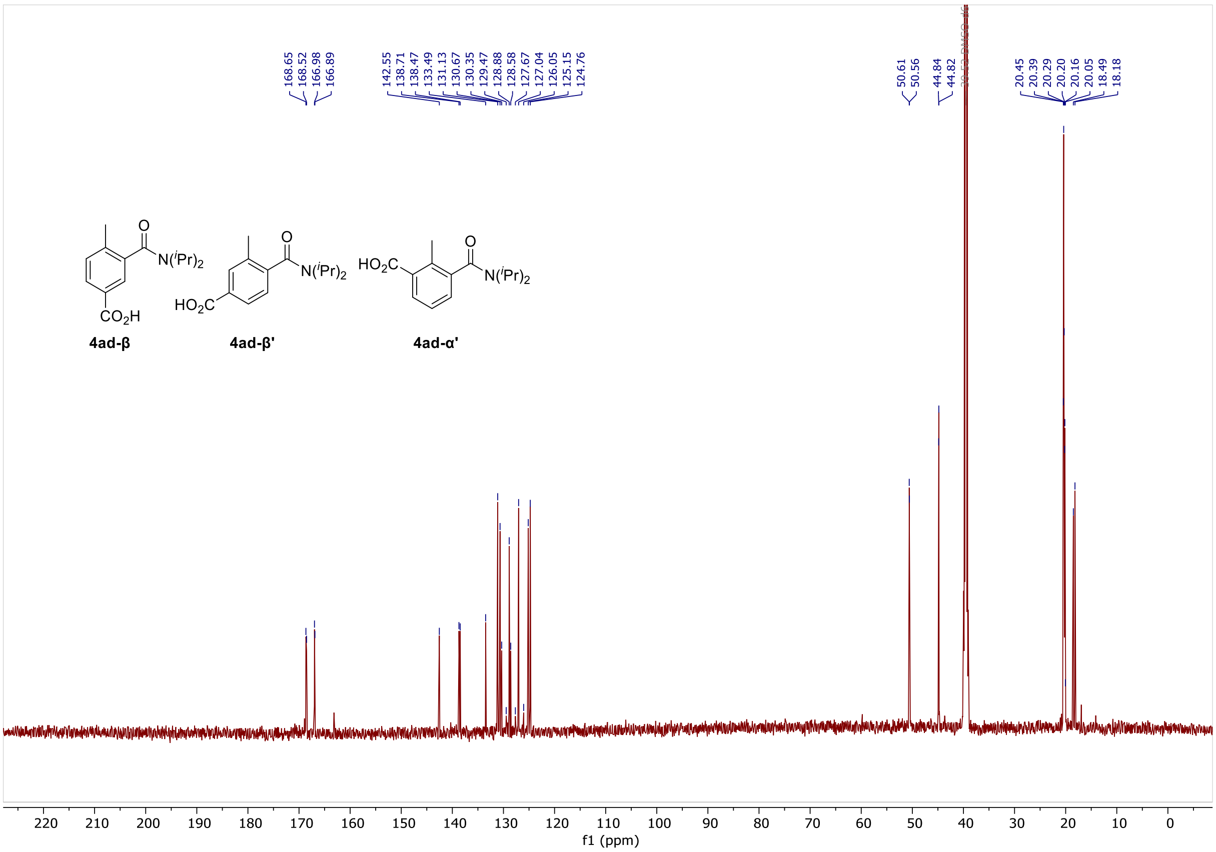

3-Bromo-1-methylbenzoic acid (4ae)

^{1}H-NMR in $CDCl_3$

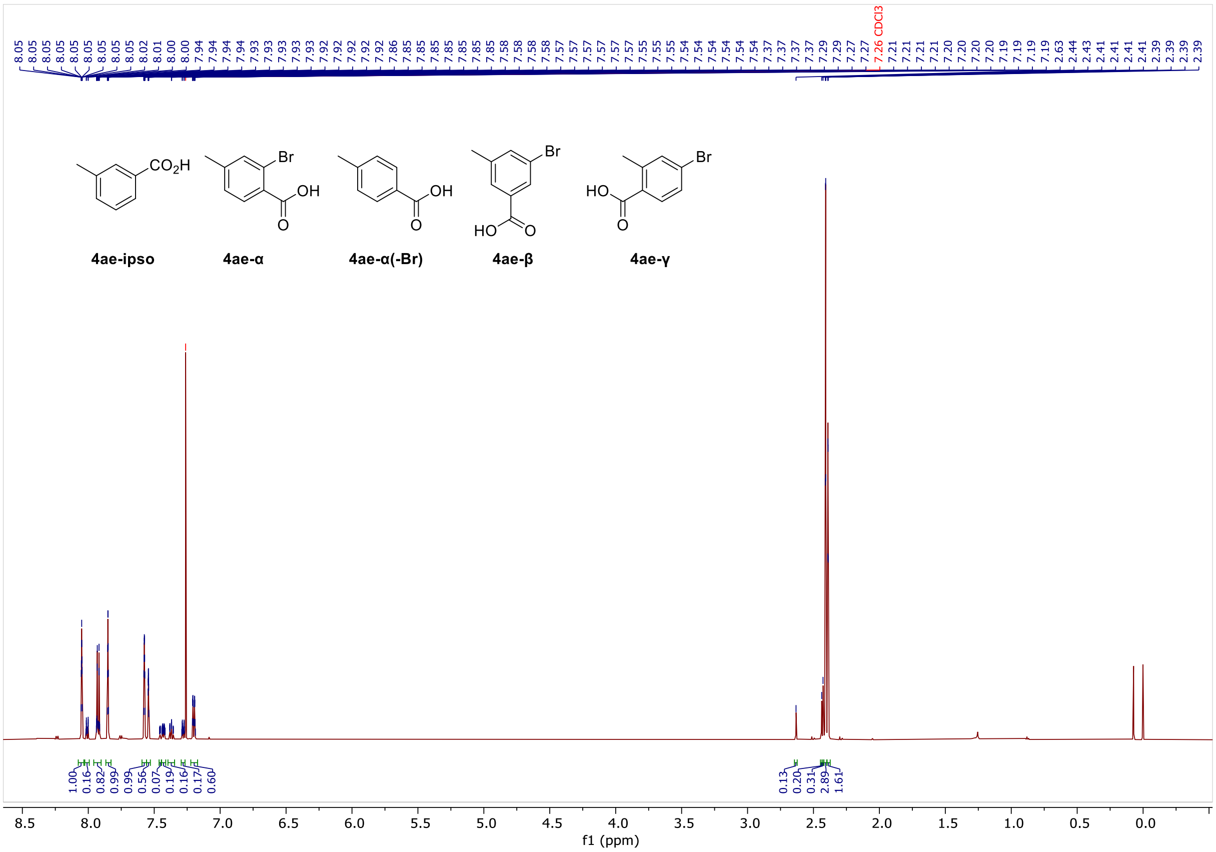

^{13}C-NMR in $CDCl_3$

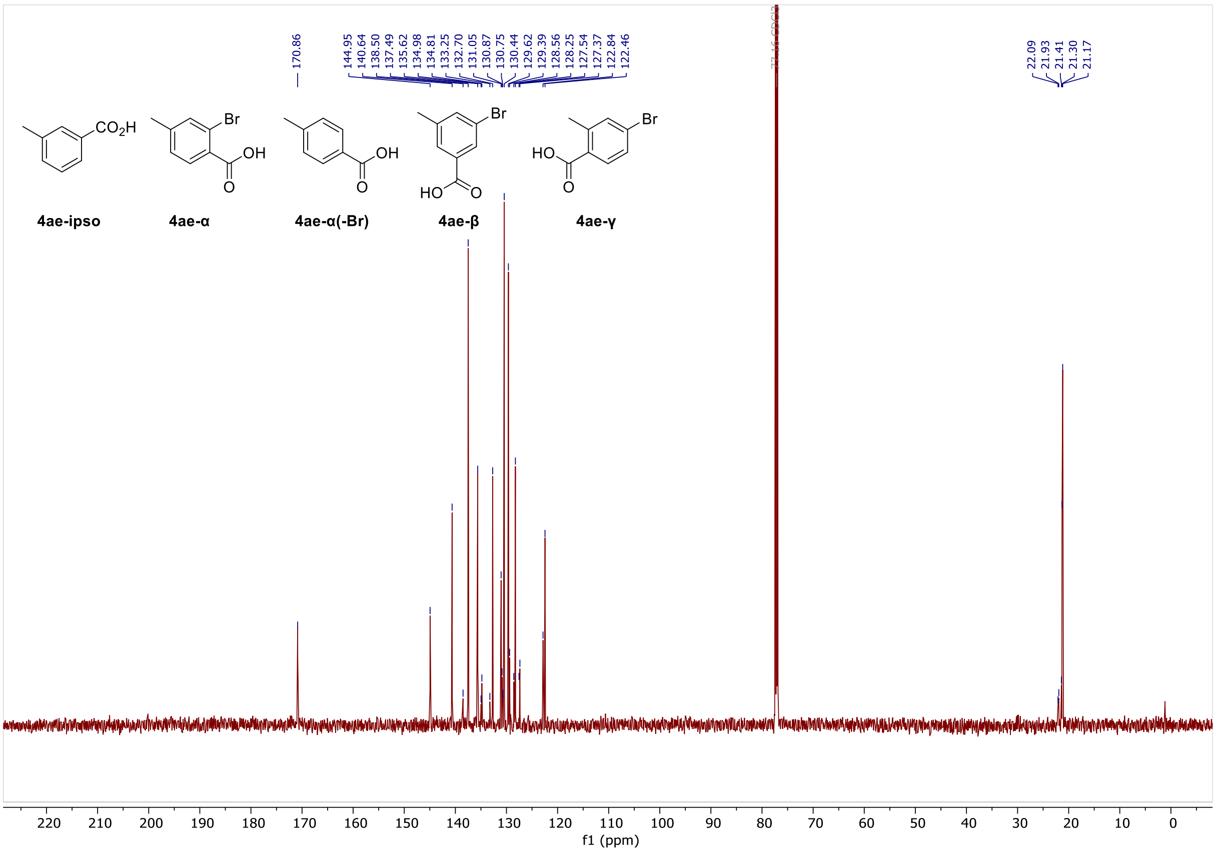

Ethylbenzoic acid from a reaction in 2,2,2-trifluoroethanol (4af)

^{1}H-NMR in $CDCl_3$

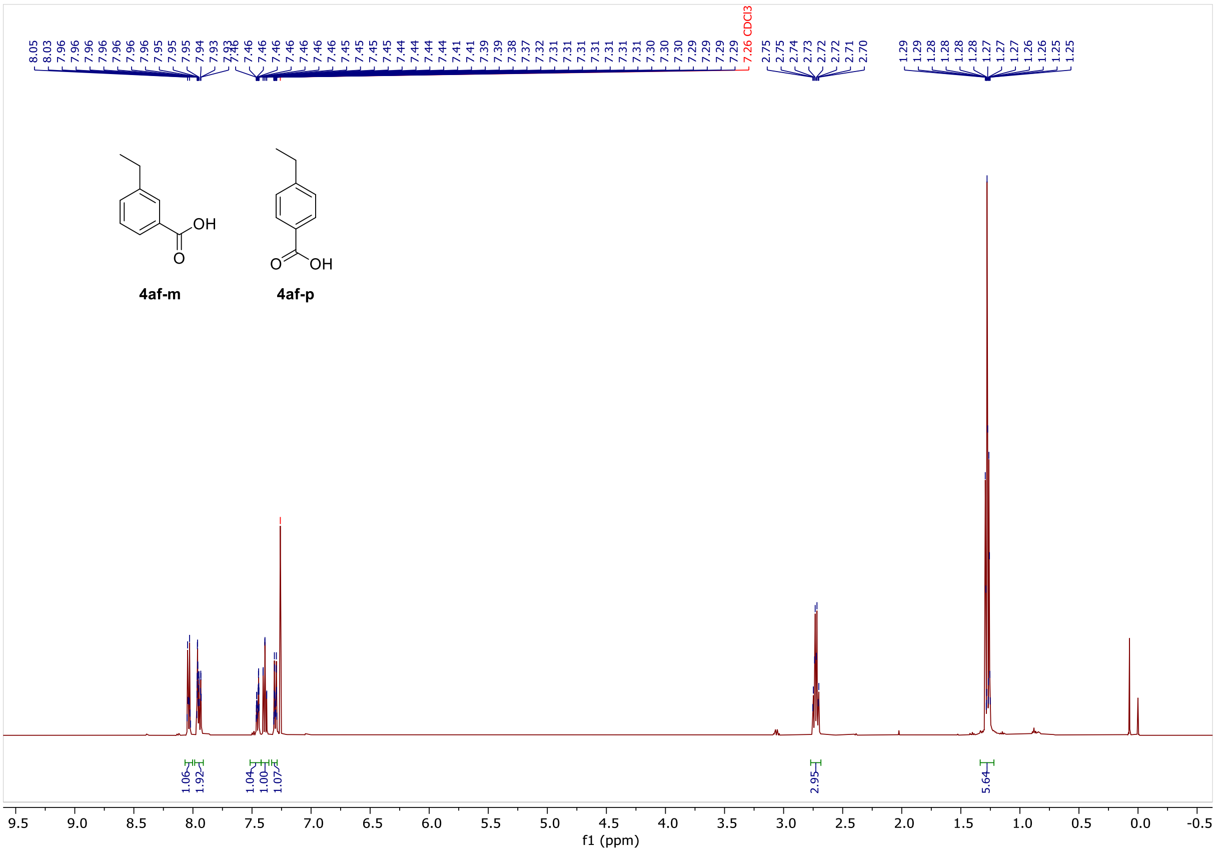

^{13}C-NMR in $CDCl_3$

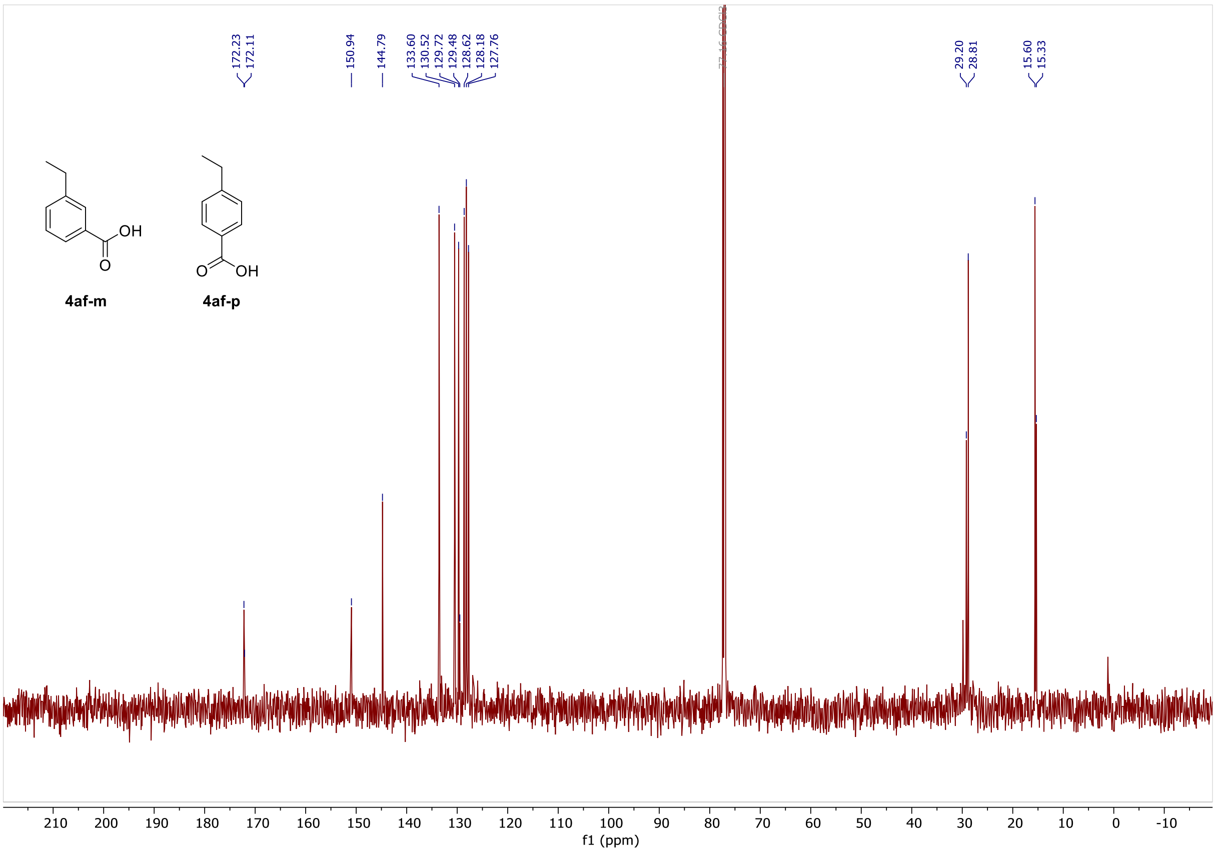

7.6 Follow-up Transformations

7.6.1 Scale-up to 1.2 mmol

1,1,1,3,3,3-Hexafluoropropanyl dimethylbenzoate (2m): Fraction 1

^{1}H NMR spectrum in $CDCl_3$

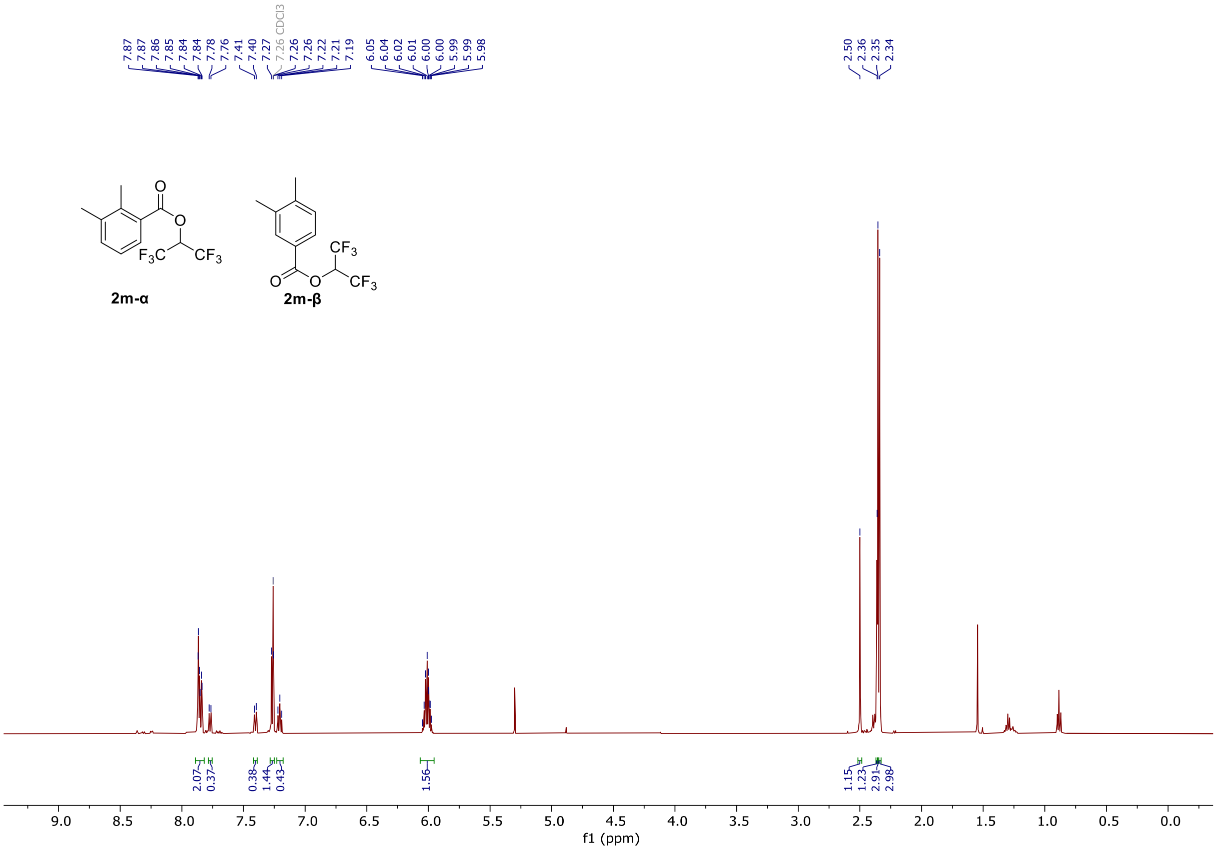

^{13}C NMR spectrum in $CDCl_3$: Fraction 1

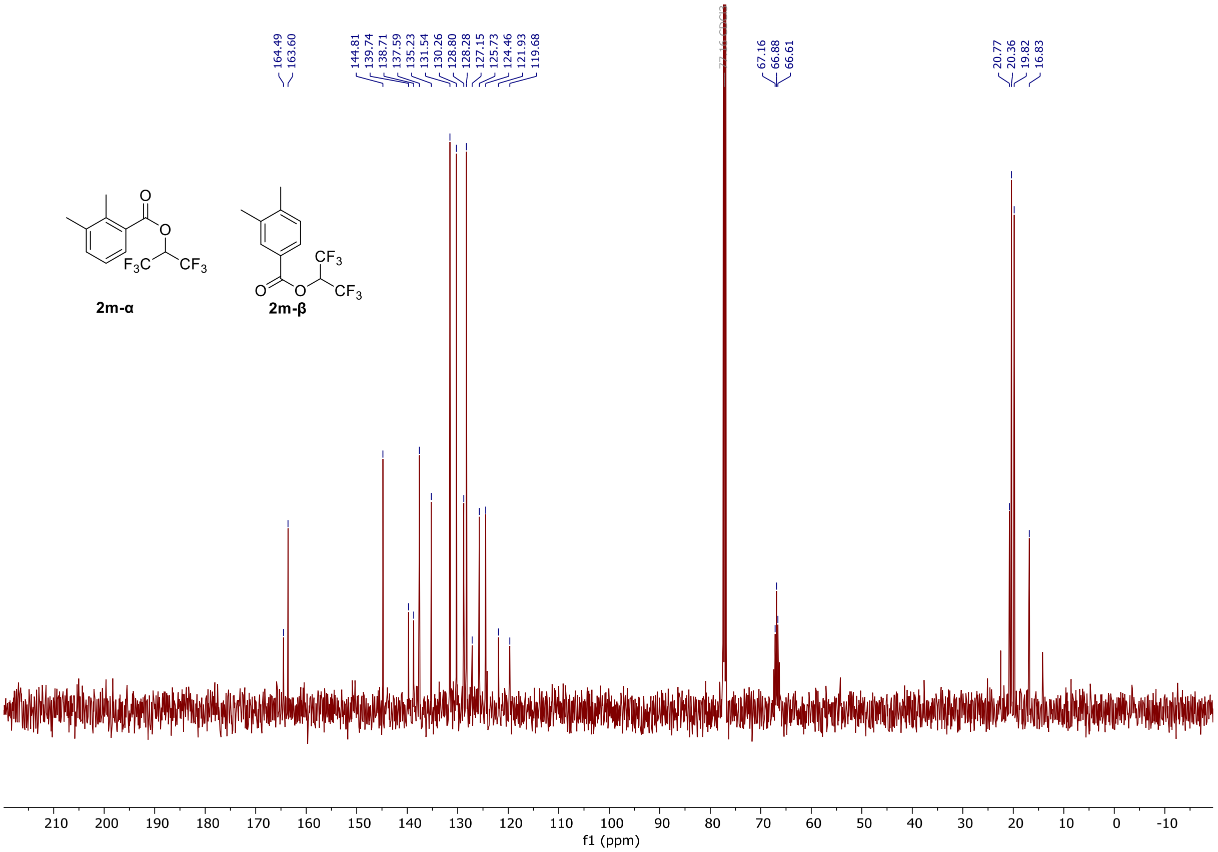

^{19}F NMR spectrum in $CDCl_3$: Fraction 1

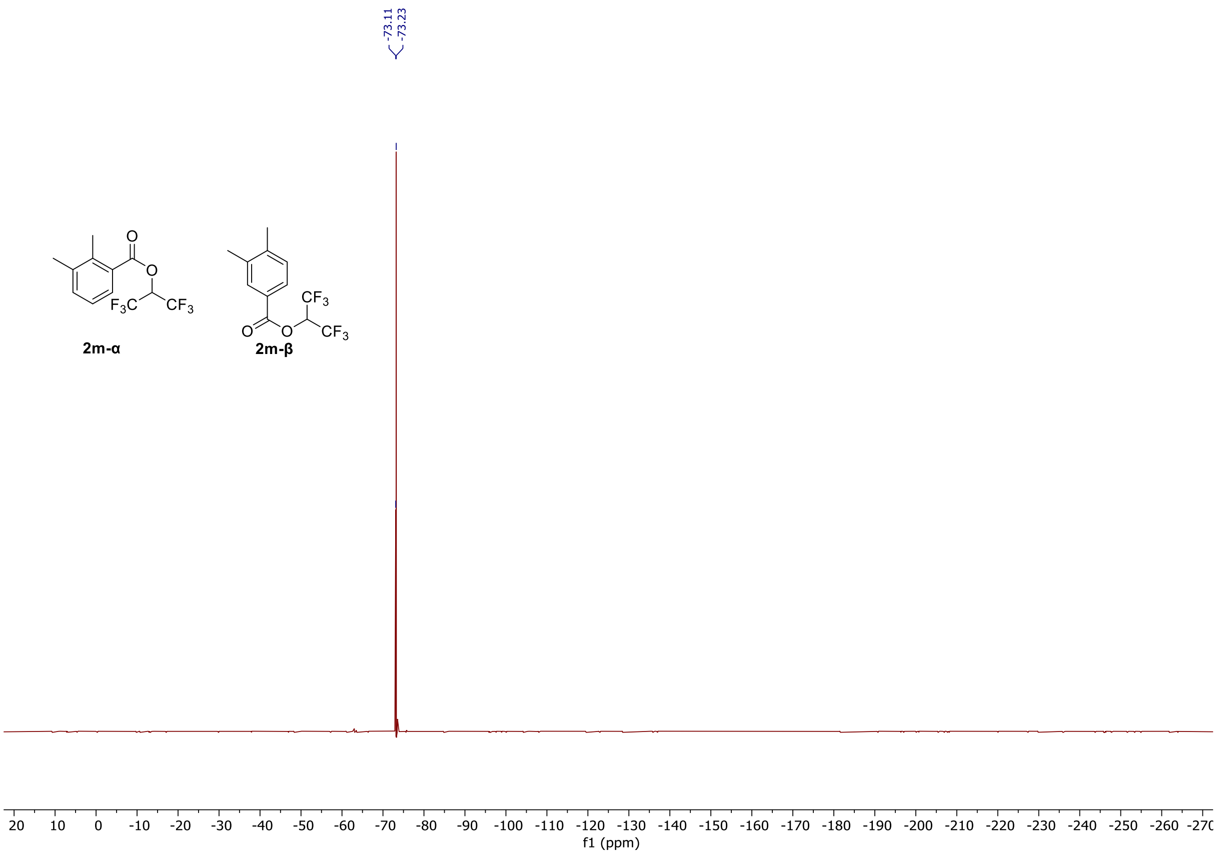

^{1}H NMR spectrum in $CDCl_3$: Fraction 2

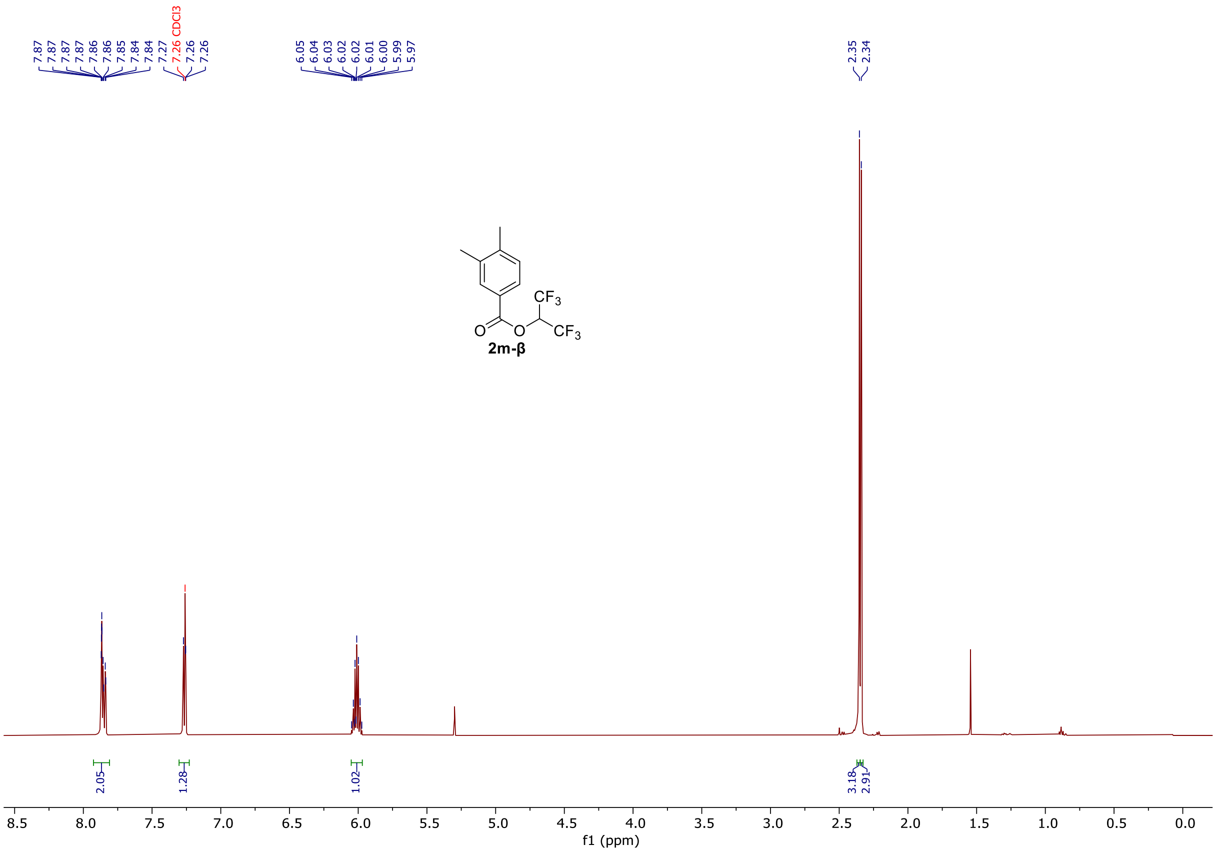

^{13}C NMR spectrum in $CDCl_3$: Fraction 2

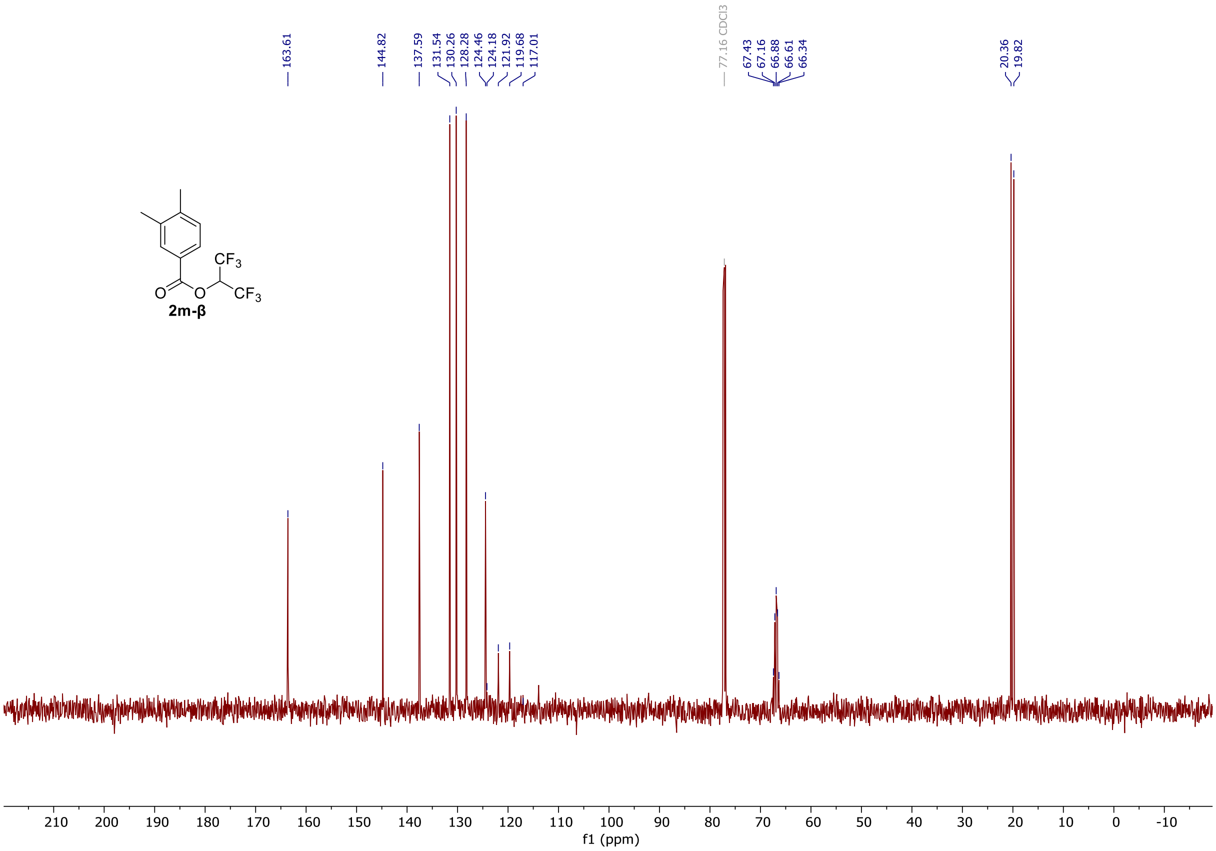

^{19}F NMR spectrum in $CDCl_3$: Fraction 2

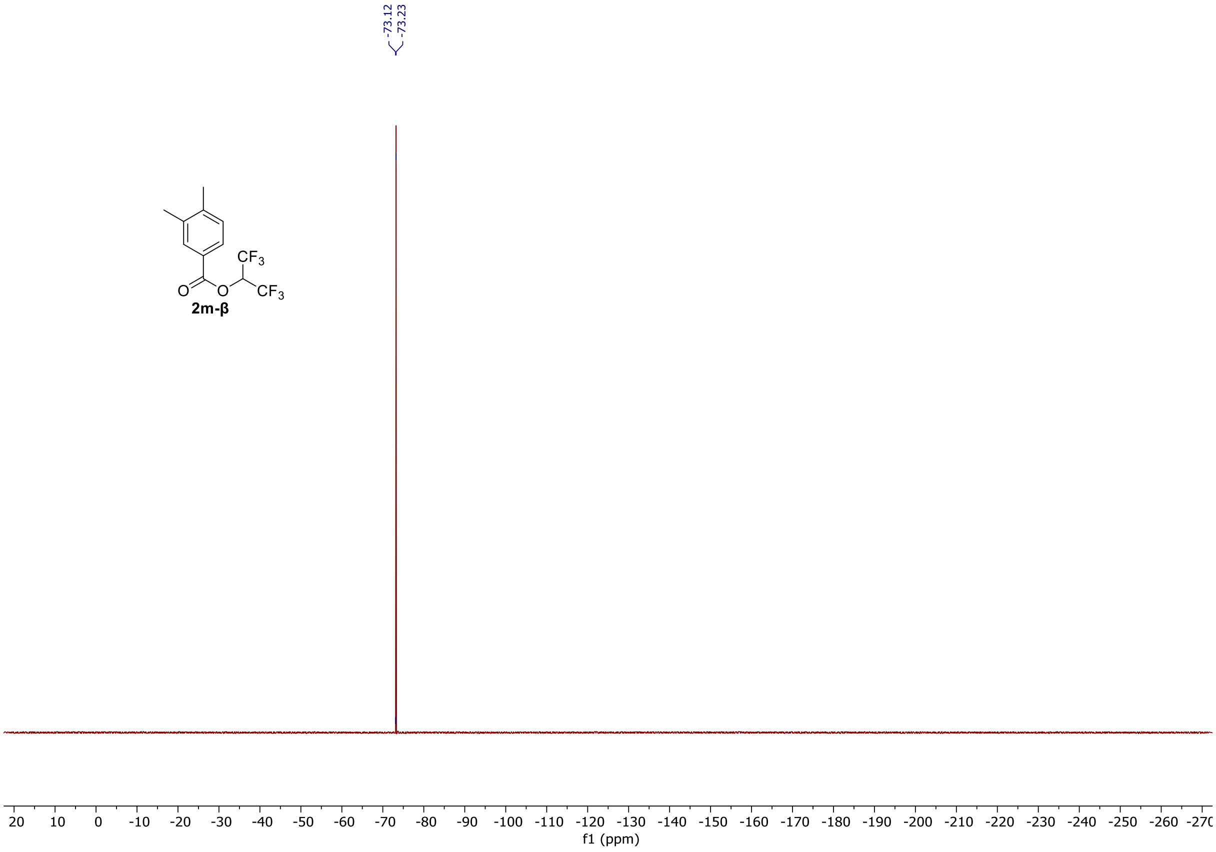

1,1,1,3,3,3-Hexafluoropropan-2-yl 3-(tert-butyldimethylsilyl)-5-((tert-butyldimethylsilyl)oxy)benzoate (2s)

^{1}H NMR spectrum in $CDCl_3$

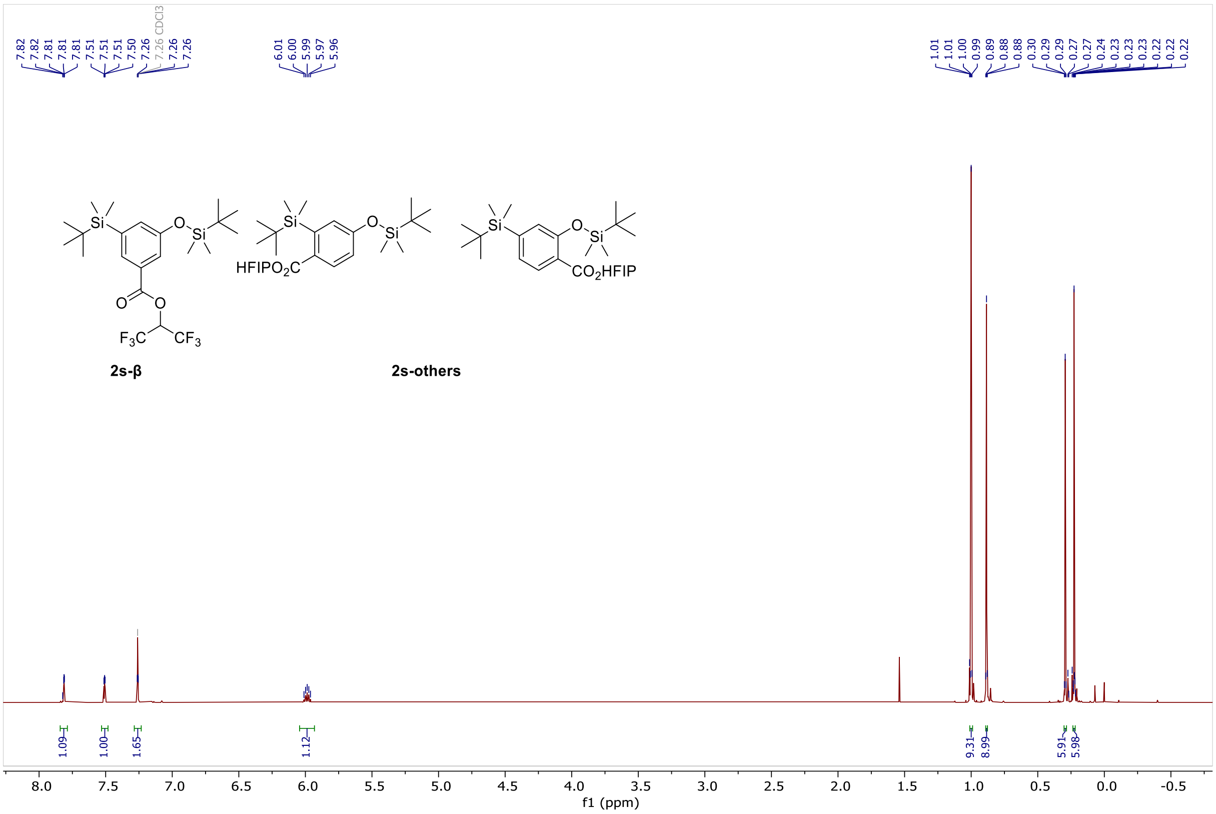

^{13}C NMR spectrum in $CDCl_3$

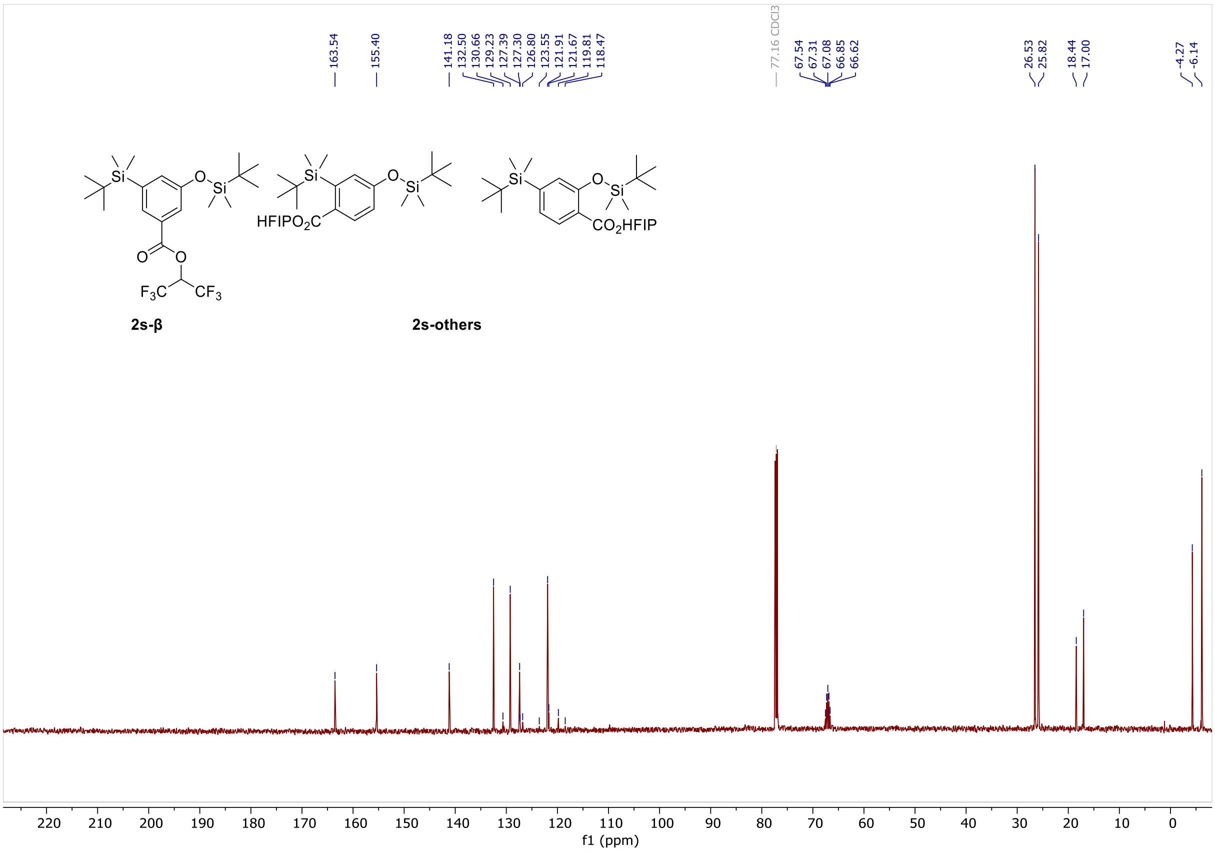

^{19}F NMR spectrum in $CDCl_3$

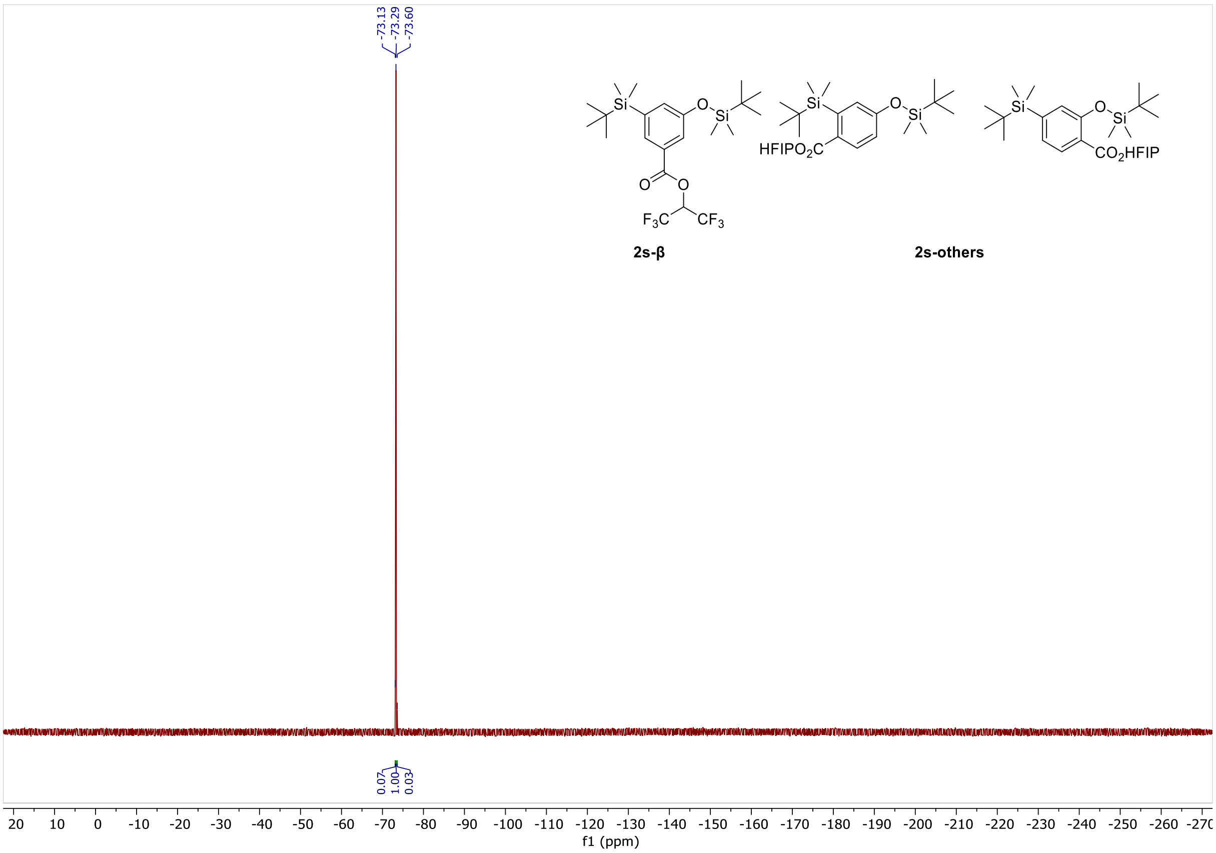

1,1,1,3,3,3-Hexafluoropropanyl dimethylbenzoate (2m) with reduced $AgNO_3$: Fraction 1

^{1}H NMR spectrum in $CDCl_3$

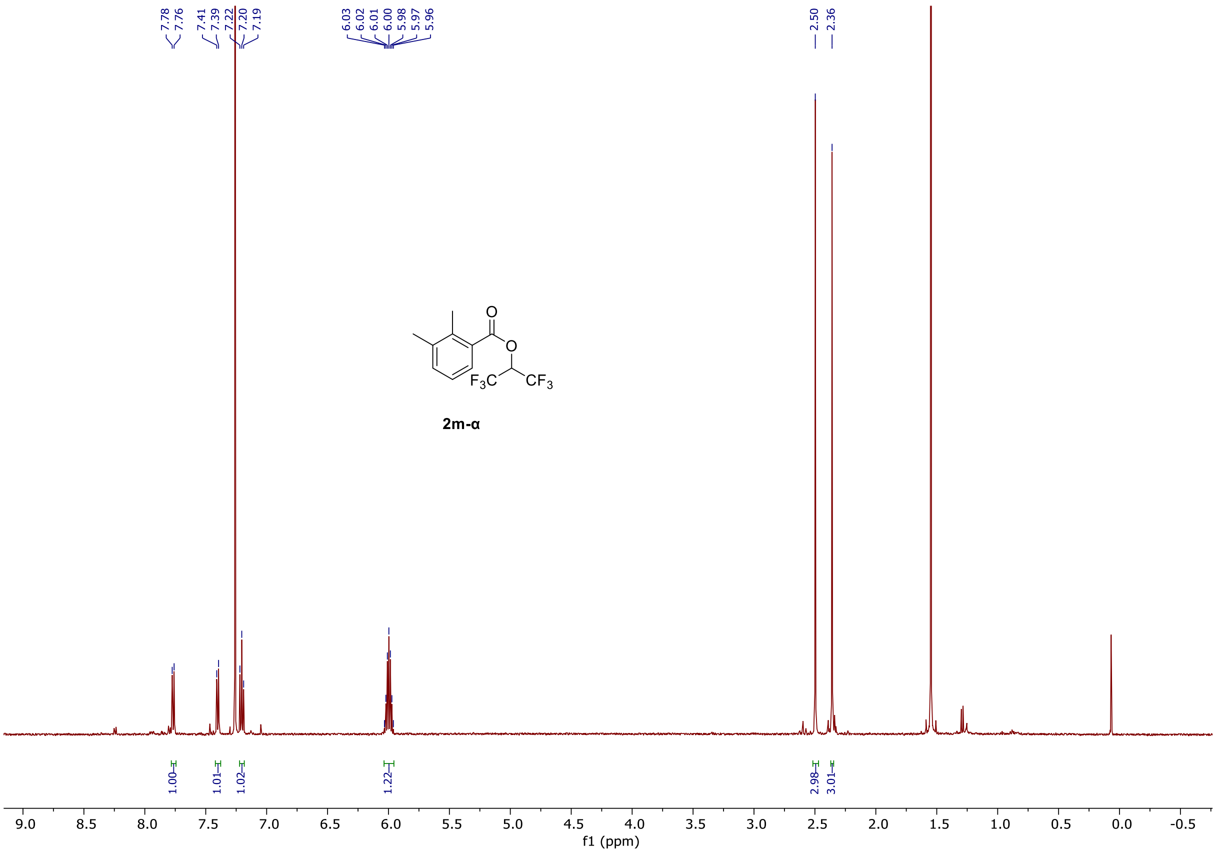

^{13}C NMR spectrum in $CDCl_3$

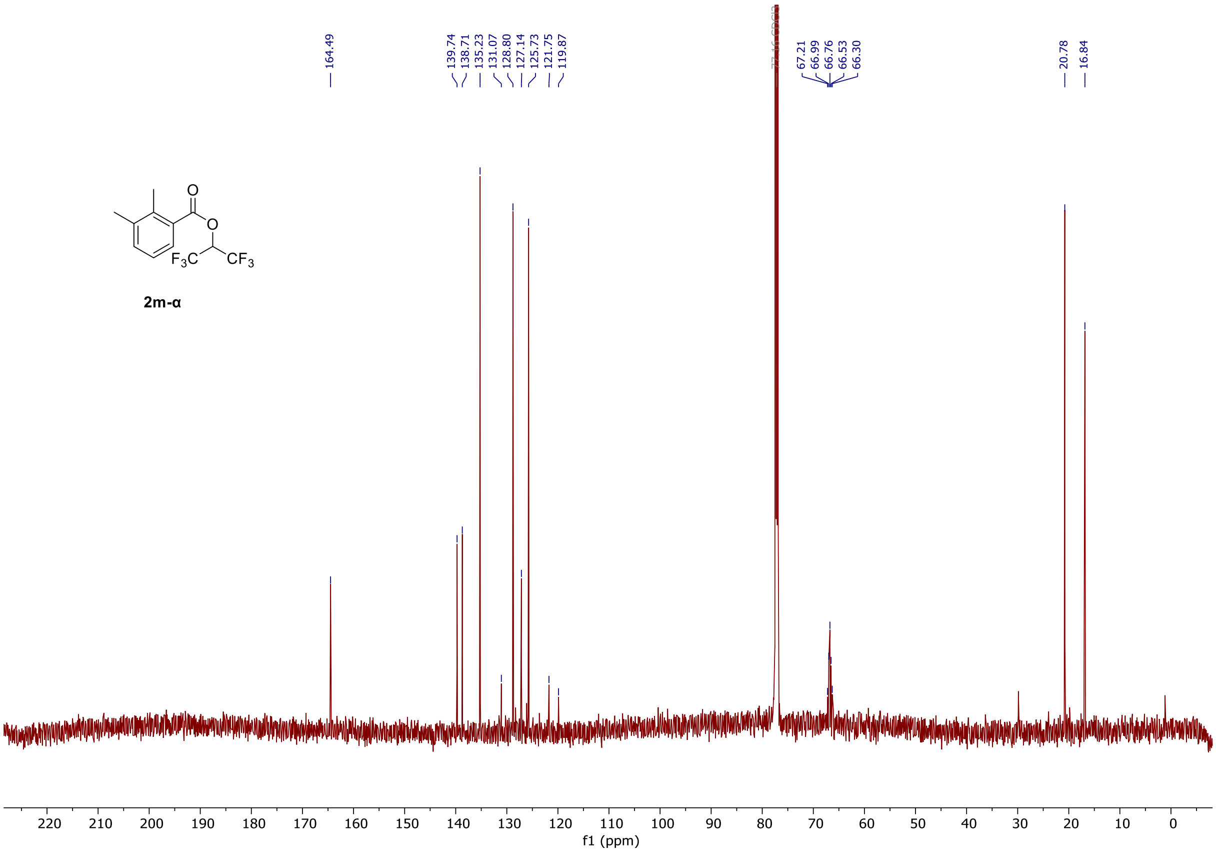

^{19}F NMR spectrum in $CDCl_3$

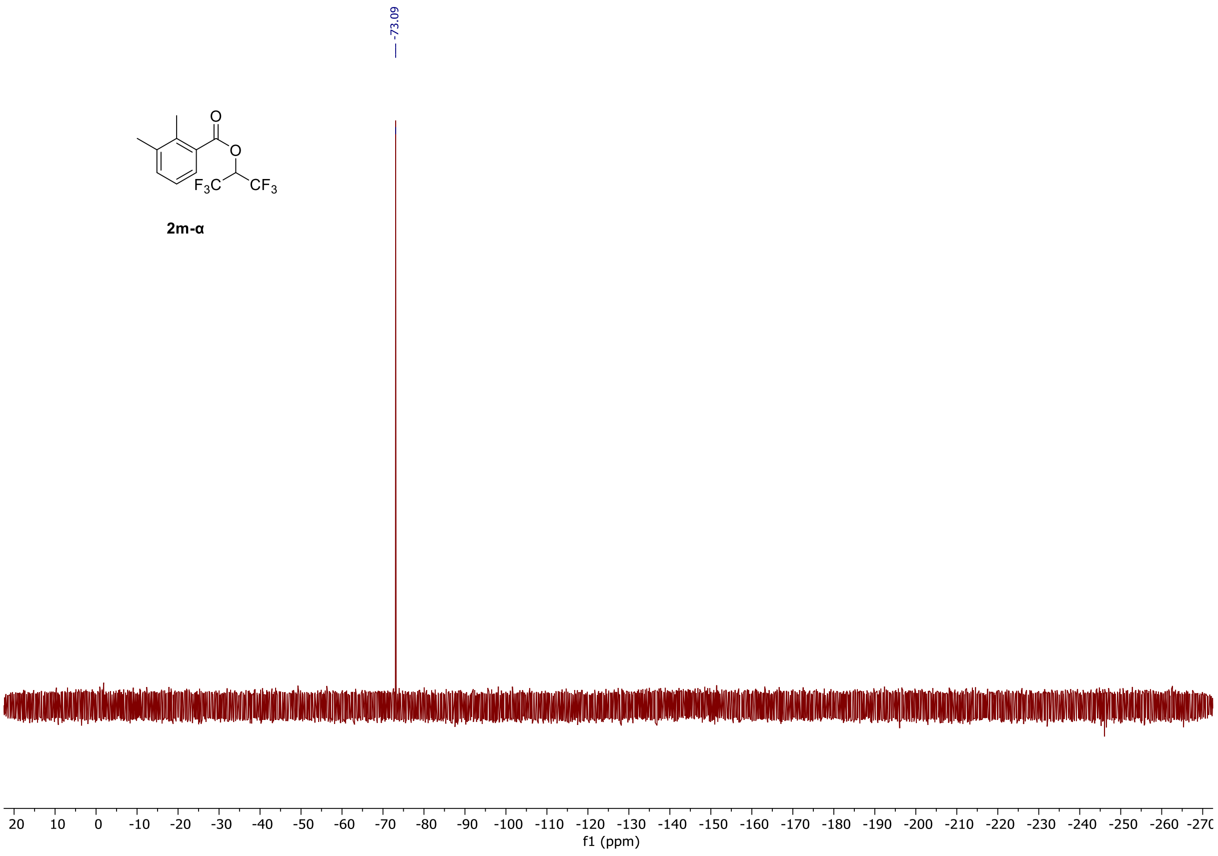

1,1,1,3,3,3-Hexafluoropropanyl dimethylbenzoate (2m) with reduced $AgNO_3$: Fraction 2

^1H NMR spectrum in $CDCl_3$

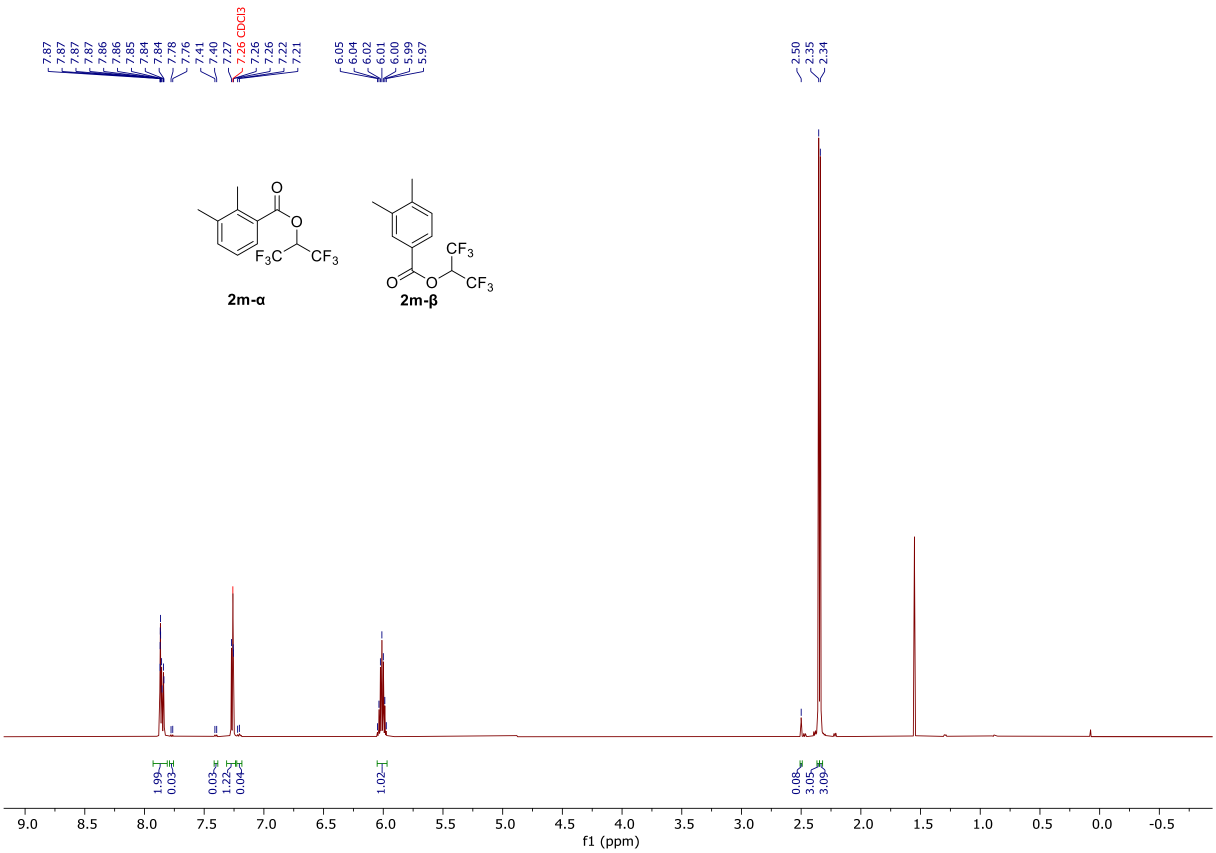

^{13}C NMR spectrum in $CDCl_3$

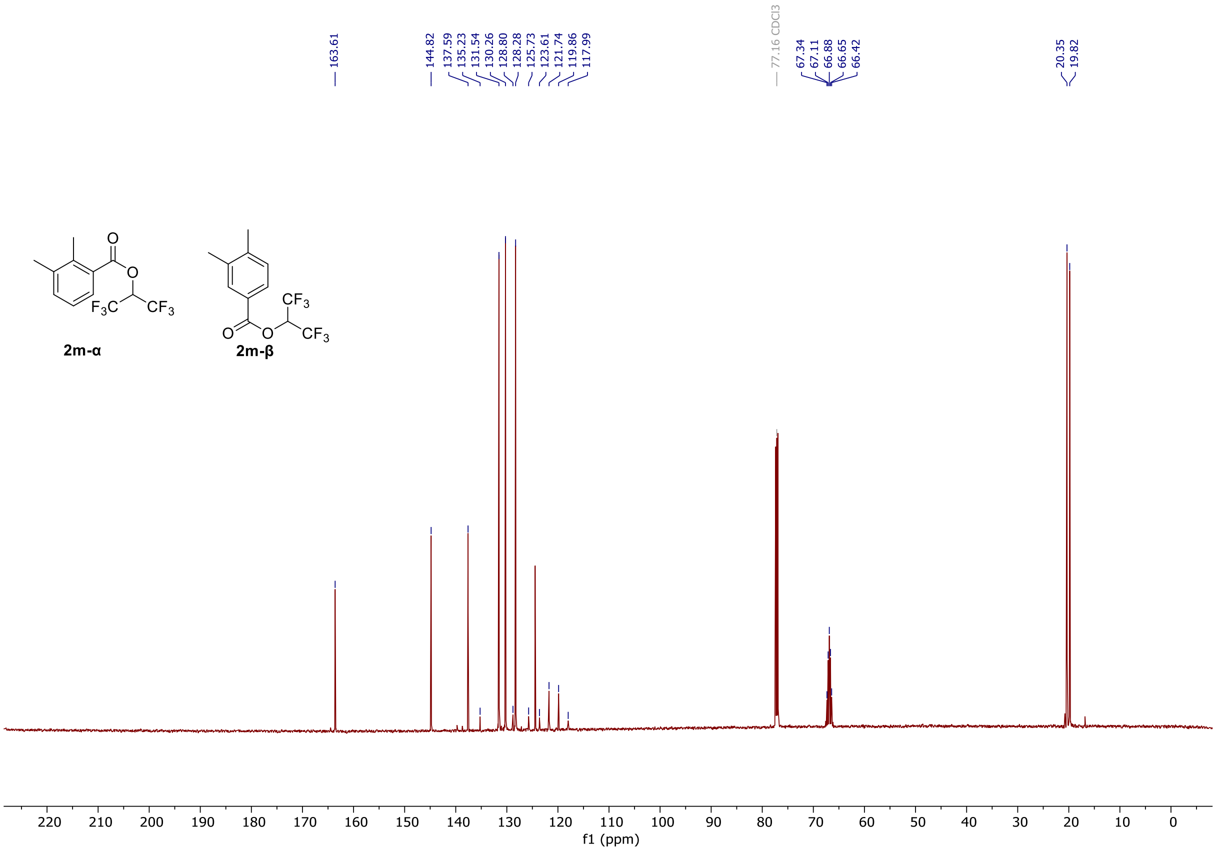

^{19}F NMR spectrum in $CDCl_3$

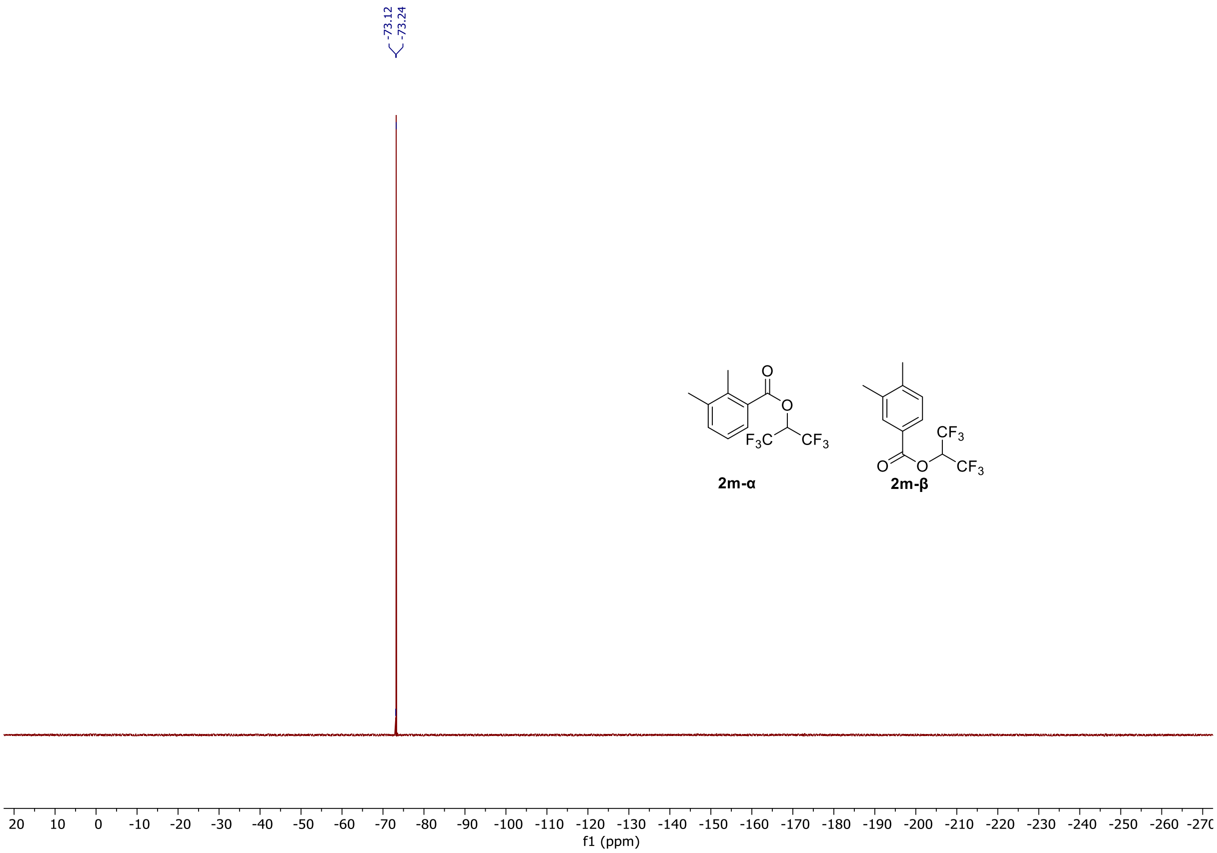

7.6.2 Synthetic Transformations of the HFIP-Ester Moiety

(3,4-Dimethylphenyl)methanol (5)

^{1}H NMR spectrum in $CDCl_3$

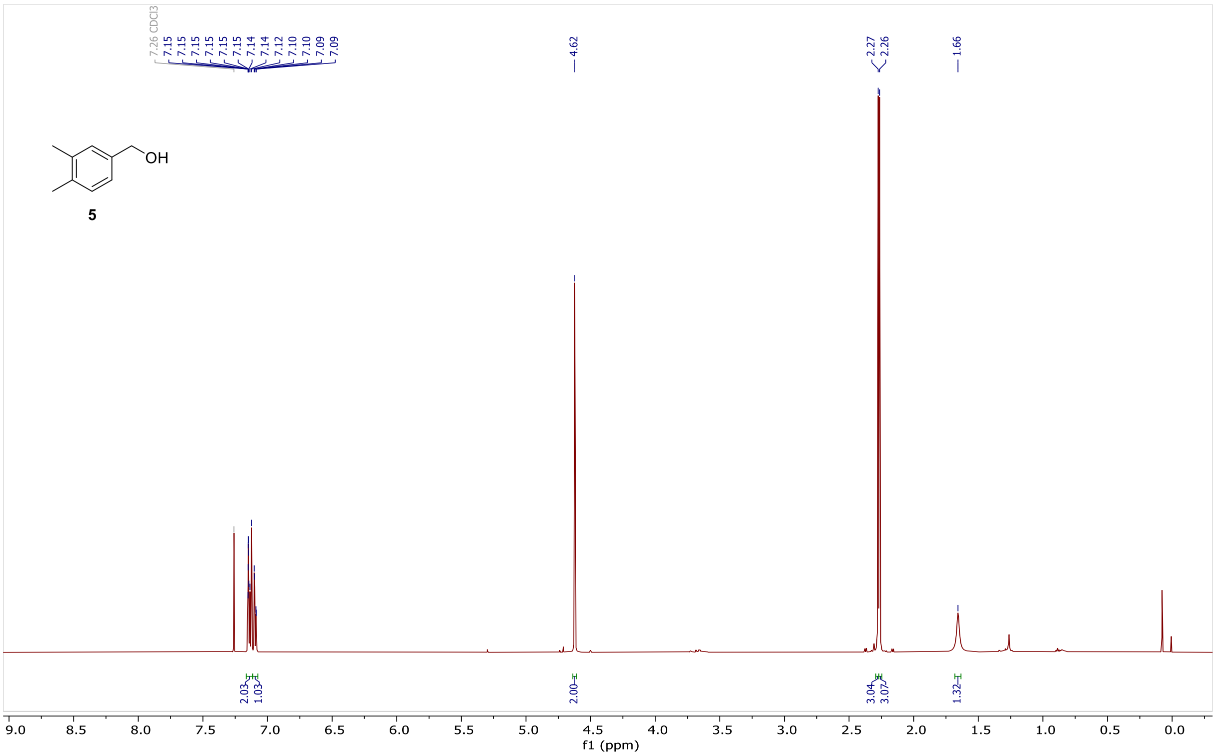

^{13}C NMR spectrum in $CDCl_3$

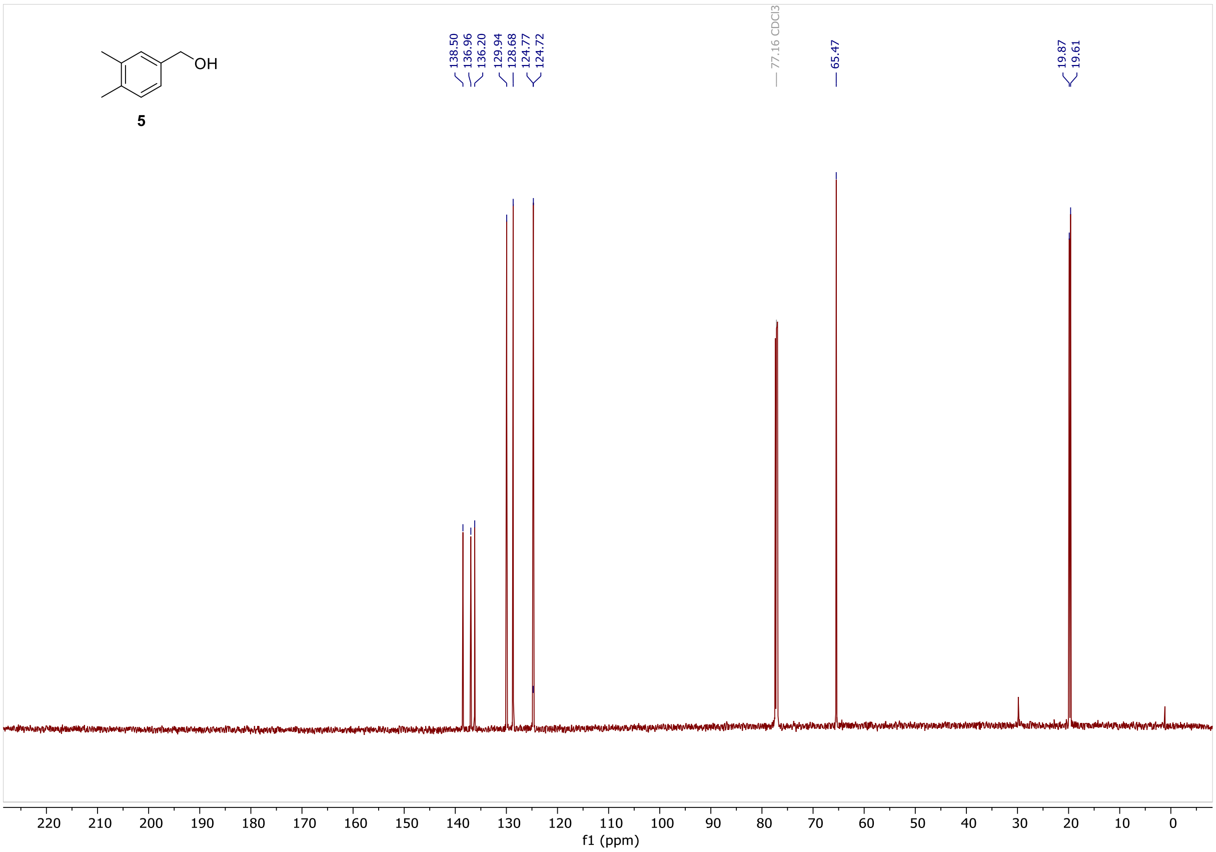

N-Benzyl-3,4-dimethylbenzamide (6)

^{1}H NMR spectrum in DMSO-d_6

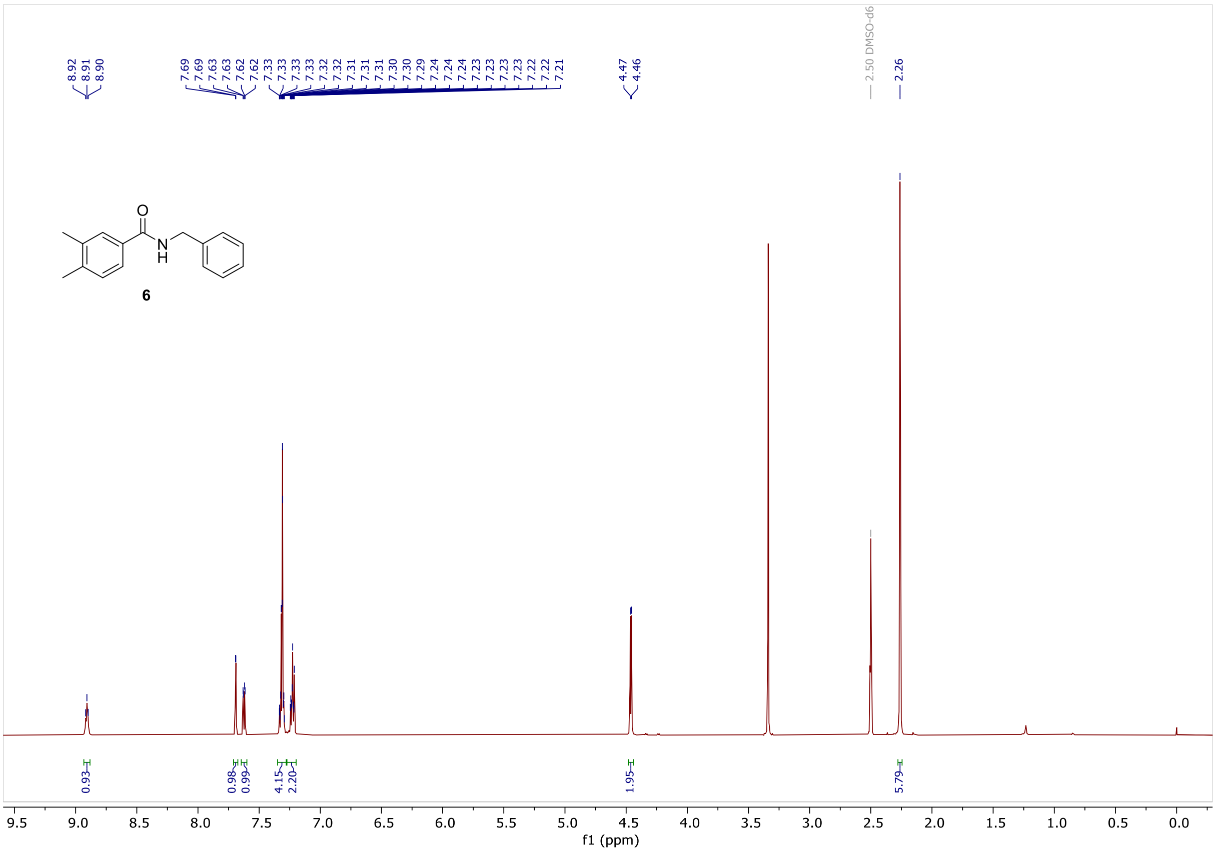

^{13}C NMR spectrum in DMSO-d_6

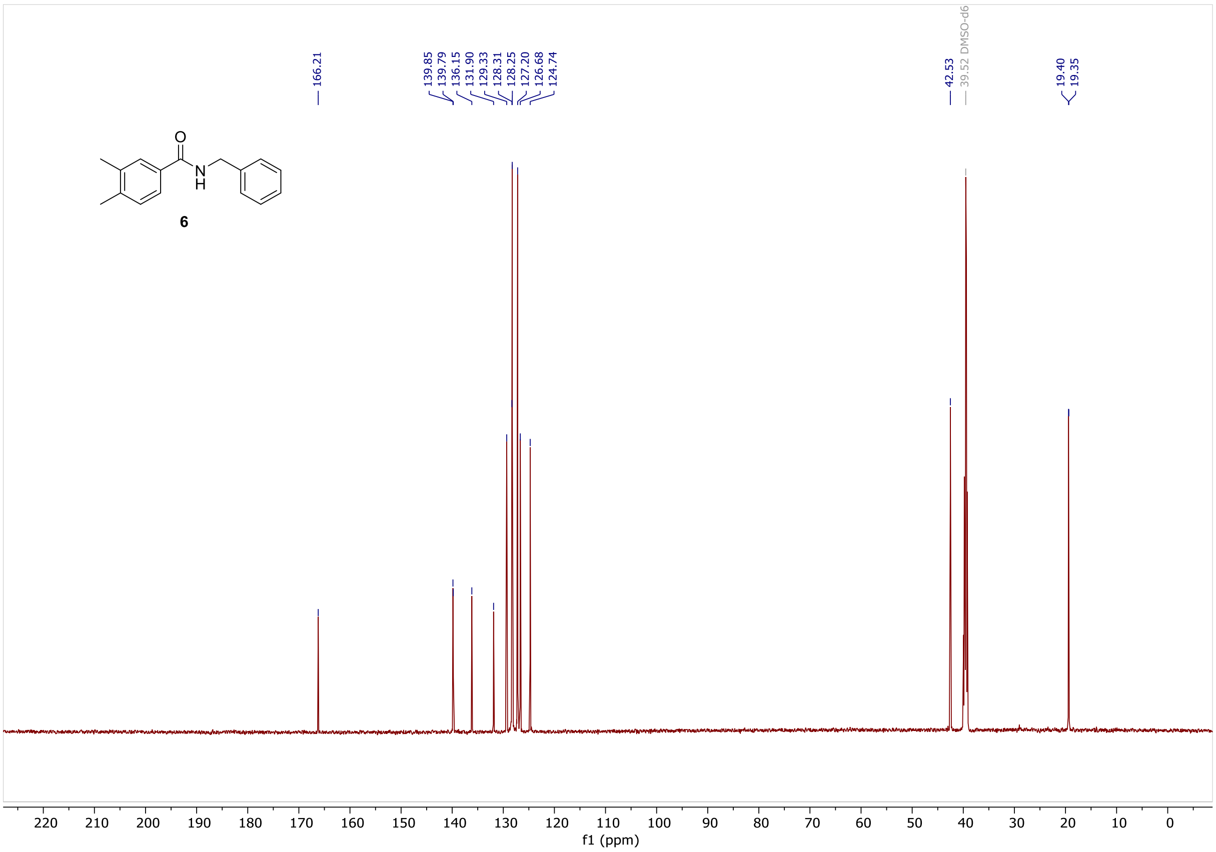

(3,4-dimethylphenyl)(morpholino)methanone (7a)

^{1}H NMR spectrum in $CDCl_3$

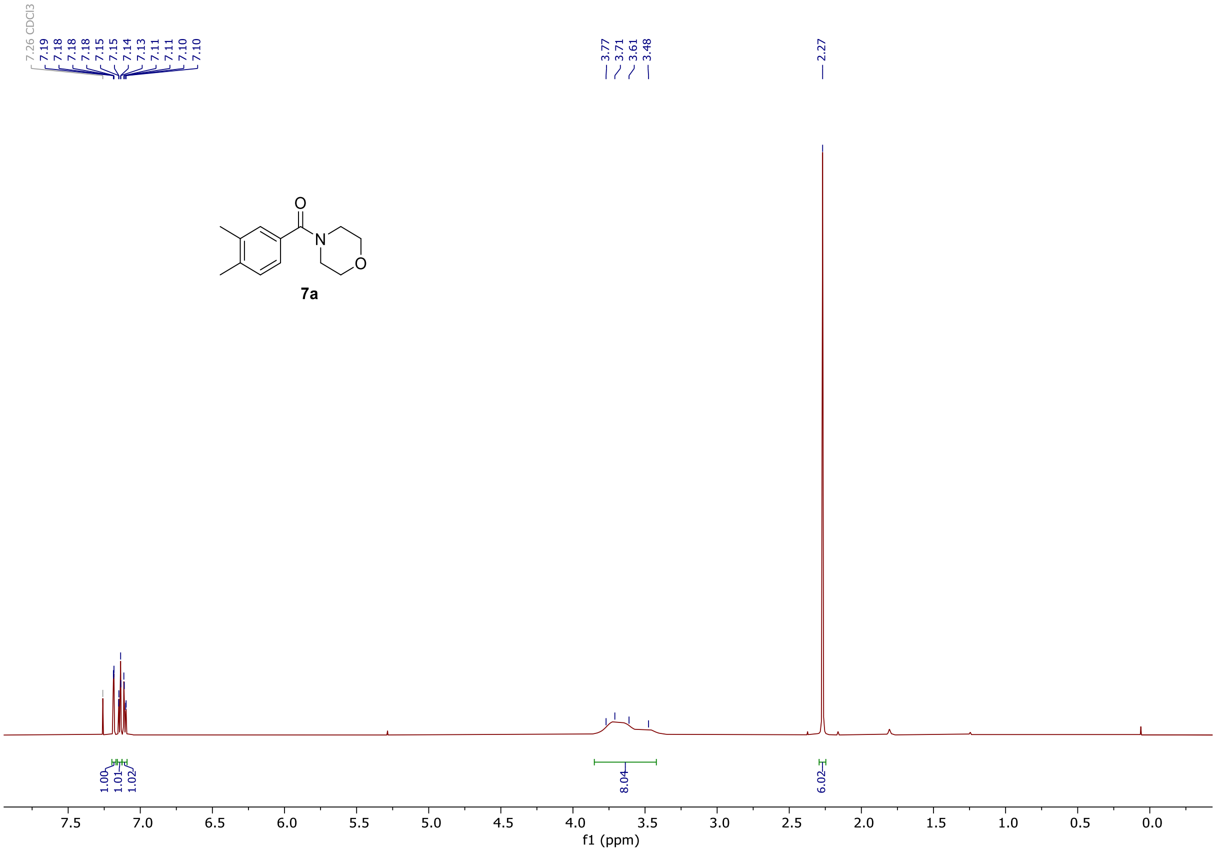

^{13}C NMR spectrum in $CDCl_3$

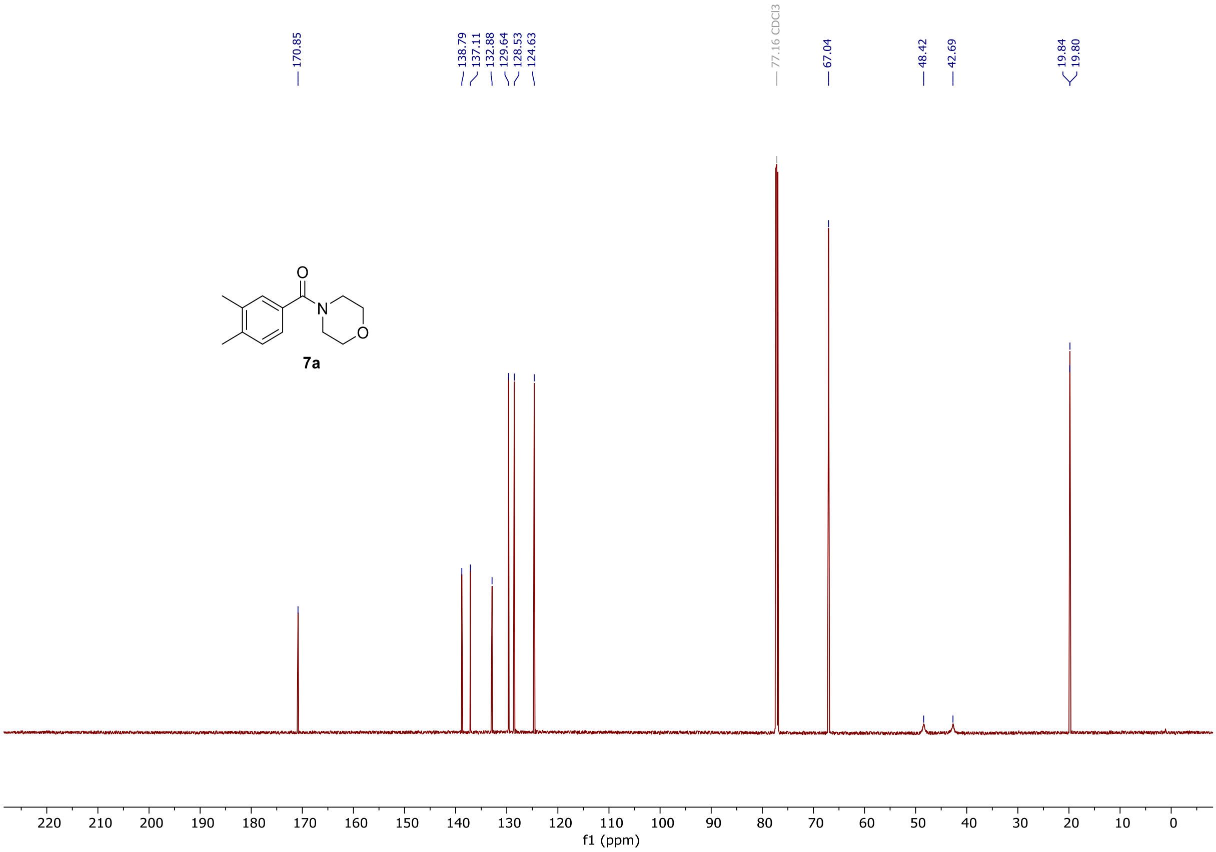

3,4-Dimethylbenzophenone (7b)

^{1}H NMR spectrum in $CDCl_3$

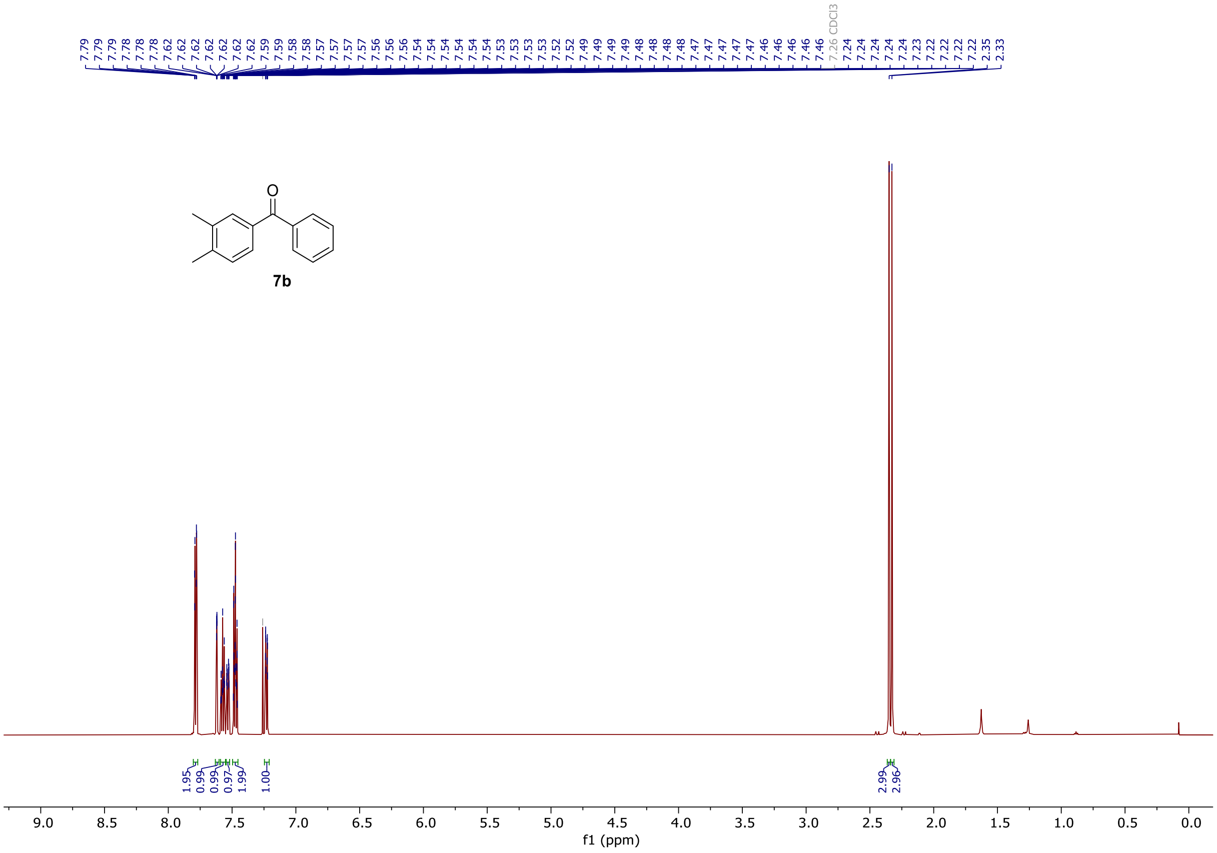

^{13}C NMR spectrum in $CDCl_3$

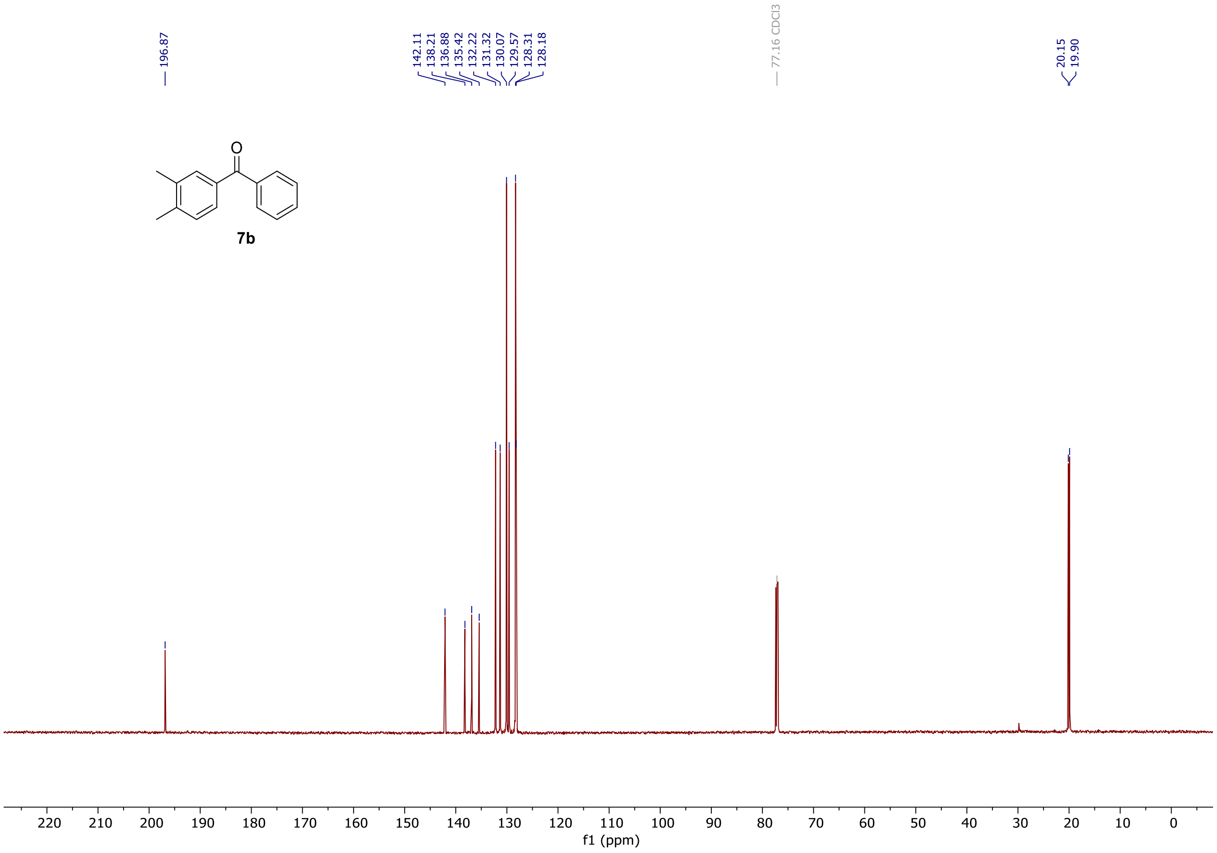

(3,4-dimethylphenyl)diphenylmethanol (8)

^{1}H NMR spectrum in $CDCl_3$

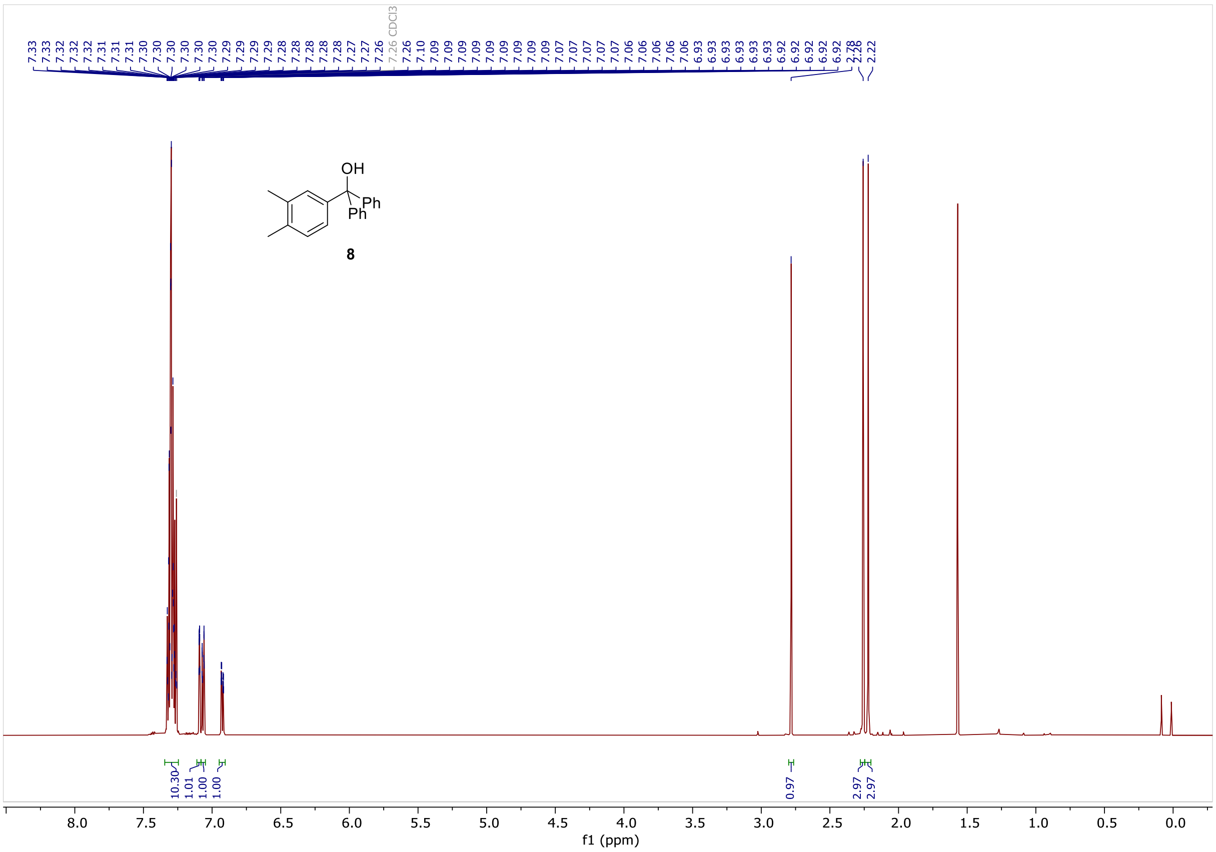

^{13}C NMR spectrum in $CDCl_3$

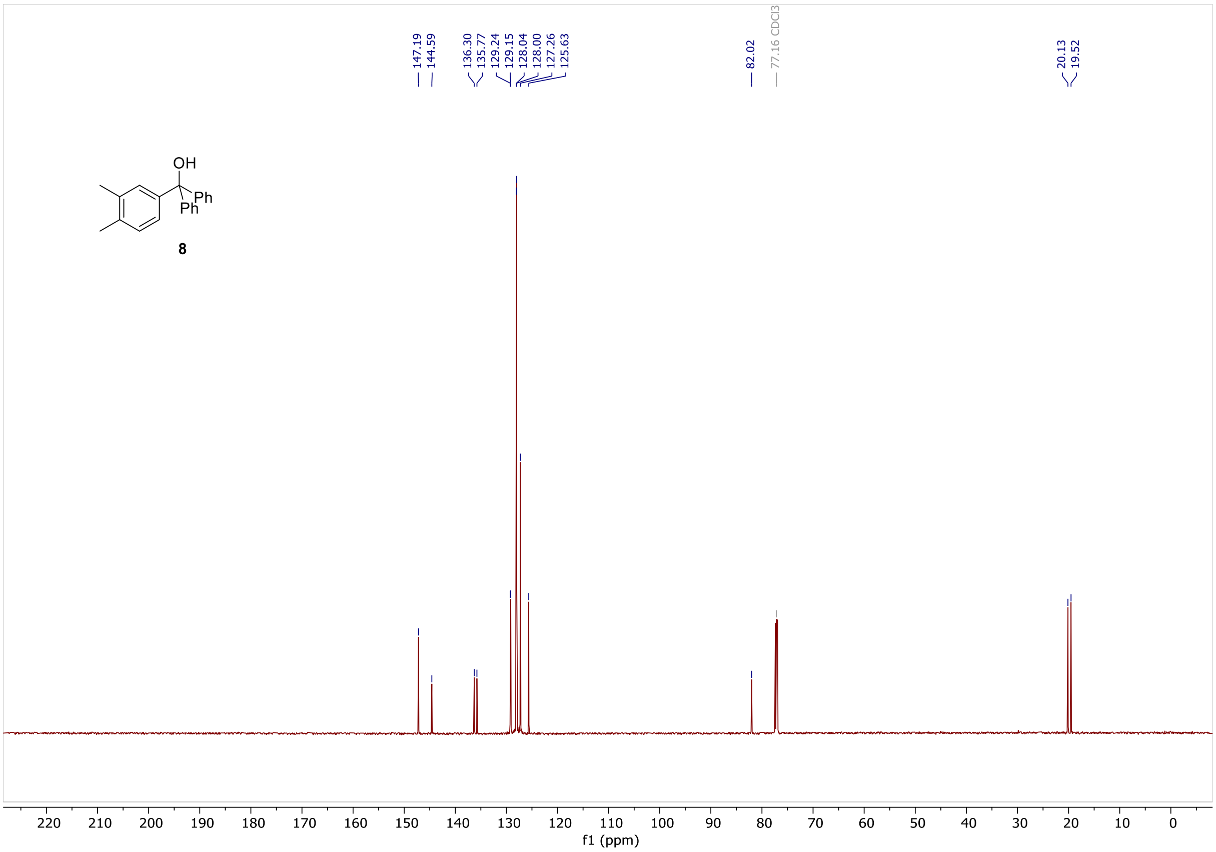

Methyl 3,4-dimethylbenzoate (9)

^{1}H NMR spectrum in $CDCl_3$

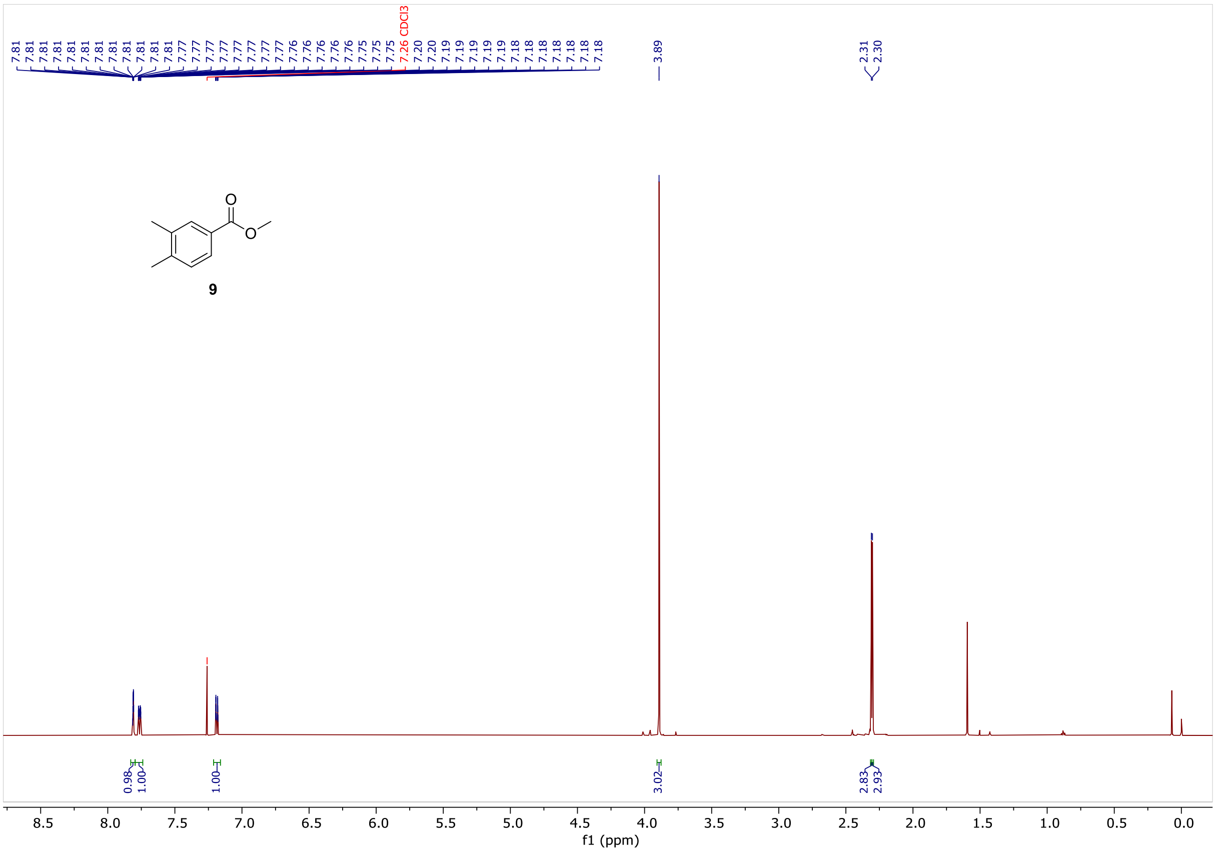

^{13}C NMR spectrum in $CDCl_3$

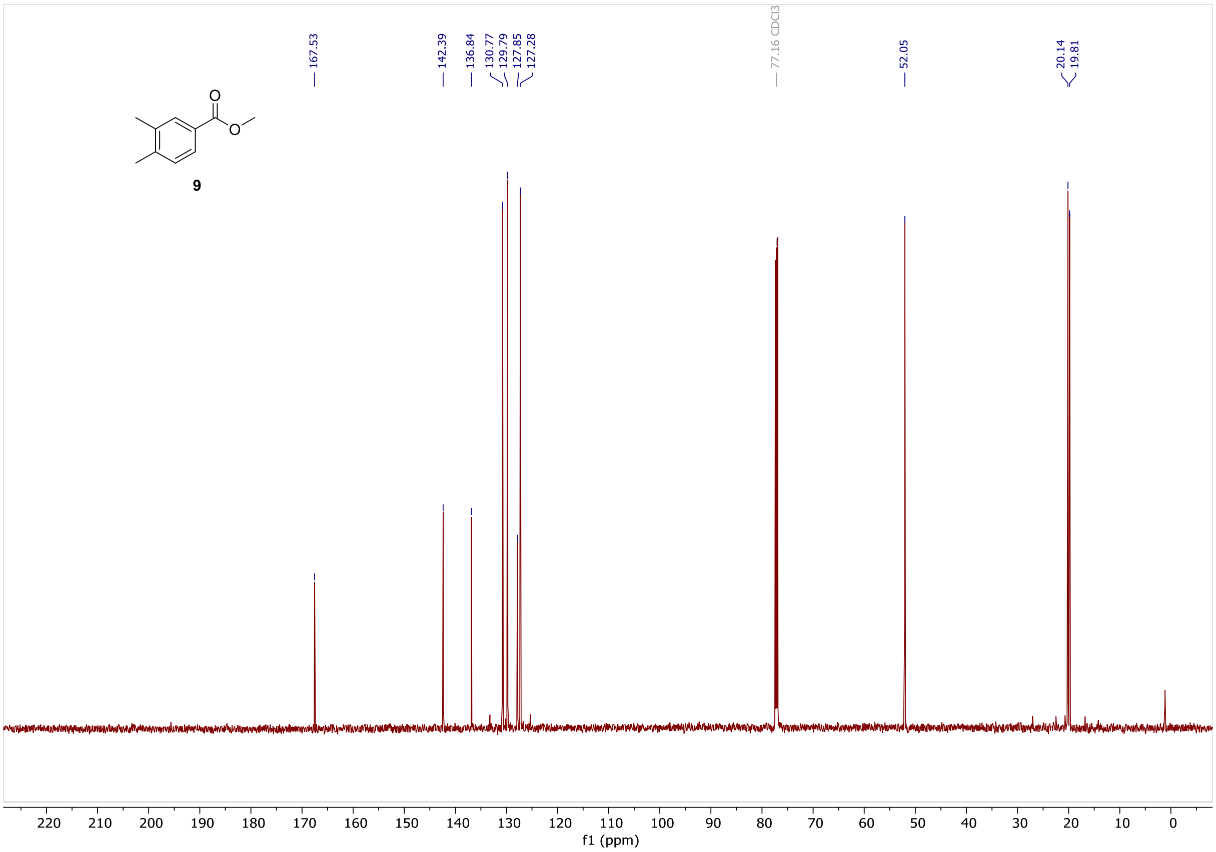

7.6.3 Net meta-Selective Carboxylation of Protected Phenols

3-(tert-Butyldimethylsilyl)-5-hydroxybenzoic acid (10)

^{1}H NMR spectrum in DMSO-d_6

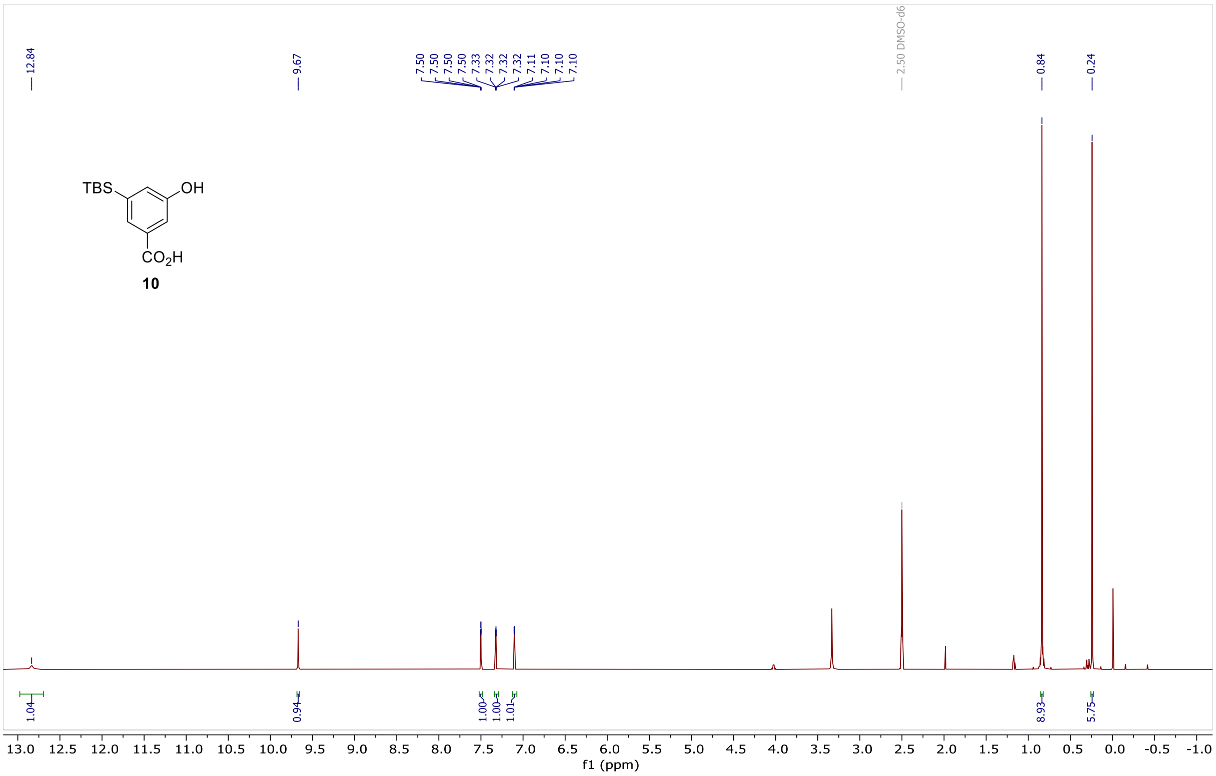

^{13}C NMR spectrum in DMSO-d_6

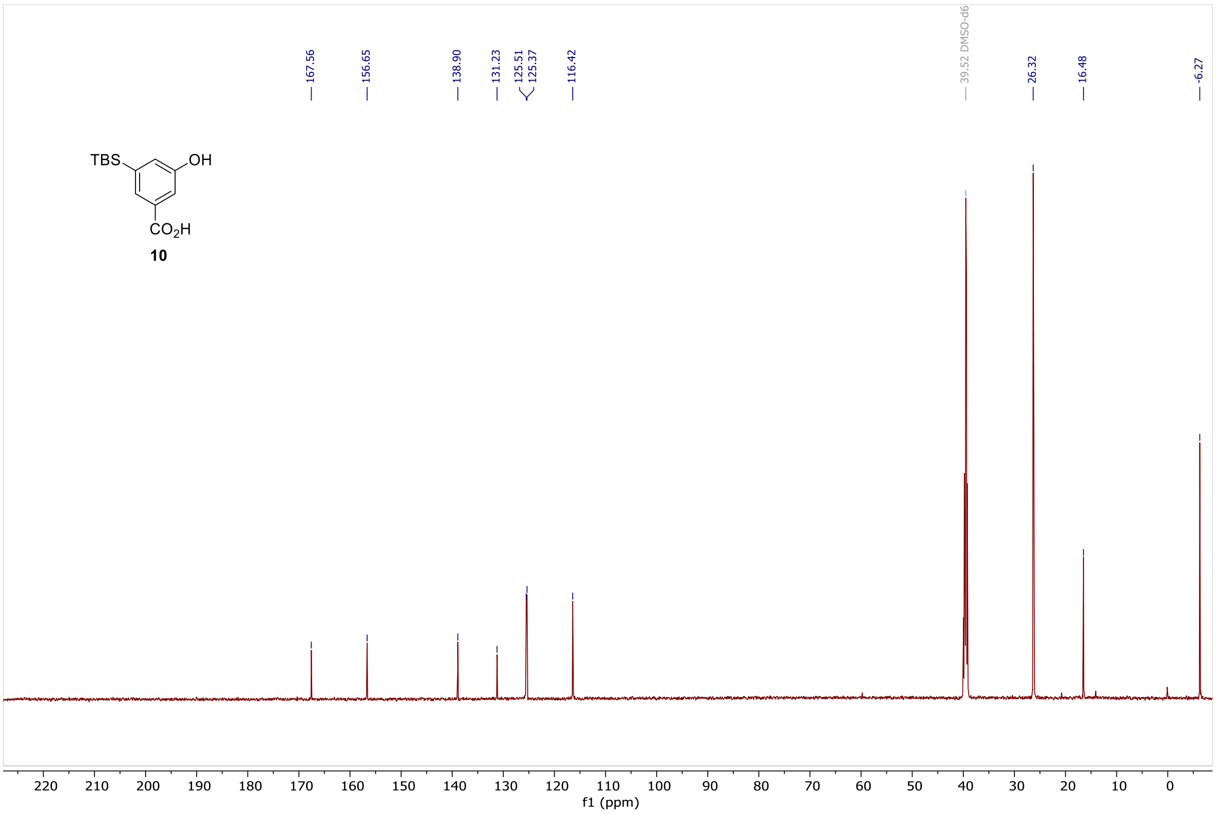

8. References

(1) Uttry, A.; Mal, S.; van Gemmeren, M. Late-Stage β-C(sp^3)-H Deuteration of Carboxylic Acids. *J. Am. Chem. Soc.* **2021**, *143*, 10895–10901.

(2) Fulmer, G. R.; Miller, A. J. M.; Sherden, N. H.; Gottlieb, H. E.; Nudelman, A.; Stoltz, B. M.; Bercaw, J. E.; Goldberg, K. I. NMR Chemical Shifts of Trace Impurities: Common Laboratory Solvents, Organics, and Gases in Deuterated Solvents Relevant to the Organometallic Chemist. *Organometallics* **2010**, *29*, 2176–2179.

(3) Zhuang, Z.; Liu, S.; Cheng, J.-T.; Yeung, K.-S.; Qiao, J. X.; Meanwell, N. A.; Yu, J.-Q. Ligand-Enabled β-C(sp^3)-H Lactamization of Tosyl-Protected Aliphatic Amides Using a Practical Oxidant. *Angew. Chem. Int. Ed.* **2022**, *61*, e202207354.

(4) Lu, P.; Burgenson, W. R.; Simmons, B. J.; Liu, S.; Yeung, K.-S.; Qiao, J. X.; Yu, J.-Q. Synthesis of Chiral Saturated Heterocycles Bearing Quaternary Centers via Enantioselective β-C(sp^3)-H Activation of Lactams. *J. Am. Chem. Soc.* **2025**, *147*, 1427–1433.

(5) Yuan, C.-H.; Wang, X.-X.; Jiao, L. Ligand-Enabled Palladium(II)-Catalyzed Enantioselective β-C(sp^3)-H Arylation of Aliphatic Tertiary Amides. *Angew. Chem. Int. Ed.* **2023**, *62*, e202300854.

(6) Fässler, J.; McCubbin, J. A.; Roglans, A.; Kimachi, T.; Hollett, J. W.; Kunz, R. W.; Tinkl, M.; Zhang, Y.; Wang, R.; Campbell, M.; Snieckus, V. Highly enantioselective (-)-sparteine-mediated lateral metalation-functionalization of remote silyl protected ortho-ethyl *N,N*-dialkyl aryl *O*-carbamates. *J. Org. Chem.* **2015**, *80*, 3368–3386.

(7) Liang, D.-D.; Streefkerk, D. E.; Jordaan, D.; Wagemakers, J.; Baggerman, J.; Zuilhof, H. Silicon-Free SuFEx Reactions of Sulfonimidoyl Fluorides: Scope, Enantioselectivity, and Mechanism. *Angew. Chem. Int. Ed.* **2020**, *59*, 7494–7500.

(8) Liang, K.; Li, X.; Wei, D.; Jin, C.; Liu, C.; Xia, C. Deprotection of benzyl-derived groups via photochemically mesolytic cleavage of C–N and C–O bonds. *Chem* **2023**, *9*, 511–522.

(9) Takale, B. S.; Thakore, R. R.; Handa, S.; Gallou, F.; Reilly, J.; Lipshutz, B. H. A new, substituted palladacycle for ppm level Pd-catalyzed Suzuki-Miyaura cross couplings in water. *Chem. Sci.* **2019**, *10*, 8825–8831.

(10) Aravinda Kumar, K.; Venkateswarlu, V.; Vishwakarma, R.; Sawant, S. A Metal-Free Approach to Carboxylic Acids by Oxidation of Alkyl, Aryl, or Heteroaryl Alkyl Ketones or Arylalkynes. *Synthesis* **2015**, *47*, 3161–3168.

(11) Jia, R.; Wang, J.; Jiang, Y.; Ni, B.; Niu, T. Photocatalyzed oxidative cleavage of C-C bond to carbonyl compounds by a recyclable homogeneous carbon nitride semiconductor/aqueous system. *Org. Biomol. Chem.* **2022**, *20*, 8305–8312.

(12) Nair, V.; Varghese, V.; Paul, R. R.; Jose, A.; Sinu, C. R.; Menon, R. S. NHC catalyzed transformation of aromatic aldehydes to acids by carbon dioxide: an unexpected reaction. *Org. Lett.* **2010**, *12*, 2653–2655.

(13) Moriyama, K.; Takemura, M.; Togo, H. Direct and selective benzylic oxidation of alkylarenes via C-H abstraction using alkali metal bromides. *Org. Lett.* **2012**, *14*, 2414–2417.

(14) Armstrong, D. R.; Garden, J. A.; Kennedy, A. R.; Leenhouts, S. M.; Mulvey, R. E.; O'Keefe, P.; O'Hara, C. T.; Steven, A. Evaluating cis-2,6-dimethylpiperidide (cis-DMP) as a base component in lithium-mediated zincation chemistry. *Chem. Eur. J.* **2013**, *19*, 13492–13503.

(15) Han, W.; Jin, F.; Zhou, Q. Ligand-Free Palladium-Catalyzed Hydroxycarbonylation of Aryl Halides under Ambient Conditions: Synthesis of Aromatic Carboxylic Acids and Aromatic Esters. *Synthesis* **2015**, *47*, 1861–1868.

(16) Liu, M.; Li, C.-J. Catalytic Fehling's Reaction: An Efficient Aerobic Oxidation of Aldehyde Catalyzed by Copper in Water. *Angew. Chem. Int. Ed.* **2016**, *55*, 10806–10810.

(17) Borthakur, I.; Joshi, A.; Kumari, S.; Kundu, S. Metal-Free Visible-Light Induced Oxidative Cleavage of C(sp^3)-C, and C(sp^3)-N Bonds of Nitriles, Alcohols, and Amines. *Chem. Eur. J.* **2024**, *30*, e202303295.
(18) Wang, Y.; Zhao, Z.; Pan, D.; Wang, S.; Jia, K.; Ma, D.; Yang, G.; Xue, X.-S.; Qiu, Y. Metal-Free Electrochemical Carboxylation of Organic Halides in the Presence of Catalytic Amounts of an Organomediator. *Angew. Chem. Int. Ed.* **2022**, *61*, e202210201.
(19) Lv, W.; Wen, S.; Liu, J.; Cheng, G. Palladium-Catalyzed ortho-C-H Methylation of Benzoic Acids. *J. Org. Chem.* **2019**, *84*, 9786–9791.
(20) Mei, T.-S.; Giri, R.; Maugel, N.; Yu, J.-Q. Pd(II)-catalyzed monoselective ortho halogenation of C-H bonds assisted by counter cations: a complementary method to directed ortho lithiation. *Angew. Chem. Int. Ed.* **2008**, *47*, 5215–5219.
(21) Gross, U.; Koos, P.; O'Brien, M.; Polyzos, A.; Ley, S. V. A General Continuous Flow Method for Palladium Catalysed Carbonylation Reactions Using Single and Multiple Tube-in-Tube Gas-Liquid Microreactors. *Eur. J. Org. Chem.* **2014**, *2014*, 6418–6430.
(22) Shil, A. K.; Kumar, S.; Reddy, C. B.; Dadhwal, S.; Thakur, V.; Das, P. Supported Palladium Nanoparticle-Catalyzed Carboxylation of Aryl Halides, Alkenylsilanes, and Organoboronic Acids Employing Oxalic Acid as the C1 Source. *Org. Lett.* **2015**, *17*, 5352–5355.
(23) Wang, B.; Sun, H.-X.; Sun, Z.-H.; Lin, G.-Q. Direct B -Alkyl Suzuki–Miyaura Cross-Coupling of Trialkyl- boranes with Aryl Bromides in the Presence of Unmasked Acidic or Basic Functions and Base-Labile Protections under Mild Non-Aqueous Conditions. *Adv. Synth. Catal.* **2009**, *351*, 415–422.
(24) Urgoitia, G.; SanMartin, R.; Herrero, M. T.; Domínguez, E. An outstanding catalyst for the oxygen-mediated oxidation of arylcarbinols, arylmethylene and arylacetylene compounds. *Chem. Commun.* **2015**, *51*, 4799–4802.
(25) Wang, R.; Tang, Y.; Xu, M.; Meng, C.; Li, F. Transfer Hydrogenation of Aldehydes and Ketones with Isopropanol under Neutral Conditions Catalyzed by a Metal-Ligand Bifunctional Catalyst Cp*Ir(2,2'-bpyO)(H_2O). *J. Org. Chem.* **2018**, *83*, 2274–2281.
(26) Caldwell, N.; Jamieson, C.; Simpson, I.; Watson, A. J. B. Catalytic amidation of unactivated ester derivatives mediated by trifluoroethanol. *Chem. Commun.* **2015**, *51*, 9495–9498.
(27) Papp, M.; Szabó, P.; Srankó, D.; Sáfrán, G.; Kollár, L.; Skoda-Földes, R. Mono- and double carbonylation of aryl iodides with amine nucleophiles in the presence of recyclable palladium catalysts immobilised on a supported dicationic ionic liquid phase. *RSC Adv.* **2017**, *7*, 44587–44597.
(28) Martín, R.; Romea, P.; Tey, C.; Urpí, F.; Vilarrasa, J. Simple and Efficient Preparation of Ketones from Morpholine Amides. *Synlett* **1997**, *12*, 1414–1416.
(29) Meng, M.; Yang, L.; Cheng, K.; Qi, C. Pd(II)-Catalyzed Denitrogenative and Desulfinative Addition of Arylsulfonyl Hydrazides with Nitriles. *J. Org. Chem.* **2018**, *83*, 3275–3284.
(30) Good, J. A. D.; Wang, F.; Rath, O.; Kaan, H. Y. K.; Talapatra, S. K.; Podgórski, D.; MacKay, S. P.; Kozielski, F. Optimized S-trityl-L-cysteine-based inhibitors of kinesin spindle protein with potent in vivo antitumor activity in lung cancer xenograft models. *J. Med. Chem.* **2013**, *56*, 1878–1893.
(31) Kelly, C. B.; Mercadante, M. A.; Wiles, R. J.; Leadbeater, N. E. Oxidative esterification of aldehydes using a recyclable oxoammonium salt. *Org. Lett.* **2013**, *15*, 2222–2225.
(32) Ghosh, K.; Iqubal, M. A.; Molla, R. A.; Mishra, A.; Kamaluddin, K.; Islam, S. M. Direct oxidative esterification of alcohols and hydration of nitriles catalyzed by a reusable silver nanoparticle grafted onto mesoporous polymelamine formaldehyde (AgNPs@mPMF). *Catal. Sci. Technol.* **2015**, *5*, 1606–1622.
(33) Nguyen, T. T.; Grigorjeva, L.; Daugulis, O. Aminoquinoline-directed, cobalt-catalyzed carbonylation of sulfonamide sp^2 C-H bonds. *Chem. Commun.* **2017**, *53*, 5136–5138.

(34) Åkerbladh, L.; Odell, L.; Larhed, M. Palladium-Catalyzed Molybdenum Hexacarbonyl-Mediated Gas-Free Carbonylative Reactions. *Synlett* **2019**, *30*, 141–155.
(35) Abbott, A. P.; Malkov, A. V.; Zimmermann, N.; Raynor, J. B.; Ahmed, G.; Steele, J.; Kočovský, P. Oxidation of Molybdenum(0) and Tungsten(0) Carbonyl Complexes with Silver Triflate. *Organometallics* **1997**, *16*, 3690–3695.
(36) Wedi, P.; Farizyan, M.; Bergander, K.; Mück-Lichtenfeld, C.; van Gemmeren, M. Mechanism of the Arene-Limited Nondirected C-H Activation of Arenes with Palladium*. *Angew. Chem. Int. Ed.* **2021**, *60*, 15641–15649.
(37) Anderson, G. K.; Cross, R. J. Carbonyl-insertion reactions of square-planar complexes. *Acc. Chem. Res.* **1984**, *17*, 67–74.
(38) Cavell, K. J. Recent fundamental studies on migratory insertion into metal-carbon bonds. *Coord. Chem. Rev.* **1996**, *155*, 209–243.
(39) Barnard, C. F. J. Palladium-Catalyzed Carbonylation—A Reaction Come of Age. *Organometallics* **2008**, *27*, 5402–5422.
(40) Sang, R.; Hu, Y.; Razzaq, R.; Jackstell, R.; Franke, R.; Beller, M. State-of-the-art palladium-catalyzed alkoxycarbonylations. *Org. Chem. Front.* **2021**, *8*, 799–811.
(41) Wang, P.; Verma, P.; Xia, G.; Shi, J.; Qiao, J. X.; Tao, S.; Cheng, P. T. W.; Poss, M. A.; Farmer, M. E.; Yeung, K.-S.; Yu, J.-Q. Ligand-accelerated non-directed C-H functionalization of arenes. *Nature* **2017**, *551*, 489–493.
(42) Fujiwara, Y.; Kawauchi, T.; Taniguchi, H. Palladium-promoted one-step carboxylation of aromatic compounds with carbon monoxide. *J. Chem. Soc., Chem. Commun.* **1980**, 220.
(43) Jintoku, T.; Fujiwara, Y.; Kawata, I.; Kawauchi, T.; Taniguchi, H. Palladium-catalyzed synthesis of aromatic acids from carbon monoxide and aromatic compounds via the aromatic C-H bond activation. *J. Organomet. Chem.* **1990**, *385*, 297–306.
(44) Lu, W.; Yamaoka, Y.; Taniguchi, Y.; Kitamura, T.; Takaki, K.; Fujiwara, Y. Palladium(II)-catalyzed carboxylation of benzene and other aromatic compounds with carbon monoxide under very mild conditions. *J. Organomet. Chem.* **1999**, *580*, 290–294.
(45) Giri, R.; Yu, J.-Q. Synthesis of 1,2- and 1,3-dicarboxylic acids via Pd(II)-catalyzed carboxylation of aryl and vinyl C-H bonds. *J. Am. Chem. Soc.* **2008**, *130*, 14082–14083.
